# ZC3HC1 Is a Novel Inherent Component of the Nuclear Basket, Resident in a State of Reciprocal Dependence with TPR

**DOI:** 10.3390/cells10081937

**Published:** 2021-07-30

**Authors:** Philip Gunkel, Haruki Iino, Sandra Krull, Volker C. Cordes

**Affiliations:** Max Planck Institute for Biophysical Chemistry, D-37077 Göttingen, Germany; Philip.Gunkel@mpibpc.mpg.de (P.G.); h.iino@lms.mrc.ac.uk (H.I.); sandra.krull@uni-tuebingen.de (S.K.)

**Keywords:** NIPA, nuclear basket, nuclear interacting partner of ALK, nuclear pore complex, nucleoprotein TPR, translocated promoter region, ZC3HC1

## Abstract

The nuclear basket (NB) scaffold, a fibrillar structure anchored to the nuclear pore complex (NPC), is regarded as constructed of polypeptides of the coiled-coil dominated protein TPR to which other proteins can bind without contributing to the NB’s structural integrity. Here we report vertebrate protein ZC3HC1 as a novel inherent constituent of the NB, common at the nuclear envelopes (NE) of proliferating and non-dividing, terminally differentiated cells of different morphogenetic origin. Formerly described as a protein of other functions, we instead present the NB component ZC3HC1 as a protein required for enabling distinct amounts of TPR to occur NB-appended, with such ZC3HC1-dependency applying to about half the total amount of TPR at the NEs of different somatic cell types. Furthermore, pointing to an NB structure more complex than previously anticipated, we discuss how ZC3HC1 and the ZC3HC1-dependent TPR polypeptides could enlarge the NB’s functional repertoire.

## 1. Introduction

The NPC is a macromolecular structure that serves as the gateway for exchanging material between the nucleus and cytoplasm in eukaryotes. Its core structure forming the actual translocation channel through the NE is flanked by ring-like structures on its nuclear and cytoplasmic side. Both rings serve as anchor sites for fibrillar appendices arranged in eight-fold rotational symmetry but distinct from each other in appearance and composition. The rectilinear fibrils emanating from the nuclear ring (NR) appear to bifurcate at their distal ends and laterally interconnect with their neighbouring fibrils, thereby forming another ring-like structure, sometimes referred to as the terminal ring (TR), with the NPC-appended fibrils and the TR commonly regarded a structural entity, nowadays called the nuclear basket (NB). Initially observed and described as nuclear fibrils or as either fish trap- or basket-like structures associated with the NPCs in vertebrate tumour cells and oocytes (e.g., [1,2,3,4,5,6,7]), findings of NBs in insect salivary gland cells (e.g., [8]), in *Saccharomyces cerevisiae* [9], and in the protozoan *Dictyostelium* [10] indicate that the NB is common to many eukaryotes and probably present in a wide range of cell types.

Even though diverse functions in different cell types and species have been ascribed to the NB or some of its attributed components, a universal, cell type-spanning function of the NB remains to be unveiled (e.g., [11,12,13]). Furthermore, even though the NB’s inventory of proteins is nowadays often regarded as known, with various metazoan and yeast proteins having been proposed as NB components and displayed in divergent NB models over time, no generally accepted blueprint of their configurations as parts of the NB appears to have prevailed so far (e.g., [13,14,15,16,17,18,19,20,21,22]).

Some studies have described a large coiled-coil protein, named TPR in vertebrates (e.g., [23,24,25,26]), and its homologs in budding yeast, called Mlp1p and Mlp2p (e.g., [27,28]), as the central architectural elements of the NB’s fibrillar scaffold, being essential for the structural integrity of the NB in both species [11,12,29,30]. Furthermore, various proteins have been identified as binding partners of TPR, Mlps, or their homologs in other phyla. Some of these proteins have indeed been shown to colocalise with the NPC-attached homologs of TPR in interphase and to reside at these sites in a TPR-dependent manner. Not regarded as contributing to NB assembly or maintenance, these NB-appended proteins have been considered using the scaffold provided by TPR as either an operational platform or a storage place at the NPC. Among these proteins are the cell cycle checkpoint regulators MAD1/Mad1p and MAD2/Mad2p (e.g., [31,32,33]), a budding yeast protein called Pml39p with a proposed role in preventing nuclear export of intron-containing pre-mRNAs [34], the Sumo protease Ulp1p in budding yeast and its metazoan homolog SENP1 (e.g., [35,36]), the components of the mRNA export complex TREX-2 (e.g., [37,38]), and the ubiquitin E3 ligase COP1/RFWD2 [39].

In the current study, we present the zinc finger protein ZC3HC1 (zinc finger C3HC-type protein 1; [40]) as a genuine NB protein of 53–55 kDa in vertebrates. Formerly, ZC3HC1 had also been called HSCP216, as its cDNA had been among those isolated from hematopoietic stem/progenitor cells [41]. In addition, it was also called ILP1 (inhibitor of apoptosis protein [IAP]-like protein 1), due to some parts of its sequence being similar to that of IAP proteins [42], and NIPA (nuclear interacting partner of ALK), after having isolated it in a yeast two-hybrid screen (Y2H) with a chimeric bait that included the receptor tyrosine kinase ALK [43]. Furthermore, NIPA had been described as a nuclear F-box protein and as primarily existing as a regular part of the SCF-type (SKP1, CUL1, F-box) of multiprotein E3 ubiquitin ligase complexes in the interphase of proliferating cells, while reported to be occurring only in minimal amounts in growth-arrested cells [44,45,46,47,48]. Moreover, NIPA had been described among these studies as targeting cyclin B1 (CCNB1) in interphase, to promote its degradation, and thereby prevent premature mitotic entry due to otherwise increased levels of nuclear CCNB1 earlier in interphase.

Here, we show that ZC3HC1/NIPA, which we regard as lacking an F-box and which we neither find to be part of an SCF complex nor required for maintaining the typical subcellular distribution of CCNB1, is an NB-resident protein, with virtually all ZC3HC1 polypeptides located there in certain types of proliferating cells in interphase. Furthermore, we describe ZC3HC1 in vertebrates as occurring at the NEs of all TPR-containing cell types of different morphogenetic origin investigated, including oocytes and non-dividing, terminally differentiated somatic cell types. Moreover, while we show that localisation of ZC3HC1 at the NE depends on TPR polypeptides already present at the NPC, we further demonstrate that also ZC3HC1 itself is needed for enabling additional amounts of TPR to occur bound to the NBs of different human cell types. Finally, we reveal that the ZC3HC1-dependent TPR populations in the somatic cells represent about half the total amount of NE-appended TPR, pointing at the NB’s structure as more complex than formerly anticipated.

## 2. Materials and Methods

### 2.1. Antibodies

General information and technical specifications regarding antibodies used in this study are provided in Supplemental Material and Methods (M&M) and Table S2.

### 2.2. Animal Tissues and Primary Cells

*Xenopus laevis* females were purchased from NASCO (Fort Atkinson, WI, USA); for further details, see Supplemental M&M. All *Xenopus* handling and surgical interventions were in compliance with German law, subject to authorisation by the Veterinary Institute of LAVES, a state office of Lower Saxony, and regularly examined and validated by the animal welfare officer and veterinarians of the Max Planck Institute for Biophysical Chemistry, under supervision of the veterinary authority of the administrative district of Göttingen. In the current study, frogs referred to as weight classes 1 (slim), 2 (average weight), and 3 (heavyweight) weighed between 105–120 g, between 140–150 g, and above 170 g, respectively. Oocytes from anaesthetised animals were obtained by ovariectomy via a small incision of 5 mm in length. Oocytes were transferred into a modified version of Barth’s saline (MBS; 88 mM NaCl, 1 mM KCl, 2.4 mM NaHCO_3_, 0.3 mM Ca(NO_3_)_2_, 0.41 mM CaCl_2_, 0.82 mM MgSO_4_, 15 mM HEPES, pH 7.6) before being processed further (see below). For obtaining *X. laevis* eggs, the animals were injected with human chorion-gonadotropin (Sigma-Aldrich, St. Louis, MO, USA) and then allowed to lay eggs in a modified version of Marc’s Modified Ringer’s solution (MMR; 100 mM NaCl, 2 mM KCl, 1 mM MgCl_2_, 2 mM CaCl_2_, 0.1 mM EDTA, 5 mM HEPES, pH 7.8). *X. laevis* erythrocytes to be studied by immunofluorescence microscopy (IFM) mostly stemmed from occasional blood droplets obtained during surgery, collected for preparing smears of such erythrocytes on SuperFrost Ultra Plus microscope slides (Gerhard Menzel B.V. & Co.KG, Braunschweig, Germany), which were then air-dried and stored at −20 °C for later use (see further below). To isolate larger numbers of erythrocytes, performed in addition to tissue and organ harvesting from the same frog, we modified a published protocol [49] to allow for NB-stabilising (NB-s) conditions before subsequent cell fractionation. In brief, blood from an adult *X. laevis* frog was collected in ERY-buffer (41.5 mM KCl, 8.5 mM NaCl, 5 mM MgCl_2_, 2.5 mM EGTA, 15 mM trisodium citrate, 10% sucrose, 20 mM HEPES, pH 7.5) supplemented with cOmplete EDTA-free protease inhibitor cocktail (Roche, Basel, Switzerland), and filled up to a volume of 50 mL. After centrifugation at 500× *g* and 18 °C for 5 min, the pellet of red blood cells was resuspended and washed with 25 mL of the same buffer and centrifuged again. This was repeated once, and the final pellet was resuspended in the desired volume, followed by cell number determination with an improved Neubauer hemocytometer and fractionation. Organs from *Macaca mulatta* were kindly provided by the German Primate Center (DPZ) in Göttingen.

### 2.3. Scanning Electron Microscopy and Immuno-SEM

Manually isolated oocyte nuclei of different stages (ranging from II to VI) of oogenesis, obtained from frogs of weight class 1, were transferred onto silicon chips (5 mm^2^) that were submerged in 5:1-H buffer (83 mM KCl, 17 mM NaCl, 10 mM HEPES, pH 7.0–7.1) without or supplemented with MgCl_2_ at concentrations ranging from 0.5 to 5 mM, depending on the experimental objective. As a standard procedure for scanning electron microscopy (SEM), the attached nuclei were then opened with tweezers, followed by spreading the NEs onto the chip and removing the nuclear contents by rinsing in buffers based on 5:1-H, which already allowed for removing much of the NE-appended materials of various kinds, while maintaining the integrity of the NPCs and NPC-attached NBs (for *Xenopus* oocyte NB-destabilizing (NB-d) conditions, see Supplemental M&M). Other protocols for treating NEs in buffers of the same or other composition, with and without nucleases and detergents, will be outlined elsewhere in detail in another study’s context. NEs treated according to the standard procedure were then fixed in MgCl_2_-containing 5:1-H containing 2% formaldehyde (FA) and 0.025% glutaraldehyde (GA) at 18 °C for 20 min, followed by post-fixation with 2% GA and 0.2% tannic acid (TA) in MgCl_2_-free 5:1-H at 4 °C overnight. After rinsing in H_2_O, the NEs were post-fixed with 0.5% OsO_4_ in H_2_O for 20 min, followed by further rinsing and subsequent staining with 0.1% uranyl acetate (UA) for 10 min, serial dehydration in ethanol, and critical point-drying in a BAL-TEC CPD 030 (Balzers, Liechtenstein) via CO_2_. Usually without having performed any further metal-coating, mounted specimens were examined via SE detection in a Hitachi S-5500 field emission in-lens scanning electron microscope (SEM) (Hitachi High-Technologies Europe GmbH, Krefeld, Germany), most commonly at 10–15 kV. Sputter-coating of specimens with chromium, presented as Figure S3, was conducted with a Leica/BAL-TEC EM MED 020 equipped with a 99.95% chromium sputter target disc (BAL-TEC) and a QSG100 quartz crystal monitor system for film thickness (Leica Microsystems). For immuno-SEM (iSEM), with all the protocol steps performed at 18 °C, the NEs spread onto the silicon chips were transferred into 5:1-H buffer containing 0.5 mM MgCl_2_ and 2% FA and fixed for 10 min. This was followed by quenching for 5 min in 5:1-H containing 50 mM NH_4_Cl, and subsequent blocking in 5:1-H with 1% gelatine from cold water fish skin (Sigma-Aldrich) for at least 30 min. Incubation with primary antibodies, in the current study from rabbits, was for 60 min. This was followed by several washes and subsequent incubation with anti-rabbit F(ab’)_2_ fragments coupled to 10 nm colloidal gold (AURION ImmunoGold Reagents & Accessories, Wageningen, Netherlands) for 60 min, with all these latter steps still performed in 5:1-H containing 1% fish gelatine. The immunogold-labelled specimens were only then rinsed with 5:1-H without gelatine, followed by post-fixation with 2% FA in 5:1-H for 20 min, and then with 0.5% GA and 0.1% TA in 5:1-H overnight. Subsequent processing was as for non-labelled specimens. The final examination in the SEM was by concurrent secondary electron (SE) and backscattered electron (BSE) detection, with signals then superimposed by using the Quartz PCI 7 software (version 7.0, Quartz Imaging Corporation, Vancouver, BC, Canada).

### 2.4. High-Pressure Freezing, Freeze-Substitution, and Post-Embedding Immuno-TEM

For high-pressure freezing (HPF), *Xenopus* oocytes of late-stage II were loaded into aluminium planchets with cavity depths of 150 µm, with HPF then conducted with a Leica EM HPM100 high-pressure freezer (Leica Microsystems). Freeze-substitution (FS) was performed in a Leica EM AFS2 (Leica Microsystems), in which specimens were first placed into 100% acetone and finally embedded in the hydrophilic Lowicryl resin K4M (Polysciences Europe GmbH, Hirschberg, Germany). For details regarding the FS procedure, see Supplemental M&M. Sections of approximately 60–80 nm thickness were cut with a Leica EM UC6 ultramicrotome (Leica Microsystems) and transferred either onto glass coverslips for IFM (see Supplemental M&M) or copper grids for immuno-transmission electron microscopy (iTEM). For immunogold-labelling, specimens were first blocked in PBS containing 25% (*v*/*v*) of DAKO antibody diluent solution S3022 (DAKO, Glostrup, Denmark) for 30 min. Then, dilutions of antibodies and subsequent incubations with them, and all washing steps, were performed in the same blocking buffer. Incubation with primary rabbit antibodies was for 90 min, followed by several washes, a 60 min incubation with 10 nm gold-coupled anti-rabbit F(ab’)_2_ fragments (AURION ImmunoGold Reagents), and further washes. Specimens were examined with an H-7600 transmission electron microscope (TEM) (Hitachi High-Technologies) at 80 kV, equipped with an Olympus MegaView 3 CCD camera. The analySIS Image Processing software iTEM (version 5.1.0.2086, Olympus Soft Imaging Solutions, Münster, Germany) was used for distance measurements.

### 2.5. Cell Culture

All cell lines used in this study, including those generated by CRISPR/Cas9n-editing, and their growth conditions, including types of media used, are listed in Table S4 and Supplemental M&M. Growth rates were determined by seeding the cells on multi-well dishes at defined cell densities and then counting cell numbers, after having singularized the cells by trypsinization, with an improved Neubauer hemocytometer every 24 h for 4 days.

### 2.6. Synchronisation, Transfection, and Post-transcriptional Gene Silencing of Cultured Cells

Cell cycle synchronisation of human cells of lines HeLa, HCT116, and U-2 OS were performed as described [50,51], with some modifications as outlined in the Supplemental M&M, while hTCEpi cells were grown to confluency, which in this cell line results in contact inhibition and cell cycle arrest, from which the cells were released again by sub-culturing. Transfections of human cells with small interfering RNAs (siRNAs) (for list of siRNAs used in this study, see Table S3) were performed with HiPerFect (QIAGEN, Hilden, Germany), according to the manufacturer’s instructions. For most RNA interference (RNAi) experiments, individual 21 nt siRNA duplexes were applied at 6 pM, i.e., at about 40 ng of double-stranded siRNA per cm^2^ of growth area. For RNAi data presented in the central part of this study, cells were harvested at three days post-transfection (for other cell lines and experimental details, see Supplemental M&M).

### 2.7. Immunofluorescence Microscopy

Cryostat sectioning of oocytes and animal tissues, fixation, and immunolabelling of the defrosted sections for IFM was similar to former procedures (e.g., [25,52]) but with several modifications outlined in the Supplemental M&M. Defrosted blood smears of *X. laevis* were processed similarly as cultured vertebrate cells seeded on coverslips. In brief, the specimens were usually fixed for 30 min with 2.4% of freshly prepared and methanol-free FA in 1×PBS, followed by quenching with 50 mM NH_4_Cl in PBS, and then permeabilisation usually with 0.25% Triton X-100 (TX-100) in PBS. In some experiments, 0.005% digitonin, which allows for preserving intactness of the NE, was used as the detergent instead. Blocking and antibody incubations were performed with 1% BSA in PBS, with sufficient washes in PBS between these steps. DAPI or Hoechst 33342 (1 µg/mL) for DNA staining was usually added during incubation with the secondary antibodies. Specimens were finally mounted in SlowFade Gold Antifade Mountant (Invitrogen, Carlsbad, CA, USA). In cases in which detergent-permeabilisation was carried out before FA-fixation, cells were treated with 0.25% TX-100, sometimes in PBS with MgCl_2_ freshly added to a final concentration of at least 7 mM, but mostly in buffers optimised for NB stabilisation, depending on the experimental objective, followed by fixation and quenching, yet omitting subsequent treatments with detergents. Specimens were inspected with the Leica TCS SP5 or SP8 confocal laser-scanning microscope (Leica Microsystems, Wetzlar, Germany). ImageJ/FiJi software (versions 1.50i–1.51t, National Institutes of Health, USA; [53]) was used to generate line profiles and quantify signals at the NEs on micrographs obtained by IFM.

### 2.8. Cell Fractionation and Egg Extracts

Manual isolation of stage II, III, V, or VI nuclei from *X. laevis* oocytes in 5:1-H buffer (see above) at 18 °C, the preparation of the annulate lamellae (AL)-containing fraction from oocytes that were first manually defolliculated and then enucleated before the AL isolation procedure, and the isolation of cytosol from such enucleated oocytes, were in essence as described [52], with some modifications as specified in the Supplemental M&M. Manual isolation of oocyte NEs under conditions maintaining NB integrity, and nuclear contents of oocytes to be used for standard protein-biochemical analyses were also performed in 5:1-H buffer at 18 °C, in principle following a procedure described in detail earlier [54] (for further details and *Xenopus* oocyte NB-d conditions, see Supplemental M&M). Fractionation of cultured populations of XL-177 and human cell lines was in principle as described [14,55], yet also with different sets of buffered solutions, including such formerly used for enrichment and isolation of nuclei and NPCs (e.g., [56]), and others that allow for maintaining the interaction of ZC3HC1 with the NBs and the lamina-NPC-NB (LNN)-enriched fractions of different cell types. In brief, the LNN-enriched fraction of XL-177 cells presented in Figure 3C was obtained by resuspending a pellet of these cells, sedimented by 800× *g* centrifugation for 3 min, in an RT-warm NB-s solution containing 41.5 mM KCl, 8.5 mM NaCl, 5 mM MgCl2, 2.5 mM EGTA, 2 mM DTT, 10% sucrose, 10 mM HEPES, pH 7.2, 0.2% TX-100, and cOmplete EDTA-free protease inhibitor cocktail (Roche), followed by incubation for 4 min and centrifugation for 3 min at 20,000× *g* and RT. The soluble and LNN-enriched pellet fractions of HeLa cells presented in Figure 3D were obtained by resuspending pellets of these cells, sedimented by 900× *g* for 3 min, in (i) an RT-warm NB-s solution containing 20.75 mM KCl, 4.25 mM NaCl, 5 mM MgCl2, 2.5 mM EGTA, 2 mM DTT, 10% sucrose, 20 mM HEPES, pH 7.25, 0.25% TX-100, and cOmplete EDTA-free protease inhibitor cocktail, and in (ii) an ice-cold, exemplary NB-d solution, formerly used for the isolation of morphologically intact mammalian NPCs [56], containing 0.1 mM MgCl_2,_ 1 mM DTT, 10% sucrose, 20 mM triethanolamine, pH 8.25, 0.25% TX-100, and the cOmplete EDTA-free protease inhibitor cocktail. HeLa cells in NB-s buffer were incubated at RT for 8 min, and those in NB-d buffer on ice for 2 min, each followed by centrifugation at 20,000× *g* for 3 min. LNN fractions of different human cell lines presented in Figure 7 were prepared like for the corresponding materials in Figure 3D, except for having incubated the cells in NB-s buffer at RT for 4 min only. Preparation of egg extracts capable of in vitro assembly of nuclei was, for the most part, as described [57], with some modifications specified in the Supplemental M&M.

### 2.9. Immunoprecipitation, Phosphatase Treatment, and Immunoblotting

For IPs of native proteins, antibodies had first been covalently bound to Protein A-coupled magnetic Dynabeads (Invitrogen). For details regarding this procedure, see Supplemental M&M. Incubations of beads with *Xenopus* egg extracts diluted in four volumes immunoprecipitation (IP) buffer (41.5 mM KCl, 8.5 mM NaCl, 5 mM MgCl_2_, 2.5 mM EGTA, 10% sucrose, 20 mM HEPES pH 7.25), supplemented with cOmplete Mini EDTA-free protease inhibitor cocktail (Roche), was performed for 30 min at RT. This was followed by the magnetic collection of beads, subsequent brief washes in IP buffer containing 0.02% Tween-20, and final elution of bound proteins with a pH 2.5 solution of 100 mM glycine. For the dephosphorylation of ZC3HC1 in egg extracts, the concentrated extract obtained after 250,000× *g* centrifugation (see Supplemental M&M) was first diluted about six-fold by adding various solutions, including the concentrated buffer for protein metallophosphatases (NEB, Ipswich, MA, USA) diluted to 1x, the cOmplete Mini EDTA-free protease inhibitor cocktail, and a stock solution of MnCl_2_, the latter added at a final concentration of 1 mM. Per microliter of the undiluted egg extract, equivalent to the cytosolic volume of about two eggs, 200 units of Lambda protein phosphatase (NEB) were added, and for phosphatase inhibition in control experiments, the phosphatase inhibitors vanadate and sodium fluoride were additionally added at the final concentrations of 10 mM and 50 mM, respectively. For data presented in the current study, incubations were carried out at 18 °C for 15 min. SDS–PAGE [58] and subsequent immunoblotting (IB), using nitrocellulose membranes, were done as described earlier (e.g., [11,25]), yet with some modifications outlined in the Supplemental M&M.

### 2.10. ZC3HC1 Gene Disruption in Human Cell Lines

Following the Cas9 double-nickase approach [59], indels in the first exon of the *ZC3HC1* alleles within the genome of human cells were achieved by CRISPR/Cas9n technology, thereby targeting this *ZC3HC1* exon by two pairs of sgRNAs, using each pair independently from the other. Sequence design for the chimeric single guide RNAs (sgRNA) was done using the CRISPR Design tool formerly provided online by the Zhang laboratory [60,61]. The required total of four pairs of complementary, to be annealed oligonucleotides (Sigma-Aldrich), corresponding to the two pairs of sgRNAs (pair 1: sgRNA1, GAGGGGATTGCCCCGGAAGA and sgRNA2, gCTATCAGCTGCCGGATT TTC; pair 2: sgRNA3, GCAGTAGTTCGCTCCCCAGA and sgRNA4, GCAAACGCTTGT CCCTCACA; only sgRNA targeting sequences shown), possessed additional, not presented, single-strand sequence overhangs. These were complementary to those of the BbsI-digested bicistronic Cas9n expression vector pSpCas9n(BB)-2A-Puro (PX462) V2.0 [62] kindly provided by Feng Zhang (Addgene plasmid # 62987; [63]; see also Table S5) into which the four annealed oligonucleotides were cloned. The 5′-end-positioned guanine nucleotide for sgRNA2, here written in lowercase (see above) because of not being complementary to the ZC3HC1 sequence, was included to enhance U6 promoter-driven expression of guide RNAs. Cultured cells were then separately transfected with each one of the two pairs of resulting Cas9n expression vectors, thereby applying the two plasmids corresponding to one pair of sgRNAs in equal amounts. On the following day, the cell culture medium was replaced with fresh medium supplemented with puromycin at a final concentration of 5 µg/mL for 12 h to select for transfected cells. Populations of surviving cells were then subjected to serial dilutions to obtain monoclonal cell lines that were further screened via IFM. Monoclonal populations of the ZC3HC1 KO cell strains were further analysed by IB and by DNA sequencing of subcloned PCR products. Sequences of oligonucleotides, used as forward and reverse primers for PCR and subcloning of PCR products, were complementary to genomic sequences flanking exon 1 of the human ZC3HC1 gene and read CCCTGCTGAATTTGGACTGACAGCG and CTGCGGTTGGGAATCTGGTACACC.

Extended Materials and Methods, also including further information relating to experiments presented only as Supplemental Figures, are provided as part of the Supplementary Materials.

## 3. Results

### 3.1. ZC3HC1 Is an NB-Resident Protein

**Figure 1:**
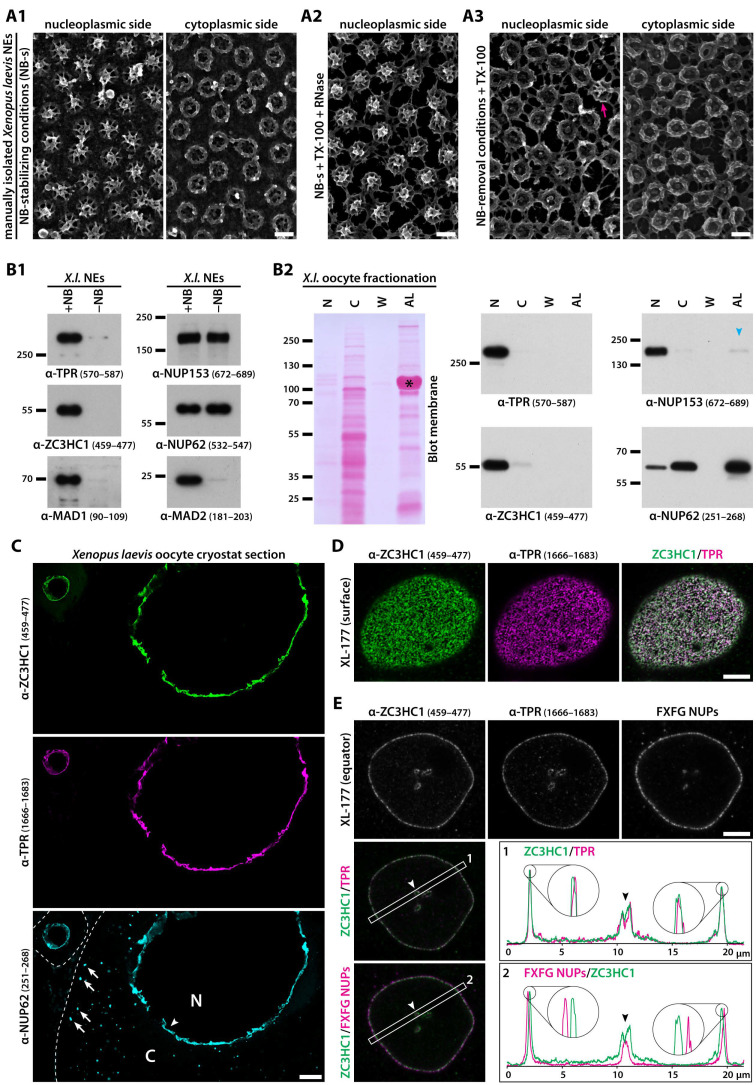
ZC3HC1 is a NE-associated protein in *Xenopus laevis* oocytes and cultured cells. (**A**) SEM micrographs of manually isolated and then differently treated *X. laevis* NEs from a weight class 1 frog’s late-stage V oocytes (for weight class definition, see Material and Methods). (**A1**) Nuclear and cytoplasmic face of NEs that had been isolated under NB-stabilising conditions (NB-s) preserving NPC and NB integrity while largely removing other NE-associated materials. (**A2**) Nuclear face of NEs treated with TX-100 and RNases, after having been isolated like the NEs presented in (A1), maintaining NB integrity. (**A3**) Nuclear and cytoplasmic face of the same NE, which had been treated with TX-100 but not with nucleases, following an NB-removal procedure that nonetheless allowed for maintaining the integrity of the lamina, the actual NPCs and the appendices at the NPCs’ cytoplasmic side. One of those sporadically observed NBs that had been disassembled only partially (arrowhead) is shown as a point of reference for the NE’s nuclear side. Bars, 100 nm; same magnification for all SE micrographs. (**B**) IB of subcellular fractions of *X. laevis* oocytes. (**B1**) IB of NEs that had been manually isolated from late-stage V oocytes, then incubated as two separate batches in parallel, either under NB integrity (+NB) or NB removal (-NB) conditions, subsequently extracted with TX-100, and finally sedimented by centrifugation. Labelling with indicated antibodies (target regions in parentheses) was on the upper and lower parts of the same membrane and on an identical duplicate, respectively. Since the total amount of loaded NE proteins did not exceed 500 ng per lane, resulting in polypeptide patterns not visible after staining by dyes like Ponceau S (PS), the stained membranes are not shown here. Note that ZC3HC1 was no longer present among the NE proteins after having applied conditions that removed most of the NB scaffold protein TPR and other NB proteins, like MAD1 and MAD2. By contrast, components of the NPC, like NUP62, had remained largely unaffected, and the NE-associated amount of NUP153 had only been slightly reduced in the absence of the NBs. (**B2**) IB of the soluble cytosolic proteins and particulate cytoplasmic materials (C) that after low-speed centrifugation had remained in the supernatant, the AL-containing membrane fraction, which still contained some contaminating yolk protein (asterisk marks major representative), the second wash of this latter fraction (W), and the total of proteins from the intact nuclei (N). All fractions stemmed from a batch of a weight class 3 frog’s stage VI oocytes that had been manually enucleated first. The oocytes’ other fractions, including other organelles and membranes, pigment granules, and the bulk of yolk, all of which were devoid of ZC3HC1 and TPR, are not shown here. Labelling for xlZC3HC1 and reference proteins xlTPR and xlNUP153, the latter recurrently detected also within the *Xenopus* oocyte’s AL fraction in trace amounts (blue arrowhead), and xlNUP62, as a regular part of the ALPCs, was on the upper and lower parts of the PS-stained membrane shown here, and on an identical duplicate. Each lane was loaded with the respective fraction or washing solution volume corresponding to only one oocyte, explaining why materials in lane N were hardly visible after PS-staining. Note that ZC3HC1, in stark contrast to NUP62, was not detectable in the AL fraction and instead turned out to be a primarily nuclear protein, just like TPR. (**C**) Triple-labelling IFM of cryostat sections of *Xenopus* oocytes with guinea pig, rabbit, and mouse antibodies for xlZC3HC1, xlTPR, and xlNUP62, respectively. Note that while NUP62 was seen both at the NE (arrowhead) and the cytoplasmic AL (some marked by arrows), ZC3HC1 and TPR were only detectable at the NE. The nucleus (N) and cytoplasmic compartment (C) of the stage VI oocyte are marked, as is the nucleus (asterisk) of an early-stage oocyte in which colocalisation at the NE occurred too. Oocytes were from a weight class 2 frog, with commonly less AL material than in class 3 frog oocytes. White dashed lines demark the cell boundaries of the early- and late-stage oocyte. Bar, 50 µm. (**D**) Double-labelling IFM of XL-177 cells for xlZC3HC1 and xlTPR, with the focal plane at the polar region of a representative nucleus. Note that both proteins at this resolution appeared to largely colocalise in dots, which for TPR were already known to represent NPC-associated NBs. Bar, 5 µm. (**E**) IFM of XL-177 cells for xlZC3HC1 and xlTPR and with a monoclonal antibody (mAb), mAb414, which binds to several NPC-located FG-repeat nucleoporins (NUPs). The focal plane was at the equator of a representative nucleus. Rectangles in the overlay micrographs were analysed by the ImageJ software, with line profiles plotted. Note the 4× enlarged line profile sections, showing almost complete overlap of IF-labelling for NE-associated ZC3HC1 and TPR, and an offset location of ZC3HC1 towards the nuclear interior relative to the FG-repeat NUPs, with approximate distances of around 150 nm between the line profile peaks for the labellings with mAb414 and for ZC3HC1. Note also the inverse order of labelling for ZC3HC1 and the FG-repeat NUPs at an NE invagination (arrowhead), compared to the NE’s not folded parts. Bar, 5 µm.

Following the lampbrush chromosome stage of oogenesis, the oocyte nuclei of the clawed frog *Xenopus laevis* are characterised, amongst other features, by enormous dimensions and the virtual absence of NE-associated chromatin (e.g., [64]). These specifics allow for manual isolation of the oocytes’ nuclei and the NEs from them, which can yield envelopes with only sparse remnants of contaminating cytoplasmic and nuclear material, while the NPCs and their NBs appear morphologically intact (Figure 1(A1)). The cleansed NEs then also lack most of the additional fibrillar materials of differing appearances that have been described as appendices at the NE’s nuclear side in amphibian oocytes (e.g., [65,66,67,68,69,70]). Such NEs can then be further treated with RNases and non-ionic detergents, which in buffers maintaining NB integrity allows for much of the membrane lipids to be extracted while still leaving the NPCs and their NBs intact (Figure 1(A2)). The NB, however, whose appendage to the NPC depends on a combination of different types of non-covalent bonds, can also be specifically destabilised, which allows for detaching the NB’s components from an intact NE, while at the same time neither affecting the morphological integrity of the NPC proper nor the appendage of proteins at the NPC’s cytoplasmic side. Hence largely devoid of NBs, the NE can then be treated once again with nucleases, non-ionic detergents, or both (Figure 1(A3), and our unpublished data).

In the course of characterising the protein composition of such *X. laevis* oocyte NEs that we had manually isolated in large numbers and then processed in a variety of ways, we had identified ZC3HC1 as one of those proteins that remained robustly associated with them. This interaction withstood additional treatments with nucleases and non-ionic detergents, as long as these did not affect NB integrity. In contrast, ZC3HC1 had been absent from these NEs under conditions that allowed for selective disassembly and detachment of the NPC-appended NB (corresponding mass spectrometry data to be presented elsewhere). Next, we then raised antibodies against various oocyte NE proteins, among which were also several against ZC3HC1, which made it possible to further analyse the protein’s subcellular distribution in the oocyte by IB (Figure 1B, and further below) and IFM (Figure 1C; for further characterisation of antibodies, see Figure S1).

First, this allowed us to confirm that ZC3HC1 is part of the oocyte’s complement of NE proteins as long as the NBs are present, while absent under conditions that cause NBs to be detached from the NE (Figure 1(B1)). These findings resembled similar ones for NB scaffold protein TPR and proteins whose NE association was known to largely depend on TPR, like, e.g., MAD1 and MAD2 (e.g., [32,36]).

By contrast, NPC components like the FG-repeat nucleoporin NUP62 (e.g., [71,72,73,74]), a protein of the central translocation channel, and NUP153, another FG-repeat nucleoporin [75] and a TPR-binding partner appended to the NR independently of TPR (e.g., [50,75]), were not affected by the removal of the NBs from the detergent-treated NEs (Figure 1(B1)), which was in line with such NB removal not notably affecting the NPC’s overall morphology.

Next, further IB experiments revealed that ZC3HC1 is, in fact, primarily a nuclear protein within the oocyte, with only trace amounts detectable within the cytosol, where they perhaps represent newly synthesised proteins in transit to the nucleus (Figure 1(B2)). Especially noteworthy, ZC3HC1 was found virtually absent from the oocyte’s annulate lamellae (AL). These AL, conspicuous arrangements of folded membranes of ER origin (e.g., [76,77]), which contain densely packed cytoplasmic pore complexes (in the following called ALPCs), are particularly abundant in amphibian oocytes, including those of *X. laevis* (e.g., [52,78]). 

Such oocyte ALPCs are composed of proteins of the NPC proper, including the *Xenopus* homolog of the transmembrane protein NUP210 and the channel protein NUP62 [52,79], the latter as the one example shown here too, and they also comprise those proteins tethered to the NPC’s cytoplasmic side, such as NUP358 [80]. However, they contain only trace amounts of NUP153 (Figure 1(B2)), and they lack TPR [25] as well as at least some of the other proteins located at the nuclear side of the NE (e.g., [52,81]). In the oocyte of a weight class 3 frog, the total number of ALPCs can exceed the number of NE-embedded NPCs by up to an order of magnitude, and this comes along with the largest amounts of most of the NPC proteins being AL- rather than NPC-associated in such oocytes ([52,80], and our unpublished data). While this is conceivable when comparing the nuclear and AL-associated amounts of NUP62, such conspicuous amassments of AL were found to be entirely devoid of ZC3HC1, just as is the case for TPR (Figure 1(B2)).

However, neither absence from the AL nor detachment together with known NB proteins from the oocytes’ NEs already sufficed for unequivocally classifying ZC3HC1 as an NB-associated protein. Furthermore, we could not claim ZC3HC1 to be an exclusively NE-associated protein in the oocyte (Information SI 1), as it also existed in varying amounts in soluble form within these nuclei. Moreover, we had not yet been able to exclude at this point the possibility that the occurrence of ZC3HC1 at the oocyte’s NE, where it appeared enriched (Figure 1C), might merely be a specific feature of only this particular cell type, known to stockpile at least some proteins at different storage sites within the cell for later use during embryogenesis (e.g., [64,82]).

To promptly address this possibility, we inspected other types of *Xenopus* cells by IFM, including the cultured *Xenopus* cells of the tadpole-derived line XL-177 [83,84]. Such analyses revealed that ZC3HC1 is an NE-associated protein in this somatic cell type, too (Figure 1D,E), where it also locates at the NPC’s nuclear side (Figure S2A). Moreover, even by such conventional confocal laser scanning microscopy, it already became evident that ZC3HC1 locates notably further away from the proteins of the NPC proper, and that it instead appeared to colocalise with the NB scaffold protein TPR (Figure 1E). The offset location of ZC3HC1 towards the nuclear interior was especially notable upon double-labelling IFM with mAb414. This mAb labels several FG-repeat NUPs in IB, including xlNUP62 and xlNUP153 ([71]; Figure S1(G2)), but when used for standard IFM of cells in interphase, it preferentially labels the NPC’s cytoplasmically exposed FG-repeat NUPs, including a NUP62 subpopulation (our unpublished data).

Subfractionation of XL-177, thereby preserving NB integrity, revealed that in this cell type, too, certain amounts of ZC3HC1 exist in a soluble form in interphase. Such additional ZC3HC1 was not only detectable in proliferating sub-confluent cell populations but also within such that had reached densely packed confluency, which in this adherent cell type comes along with a markedly decreased growth rate. However, the largest amount of ZC3HC1 within this cell type was nonetheless clearly detectable within its LNN-enriched fractions (Figure S2B). Eventually, this turned out to be the case for yet other cell types as well, in some of which ZC3HC1 was even located exclusively at the NE and only detectable within the LNN-enriched materials (see further below).

Confident at some point that ZC3HC1 localisation at the oocyte’s NE was not merely a speciality of this cell type but was turning out to be common to all cell types investigated in the current study, we aimed at visualising the objects that were immunolabelled with the ZC3HC1 antibodies. To this end, we first performed iSEM on *X. laevis* NEs from stage V oocytes, obtained from a weight class 1 frog (for details regarding the choice of frogs and oocytes, see Information SI 2).

As an essential prerequisite, we had established a modified procedure of sample preparation for iSEM that rendered conventional heavy metal-coating superfluous (for further details, also regarding iSEM data interpretation, see Information SI 3). In the following, this allowed us to assign the vast majority of the high-energy BSE signals to the actual immunogold-particles (IGPs) from which these BSEs originated because these IGPs were then also discernible via imaging with the low-energy SEs.

To first immunolabel the NPC-proximal and -distal parts of the NB as reference points, we used antibodies specific for amino-terminal (NT) domain segments of xlNUP153 (aa 40–327) and xlTPR (aa 9–25). NUP153 is an NPC protein whose N-terminal part, including its NPC binding-domain (NPBD) and its additional nuclear membrane-binding domain (NMBD; e.g., [85,86,87,88]), is known to be positioned at or just next to the NPC’s NR in *X. laevis* and humans (e.g., [14,89]). TPR’s N-terminus, on the other hand, had already been found located within the NB’s TR region ([14]; Figure S3B,C).

**Figure 2:**
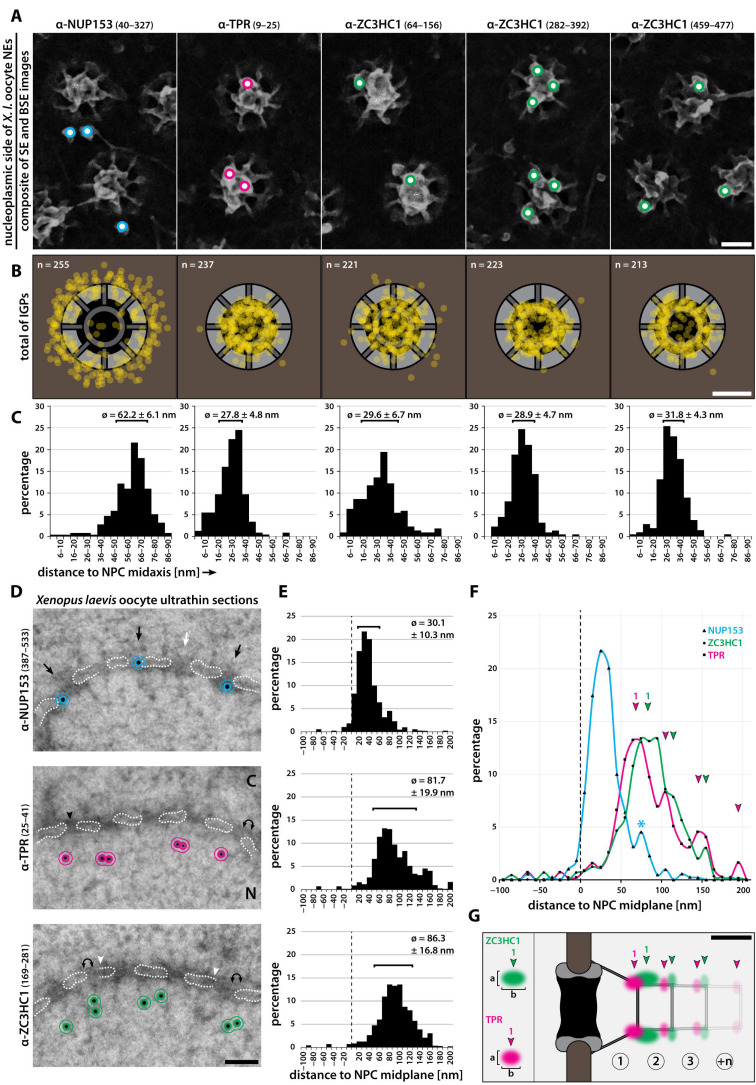
ZC3HC1 is located at the NB primarily in the TR region. (**A**) Immuno-SEM of manually isolated *X. laevis* stage V oocyte NEs with antibodies for different parts of xlZC3HC1, the NT of xlTPR, and the NPBD of xlNUP153 and part of its NMBD. Representative composite images consist of SE micrographs of the NE’s nuclear face and the superimposed BSE images reflecting the IGPs’ positions, encircled in blue for NUP153, magenta for TPR, and green for ZC3HC1 (for the corresponding separate SE and BSE images, see Figure S3(D2)). Note that while the NUP153 antibodies decorated sites close to the NR, antibodies for ZC3HC1 primarily labelled the TR region, similar to IGP distribution for TPR’s NT. Bar, 50 nm. (**B**) Distributions of IGPs relative to an idealistic NPC and its NB in a face-on view. Schemes are drawn to scale, with the outer and inner diameters of the NR corresponding to 110 nm and 70 nm, and the outer diameter of the TR to 55 nm (for details regarding data acquisition and presentation, see Figure S3E,F). Bar, 50 nm. (**C**) Bar diagrams corresponding to the datasets presented in (B), representing the percentages of IGPs, grouped within windows of 5 nm width, that were located at differing distances away from the NB’s longitudinal axis, here represented by the y-axis. Values of the mean radial distances, provided together with standard deviation (SD) values, were deduced from the middle 68% of all measured distances values (contingents demarked by brackets) for those IGPs presented in (B) (for further information, see Figure S3(F1–6)). (**D**) Post-embedding iTEM on ultrathin sections of high-pressure-frozen, freeze-substituted, and then resin-embedded late-stage II oocytes of *X. laevis*. Sections had been labelled with antibodies for the central part of xlZC3HC1, the NT of xlTPR, and an N-terminal region of xlNUP153 that also comprises part of its TBD. Cytoplasm (C) and nuclear compartment (N), separated by the NE, are oriented toward the top and bottom. Parts of the NE segments presented, including their NPC wall-forming portions, are partially highlighted by white dashed lines to facilitate recognition. IGPs are shown encircled, with a diameter of 40 nm for the outer circles (for the rationale, see Figure S5C). Black arrows demark examples of NPCs in cross-section that had been IGP-decorated while the white arrow points at an NPC that had escaped labelling. White arrowheads point at parts of the NE where the actual phospholipid bilayer boundaries were not discernible, the black arrowhead at the example of an NPC whose non-diametric perpendicular section only yielded a small circle segment, and the double-headed arrows demark parts of the NE that appeared skewed and distorted for different reasons, with these and other features exclusion criteria (outlined in Figure S6A) for IGP distance measurements. Bar, 100 nm. (**E**) Bar diagrams representing the distribution of IGPs relative to the NE midplane (dashed vertical). Each histogram represents the mean of three different series of altogether several hundred measurements (see also Figure S6), comprising the percentages of those IGPs within windows of 10 nm width that were detected up to 100 nm and 200 nm away from the midplane of sectioned NPCs at their cytoplasmic (negative values) and nuclear sides (positive values). Provided values of mean distances and corresponding SD values are based on the middle 68% of all measured distance values for each target (contingents demarked by brackets). Note that ZC3HC1 and TPR-NT IGPs were found enriched in a similar region at the nuclear side of the NPC, which also harbours the NB’s TR, while the majority of NUP153-TBD IGPs were in closer proximity of the NE. (**F**) Incorporation of the three datasets, shown in (E), into one diagram as line graphs to facilitate comparison of relative IGP peak positions. Note that the major peak (numbered as 1) for ZC3HC1 was located slightly further away from the NPC midplane (dashed vertical) than the major one for the NT of TPR. Furthermore, in addition to each of these proteins’ major peaks, minor amounts of corresponding IGPs also appeared to be locally enriched, seemingly in intervals, further away from the NE, with the peaks for TPR-NT and ZC3HC1 marked by arrowheads in magenta and green, and the first additional minor peak for NUP153 by an asterisk. (**G**) Model for ZC3HC1 as a TR-resident and TPR-NT-neighbouring component of the NPC-attached prototypic NB, depicted in lateral view, and as a potential component of additional, sporadically observed TR-appended NB-like fibrillar structures (NBLS) described in Figure S7. Except for the fibril widths, the dimensions of the model segment meant to represent the NPC-attached NB, here numbered as ①, correspond approximately to those of the FA-fixed NB on an isolated *Xenopus* oocyte NE, commonly regarded as prototypic. In addition, this highly simplified model approximately outlines the contours of the NBLS, here numbered ②, ③ and +n, that can occur stacked on top of the TR of an NPC-attached NB, with these NBLS here depicted with increasing degree of transparency the further away positioned from the NB, to reflect the assumed decreasing frequency by which such NBLS occur stacked one on top of the other within the late-stage II oocyte. The length of such an NBLS is here depicted corresponding to about 42.5 nm. The green- and magenta-coloured clouds are meant to show the approximate distribution of those ZC3HC1 and TPR-NT IGPs that represent the major and minor peaks also arrowhead-marked in the respective colour in (F), with their positions relative to the NE midplane in (G) aligned to those in (F). Regarding the minor peaks’ coloured clouds, the increasing degree of transparency is approximately proportional to the corresponding peak decrease in (F). The width (here depicted as [a]) of the first clouds and their positioning relative to the NB’s central longitudinal axis is meant to reflect the relative positions of those IGPs detected in iSEM and shown in (B) that represent the central 68% of all signals around the mean distribution for TPR-NT and the mean for collectively all of the three ZC3HC1 datasets. The length (here depicted as [b]) of the first clouds and their positioning relative to the NPC’s midplane reflects the chord lengths of collinear sections, in parallel to the abscissa, through the main peaks for TPR-NT and ZC3HC1 in (F), which here corresponds to distances from the NPC midplane of 57–79 nm for TPR-NT and 69–98 nm for ZC3HC1. Bar, 50 nm.

Following immunogold-labelling with these antibodies, more than 97% of those BSE signals that had emanated from solitary, morphologically intact NBs (see also Table S1) could be unambiguously superimposed onto the actual IGPs now visible in the SE modus (see also Figure S3(D1)). Regarding the N-terminal part of xlNUP153, the visible IGPs were mainly (95.7%) located at the base of the NBs, close to the NPC’s NR. By contrast, in the case of the N-terminus of xlTPR, the visible IGPs were found almost exclusively at the NB scaffold’s distal end (95.9%), i.e., in the TR region (Figure 2A, Table S1, and further below), with both findings in line with former immuno-EM localisations (e.g., [14,89]).

In parallel to such iSEM of xlNUP153 and xlTPR, immunolabelling was performed with antibodies specifically targeting three non-overlapping segments of xlZC3HC1, comprising aa 64–156, aa 282–392, and aa 459–477, and all of these resulted in a near-exclusive decoration of the NB. While only a minor proportion of the BSE signals that had been assignable to an NPC or solitary NB yet not to any visible IGP in the SE modus (0.4–2.6%) appeared to stem from sites located beneath the TR and closer to the NR, the majority of the NB-associated IGPs (>97%) were again clearly visible also in the SE modus. And most of these IGPs (87.7–94.2%) were found to decorate the NB’s TR region (Figure 2A, Table S1, and further below). In some of the few cases in which a ZC3HC1 IGP did not appear located there, it seemed as if the IGP, while connected to the TR’s outer perimeter, had collapsed down onto the side of the NB, with corresponding BSE signals then seemingly originating from sites closer to the NR.

 Nonetheless, despite such uncertainties regarding the localisation of a small amount of IGPs, and with only a minor number of gold particles assignable to the NR region, iSEM with these ZC3HC1 antibodies indicated that in the *Xenopus* oocyte, the NB-associated population of this protein is primarily located at the NB’s distal end. There, some ZC3HC1 IGPs even appeared to decorate additional fibrillar material, sometimes notable on top of some of the NBs, onto which such fibrils occasionally also appeared to have collapsed in the course of the process of sample preparation (our unpublished data). However, while we also made similar observations when inspecting NBs decorated with IGPs for TPR’s NT, we regard work aiming at unequivocally discerning the details regarding structure and composition of such TR-appended additional fibrils as beyond the scope of the current study.

At this point, one also had to call to mind that the appearance of the immunolabelled NB as visualised by SEM, following manual isolation of the NEs and subsequent fixation with chemical cross-linkers like FA and GA, might differ to some extent from its natural morphology in the living cell. In addition, it was not unexpected that conventional iSEM using gold-coupled IgGs would not allow for determining which of the NB’s structural elements in the TR region precisely had been labelled with the TPR-NT and ZC3HC1 antibodies. The reason being that an IGP’s position in iSEM does not necessarily reflect the exact position of the target site, with some offset possible between epitope and the mean of IGP positions (Information SI 3).

Despite such considerations regarding the degree of resolution achievable by iSEM, we deemed the IGP’s mean positions relative to the labelled NB’s medial axis, i.e., its longitudinal axis, informative. Therefore, we plotted the BSE signals’ positions, relative to the NB and NPC, onto the scheme of an idealistic NB structure, which included a highly simplified scheme of the TR in top-view. In doing so, we saw to it that the plots reflected the distance between each BSE signal’s position and the closest neighbouring NB fibril, as seen in the composite BSE-SE image, as accurately as possible. The BSE signals to be plotted included all of those emanating from NPCs with a solitary, morphologically intact NB and from neighbouring sites, irrespective of whether these BSE signals were assignable to a visible IGP or not. Next, we measured the distance between these signal positions and the NB’s medial axis (Figure S3E).

The resulting BSE signal plots and measurement data were further categorised (Figure S3F), with the largest of several groups of BSE sub-datasets being the one in which a BSE-corresponding IGP was visible in the SE modus and located within the area of a disk of 180 nm in diameter, with the morphologically intact-appearing NB in its centre (Figure 2B,C).

Altogether, these IGPs represented at least 97% of the BSE signals for each of the here used antibodies at solitary and intact NBs (for the additional sub-datasets, presented for further comparison, again see Figure S3F). With these datasets eventually confirmed as sufficiently robust and diagnostically conclusive within the bounds of feasibility, it did not come as a surprise that some of these measurements illustrated apparent differences in the radial distributions of the different IGP populations, with the gold particles for the N-terminal part of NUP153 most distally positioned. By contrast, IGPs decorating the NT of xlTPR were much closer to the NB’s medial axis, as their region of occurrence was corresponding to the area harbouring the elements forming the TR. Furthermore, those IGPs that had labelled the different parts of xlZC3HC1 were also primarily positioned in the TR region, yet with some differences regarding the radial distances of the IGP-decorated ZC3HC1 segments from the NB’s central axis (Figure 2B,C).

To illustrate and determine the actual distance between the NE and the NB-associated xlZC3HC1 polypeptides, we subsequently performed post-embedding iTEM on ultrathin sections of resin-embedded late-stage II *Xenopus* oocytes. These had been high-pressure frozen and then freeze-substituted with solutions and resins in the absence of cross-linkers like FA and GA. Oocytes of this stage of oogenesis were chosen because we already knew that TPR and ZC3HC1 were located at the NE also in the early stages of oogenesis (e.g., Figure 1C) and because the size of such a stage II oocyte was still small enough to allow for uniform vitrification of the subcellular regions surrounding the NE (for further details regarding the rationale regarding the choice of oocytes for HPF, for late-stage II NB morphology relative to that of late-stage V oocytes, for specimen appearance after HPF and FS, and for assessment of antibody performance on corresponding ultrathin sections by IFM, see Information SI 4 and SI 5, and Figures S3G, S4, and S5). 

Furthermore, post-embedding iTEM methodology was chosen because antibodies binding to NPC epitopes only exposed to the surface of sections can experience far more rotational freedom than when the same epitopes are targeted in pre-embedding iTEM. In the latter case, structure integrity can restrict epitope accessibility, thereby delimiting the range of angles by which antibodies can bind to these epitopes, ultimately resulting in the IGPs’ mean position being distinctly offset from the actual target site. By contrast, while an individual IGP in post-embedding iTEM can be similarly far apart from its target as in pre-embedding iTEM, the mean position of a sufficient number of such IGPs can eventually define the actual location of many target proteins at the NPC and NB more accurately (e.g., [14,74]).

In addition to ZC3HC1 immunogold-labelling, antibodies against NUP153 and TPR that had turned out suitable for post-embedding iTEM were used for reference labellings again. Among these were antibodies against the NT of xlTPR and a domain segment of xlNUP153 that included part of its TPR-binding domain (TBD), known to be located at the NR (e.g., [14,89]). Such xlNUP153 IGPs then were detected predominantly close to the nuclear side of the NPC (Figure 2D), thereby exhibiting a rather narrow Gaussian distribution, with the majority of gold grains (68%) located between 12 to 54 nm away from the NE’s midplane, with a therefrom deducible mean of 30.1 nm (Figure 2E,F; for details regarding data acquisition and evaluation, see Figures S5 and S6).

By contrast, IGPs for the NT of xlTPR were found located deeper within the nuclear interior (Figure 2D), with a more widely spread distribution and the majority of grains (68%) located 53 to 128 nm away from the NPC’s midplane, resulting in a deducible mean of 81.6 nm (Figure 2E,F). While technical features related to sample processing and sectioning for post-embedding iTEM, in addition to potential biological causes (see Information SI 6), might have contributed to such spread distribution, we also considered the possibility that such mean value might reflect the mean of two adjoining Gaussian peaks, with their flanks overlapping in such a manner that the individual peaks could not be clearly resolved by iTEM. We did not regard such a scenario improbable since additional, albeit minor peaks, with seemingly regular spacing between each other, were notable even further away from the NE midplane (magenta arrowheads in Figure 2F), possibly representing additional sites of locally enhanced TPR-NT immunogold distribution deeper within the nucleus (further addressed in Figure S7).

In the case of xlZC3HC1, the IGPs were found distributed very similarly to those for xlTPR-NT, with again most immunogold (68%) located 55 to 119 nm away from the NPC’s midplane, at a therefrom deducible mean distance of 86.3 nm, yet again not excluding the possibility that this might reflect the mean of an overlap between two peaks of IGP distribution. Furthermore, minor additional peaks were noted again deeper within the nuclear interior, possibly reflecting additional sites of ZC3HC1 residence close to the additional ones for TPR’s NT (Figure 2D–F, and Figures S5(C6), S6 and S7). It is known that some of the NPC-appended NBs in the *Xenopus* oocyte can possess one or more additional NB-like structures (NBLS), each about 40–45 nm in length, deducible also from their initial presentation [90]. Such NBLS are stacked on top of the TR, thereby forming repetitive arrangements of cylindrical shape projecting further into the nuclear interior, as seen here (Figure S7) and in the past (e.g., [66,67,90]). We thus consider it possible that such additional NBLS not only contain TPR, which already earlier had been noted to be part of NPC-appended fibrillar material beyond the dimensions of the NB proper (e.g., [25]) but ZC3HC1 as well.

While the abovementioned mean values hence might overvalue to some extent the distance of each protein’s first main peak from the NPC’s midplane, we nonetheless are confident that the similar distribution of the IGPs for ZC3HC1 and TPR’s NT, in an area distant from the NPC proper, is diagnostically conclusive. In fact, even though the NB itself is generally not discernible on such ultrathin perpendicular sections, most of the NPC-to-IGP distances determined for ZC3HC1 and TPR’s NT corresponded rather well to the area that harbours the NB scaffold’s distal end (e.g., [11]). 

In summary, we, therefore, deemed it justified to conclude that our iSEM and iTEM data jointly depicted the NE-associated ZC3HC1 as a protein primarily located at the distal end, i.e., in the TR region, of the NPC-attached NB in *Xenopus* oocytes commonly regarded as prototypic (① in Figure 2G). At the same time, we are not excluding the possibility that additional populations of NB-associated ZC3HC1 and TPR polypeptides can also occur beyond the dimensions of the NB proper, possibly as components of additional NB-like entities (②, ③, and +n, in Figure 2G; Figure S7).

### 3.2. Residency of ZC3HC1 at the NE and Colocalisation with NB Scaffold Protein TPR Is Common in Proliferating Cells and Post-Mitotic Tissue Cells of Different Germ Layer Origin

While TPR is ubiquitously present at the NEs of proliferating and post-mitotic cells of different morphogenetic origin [25,55], we were initially uncertain whether ZC3HC1 might also occur at the NEs of terminally differentiated cells that have exited the cell cycle and whether this protein might be in line for qualifying as a generally NB-resident protein. This uncertainty was also due to ZC3HC1 having been described until then as a protein primarily occurring in proliferating cells, with only minimal amounts reported to exist in growth-arrested cells [44].

**Figure 3:**
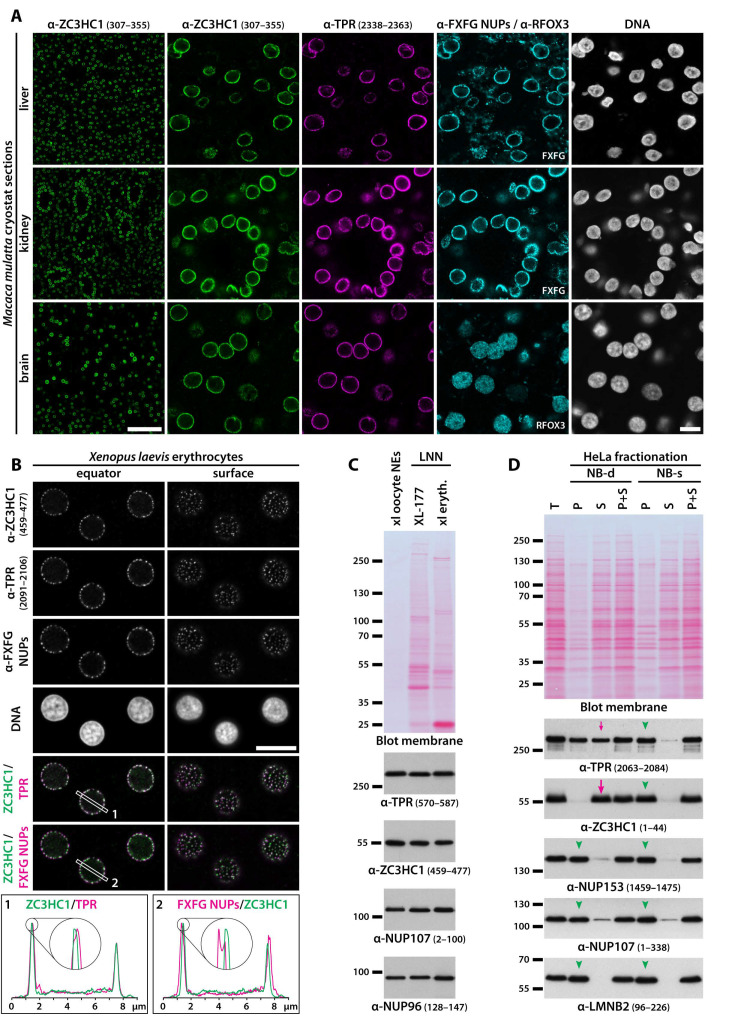
Widespread occurrence and anchorage of ZC3HC1 at the NEs of proliferating and non-dividing, terminally differentiated cells of different morphogenetic origin. (**A**) IFM of cryostat sections of the liver, the kidney’s renal pyramids and columns, and the forebrain’s prefrontal cortex from *Macaca mulatta*, representing tissues originating from the three germ layers. Overviews (left side) only show labelling for hsZC3HC1, while the micrographs at higher magnification also present the identical specimens’ triple-labelling with further antibodies, including such for TPR. For further comparison, liver and kidney sections were labelled with mAb414, while the cerebrum section was labelled for the neuronal cell marker RFOX3/NeuN to distinguish neuronal cells from glial cells. Note that ZC3HC1 and TPR were found colocalising at the NEs of cells present in these tissues. Bars, 100 µm (overview) and 10 µm, respectively. (**B**) IFM of non-dividing, mature *X. laevis* erythrocytes. The nuclei are shown in two focal planes, with the one on the nuclei’s equator and the other near the nuclei’s surface. Note the 4× enlarged line profile sections, showing ZC3HC1 at this level of resolution colocalising with TPR’s C-terminal domain, the latter known to be positioned in the TR region too. Bar, 10 µm. (**C**) IB of the LNN-enriched fraction of XL-177 cells and *Xenopus* erythrocytes obtained after extraction with TX-100, and of manually isolated and cleansed *Xenopus* oocyte NEs, obtained from a weight class 1 frog’s stage V oocytes and possessing intact NBs and NPCs, but hardly any of the different types of fibrillar appendices found appended in varying amounts to an oocyte NE from a weight class 3 frog. Loading amounts had been adjusted in such a manner that similar IB signal intensities were obtained for ZC3HC1. Labelling with indicated antibodies was performed on the upper and lower parts of the membrane stained with MemCode but here shown colour-converted from blue to red. Note that while the NE-associated amounts of TPR and ZC3HC1 relative to the reference NPC scaffold proteins NUP107 and NUP96 appeared higher in oocytes and XL-177 cells than in erythrocytes, the amount ratios between TPR and ZC3HC1 within the NB-enriched materials of these three different cell types appeared to be rather similar. (**D**) IB of cell extracts obtained from a confluent, not synchronised population of HeLa cells following fractionations of similar cell numbers performed in parallel, having applied either NB-s conditions, maintaining NB integrity, or NB-d conditions, causing partial or complete detachment of the one or other NB component from otherwise intact NPCs. Lanes were loaded with the non-fractionated cells’ total cell proteins (T), the soluble proteins released during extraction with TX-100 in NB-s or NB-d buffer (S), and the corresponding non-soluble pellet fractions (P). In addition, the same amounts of S and P materials from each fractionation were also loaded together in one lane to compare the resulting amount with that of the non-fractionated cells, demonstrating that hardly any amount of protein had been lost during the S and P fractions’ preparation. Labelling for hsZC3HC1, hsTPR, hsNUP153, and hsNUP107, and with mAb X223 cross-reactive with human LMNB2, the latter as an additional reference, was performed on the upper and lower parts of the MemCode-stained membrane shown colour-converted to red, and on an identical duplicate. Note that NUP153, anchored to the NPC’s NR, had remained bound to the LNN-enriched pelletable material, irrespective of whether NB-d or NB-s conditions had been applied, as it also was the case for NUP107 and LMNB2 (green arrowheads). Similarly, when having applied NB-s conditions, essentially all ZC3HC1 and TPR had remained bound to the LNN-enriched material too (arrowheads), even after the prolonged incubation at RT and despite efficient cell extraction and solubilisation of most of the cells’ other proteins. By striking contrast, essentially all ZC3HC1 had turned soluble (magenta-coloured large arrow) already after a short incubation at low temperature and in the absence of adequate amounts of divalent cations (for further details, see Materials and Methods). Further note that this had been accompanied by the loss of about half the cell’s total amount of TPR (magenta-coloured small arrow). As an aside, NUP107 polypeptides, also known to be part of ER-embedded individual pore complexes and small AL sheets, both of which are present in HeLa cells too (e.g., [99]), had contributed to the minor amounts of NUP107 in the soluble fractions following TX-100-extraction.

To address this issue, we examined whether and where ZC3HC1 could be detected within diverse types of terminally differentiated cells. First, we performed IFM on cryostat sections of a range of different *Xenopus* tissues, including such of endodermal, ectodermal, and mesodermal germ layer origin, and found that NEs of cells positive for TPR were generally also positive for ZC3HC1 (Figure S1H). Furthermore, to study ZC3HC1 and its occurrence in mammals too, we raised antibodies against the human homolog, which allowed for visualising its nearly ubiquitous occurrence also at the NEs of all mammalian cell types later investigated in this study, including cells from mice (our unpublished data) and rhesus macaques (Figure 3A). 

Strikingly, while other subcellular components were not visibly labelled, ZC3HC1 was again found colocalising with TPR at the NEs of cells within tissues of all three germ layer origins. Among these were even the NEs of neuronal cells present in the frontal lobe of the cerebrum of adult animals, i.e., the NEs of cells of which most had been in a post-mitotic state for several years (Figure 3A).

The nucleated *Xenopus* erythrocyte represents another extreme example of a terminally differentiated cell type no longer capable of cell division. It is characterised by highly condensed chromatin, hardly any transcriptional activity (e.g., [91,92,93]), and low NPC density, with the latter likely similar to that in the red blood cells of other tetraploid anurans, for which less than 5 NPCs per µm^2^ of the nuclear surface had been determined [94]. Already known to possess NPC-associated TPR [25], we found ZC3HC1 also in this cell type to be colocalising with TPR next to the NPCs, i.e., adjacent to, but nonetheless spatially clearly apart from other components of the NPC (Figure 3B). Furthermore, also upon shutdown of gene transcription in tumour cells, ZC3HC1 remained located at the NE, together with TPR (Figure S8).

To further scrutinise the validity of such IFM-based findings of ZC3HC1 located at the NE in different tissues, and, in particular, to assess whether its amounts, relative to those of TPR and attributable to the NB, might differ between proliferating and non-proliferating cell types, we investigated this by immunoblotting the proteins of NEs and LNN-enriched fractions that we had obtained from different types of cells. We were already aware by then that some of the more common cell fractionation procedures can cause ZC3HC1 and certain amounts of TPR to be detached from the NE, as will be exemplified further below. Therefore, we took care that their association with the actual NBs was maintained when isolating such LNN-enriched materials and that only the genuine nucleoplasmic populations of soluble ZC3HC1 and TPR were removed from those cells in which such additional pools exist.

Furthermore, to compare the amounts of ZC3HC1 also relative to those of NPC proteins, the NEs and LNN-enriched materials were immunoblotted in parallel for NUP107 and NUP96. Like many components of the NPC scaffold, these are long-lived proteins with little turnover once they are part of an NPC, and their copy numbers per NPC are regarded as rather constant in different cell types, as long as these stem from the same organism (e.g., [95,96,97,98]).

To compare these relationships preferably within cell types notably differing from each other in many aspects, we immunoblotted, next to each other, the proteins of the manually isolated and cleansed NEs of *Xenopus* oocytes and those of the LNN-enriched materials of TX-100-extracted XL-177 cells and *Xenopus* erythrocytes. Protein amounts loaded for this purpose were adjusted for yielding similar immunoblot signal intensities for ZC3HC1 to facilitate the assessment of ratios between ZC3HC1 and the other proteins (Figure 3C). The results of such IB experiments revealed that the ratio of those ZC3HC1 to TPR polypeptides that had remained bound to the LNN-enriched materials, where they hence can be regarded components of the NB, appeared to be quite similar in these cell types, further pointing at some possibly distinct relationship between ZC3HC1 and TPR (Figure 3C).

In addition, these findings also indicated that copy number relationships between ZC3HC1 and NUP107 or NUP96 in different cell types could be similar too, at least when comparing cleansed stage V oocyte NEs with LNN-enriched materials obtained from XL-177 cells (Figure 3C). However, we also noted that the NE-associated amounts of TPR and ZC3HC1 relative to those of the NPC proteins could be moderately higher in oocytes than in erythrocytes (Figure 3C). We regard it worth mentioning that after having also performed such IB experiments with the cleansed oocyte NEs and the erythrocyte-derived LNN-enriched materials obtained from other frogs, differing in age, weight, and feeding status, it was evident that such ratio of NB to NPC proteins could vary to some extent. Nonetheless, we found it to be generally higher in the oocyte NE materials than in the corresponding animal’s LNN fractions of its erythrocytes, and sometimes even in comparison to the LNN materials of the XL-177 cells. 

However, while the quantity ratios of NE-associated NB proteins to NPC scaffold proteins might thus differ to some extent in the different types of *Xenopus* cells, the quantity ratio of ZC3HC1 to TPR within such NE materials generally appeared to be more constant. This, in turn, tempted us to consider it possible that ZC3HC1, as an NB-associated protein, might commonly occur in amounts rather proportional to those of the NB-forming TPR polypeptides (for further considerations on this topic, see Information SI 7), and later in our study, we would examine this possibility in human cell types (see below).

Furthermore, these cell fractionation experiments had also demonstrated that the interactions between ZC3HC1 and other components of the LNN-enriched materials from very different types of cells could be quite enduring, even in the course of quite different isolation procedures, as long as specific physicochemical parameters were ensured. On the other hand, we found them also rapidly destabilised when the cells were exposed to certain non-physiological conditions, here referred to as the NB-d conditions (see also Information SI 8). 

For example, the interaction between the NB and ZC3HC1 could be easily dissolved by the lack of adequate concentrations of divalent cations like Mg^2+^ within the one or other fractionation buffer and by the common mode of performing at least some of the fractionation steps at low temperatures near the freezing point. If not counterbalanced by other means, such conditions in combination exhibited additive effects, allowing for rapid and complete detachment of all ZC3HC1 polypeptides from the NBs of at least some cell types within less than a minute after having been exposed to them. Here, we exemplified this by single-step fractionation of HeLa cells, which only took a few minutes and was conducted at low temperature and in the presence of only trace amounts of Mg^2+^, which resulted in the detachment of essentially all ZC3HC1 from the LNN fraction (Figure 3D). Furthermore remarkable, the complete loss of ZC3HC1 under such conditions was accompanied by the detachment of seemingly about half the total amount of NE-associated TPR, while NPC scaffold proteins like NUP107 and even other proteins directly attached to the NPC’s nuclear side, like NUP153, remained largely unaffected (Figure 3D).

By contrast, many of those fractionation conditions that had allowed for essentially all ZC3HC1 to remain bound to the LNN fractions, then also together with all TPR, had included higher concentrations of divalent cations and performing the fractionations at room temperature. Hence, several of those conditions that allowed for keeping all ZC3HC1 and TPR bound to the NE, referred to as the NB-s conditions, more closely imitated the physiologically relevant conditions within the cells. This is exemplified by an NB-s fractionation of HeLa cells, conducted in parallel to the NB-d fractionation procedure that resulted in complete detachment of ZC3HC1 (Figure 3D).

In summary, these results already suggested some kind of interdependency between ZC3HC1 and TPR, possibly even indicative of different levels of interactions existing between these proteins. Moreover, our data by then were consistently pointing at ZC3HC1 being an NB protein primarily residing together with TPR at the NPCs of a wide range of cell types, irrespective of whether these are in an actively cycling state or have exited the cell cycle.

### 3.3. ZC3HC1 and TPR Engage in Physical Interactions and Depend on Each Other for Undiminished NE-Association of Both Proteins

While its localisation at the NB in *Xenopus* oocytes had already indicated that ZC3HC1 is a likely interaction partner of protein TPR, it initially seemed challenging to demonstrate by stringently controlled IP experiments unequivocally a genuine capability of interaction between the cells’ native proteins. Some of the cell types inspected in the current study lacked naturally occurring pools of soluble TPR and ZC3HC1 in interphase, and others harboured either only one or the other of the two proteins in soluble form in varying amounts (see also Information SI 9 and Figure S16(C1)). 

Therefore, we aimed at extracts in which the amounts of endogenous ZC3HC1 polypeptides, and in particular those of TPR, that are in a naturally still soluble state but already at the verge of being interaction-competent, are much higher than in somatic cell extracts or in the immature *Xenopus* oocyte arrested in G2. This happens to be the case in the cytosol of the mature *X. laevis* egg when it has become NE-assembly-competent by having released the egg from its metaphase arrest [57]. This procedure allows for at least certain amounts of the various soluble components required for the assembly of NPCs and NBs to occur in a dephosphorylated state again, which in turn renders them ready to form such structures again (e.g., [89,100]). 

**Figure 4:**
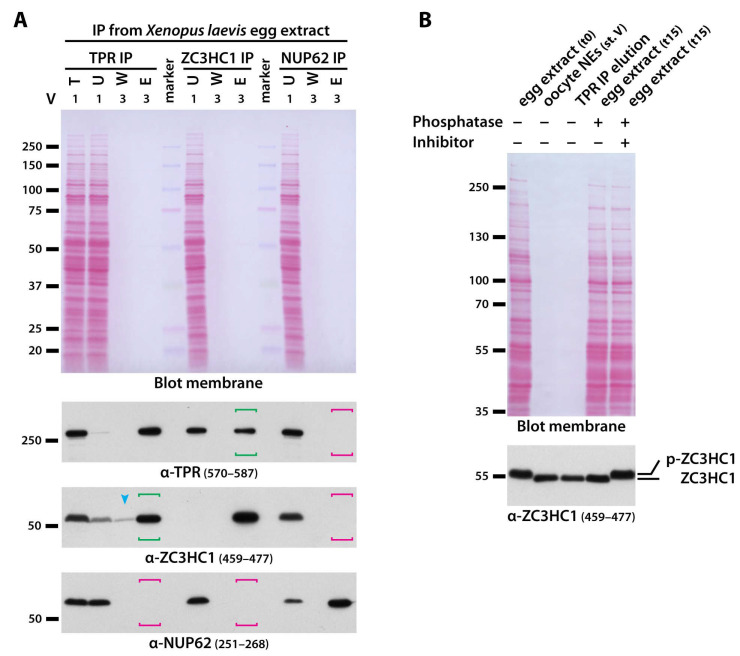
Upon removal of phosphate modifications acquired during egg arrest at metaphase, soluble ZC3HC1 can re-engage in specific physical interactions with TPR even in solution. (**A**) IP of TPR and ZC3HC1 from 250,000× *g* supernatants of *X. laevis* egg extracts competent for post-mitotic nuclear assembly. IP of NUP62 from the same supernatant was performed as a control in parallel. Lanes were loaded with the total soluble cell proteins (T), with those proteins that had remained unbound (U) after bead incubation, those released during the third of three successive washing steps (W), and those obtained after final elution (E). Loadings in T and U represented the same volume fraction of the respective samples’ total amount (1 V), while the loadings in lanes W and E represented three-fold higher relative amounts (3 V). Immunolabelling was on the upper and lower parts of the PS-stained membrane shown here and on an identical duplicate. Cases in which a protein had been co-immunoprecipitated with the IP’s actual target protein are framed with brackets in green; brackets in magenta accentuate those in which no co-IP had occurred. Note that a subpopulation of TPR polypeptides had been co-immunoprecipitated together with ZC3HC1 and that, *vice versa*, a large proportion of the extract’s total content of ZC3HC1 had been co-immunoprecipitated with TPR. By contrast, neither TPR nor ZC3HC1 had been isolated together with NUP62, or *vice versa*. As an aside, note that some ZC3HC1 was found gradually detaching from the immunoprecipitated TPR during washes (blue arrowhead). Such polypeptides appear to represent one of seemingly two ZC3HC1 populations attracted by TPR, of which one is less tightly associated with TPR in vitro than the other. (**B**) IB of ZC3HC1 as the protein co-immunoprecipitated with TPR, as a component of manually isolated NEs from *Xenopus* stage V oocytes, and as a protein within the extract from metaphase-arrested *Xenopus* eggs, with such extract having been left untreated (t0), and after incubation for 15 min (t15) with λ phosphatase alone, and with the same amount of λ phosphatase supplemented with phosphatase inhibitor. The corresponding PS-stained membrane lacked the proteins below 30 kDa due to intentionally prolonged SDS-PAGE for better separation of ZC3HC1 polypeptides. Note that ZC3HC1 dephosphorylated with λ phosphatase exhibited electrophoretic mobility similar to that of the NB-associated ZC3HC1 polypeptides and those co-immunoprecipitated together with TPR from assembly-competent egg extracts.

When using these quasi-post-mitotic egg extracts for the IP of TPR, it was possible to specifically co-immunoprecipitate a distinct subpopulation of ZC3HC1 polypeptides (Figure 4A). Apparently, a major proportion of both proteins had indeed been dephosphorylated again to such an extent (Figure 4B) that this then allowed for renewed physical interaction. 

Furthermore, the responsible endogenous phosphatases were sometimes found to be active for some time even within the finally prepared extracts, gradually increasing the amounts of dephosphorylated ZC3HC1 in the test tube, which appeared to correlate with a then steadily improving co-IP with TPR. On the other hand, in some other batches of egg extracts that had been treated to be assembly-competent, more ZC3HC1 polypeptides were found still phosphorylated, and these then were still incapable of binding to TPR (our unpublished data).

Clearly though, using those batches of truly post-mitotic extracts with which it was possible to co-immunoprecipitate the dephosphorylated ZC3HC1 polypeptides together with immunoprecipitated TPR (Figure 4A), it was, *vice versa*, also possible to specifically co-isolate a notable amount of TPR when immunoprecipitating ZC3HC1 as the actual target for IP (Figure 4A; for explanations regarding non-quantitative co-IP of TPR upon quantitative IP of ZC3HC1, and *vice versa*, see Information SI 9). By contrast, several control IPs, including, for example, the nearly complete IP of all NUP62 from such assembly-competent extracts, did not even result in traces of TPR or ZC3HC1 being co-isolated (Figure 4A, and our unpublished data). Furthermore, other formerly reported binding partners of ZC3HC1 turned out not to be specifically co-isolatable with ZC3HC1 in considerable amounts from any cell extract used in the current study, i.e., neither from the post-mitotic *Xenopus* egg extracts nor from the interphase extracts of other cell types, as will be later shown in Figure S11.

**Figure 5:**
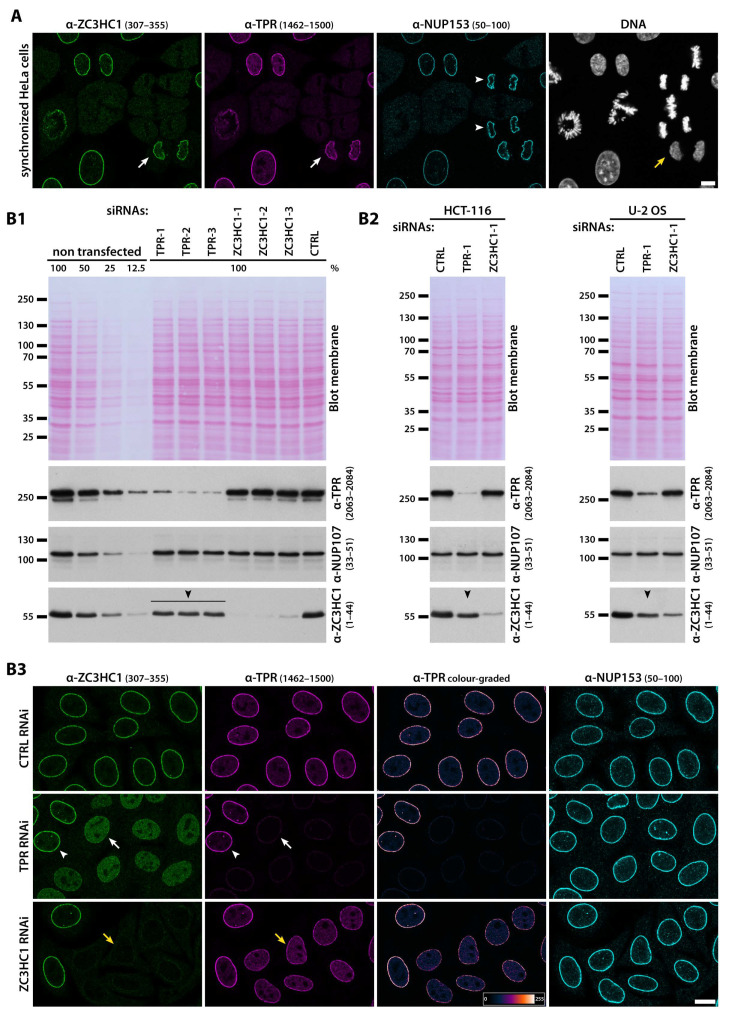
ZC3HC1 is strictly TPR-dependent for its own NE-association but also itself required for proper positioning of a substantial amount of TPR at the NB. (**A**) IFM of HeLa cells with antibodies for hsZC3HC1, hsTPR, and hsNUP153. Populations had been synchronized to increase the number of cells progressing through mitosis, next to some already in the G1 phase and a few still late in G2. Note that concurrent reassociation of ZC3HC1 and TPR with the NE (white arrows) was seen to occur late towards the end of telophase and early in G1, paralleling the onset of chromatin decondensation (yellow arrow), while NUP153 was part of the NE already notably earlier (white arrowheads). Bar, 10 µm. (**B**) RNAi experiments with HeLa and other cell lines, harvested at day 3 post-transfection. (**B1**) IB of whole-protein extracts from HeLa cells that had been transfected with control siRNAs (CTRL) or several different TPR and ZC3HC1 siRNAs (1–3). For estimating overall KD efficiencies, the total extracts from non-transfected cells had been loaded as serial dilutions (100 to 12.5%) in parallel, with the 100% loading equivalent to the amount from the same number of cells from the RNAi experiments. Immunolabelling was on different parts of the PS-stained membrane shown here. Note that TPR KD in this HeLa subline had resulted in a moderate reduction in the cellular amounts of ZC3HC1 (marked by arrowhead). (**B2**) IB of whole-protein extracts from U-2 OS and HCT116 cells transfected with control, TPR or ZC3HC1 siRNAs. Immunolabelling was on different parts of the shown membrane. Note that upon RNAi-mediated KD of TPR, cellular amounts of ZC3HC1 were reduced (arrowheads). (**B3**) IFM of HeLa cells treated with siRNAs. Upon TPR RNAi, only traces of TPR-staining were seen at most of the cells’ NEs; the usually bright NE-staining by TPR antibodies was only visible in cells that had remained non-transfected, here shown as a reference. Note that marked NE staining for ZC3HC1 was only seen in such TPR-positive cells (one marked by arrowheads), while in the TPR-deficient ones (one marked by white arrows), ZC3HC1 was found distributed throughout the nuclear interior. Upon ZC3HC1 RNAi, signal intensities for TPR at the NEs of the ZC3HC1-deficient cells (one marked by yellow arrows), compared to the non-transfected ones, appeared notably reduced, accompanied by the appearance of some additional staining for TPR within the nuclear interior, while NE-association of NUP153 did not appear affected. Further note that the micrographs for TPR are also shown colour-graded to display differences in pixel intensities via a colour look-up table (LUT), revealing a reduction of immunolabelling intensity for TPR at the ZC3HC1-deficient NE by about half. Bar, 10 µm.

Apart from the data so far, further evidence in line with a close connection between ZC3HC1 and TPR came from studying the chronological order by which these proteins are recruited back to the reassembling NPCs in HeLa and XL-177 cells at the end of mitosis (Figure 5A, Figure S9). In contrast to the reincorporation of NPC components like NUP153, which starts in anaphase, reattachment of ZC3HC1 to the post-mitotic NPCs happened much later. In fact, we found it to occur essentially concurrent to that of TPR, already known to become bound to the NPC only late in telophase or early in G1 when nuclear protein import activity has been resumed (Figure 5A; see also, e.g., [50,101,102]).

The relationship between ZC3HC1 and TPR was further underscored by the results of RNAi experiments in human cell lines, found more convenient for such approach than the XL-177 cell line from the allotetraploid organism *X. laevi*s. Even though target protein knockdown (KD) had been possible in XL-177 cells (Figure S1G), this had sometimes only been achievable with extra effort, rendering this cell line less suitable for systematic studies by RNAi (for further details, see Information SI 10).

Conducting RNAi experiments in the human HeLa cell line first, we found that upon RNAi-mediated KD of TPR (Figure 5(B1)), the initially prominent staining for ZC3HC1 at the NE was quantitatively lost, and a nucleoplasmic pool of ZC3HC1 became visible instead (Figure 5(B3)). Such evidence of ZC3HC1 being NPC-associated in a TPR-dependent manner did not come as a surprise, as TPR was known to be essential for NB scaffold formation and integrity [11,14], and ZC3HC1 had been primarily located at the NB’s distal end (Figure 2). Of further note, cellular amounts of ZC3HC1 appeared somewhat reduced in HeLa and other human tumour cell lines, like HCT116, in which TPR had been knocked down by RNAi too (Figure 5(B1,B2)), and in U-2 OS cells, such reduction upon TPR RNAi was particularly striking (Figure 5(B2)). This suggested that residence at the NB might even protect ZC3HC1 from a more rapid turnover, albeit to an extent differing between cell lines.

More remarkable though, we found that also RNAi of ZC3HC1 itself resulted in a conspicuous, yet never complete reduction of the intensity of TPR-immunolabelling at the NE, with the latter then mostly appearing reduced by about half. This, in turn, was accompanied by the appearance of a nucleoplasmic pool of TPR, clearly notable in some cell lines but not in others, several days after having initiated the RNAi-mediated KD of ZC3HC1 (Figure 5(B2), and our unpublished data). Most remarkable, this reduction of NE-associated TPR-immunolabelling upon ZC3HC1 RNAi was not due to a reduced number of NPCs because immunostaining for other NPC proteins, like, for example, NUP358 and components of the Y-complex, remained unchanged (Figure S10A). Accordingly, the comparison of IFM images from the ZC3HC1-positive and ZC3HC1-deficient cells’ pole regions did not reveal any notable reduction in the mean NPC densities per NE surface areas either (Figure S10B).

The observation of a notable reduction in the NE-associated amounts of TPR when ZC3HC1 was absent (Figure 5(B3)) added to our finding that complete solubilisation of ZC3HC1 in the course of certain cell fractionation procedures is accompanied by the release of a large amount of TPR from an LNN-enriched fraction (Figure 3D). Altogether, these results suggested that ZC3HC1 might play a role in allowing for a distinct population of TPR polypeptides to be positioned at the NB, a notion further examined in the later course of our study (see below).

### 3.4. ZC3HC1 Is Not Required for Cellular Housekeeping Activities in Human Tumour and Non-Tumour Cell Lines of Ectodermal, Mesodermal, and Endodermal Origin

While ZC3HC1 presented itself in the current study as a TPR-interacting and NB-resident protein of ubiquitous occurrence, former reports on this protein described it as a nuclear F-box-containing protein with a fundamental role in cell cycle progression and primarily occurring in proliferating cells as a stably bound part of a nuclear SCF-type E3 ubiquitin ligase in interphase (e.g., [44,45,46]); a finding that was recently reported confirmed again [103]. Along this line, ZC3HC1 had been described as detaching from such a nuclear SCF core complex, consisting of subunits SKP1, CUL1 (Cullin 1), and RBX1, only very late in G2, just before mitosis, which supposedly would then result in rapid degradation of ZC3HC1 [44,45,47]. The alleged main function of ZC3HC1, as such an SCF component in interphase, was to bind and promote degradation of those cyclin B1 (CCNB1) polypeptides that leak into the nucleus during interphase, i.e., prior to regular accumulation of CCNB1 within the nucleus at the onset of mitosis. As a direct consequence of such nuclear CCNB1 degradation by SCF-ZC3HC1, premature mitotic entry was reportedly prevented [44,45], and RNAi-mediated ZC3HC1 deficiency had been accordingly reported to cause nuclear accumulation of CCNB1 in interphase. In addition, such a knockdown of ZC3HC1 had been also described as accounting for cell cycle arrest in prometaphase, and as triggering apoptosis, especially in tumour cells like HeLa [44,45,46,104].

However, in the current study, it was not possible to obtain evidence supporting these former statements and findings regarding ZC3HC1. On the contrary, we neither found human ZC3HC1 nor its homologs in different phyla possessing any one of either the F-box or F-box-like consensus sequences provided by the major sequence motif databases. Furthermore, despite extensive efforts, we could neither find any evidence in support of the notion of ZC3HC1 naturally occurring as a genuine part of a cellular ZC3HC1-SCF complex (Figure S11), nor for an interaction between ZC3HC1 and CCNB1, nor for ZC3HC1 being directly involved in regulating the subcellular distribution and amounts of CCNB1 in proliferating cells in interphase (Figure S12, and further below). Also contrary to former reports, we did not note any pronounced triggering of apoptosis as a direct consequence of ZC3HC1 deficiency in HeLa cells, and our findings also did not support the notion that ZC3HC1 may be a protein with a major role in the inhibition of apoptosis in tumour cells in general (Figure S13).

In fact, in the course of our experiments, initially all performed with cells in which ZC3HC1 had been knocked down by RNAi, we had realised that this protein was most likely neither essential for cell cycle progression nor cellular housekeeping activities in interphase (Figure S14). However, even though these early results turned out to be in accordance with the finding that mice in which the ZC3HC1 gene had been knocked out by homologous recombination are viable [104,105], the alleged role for ZC3HC1 in regulating cell cycle progression via controlling cellular levels of CCNB1 was recurrently reported to manifest itself in a range of aneuploid human tumour cell lines and other types of immortalised human cells [104,106,107,108]. HeLa cells were actually suggested to be amongst those cells in which such ZC3HC1 knockdown phenotypes were most pronounced [104], ranging from most cells of a ZC3HC1-deficient HeLa population being no longer capable of cell cycle progression and entering apoptosis instead [104], to hyperproliferation of another ZC3HC1-deficient HeLa population and conspicuous increase in its cell numbers [108], when using the one or other ZC3HC1 siRNA.

Apart from such studies with sometimes seemingly mutually exclusive phenotypes, we had to take into consideration, though, that RNAi might not cause complete elimination of all target transcripts in the HeLa cells and that trace amounts of ZC3HC1 might still be synthesised. Therefore, as long as we saw to it that only non-toxic amounts of siRNAs were administered to the cells, we could not unequivocally exclude that our inconspicuous RNAi data were simply reflecting the outcome of an incomplete loss of ZC3HC1. We, therefore, eventually decided to make use of the by then available CRISPR/Cas9n technology (e.g., [59,109,110]) in order to further address the issue of whether the complete absence of ZC3HC1 would or would not be tolerated by proliferating human cell lines.

**Figure 6:**
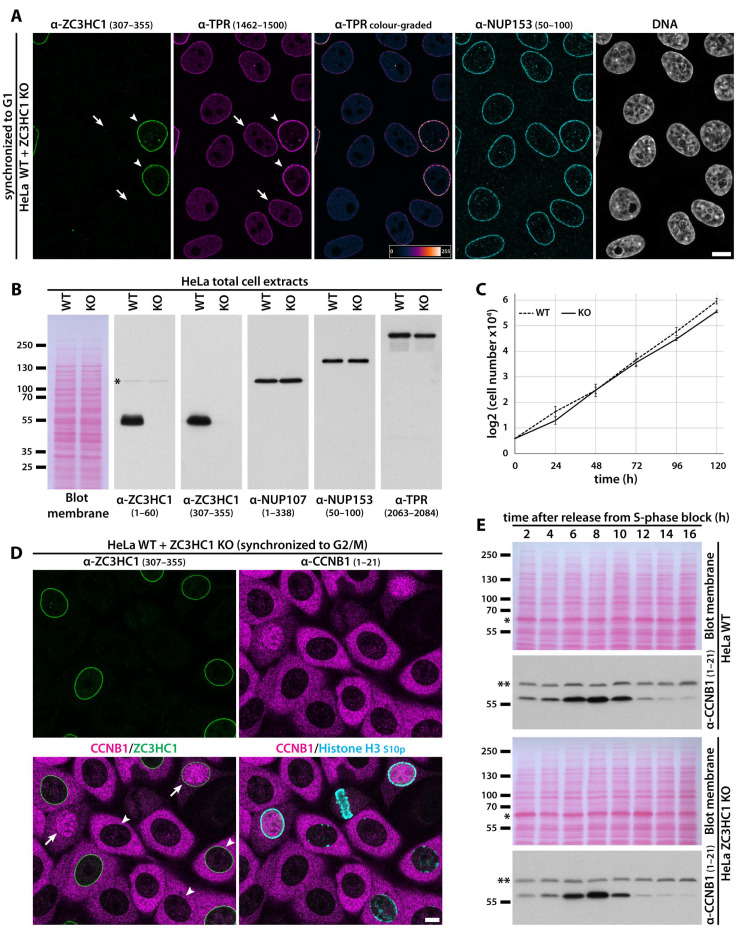
CRISPR/Cas9n-mediated ZC3HC1 gene disruption in HeLa cells neither prohibits cell cycle progression nor alters subcellular CCNB1 distribution. (**A**) IFM of HeLa WT cells grown together with cells of a stable HeLa ZC3HC1 KO line, with these mixed populations of WT and KO cells having been synchronised to the G1-phase. Two WT cells, positive for ZC3HC1, are marked by arrowheads. Note that in the neighbouring KO cells (examples marked by arrows), ZC3HC1 was neither detectable at the NEs nor anywhere else (see also Figure S15(B1)). Signal intensities for TPR at the NEs of the KO cells appeared reduced by about half, accompanied by the appearance of some additional staining for TPR within the nuclear interior, while NE-staining for NUP153 appeared unaffected. Bar, 10 µm. (**B**) IB of total cell extracts from HeLa WT and ZC3HC1 KO cells. Labelling with two different ZC3HC1 antibodies and for TPR, NUP153, and NUP107 for comparison was on the PS-stained membrane and on duplicates with identical loadings. A cross-reaction of one of the antibodies with an unrelated soluble protein is labelled by an asterisk. Note that ZC3HC1 was not detectable in this KO cell line and that TPR was only moderately reduced. (**C**) Time course of population growth of HeLa WT and ZC3HC1 KO cells. Data points with SDs were the mean results from three separate experiments, representing the growth of defined starting populations over 4 days. Note that the proliferation rate of the ZC3HC1 KO cells was similar to that of the WT progenitor cells. (**D**) IFM of co-cultured and cell cycle-synchronised HeLa WT and ZC3HC1 KO cells, harvested as a population enriched in cells in G2 and at the onset of mitosis. The cells were then immunolabelled for ZC3HC1 and CCNB1, and with an antibody targeting the phosphorylated serine 10 (S10p) of histone H3 (H3-S10p), as a very early hallmark for the onset of mitosis. Arrows mark cells at the G2/M transition point when staining for H3-S10p all along the NE is already prominent and when CCNB1 is readily imported into the nucleus within a time window of a few minutes. Arrowheads, by contrast, mark cells shortly before this time point. Note that while H3-S10 phosphorylation had already commenced in these arrowhead-marked cells, CCNB1 still appeared similarly well excluded from the nuclei of the WT and KO cells, even just before the actual end of G2. Bar, 10 µm. **(E**) IB of total cell extracts obtained from cell cycle-synchronised populations of WT and ZC3HC1 KO cells that had been harvested at indicated time points after the release from a thymidine-induced S-phase block. Immunolabelling for CCNB1 was performed on the PS-stained membranes shown here. Single asterisks mark BSA as part of trace amounts of culture medium not entirely removed from this experiment’s intentionally unwashed cells. Double asterisks mark a cross-reaction of the CCNB1 antibody, while CCNB1 itself, with a sequence-deduced Mr of about 48 kD, exhibited a gel-electrophoretic mobility equivalent to about 60 kD following SDS-PAGE by the current study’s commonly used method. Note that gradual CCNB1 accumulation over time, and its total amounts at corresponding time points, did not notably differ between WT and KO cells.

To this end, we first knocked out all ZC3HC1 alleles in the well-characterised HeLa subline commonly used in our study (Information SI 11) and managed to obtain a stable cell line that was completely devoid of ZC3HC1 polypeptides (Figure 6A,B and Figure S15(A,B1)). Like upon ZC3HC1 RNAi, such ZC3HC1 gene disruption resulted in some TPR now appearing distributed throughout the nuclear interior of these ZC3HC1 KO cells too (Figure 6A and Figure S15(B2)), yet with the amounts of such obviously soluble TPR polypeptides varying between individual cells within a population as well as between the individual sublines that we had isolated from the initial population of non-clonal KO cells (Figure S15(C1), and our unpublished data). However, the most apparent finding that turned out generally valid for all the ZC3HC1 KO cell lines isolated in the course of our study was the conspicuous, apparently similar degree of reduction in immunostaining for TPR at the NEs of such KO cells (Figure 6A, Figure S15(B2), and further below).

Apart from these phenotypes, though, the KO cells’ proliferation rate was similar to that of the original, ZC3HC1-positive WT version of this HeLa strain (Figure 6C). Furthermore, subcellular CCNB1 distribution within the ZC3HC1 KO cells appeared essentially indistinguishable from that in WT cells when having compared cells by IFM that had been cell cycle-synchronised and grown next to each other on the same coverslip (Figure 6D and Figure S15D) and when having quantified nuclear CCNB1 immunostaining (Figure S15E). Further in line with these findings, comparative IB of cell extracts from cell cycle-synchronised populations of KO and WT cells did not reveal any pronounced differences regarding the cellular amounts of CCNB1 at different time points of the cell cycle either (Figure 6E).

While the HeLa cell line [111] originates from a cervix adenocarcinoma [112] and thus is likely of mesodermal germ layer origin (e.g., [113]), we then also wanted to investigate how other human cell lines would cope with a complete loss of ZC3HC1. Therefore, we knocked out all ZC3HC1 alleles in U-2 OS cells too, which represent a hyper-triploid, chromosomally highly altered female cell line that stems from an osteosarcoma ([114]; see also characteristics for the U-2 OS [ATCC HTB-96] cell line at [115]) and is therefore of mesodermal origin as well. Furthermore, we disrupted the ZC3HC1 alleles in HCT116 cells, a near-diploid male cell line derived from a primary colon carcinoma (e.g., [116]), as a representative of the endodermal lineage. In addition, CRISPR/Cas9n-mediated disruption of the ZC3HC1 allele was also performed in the male, diploid, non-tumour cell line hTCEpi, which stems from the corneal epithelium [117] and is, therefore, a descendant of the ectoderm (Figure S15A).

In line with the outcome of ZC3HC1 RNAi experiments performed with these cell lines earlier, which had not revealed any essential role for ZC3HC1 either, we obtained stable ZC3HC1 KO strains for these cell lines (Figure S15(A,C2,F–H)). These exhibited subcellular distribution of CCNB1 just like in the WT progenitor lines and inconspicuous cell cycle progression within each line’s conventional growth medium (Figure S15J, and our unpublished data). Altogether, these data confirmed that aneuploid tumour cell lines of different tissue origins and a non-tumour cell line of normal diploid karyotype could get along quite well without ZC3HC1 under standard cell culture conditions.

Apart from their tolerance of ZC3HC1 deficiency, these additional ZC3HC1 KO cell lines had yet another feature in common. While some did and others did not exhibit an immediately apparent pool of TPR distributed throughout the nuclear interior, all of them displayed a conspicuously reduced intensity of TPR-immunolabelling at the NE (Figure S15F,G), just like it had been noted in HeLa cells lacking ZC3HC1.

### 3.5. Presence of ZC3HC1 Is Required for NE-Localisation of about Half the There Positioned Amount of TPR in Several Human Cell Lines

Recurrently having noted by then that the NE-associated amounts of TPR appeared reduced when ZC3HC1 was absent (e.g., Figure 5B, Figure 6A, and Figures S10 and S15(C2,F,G)), irrespective of whether this had been achieved by RNAi or CRISPR/Cas9n-mediated gene disruption, we set out to investigate this distinct phenotype in more detail.

**Figure 7:**
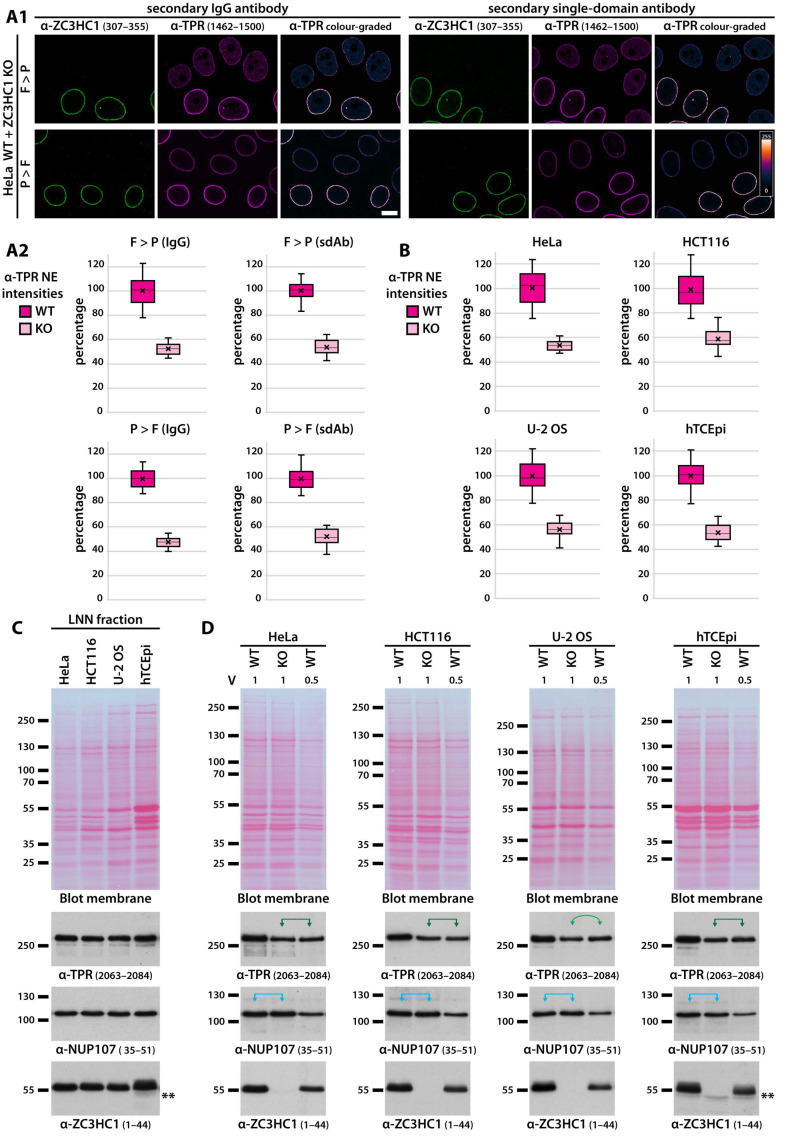
Quantification of the relative amounts of NB-associated TPR in the WT and ZC3HC1 KO cells of four different cell lines reveals a reduction by about half upon the absence of ZC3HC1. (**A**) Quantification of the relative amounts of NB-associated TPR, in the HeLa WT and ZC3HC1 KO cells, following different procedures of specimen preparation and immunolabelling. (**A1**) IFM of mixed populations of HeLa WT and ZC3HC1 KO cells that had been co-cultured, cell cycle-synchronised, and harvested in G1. Cells were permeabilised with TX-100 either after (F > P) or before fixation (P > F), the latter resulting in the removal of the nuclear pool of soluble TPR. Labelling was with guinea pig antibodies for ZC3HC1, and a mAb for TPR, with the latter detected with either fluorophore-conjugated IgGs or a mouse IgG1-specific single-domain antibody (sdAb). The micrographs for TPR are again also shown colour-graded, displaying that signal intensity relationships between the WT and KO cells’ NEs were very similar within the different types of specimens. Bar, 10 µm. (**A2**) Quantification of signal yields for immunolabelled TPR at the NEs of HeLa WT and ZC3HC1 KO cells, following specimen preparation and immunolabelling as in (A1). Randomly chosen NE segments for quantifications via ImageJ were from essentially all labelled cells in equatorial view within randomly chosen images of mixed populations of WT and KO cells, with such images obtained from the four differently prepared specimens (for details, see Figure S16A). Box plots display the relative signal intensity values, with the arithmetic means marked by x, with the ones for the WT set to 100%, and with the SDs provided. Note that the mean TPR signal yield for the KO cells’ ZC3HC1-free NEs was only about half the WT cells’ corresponding value, largely irrespective of whether cells had been permeabilised before or after fixation or whether fluorescence stemmed from secondary IgGs or sdAbs. (**B**) Quantification of TPR signal yields at the NEs of WT and ZC3HC1 KO cells of HeLa, HCT116, U-2 OS, and hTCEpi. WT and ZC3HC1 KO cells co-cultured as mixed populations and harvested in G1 had been permeabilised with TX-100 after fixation, and immunolabelled for ZC3HC1 and TPR, with the latter then detected with fluorophore-conjugated, mouse IgG-specific sdAb. The dataset for HeLa represented an independent experiment distinct from the corresponding one presented in (A). Like for (A2), the mean TPR signal yield for the KO cells’ ZC3HC1-free NEs was only about half the WT cells’ corresponding value, with this applying to all the four cell lines. (**C**) IB of LNN materials obtained from WT cells of lines HeLa, HCT116, U-2 OS, and hTCEpi. Loading amounts had been adjusted for similarity of NUP107 signal intensities to facilitate comparability of the NE-associated amounts of TPR and ZC3HC1. Immunolabellings were on the membrane shown here and on a duplicate with identical loadings. The ZC3HC1-unrelated cross-reaction in the LNN materials of WT and KO cells of line hTCEpi, already addressed in Figure S15I, is marked by a double-asterisk. Note that amount relationships between TPR, NUP107, and ZC3HC1 were rather similar in the four different cell lines. As an aside, also note that the degree of post-translational modification of ZC3HC1 can differ between cell lines, with such modifications generally most pronounced in the LNN materials isolated from interphase and G0 populations of hTCEpi cells. (**D**) IB of LNN materials obtained from WT and ZC3HC1 KO cells of lines HeLa, HCT116, U-2 OS, and hTCEpi, harvested shortly before having reached confluency, in order to compare the relative amounts of NE-associated TPR in the ZC3HC1 KO versus WT cells for each cell type. Loadings for each cell line represented the same proportion of the WT and KO cells’ adjusted LNN materials (1 V), next to which half of this amount from the WT cells’ LNN fraction (0.5 V) was loaded as well. Immunolabelling was on the upper and lower parts of the membranes shown here and on identical duplicates. A double-asterisk again marks the ZC3HC1-unrelated cross-reaction in the LNN materials of hTCEpi. Note that for each cell line’s WT and KO cells, the signal intensity for NUP107 in the 1 V lanes, marked by arrow-tipped brackets in blue, was essentially the same. By contrast, TPR signal intensities in the LNN fractions of the ZC3HC1 KO cells amounted, at most, to only about half of the intensity in the corresponding WT cells’ fraction obtained from about the same number of cells. Accordingly, the signal intensities for TPR in the 1 V lanes of the KO cells of lines HeLa, HCT116, and hTCEpi were highly similar to those for TPR in the 0.5 V lanes of the corresponding WT cells, with such relationships marked by arrow-tipped horizontal brackets in dark green. In contrast, in the 1 V lane of the U-2 OS KO cells, the TPR signal intensity was slightly lower than in the WT cells’ 0.5 V lane, with this then marked by the double-headed curved arrow in lighter green.

Initial assessments of the degree of TPR reduction at the NE as a result of ZC3HC1 deficiency were based on the quantification of immunofluorescence (IF) intensities for TPR at the NEs of neighbouring WT and KO cells of each cell type after having cell cycle-synchronised and grown them together on the same coverslip. The primary antibodies used for such IF staining had been controlled to only target TPR epitopes that are not modified post-translationally in these cell lines in interphase, neither in their WT nor their ZC3HC1 KO version (our unpublished data). Following IFM, this quantification approach revealed that the mean IF intensity for TPR at the KO cells’ NEs only reached approximately half the value for the corresponding WT cells. This finding applied to all four of the KO cell lines investigated, with the mean values for all of these cell types and the different experimental approaches ranging from 47% to 59%, with the mean of all reaching 52% of the WT cells’ mean amount (Figure 7A,B and Figure S16A,B). Such a reduction by about half was consistently noted, regardless of whether cells had been fixed before or after permeabilisation, whether primary antibodies had targeted different TPR epitopes, whether IgGs or sdAbs had been used as secondary antibodies, or whether cells had been harvested in G1 or G2 (Figure 7A,B, Figure S16A,B, and our unpublished data).

However, at this point, we nonetheless still had to keep in mind that IFM-based quantification of the relative amount of a target protein needs to be interpreted with some caution, especially when such a target, like TPR, might exist as a component of different parts of a subcellular structure (for further considerations on this topic, see Figure S16). Hence, in view of the caveats and some potential limitations regarding the diagnostic value of IFM-based quantifications, we aimed at complementing the IFM-based approach by quantitative IB of the cell lines’ LNN-enriched fractions.

We deemed such an IB approach suitable because several prerequisites were already fulfilled. First, we had found the LNN fractions of all the four WT cell lines to contain the cells’ almost entire amount of TPR (Figure S16(C1)). In addition, we had confirmed by different means that the residual population of NPC-associated TPR in the ZC3HC1 KO cells remained stably bound to the NPCs as long as NB-s conditions were applied (our unpublished data, to be presented in another context elsewhere), with this being in line with findings presented in Figure 7A, showing that extraction with TX-100 prior to fixation, in the presence of Mg^2+^ cations, does not result in conspicuous loss of the KO cells’ NE-associated amounts of TPR. Furthermore, with some of the TPR, ZC3HC1, and NUP107 antibodies available to us, it had turned out possible to define for these antibodies a narrow range, sufficiently overlapping, within which a linear relationship between protein amount and immunoblot signal intensity could be displayed for all three proteins, and this within the range of the LNN amounts from each cell line that could be loaded for SDS-PAGE (Figure S16(C2)).

Given these facts, we then first compared with each other the NUP107-normalised LNN-enriched fractions of the WT versions of the HeLa, HCT116, U-2 OS, and hTCEpi cells. This revealed that in all the four adherent cell lines, when harvested just before or just after having reached confluency, not only the relationships between the NE-associated amounts of TPR and NUP107 were rather similar but also those between TPR and ZC3HC1 (Figure 7C). This then also allowed us to conclude that these relationships were neither affected much by these cell lines’ extreme differences in karyotype nor by the lack of autosomal gene dosage adjustment in some tumour cell lines, like in HeLa (e.g., [118,119]). Only in proliferating populations of U-2 OS cells did we sometimes note that ZC3HC1 amounts relative to those of NUP107 and TPR were somewhat lower than in the other cell lines’ LNN materials (our unpublished data) while being similar again to the other lines’ amount ratios between TPR, ZC3HC1, and NUP107 once the U-2 OS populations had reached confluency. Furthermore, when cell populations had been kept in a confluent state for a while, we recurrently noted a tendency for a moderate increase in the amount ratios of TPR to NUP107 and of TPR to ZC3HC1 in some of the cell lines’ LNN materials. This applied to populations of hTCEpi cells, which cease to proliferate due to contact inhibition after having reached confluency, and especially held true for the U-2 OS subline used in the current study, which also exhibited a remarkable slowdown in population growth once cells had reached confluency.

However, while such a moderate increase in the amount ratio of NE-associated TPR to NUP107 and ZC3HC1 was sometimes also noted for some HeLa sublines, including this study’s most commonly used one, this was neither the case for other HeLa sublines nor for HCT116, with the latter instead featuring a small yet more obvious surplus of soluble ZC3HC1 than the other three cell lines (Figure S16(C1)).

Finally, we immunoblotted the LNN-enriched fractions obtained from these WT progenitor cell lines next to the same materials isolated from the four corresponding ZC3HC1 KO lines. Again, minor amount variations were only sometimes noted, with seemingly slightly more than half of the TPR materials missing in some of the KO cells’ LNN-enriched fractions, especially when cells had been harvested only after having been in a confluent state for some time. In general, however, and in particular when WT and KO cells had been harvested as still proliferating, sub-confluent populations, the NE-associated amounts of TPR were found to be indeed very similarly reduced by about half in all of the four KO cell lines (Figure 7D, and our unpublished data).

Overall, these findings corroborated our conclusion that, in general, at least half the amount of TPR positioned at the NEs of these different human cell lines depends on the presence of ZC3HC1.

## 4. Discussion

### 4.1. ZC3HC1 Qualifying as an NB Protein

The body of evidence that led to the conclusion that ZC3HC1 represents a true NB protein can be summarised as follows. First, ZC3HC1 is a ubiquitous protein presumably occurring in the vast majority of cell types of different germ layer origin that also contain TPR located at their NEs. Since TPR can be regarded as the main architectural component of the NB, such different cell types, too, are presumed to possess NBs appended to their NPCs. Furthermore, in all of the cell types inspected so far, ZC3HC1 polypeptides are colocalising with TPR at the nuclear periphery, and in many of these cell types, the subcellular distribution of ZC3HC1 appears essentially restricted to this position, as is also the case for TPR.

The commonality of ZC3HC1 and TPR is further highlighted in proliferating cells where both proteins concurrently disassemble from the NE in prophase and synchronously re-associate with the newly assembled NPCs very late at the end of mitosis. Interestingly, TPR and ZC3HC1 also have very similar half-lives in different human cell types [98], which in some cell types notably differ from the half-lives of NB anchor point proteins at the NR, like NUP107. This suggests that the turnover of TPR and ZC3HC1 might also be tied together in some way.

Furthermore, electron microscopy of *Xenopus* oocyte NEs immunolabelled for ZC3HC1 demonstrated that the protein is positioned, next to the NT of TPR, at the NB’s distal end, which comprises the actual TR and probably additional material appended to it. By contrast, ZC3HC1 turned out to be absent from the cytoplasmic pore complexes of the annulate lamellae that occur in various cell types and are also devoid of TPR, which further accentuates the NB membership of ZC3HC1.

Moreover, the NE-associated amounts of ZC3HC1 and TPR relative to each other are similar in conspicuously different cell types, which hints at some special stoichiometric relationship between both proteins. In fact, even in a cell type with no proliferative activity and barely any transcription, like the amphibian erythrocyte, we had found the amount ratio between NB-associated ZC3HC1 and TPR to be similar to that in proliferating cells of the same species. Furthermore, photobleaching and yeast two-hybrid experiments, which will be presented in a separate study, have revealed longer-lasting residency of ZC3HC1 at the NBs of living cells and in vivo interactions between ZC3HC1 and TPR that can be rather robust. In addition, co-IPs of ZC3HC1 with TPR from NE-assembly-competent egg extracts, as shown in the current study, and the co-isolation of TPR upon IP of ectopically expressed ZC3HC1 from human cell extracts (Figures S11(D2,D3), and S12A), represent a further indication that these proteins can engage in durable physical interactions both in vivo and in vitro.

However, the most remarkable piece of evidence that underscores the unique relationship between ZC3HC1 and TPR is the specific mutual dependency of these two proteins with respect to their binding to the NPC. Not only is TPR strictly essential for the NB-association of ZC3HC1, but also the absence of ZC3HC1 itself causes about half the amount of TPR, which is normally appended to the NEs of different types of cultures cells, to then no longer be located there. Such effects on TPR’s localisation at the NE are not notable upon RNAi of any of the other NB-resident proteins reported to date, among which are MAD1, GANP, and SENP1, which all appear dispensable for TPR’s association to an NPC (e.g., [32,37]; our unpublished data). So far, only those TPR-interacting proteins that are part of the NPC proper, like, e.g., NUP153, were known to play a role in the appendage of TPR to the NPC.

A possible explanation as to why ZC3HC1, as well as several other proteins normally resident at the NB, had not been detected in the course of former mass spectrometric studies on the NPC’s proteome, appears to be at hand. Evidence suggests that these proteins had been solubilised during the process of isolating the cellular materials that eventually were analysed, which also included materials primarily consisting of NPCs. While such pioneering studies had led to the first, almost complete descriptions of the protein composition of the NPC in budding yeast and rats (e.g., [56,120]), the underlying isolation procedures likely entailed losing some of those proteins that were more peripherally attached to the NPC.

All taken together, we see no obligatory contradiction between ZC3HC1 being a regular NB component and the instability of this relationship when exposed to certain non-physiological conditions. We actually do not regard it as unreasonable that the affinity between ZC3HC1 and the NB might even vary during naturally occurring alterations of environmental conditions. Being capable of responding to such changes, thereby rendering a usually tight interaction sufficiently flexible on demand, might allow for dynamic rearrangements that could be of physiological relevance. We also regard it as noteworthy that the biochemical stability of certain interactions between the NB and ZC3HC1 can even differ to some extent between different cell types (our unpublished data). In fact, we deem it possible that the interplay between some of the NB-resident amounts of TPR and ZC3HC1 is by default either more or less flexible in the one or other cell type, and that also signalling events can modulate these interactions.

Even though the findings made in the current investigation are at variance with formerly reported perceptions regarding the function and the binding partners of ZC3HC1, we feel confident in concluding that ZC3HC1 is neither a naturally occurring component of SCF complexes nor required for the degradation of CCNB1. After having scrutinised the most conflicting issues experimentally (Figures S11–S13), we complemented this by also discussing each of them point by point (see Supplemental Discussion).

### 4.2. ZC3HC1 as a Non-Essential Protein at the NB

Apparently, ZC3HC1 is neither essential for mammalian cell line proliferation in general nor for the growth and viability of any of those model organisms in which the gene for the likely ZC3HC1 homolog has been inactivated so far. For example, no or only minor effects regarding cell growth during standard culture conditions were observed upon knocking out Rsm1p and Pml39p in *Schizosaccharomyces pombe* and *Saccharomyces cerevisiae*, respectively (e.g., [34,121]), with these proteins being the unequivocal homologs of ZC3HC1 in these species, as we will illustrate in a separate study. Similarly, after having destructed the ZC3HC1 gene in several human cell lines by CRISPR/Cas9n technology, we did not note any generally obvious growth phenotype, in line with genome-scale CRISPR KO screens in human tumour cell lines [122]. 

Furthermore, deficiency of the apparent ZC3HC1 homolog (C49H3.9) in *C. elegans* was neither found to be lethal nor was any other phenotype observed so far (e.g., [123,124]). Moreover, destruction of the ZC3HC1 gene in the mouse is not necessarily lethal either. However, in this model organism, the knockout comes along with an increased rate of embryonic lethality, as homozygous KO animals per litter can be lower than expected, and with several phenotypic abnormalities in the adult KO mice. Among these are the sterility of males, often reduced body weight, certain skeletal malformations occasionally observed in some of the ZC3HC1-deficient animals, mild hypoplasia of the bone marrow and ovary, as well as other phenotypes, yet also with variabilities between KO mice of different genetic background ([104,105,125,126], and our unpublished data). However, adequate housing conditions provided, some of the animals among a population of homozygous ZC3HC1 KO mice can reach high ages in seemingly good physical condition despite being truly ZC3HC1-deficient (our unpublished data).

Altogether, these findings raised the fundamental question of why the removal of this unique protein, with no apparent substitute in sight that might compensate for its loss, actually seems to affect neither the routine performance of unicellular organisms like *S. cerevisiae* and *S. pombe* nor that of various types of human cells. While the phenotypes of the ZC3HC1 KO mouse might hint at some role in cell type-specific differentiation processes, with ZC3HC1 either directly involved or indirectly via the ZC3HC1-dependent pool of NB-associated TPR, it seems unlikely that the possibly hundreds of different cell types that constitute a mammal, of which probably most possess ZC3HC1 at the NE, should not share a ZC3HC1 function that is common to most if not all of these cell types. Consequently, one might expect these different cell types to exhibit a common phenotype once they are devoid of ZC3HC1 in a KO organism. In fact, given that ZC3HC1 homologs exist as unique proteins in numerous species all across the eukaryotic realm, as will be presented in a separate study, one might even expect some species-spanning universal function being the raison d’*ê*tre for this evolutionarily conserved protein. 

Nonetheless, this assumption does not exclude the possibility that cell type- or species-specific functions might exist as well. However, it is conceivable that a ZC3HC1 homolog, and the homologous TPR polypeptides it might also in other species enable to occur NB-appended, will only unveil some of its properties when the corresponding model organism is exposed to environmental stress factors.

### 4.3. ZC3HC1 and TPR as a Functional Ensemble at the NB?

Among the next questions inevitably arising is the one whether ZC3HC1 can only be looked upon as part of some functional ensemble together with TPR, or whether it might also act as a standalone protein with additional autonomous, i.e., essentially TPR-independent functions that simply need to be executed at the nuclear periphery. In this latter scenario, ZC3HC1 would use the TPR polypeptides at the NB as anchor points, which in principle could then even be regarded as exchangeable for other proteins as long as they would provide such binding sites too. Such anchor points would then enable ZC3HC1 to use the NB as an operational platform for distinct tasks that have to be fulfilled at this location, perhaps by transiently interacting there with yet other proteins. Currently, however, none of the data available to us is pointing at autonomous ZC3HC1 functions that could be effectively uncoupled from TPR and would pertain to all cell types and species. Thus, at this point, one unavoidably arrives at the other scenario and the question of what a ZC3HC1-TPR ensemble at the NB might be good for.

From among the ideas hatched in this context so far, we are currently considering especially two main scenarios that are not necessarily mutually exclusive, in which ZC3HC1 does not function without TPR. In the one, we regard the NB-bound ZC3HC1 as a protein that would act as a TPR insulator, and in the other, ZC3HC1 would play some role in the NB assembly process or its maintenance.

In the first scenario, ZC3HC1 would bind to distinct parts of TPR to prevent other proteins from binding there. The unmasking of such binding sites, making them accessible for other proteins, would occur only on demand, i.e., in a signal-induced manner. However, while we regard it as tempting to envision such a concept of ZC3HC1 perhaps acting as an adjustable insulator of TPR, this still needs to be scrutinised in further experimental detail.

Unlike this concept of controllable masking of TPR segments, the ZC3HC1 polypeptides in the other scenario would play, again not necessarily in a mutually exclusive manner, some role in recruiting distinct amounts of TPR or as architectural elements of the NB. As such, one could imagine ZC3HC1 to be required for stabilising direct interactions between the different TPR subpopulations at the NB, thereby allowing them to be more stably held in place. ZC3HC1 might even be needed for directly recruiting the additional amounts of TPR and tethering these to the NB, thereby acting as a link between the different populations of TPR. Both of these scenarios are based on our finding, valid for several different cell types, that there are at least two large subpopulations of NB-associated TPR polypeptides, of which the one is only found appended to the other when ZC3HC1 is present. Inevitably, this leads to the next question, namely, what such second-population TPR at the NB might be good for. And once again, several scenarios are conceivable.

In one of those only relating to the architecture and stiffness of the NB, the additional TPR polypeptides would play a role in enhancing general NB stability. Even though it remains to be investigated in all detail in how far lack of about half the total TPR amount might sometimes affect the overall stability of the NPC-anchored NBs in a living ZC3HC1 KO cell, one could imagine that the ZC3HC1-dependent additional pool of TPR could result in a more robust NB. The higher rigidity would then perhaps also allow for better maintaining NPC-adjacent heterochromatin exclusion zones (HEZs), which formerly have been shown to depend on TPR [11].

In again other scenarios, one would look upon the ZC3HC1-dependent TPR polypeptides at the NB as being there in order to increase the NB’s functional repertoire in a variety of ways; for example, by enabling the NB proper to modulate its architecture and performance on demand, like by allowing the NB to more flexibly adopt different conformations, or by simply providing more TPR polypeptides as binding sites for other molecules. In this latter context, we can imagine that if an NPC-associated TPR polypeptide functions as a temporary anchor-point for a subset of genes or as a transient docking site for some but not other cargo molecules on their way to and from the NPC, the ZC3HC1-conferred stabilisation or attachment of additional TPR polypeptides would simply provide additional ones of such anchor points or docking sites close to the NPC. There they might participate in perinuclear transcription regulation, for example, of stress-responsive genes whose transcripts need to be most efficiently exported upon acute demand, or in fine-tuning distinct pathways for different types of cargos that have to transit the NB.

Within these scenarios, we are also considering the ideas that such ZC3HC1-dependent additional pools of TPR polypeptides can form the kinds of additional NB-like structures that can be seen appended to the TR of the NPC-attached NB in oocytes (see Figure S7) and that such TPR- and ZC3HC1-containing cylindrical arrangements can also exist in somatic cells. There, too, these structures would then allow for an outreach deeper into the nuclear interior and possibly enable the formation of those NPC-adjacent HEZs that have been shown to go beyond the dimensions of the NPC-anchored NBs commonly regarded as prototypic. In fact, such NPC-associated long cylinders have been noted to, in principle, also exist in human cell lines like HeLa (e.g., Supplemental Figure S1 in [11]). In this context, we also consider it noteworthy that TPR has recently been found to play a crucial role in heterochromatin-rearrangements that are triggered by oncogenic signalling [127], and this now raises the question of whether ZC3HC1, and those TPR polypeptides depending on ZC3HC1, might be the proteins enabling such processes.

Moreover, we also do not consider it unrealistic that the second pool of TPR polypeptides could contribute to the formation of a meshwork, composed of TPR’s largely unstructured carboxy-terminal “tail” domain [14,128] (Figure S3C, and our unpublished data). Such a meshwork could then be denser or more expanded than if it were formed only by those TPR polypeptides anchored to the NPC independently of ZC3HC1. In fact, we can even imagine a scenario in which the resulting meshwork of TPR tail domains could exhibit sieve-like properties similar to but nonetheless also distinct from those of the FG-repeat nucleoporins that enable the NPC to act as a selective permeability barrier (e.g., [129,130]). An NB-associated sieve might also allow for the selective and perhaps facilitated passage of some molecules while denying access for others, and such a second sieve might be more functional in the presence of the ZC3HC1-dependent additional amounts of TPR.

Further work will now need to address and scrutinise these different scenarios in all detail. While we have characterised ZC3HC1 in the current study as a novel genuine component of the NB that engages in an interdependent relationship with the NB’s scaffold protein TPR, coming studies will need to unveil how close and complex this interaction might be.

## Data Availability

The data presented in this study are available on request to the corresponding author.

## Supplementary Materials

The following are available online at https://www.mdpi.com/article/10.3390/cells10081937/s1. Supplemental Information SI 1–11. Figure S1: Characterisation of antibodies for *Xenopus* proteins ZC3HC1, TPR, and NUP153. Figure S2: Subcellular location of xlZC3HC1 in XL-177 cells. Figure S3: Background information, preconditions, procedural approaches, and resulting datasets for distance measurements between NPCs, NBs, and immunogold-labelled components visualised at *Xenopus* oocyte NEs by SEM. Figure S4: The appearance of subcellular features of late-stage II and early-stage III oocytes, following high-pressure freezing, freeze-substitution in the absence of chemical fixatives, and embedding in the hydrophilic resin K4M. Figure S5: Suitability assessment of high-pressure frozen and freeze-substituted late-stage II oocytes for immunolabelling, using IFM for rapid screening of antibody performance on ultrathin sections and iTEM for the identical specimens’ subsequent inspection with selected antibodies. Figure S6: Guidelines for and results of distance measurements in post-embedding iTEM of K4M-embedded oocytes. Figure S7: Additional NB-like structures stacked on top of the NB proper. Figure S8: IFM and IB of ZC3HC1 upon shutdown of gene transcription in human tumour cells. Figure S9: Disassembly during entry into mitosis and post-mitotic reassociation of ZC3HC1 and TPR to the newly assembled NE in proliferating cells. Figure S10: IFM of several NPC components in ZC3HC1-deficient HeLa cells. Figure S11: ZC3HC1 in interphase neither being part of an SCF complex nor a binding partner of SKP1 under normal circumstances in vivo. Figure S12: Evidence for ZC3HC1 neither binding CCNB1 nor playing a role in directly regulating the cellular levels of CCNB1 in proliferating cells. Figure S13: ZC3HC1 deficiency neither triggers apoptosis in different human cell types nor enhances apoptotic phenotypes upon induction of apoptosis in HeLa cells. Figure S14: ZC3HC1 is not required for cellular housekeeping activities of cultured cells in interphase, with cell culture expansion, subcellular distribution of poly(A)+ RNA, bulk translational activity, bulk transcriptional activity, rates of replication, and the bulk of nucleocytoplasmic transport remaining largely unperturbed in ZC3HC1-deficient cell populations. Figure S15: Characterisation of human cell lines of mesodermal, endodermal, and ectodermal origin following CRISPR/Cas9n-mediated *ZC3HC1* gene knockout. Figure S16: Approximations of the relative amounts of NE-associated TPR in WT and ZC3HC1 KO cells. Supplemental Discussion. Supplemental Material and Methods. Table S1: iSEM evaluation datasets. Table S2: Antibodies. Table S3: siRNAs. Table S4: Cell lines. Table S5: Mammalian expression vectors. Supplemental References.

## Abbreviations

aa, amino acids; AL, annulate lamellae; ALPC, annulate lamellae pore complexes; BSE, backscattered electron; CRISPR/Cas9n, Clustered Regularly Interspaced Short Palindromic Repeats/CRISPR-associated protein 9 nickase; CT, carboxy-terminus; FA, formaldehyde; GA, glutaraldehyde; IB, immunoblotting; IFM, immunofluorescence microscopy; IGP, immunogold particle; iSEM, immuno-scanning electron microscopy; iTEM, immuno-transmission electron microscopy; KD, knockdown; KO, knockout; LNN, lamina-NPC-NB; LUT, look-up table; NB, nuclear basket; NB-d, nuclear basket-destabilising; NBLS, NB-like structures; NB-s, nuclear basket-stabilising; NE, nuclear envelope; NPBD, nuclear pore complex-binding domain; NMBD, nuclear membrane-binding domain; NPC, nuclear pore complex; NR, nuclear ring; NT, amino-terminus; NUP, nucleoporin; PS, Ponceau S; RNAi, RNA interference; SD, standard deviation; SEM, scanning electron microscope/microscopy; siRNA, small interfering RNA; TBD, TPR-binding domain; TEM, transmission electron microscope/microscopy; TR, terminal ring.
