# Supplementary material for: ZC3HC1 Is a Novel Inherent Component of the Nuclear Basket, Resident in a State of Reciprocal Dependence with TPR
Source: Cells. 2021 Jul 30;10(8):1937. doi: 10.3390/cells10081937 (PMC8393659; doi:10.3390/cells10081937)
Supplement: Supplementary file 1 [file cells-10-01937-s001.zip › cells-1292547-supplementary.pdf]

cells

Article

ZC3HC1 is a Novel Inherent Component of the Nuclear Basket, Resident in a State of Reciprocal Dependence with TPR

Philip Gunkel, Haruki Iino, Sandra Krull and Volker C. Cordes *

Max Planck Institute for Biophysical Chemistry, D-37077 Göttingen, Germany;
Philip.Gunkel@mpibpc.mpg.de (P.G.); h.iino@lms.mrc.ac.uk (H.I.); sandra.krull@uni-tuebingen.de (S.K.)
* Correspondence: Volker.Cordes@mpibpc.mpg.de; Tel.: +49 (0)551 2012404

Supplemental Information, Data, Material and Methods, and Discussion

Table of content

1. Supplemental Information

1.1 Early considerations regarding the subcellular localisation of ZC3HC1 in *Xenopus* oocytes. 4
1.2 Choice of frogs and stage V oocyte NEs for iSEM. 5
1.3 Considerations regarding iSEM data and their strength of evidence. 6
1.4 Choice of oocytes for HPF and post-embedding iTEM. 8
1.5 Assessment of antibody performance by IFM, on ultrathin sections of resin-embedded materials, prior to their use in post-embedding iTEM. 9
1.6 Spread IGP distribution in post-embedding iTEM either due to non-perpendicular cross-sections through a resin-embedded *Xenopus* oocyte NE or reflecting natural NB properties. 10
1.7 Reflections on the amount relationships between ZC3HC1, TPR, and NPC scaffold proteins in different cell types. 12
1.8 Destabilisation of ZC3HC1-NB interactions during cell fractionation. 15
1.9 Reflections on co-immunoprecipitation of TPR and ZC3HC1 from the extracts of cultured cells and oocytes and from post-mitotic *Xenopus* egg extracts. 17
1.10 Considerations regarding RNAi in cell lines of the allotetraploid organism *Xenopus laevis*. 18
1.11 A HeLa subline termed P2 and its use for CRISPR/Cas9n-editing. 19

2. Supplemental Figures

1. Characterisation of antibodies for *Xenopus* proteins ZC3HC1, TPR, and NUP153. 20
2. Subcellular location of xlZC3HC1 in XL-177 cells. 28
3. Background information, preconditions, procedural approaches, and resulting datasets for distance measurements between NPCs, NBs, and immunogold-labelled components visualised at *Xenopus* oocyte NEs by SEM. 30
4. The appearance of subcellular features of late-stage II and early-stage III oocytes, following high-pressure freezing, freeze-substitution in the absence of chemical fixatives, and embedding in the hydrophilic resin K4M. 36
5. Suitability assessment of high-pressure frozen and freeze-substituted late-stage II oocytes for immunolabelling, using IFM for rapid screening of antibody performance on ultrathin sections and iTEM for the identical specimens' subsequent inspection with selected antibodies. 38
6. Guidelines for and results of distance measurements in post-embedding iTEM of K4M-embedded oocytes. 42
7. Additional NB-like structures stacked on top of the NB proper. 51
8. IFM and IB of ZC3HC1 upon shutdown of gene transcription in human tumour cells. 53
9. Disassembly during entry into mitosis and post-mitotic reassociation of ZC3HC1 and TPR to the newly assembled NE in proliferating cells. 56
10. IFM of several NPC components in ZC3HC1-deficient HeLa cells. 58
11. ZC3HC1 in interphase neither being part of an SCF complex nor a binding partner of SKP1 under normal circumstances *in vivo*. 61
12. Evidence for ZC3HC1 neither binding CCNB1 nor playing a role in directly regulating the cellular levels of CCNB1 in proliferating cells. 82
13. ZC3HC1 deficiency neither triggers apoptosis in different human cell types nor enhances apoptotic phenotypes upon induction of apoptosis in HeLa cells. 87
14. ZC3HC1 is not required for cellular housekeeping activities of cultured cells in interphase, with cell culture expansion, subcellular distribution of poly(A)+ RNA, bulk translational activity, bulk transcriptional activity, rates of replication, and the bulk of nucleocytoplasmic transport remaining largely unperturbed in ZC3HC1-deficient cell populations. 90
15. Characterisation of human cell lines of mesodermal, endodermal, and ectodermal origin following CRISPR/Cas9n-mediated ZC3HC1 gene knockout. 98
16. Approximations of the relative amounts of NE-associated TPR in WT and ZC3HC1 KO cells. 108

3. Supplemental Discussion

3.1 Former conception of ZC3HC1 as a protein with an anti-apoptotic function. 113
3.2 Former perception of ZC3HC1 as an F-box protein. 114
3.3 Former perception of ZC3HC1 as a stable component of an E3 ubiquitin ligase of the SCF-type. 115
3.4 Former perception of ZC3HC1 as a protein binding CCNB1 for subsequent degradation. 117
3.5 Former perception of ZC3HC1 as a binding partner of the tyrosine kinase NPM-ALK. 118

4.Supplemental Material and Methods

4.1 Production of antibodies. 119
4.2 RNA isolation from *Xenopus* oocytes, and synthesis and cloning of cDNAs. 119
4.3 Feeding specifications for *Xenopus laevis* females. 119
4.4 IFM of cryostat and ultrathin sections. 120
4.5 Isolation of AL-enriched subcellular fractions, cytosol, and NEs from *Xenopus* oocytes. 120
4.6 Freeze-substitution. 121
4.7 Production of mitotic and nuclear assembly-competent *Xenopus* egg extracts. 122
4.8 Covalent binding of antibodies to Protein A-coupled magnetic beads. 122
4.9 Immunoblotting. 123
4.10 Culturing of cell lines. 123
4.11 Induction of apoptosis and TUNEL assay. 124
4.12 Cell cycle synchronisation of human cells. 124
4.13 Transfection of *Xenopus* cells with siRNAs and of human cells with expression vectors. 124
4.14 Immunoprecipitation of ectopically expressed proteins. 125
4.15 EU and EdU incorporation experiments. 126
4.16 IFM-compatible fluorescence in situ hybridisation (FISH). 126

5. Supplemental Tables

1. iSEM evaluation datasets. 128
2. Antibodies. 130
3. siRNAs. 132
4. Cell lines. 133
5. Mammalian expression vectors. 134

Supplemental References 135

1. Supplemental Information

1.1. Supplemental Information 1. Early considerations regarding the subcellular localisation of ZC3HC1 in Xenopus oocytes.

Since AL lack not only NB proteins but also some of those that are directly appended to the NE's nuclear membrane, the finding of ZC3HC1 not being located at the AL did not suffice on its own for already classifying it unequivocally an NB-associated protein. Furthermore, even though we had found ZC3HC1 detached from the NE together with known NB proteins upon NB disassembly, we could not yet exclude, at this early point in our investigation, the possibility that ZC3HC1 might be attached to another component of the NE, as a protein whose localisation there would just coincidentally be also affected by those conditions that lead to NB disassembly. In fact, we had noted this being the case for a few other NE-associated proteins that eventually turned out not to be NB-associated (our unpublished data). Moreover, even though IFM had revealed that ZC3HC1 is a protein conspicuously enriched at the oocyte's NE (Figure 1C and Figure S1F), manual subfractionation of oocyte nuclei had shown that ZC3HC1 in this cell type was not exclusively located there. While the mass spectrometry-based estimation of the relative copy numbers of those ZC3HC1 polypeptides that were robustly tethered to the cleansed NEs had indicated early on that such numbers were apparently very similar to those of several NPC proteins, the IB of such NE materials loaded next to the same oocytes' nuclear contents had revealed ZC3HC1 also to be located within the oocytes' nuclear interior, with such amounts notably varying between different batches of oocytes. Indeed, having compared the subnuclear occurrence of NPC and NB proteins in the oocytes of frogs of different ages and bodyweights, it had become evident that ZC3HC1 was generally among those NPC and NE-associated oocyte proteins for which varying, yet always noticeable amounts were commonly detectable also within the nuclei's manually demembranated nuclear contents. While the latter also included pelletable ZC3HC1- and TPR-containing material that had possibly been detached from the NE, a significant proportion of ZC3HC1 within such nuclear content fractions also existed in soluble form, likely representing a naturally occurring subpopulation (see also Figure S11A2). Not until later did we find this to be a feature of the oocytes that notably differed from other cell types in which ZC3HC1 turned out to be located virtually only at the NE (see further below). Therefore, and even though such additional ZC3HC1 polypeptides deeper within the oocyte nucleus had not been discernible by conventional IFM, which probably had to do with the oocyte's enormous nuclear volume and the amounts of soluble ZC3HC1 diluted therein beyond unambiguous detectability by microscopy, we could not claim ZC3HC1 to be an exclusively NE-bound protein in the *Xenopus* oocyte.

1.2. Supplemental Information 2. Choice of frogs and stage V oocyte NEs for iSEM.

Xenopus laevis NEs from stage V oocytes were isolated from slim frogs that had been kept on a legitimate, nutrient-balanced feeding schedule with caloric restriction. Such diet allowed to encounter far less of the conspicuous accumulations of NE-associated materials of different kinds, including fibrillar materials appended to the NB's distal ends and projecting deeper into the nuclear interior [e.g., 1–7], that can be especially abundant in the fully grown stage VI oocytes of heavyweight frogs fed on a high-caloric protein-rich diet. Therefore, feeding conditions that allowed for encountering less of such seemingly stockpiled materials consequently facilitated the examination of the NB proper and allowed for a more unequivocal evaluation of the iSEM data. In the case of the iSEM data presented in the current study, all immunogold-labellings were performed in parallel to allow for their unambiguous comparison, using for this purpose oocyte NEs from a frog of 112 g bodyweight. Additional labellings performed for data reproducibility, using oocytes from frogs weighing between 105 g and 135 g, yielded essentially indistinguishable results regarding immunogold distributions at their NEs (data not shown).

1.3. Supplemental Information 3. Considerations regarding iSEM data and their strength of evidence.

In general, iSEM allows for an expeditious three-dimensional visualisation of the immunogold-labelled target, provided that the object of interest can be exposed to imaging via secondary electrons (SEs), as is the case with the manually isolated oocyte NE. The two-dimensional coordinates of individual immunogold particles (IGPs), which in conventional iSEM are commonly not identifiable as such in the SE image, as explained below, are thereby usually obtained via the detection of their high-energy backscattered electrons (BSEs). The latter are then superimposed onto the topographic images, obtained in parallel via detection of the ejected low-energy SEs. However, ample caution needs to be exercised when interpreting such iSEM data because a single BSE image generally does not allow to vertically resolve, i.e., along the view axis, the actual position of the IGP from which the BSEs have been ejected. While one can nonetheless assume that the coordinates of BSE signals superimposed onto the SE image of an object that is solid and only accessible from the top will reasonably well represent the actual position of the corresponding IGP, one encounters a different situation with coarse-meshed objects like the NB. In principle, such objects allow for being labelled from different sides and even allow for sites beneath them being accessible for antibodies. In conventional iSEM of the NB, it is mostly not possible to distinguish between these scenarios, namely whether an IGP is located on the NB's top or beneath it, simply because the typical procedure of coating the immunolabelled specimens and their IGPs with an additional electron-conductive layer of heavy metals generally prevents unambiguous detection of even those IGPs in the SE modus that are actually located on top of the NB. This, in turn, can lead to situations in which immunolabelled components of the NPC proper can yield detectable BSE signals that are then wrongly assigned to parts of the NB, like its TR, even though they stem from sites further beneath. In summary, it needs to be said that if an IGP is only detectable by the BSEs ejected from it, its actual position in three-dimensional space is in principle only deducible from a series of SE and BSE images acquired at different tilt angles.

In the current study, we therefore aimed at visualising the IGPs also in the SE image to distinguish between the visible ones and those concealed by parts of the NB towering above them. To this end, we have altered several steps of the conventional iSEM procedures, including specimen preparation, mounting of the specimen-laden silicon chip, and microscope settings. Altogether, this rendered additional heavy metal-coating of specimens via electron beam-evaporation or magnetron-sputtering superfluous while nonetheless allowing for different types of specimens to be inspected at elevated cathode currents and acceleration voltages of 10-15 kV, without encountering major electrostatic charging.

Regarding the particular target proteins inspected in the current study, the modified iSEM procedure and the omission of conventional heavy metal-coating allowed for clearly assigning the vast majority of high-energy BSE signals to the actual IGPs from which these BSEs originated because these IGPs, no longer buried beneath a layer of heavy metal-coating, were then clearly discernible via imaging with the low-energy SEs too. In fact, when inspecting series of images acquired from such IGP-decorated specimens at different tilt angles as a control, we found strict site fidelity of the BSE signals relative to the IGPs detected in the SE images of the NBs (data not shown).

Apart from being a precondition for being able to evaluate the iSEM data conclusively, the omission of heavy metal-coating also made it possible to more easily visualise further structural peculiarities at the NB's distal end, like the so-called TR actually not being a simple ring but rather a far more complex arrangement of fibrillar elements which we deem mainly composed of the bifurcating parts of homomeric assemblies of TPR. Branching off from the longitudinal NB fibrils, these parts of TPR are regarded then adjoining, in a staggered manner, with the corresponding parts of the neighbouring NB fibrils [8,9; our unpublished data, and Figure S3B,C].

While we regarded the modified iSEM procedure as having improved the diagnostic value of our immunogold-labellings of the NB notably, we nonetheless had to keep in mind that iSEM is a method that is, in principle, more closely comparable to pre-embedding than to post-embedding immunogold-labelling in iTEM; in the sense that like in pre-embedding iTEM, the mean of the IGPs' positions in iSEM does not necessarily reflect the exact position of a target site. This has several reasons in combination, with the following two probably being the most determining ones. The first one relates to the relatively large sizes of the IgGs and the commonly used colloidal gold particles, which together can allow for possible distances of up to about 20 nm between a target site and the centre of an individual gold particle when using intact primary antibodies, which are commonly IgGs, and 10 nm-gold-coupled $F(ab')_2$ fragments for secondary labelling. The second reason is that the target of interest in iSEM often is part of a three-dimensional object, just like in pre-embedding iTEM. This means that such an object will not necessarily be equally well accessible for an antibody from all sides. Therefore, steric constraints can dictate a certain angle preference by which the IgG will have to bind to it, causing a general offset between the epitope and the mean of the IGP positions.

1.4. Supplemental Information 4. Choice of oocytes for HPF and post-embedding iTEM.

Late-stage II oocytes were used for iTEM for two main reasons. (1) They were still small enough to be high-pressure frozen without the formation of larger ice crystals, a recurrent problem encountered when using the larger oocytes of the later stages of oogenesis for HPF (our unpublished data). In fact, stage IV and V oocytes, the latter having to be mounted defolliculated and thereby flattened out into aluminium planchets with greater cavity depths, hardly allowed for proper intracellular vitrification of large areas sufficiently deep within the cell, including perinuclear territories; an effect sometimes even observed with late-stage III oocytes. (2) In stages II and III of oogenesis, most NBs still appear to be lacking distinct types of proteinaceous appendices that can be very prominent later during oogenesis (see also Information SI 2), where such appendices of cylindrical shape were already known to contain TPR polypeptides [10]. While especially conspicuous in heavyweight frogs, such appendices are occasionally also visible, perhaps to some extent also dependent on frog age, in the late- and sometimes even in the earlier-stage oocytes of slim frogs (our unpublished data, but also see Figure S7). At least in conventional post-embedding iTEM, i.e., when using oocyte material chemically fixed prior to the embedding in resin, any frequent occurrence of such NB appendices was known to complicate the assignment of TPR IGPs to distinct parts of the actual NB proper (our unpublished data), especially since these structures, like the prototypic NB itself, are generally not visible in the ultrathin sections of the resin-embedded specimens (see also Information SI 6).

We considered it appropriate to compare iTEM data obtained from younger oocytes with the stage V oocytes' iSEM data because we had inspected NB morphology in late-stage II and early-stage III oocytes, as visualised by SEM, and found it to be very similar to that of the NB proper in the stage V oocyte of a slim frog (an exemplary SEM image of the nuclear side of a late-stage II oocyte's NE, as compared to that from a stage V NE, is presented as Figure S3G). Furthermore, for additional control, we had repeated iSEM on late-stage II and early-stage III oocyte NEs with some of the xlTPR, xlNUP153 and xlZC3HC1 antibodies on a smaller scale. Essentially confirming our former iSEM findings, this did not reveal any conspicuous differences between the iSEM datasets for stage II/III and V oocytes (our unpublished data).

1.5. Supplemental Information 5. Assessment of antibody performance by IFM, on ultrathin sections of resin-embedded materials, prior to their use in post-embedding iTEM.

Since only epitopes exposed on the surface of an ultrathin section can be labelled with antibodies in post-embedding immuno-TEM, the density of IGPs at labelled NPCs is generally low, even if such antibodies exhibit high affinity for their targets. This problem of overall low IGP density is especially evident when using single epitope-targeting antibodies specific for only one NPC or NB component [e.g., 9]. After all, even in an ideally sectioned NPC, only epitopes corresponding to at most two-eights of the eight-fold rotationally symmetric NPC, or its NB, are exposed on the section surface. Nonetheless, in the best case, the labelling is so specific that it is instantaneously evident that only the NPC and no other structure has been immunolabelled. Only in this case, it might appear acceptable to accumulate many micrographs only showing IGPs located at or near the NE, in order to determine their mean position relative to the NPC or NB. However, for those antibodies that also produce a certain amount of undesired background-labelling, and in particular for such that at the same time exhibit even less NPC-labelling, due to lower affinity or lower antibody concentrations, it demands greater efforts for unambiguously proving that IGPs detected in the proximity of some relatively few labelled NPCs truly represent specific labelling. This is so because for accumulating a robust and conclusive dataset, it is not adequate to merely document IGPs located at a site of interest, like the NE, because this would always result in a seemingly specific enrichment of IGPs at this site, in principle even with an antibody merely producing background-labelling. Instead, one needs to systematically collect images of all detected IGPs and determine their positions relative to the site of interest. This means that images for all IGPs within the field of vision have to be taken when moving along the cross-sectioned NE during its microscopic inspection, irrespective of how far away these IGPs are from the NE. Only such datasets can answer whether those IGPs at the NE reflect some region-specific enrichment that can be regarded as statistically significant. While we propose this to be the general *modus operandi* for every antibody, it is instantaneously evident that this can turn out to be a very time-consuming enterprise [e.g., 9]. In particular, if nothing is known about the performance of a collection of antibodies in post-embedding iTEM, even though they might perform perfectly well in IFM or other pre-embedding labelling procedures like iSEM, the systematic testing of their suitability for the immunolabelling of resin-embedded materials, in which proteins had to endure procedures that might have negatively affected epitope integrity, can be very tedious, when having to be done via immunogold-labelling and evaluation by TEM.

Therefore, in the current study, we routinely pre-screened our collections of NPC and NB antibodies for their performance on the resin-embedded materials by IFM, using a conventional confocal laser scanning microscope (some examples are presented in Figure S5). Apart from also allowing for rapidly assessing ranges of suitable antibody concentrations, this approach made it possible to selectively choose those antibodies for subsequent testing and performance-optimisation in immunogold-labellings that had already revealed adequate NE-labelling and little overall background in IFM on ultrathin sections of the same specimen that was then also to be inspected by iTEM.

1.6. Supplemental Information 6. Spread IGP distribution in post-embedding iTEM either due to non-perpendicular cross-sections through a resin-embedded Xenopus oocyte NE or reflecting natural NB properties.

When comparing in post-embedding iTEM the distribution of IGPs that have labelled a target directly at the NPC, in the following called A, with the distribution of IGPs for an NPC-distal site, here called B for a target like the TR of the NPC-attached NB, one needs to take into consideration that a broader distribution for the IGPs for B does not necessarily mean that the sites at which B polypeptides occur are distributed across a larger area than the sites for A. Instead, even if the distance between A and B would not vary between different NPCs, a wider distribution of the IGPs for B can arise for technical reasons.

While we deem a more comprehensive description of the technical details eventually leading to the post-embedding iTEM procedure used in the current study as beyond its main scope, and while this also holds true for an extensive, all-encompassing explanation for how some of the additional spread for the IGPs for B at the NB's distal end can arise when using oocytes for iTEM, we nonetheless regard it important to point out already that such reasoning is based on geometric considerations. In essence, these take into account (i) that cross-sections through the NE are not always perfectly perpendicular, (ii) that such non-perpendicularity is not always immediately obvious, due to variability in the NE's staining for contrast (see further below), (iii) that the target epitopes are only accessible on the surface of sections that are 60-80 nm thick, (iv) that the *Xenopus* oocyte NE harbours an extremely high density of NPCs, often causing neighbouring NBs to be positioned closely next to each other, and finally, (v) that the only visible structure that can function as a reference point for measurements, namely the NE and its NPC, is positioned next to target site A but distant from the IGP-decorated epitopes of B at the NB's TR, the latter generally not unambiguously detectable as a structure in post-embedding iTEM. Altogether, these criteria allow for outlining scenarios, also involving trigonometric functions, that can plausibly explain why the spread of those IGPs that decorate sites further away from the NPC might to some extent not reflect physiological or architectural differences between the NBs.

Variability in the NE's contrast can arise when staining of a sectioned NE is not uniform all across the section depth of around 60-80 nm, but instead locally pronounced either towards the section surface or deeper within. The degree again by which such non-uniform contrast can manifest itself can depend on whether the specimens have been embedded in either a hydrophilic or hydrophobic Lowicryl resin and how these specimens have been treated with heavy metals for generating contrast. Having tested systematically different resins as well as freeze-substitution and immunolabelling parameters, in order to find a satisfying compromise between structure preservation, contrast, antigen preservation and accessibility, and other criteria, we eventually had to conclude that for our post-embedding iTEM project, only the hydrophilic Lowicryl resins K4M and K11M could be used, even though these came along with some disadvantages compared to the hydrophobic Lowicryl resins HM20 and HM23. The latter generally allowed for rather high contrast images of NEs that appeared more uniformly and durably stained with heavy metals, sometimes even accompanied by perhaps better, or just seemingly better, structure preservation. However, the degree by which such HM20 or HM23 sections could be specifically immunogold-labelled mainly had turned out to be insufficient and too low for any diagnostically conclusive evaluation, with this applying to most of the antibodies used in the current investigation. This was in striking contrast to the far better degree of specific immunolabelling achievable on the K4M or K11M sections when using the same antibodies. One of the downsides, though, that we had to accept for such more efficient immunolabelling was the abovementioned non-uniform heavy metal distribution that we often observed after having immunolabelled these hydrophilic resin-embedded NEs.

The reasons contributing to such lower-quality contrast appeared to be rather manifold: First, for all specimens, irrespective of whether they were later to be embedded in K4M, K11M, HM20, or HM23, *en bloc*-staining with OsO_4 had to be omitted in order to prevent antigen deterioration. Furthermore, we had found it necessary to omit water from the freeze-substitution procedure and also methanol from all substitution steps carried out at higher temperatures so that the only remaining *en bloc*-staining with heavy metal, i.e., via uranyl acetate (UA), might not have led to the same degree of UA deposition all throughout these specimens as it is commonly achievable by other protocols. This is so because UA is essentially not solvable at low temperatures in acetone and only poorly in methanol. However, since such limited UA *en bloc*-staining would have also affected those specimens to a similar extent that were later to be embedded in HM20 or HM23, we infer that the actual differences in heavy metal-staining occurred primarily during the later processes at the higher temperatures and in particular when the already resin-embedded specimens were incubated in aqueous solutions. In fact, we noted that the UA could be far more easily washed out again in varying amounts from the K4M-embedded specimens over time than from those embedded in HM20. Consequently, additional post-staining of specimens, routinely with UA and occasionally with lead citrate as well, often turned out contributing most to a K4M specimen's overall contrast. However, also such late heavy metal-staining often appeared, again for different reasons, somewhat unevenly distributed within the immunolabelled specimens once they were finally ready for microscopic inspection.

In addition to deviations from true NPC to TR distances that are a consequence of non-perpendicularly sectioned specimens and distance measurements between non-homogeneously heavy metal-stained NEs on the one side and IGP-decorated but otherwise not visible TRs on the other, we do not exclude the possibility that the widespread distribution of ZC3HC1 and TPR IGPs to some extent also reflects differences in conformations that the NBs might be able to adopt dynamically. In fact, conformational flexibility of the NB scaffold had been proposed earlier [e.g., 11], and if such former SEM-based findings do not reflect preparation and fixation artefacts, it is plausible that this flexibility would also contribute to the broader spread distribution of those IGPs that label the B sites in post-embedding iTEM.

Most of all, however, we regard it as likely, based on several lines of circumstantial evidence, that some static architectural features of the NBs are contributing most to the spread distribution of IGPs in post-embedding iTEM. In fact, in addition to the abovementioned target sites A and B, we consider it well possible that additional B-like sites exist, already in the early-stage oocyte, even further away from the NPC.

On the one hand, such broader distribution would then not only represent B sites at the TR of the NPC-anchored NB but also additional, more distally positioned TPR- and ZC3HC1-containing fibrillar material appended to the actual NB and projecting deeper into the nuclear interior, beyond the dimensions of the prototypic NB (see also Figure S7). Even though we had observed, by SEM, only minor amounts of fibrillar appendices at the NBs of those NEs that we had manually isolated from the late-stage II oocytes of slim frogs, we cannot exclude the possibility that such appendices might be more easily detached from the NEs of earlier-stage than later-stage oocytes, in the course of the manual NE isolation process.

On the other hand, yet not to be regarded as mutually exclusive, we consider it also well possible that the structural entity so far commonly regarded as the prototypic NPC-attached NB is actually a structure with a far more complex arrangement of TPR and ZC3HC1 polypeptides than formerly deemed likely. In particular, since it is already evident that the so-called TR actually bears little resemblance to a simple ring but instead rather looks like a rustic lattice fence, one could imagine that the different parts of the NB proper contain differently positioned pools of TPR and ZC3HC1 polypeptides and that these too might contribute to the IGP distribution spread.

1.7 Supplemental Information 7. Reflections on the amount relationships between ZC3HC1, TPR, and NPC scaffold proteins in different cell types.

After having evaluated all IB data of the *Xenopus* NE-containing materials, isolated several times from each of the different types of *Xenopus* cells, we had reached the conclusion that the NE-associated amounts of ZC3HC1 relative to those of TPR are generally rather similar in these different cell types, with clearly all these amount relationships being within the same order of magnitude. Furthermore, we had noted cell type-spanning similarities also applying to the ZC3HC1:NUP107 and the ZC3HC1:NUP96 ratios. However, upon more stringent comparison, there were also some differences recurrently notable. In fact, in comparison to the LNN materials of the XL-177 cells, regarded as a reference and yielding the most uniformly reproducible data, the amounts of ZC3HC1, and of TPR, relative to those of NUP107 and other NPC scaffold proteins, tended to be sometimes higher or lower in the NE materials of the other cell types. For example, a tendency for variably higher quantity ratios was evident, in particular, for the isolated NEs of oocytes obtained from heavyweight and often elder frogs. By contrast, in the LNN materials obtained from different batches of erythrocytes, the quantity ratios of ZC3HC1 and of TPR to NPC scaffold proteins tended to be lower than in XL-177. This also turned out to be the case for nuclei and their NEs that we had isolated from adult *Xenopus* brain. In these brain materials, too, the mean amounts of NE-appended ZC3HC1 and TPR, relative to those of the NPC scaffold proteins, tended to be slightly lower than in the LNN fractions of XL-177 (our unpublished data). Moreover, in these mixed populations of neuronal and glial cell nuclei, even the mean quantity ratio of NE-associated TPR to ZC3HC1 appeared to be slightly higher when compared with the other *Xenopus* cell types investigated, and this turned out to also be the case for brain and glial cell nuclei isolated from adult mice (see further below).

However, during our study, we had also gained the impression that some of the abovementioned data needed to be interpreted with caution for several reasons.

(1) Regarding the higher amounts of NE-attached TPR and ZC3HC1, relative to the NE-associated amounts of NPC scaffold proteins, in the late stages of oogenesis and particularly in oocytes of heavyweight frogs, one needed to keep in mind that such oocytes are stockpiling proteins at different storage sites within the oocyte for later use during embryogenesis [e.g., 12,13]. While many proteins of the NPC scaffold appear to be stored primarily in the cytoplasmic AL, we were convinced early on that TPR, and then also ZC3HC1, were among those proteins that are stockpiled within the nuclear interior instead, where these two proteins can be part of fibrous materials that occur appended to the NB proper and project further into the nuclear interior [e.g., 10; Figure S7, and our unpublished data]. When manually isolating and cleansing the NEs from such late-stage VI oocytes, we did not always find it possible to quantitatively remove all of this additional material, especially from the NEs of heavyweight frogs, in which amassments of NB-appended fibrous materials are particularly prominent. We deemed this a likely explanation for the varying amount ratios of NB to NPC proteins observed in different preparations of such oocyte NEs. By contrast, such problems were notably less pronounced when studying the protein composition of NEs obtained from the stage V oocytes of younger and slender frogs. Yet nonetheless, even with these latter NEs, we felt uncertain at some point as to whether the quantity ratios that we had observed could indeed be interpreted unambiguously for the following reason. On the one hand, mass spectrometric determination of the amount ratios of most of the oocyte's NE-associated proteins of the NPC proper, relative to the Y-complex proteins xlNUP107 and xlNUP96 as part of these NEs, had yielded values (our unpublished data) that turned out being very similar to those amount ratios accurately determined for the NPCs of HeLa cells [14], in which hsNUP107 and hsNUP96 occur in defined numbers within the human NPC's cytoplasmic ring (CR) and nuclear ring [NR; 14]. At first sight, all of these results appeared to clearly argue for rather similar copy numbers constituting the NPCs in these different cell types. On the other hand, however, a cryo-electron tomography (cryo-ET) study on

NPCs from the stage VI oocyte of *Xenopus laevis* had then reported conspicuous differences between the NR of a HeLa cell's NPC and the NR of the oocyte's NPC, with the authors mentioning that it had not been possible to dock the Y-complex into the NR of the *Xenopus* NPC in any consistent manner [15]. However, if it were true that architecture and thus the composition of the *Xenopus* oocyte's NPC would notably differ from that of other cell types' NPCs, with this then also applying to the Y-complex proteins, such differences in NUP107 copy numbers would have made our IB-based ratio comparisons between the NB proteins and NUP107 in different *Xenopus* cell types obsolete. Moreover, when later studies also demonstrated that the CR and NR of at least some species could indeed notably differ from each other when comparing their arrangements and their Y-complex copy numbers [e.g., 16], we regarded our concern as actually not far-fetched. Only most recently was this uncertainty regarding the *Xenopus* oocyte's NPC dispelled by another cryo-ET study, which demonstrated close structural similarity between the *Xenopus* oocyte's CR and NR, and also revealed conspicuous overall similarity between the NPCs from the oocyte and the human tumour cell [17].

(2) Regarding the lower amounts of NE-attached TPR and ZC3HC1, relative to the NE-associated amounts of NPC scaffold proteins in the nuclei and LNN materials of *Xenopus* erythrocytes and brain cells, several explanations were imaginable. (i) First, we could not exclude that a small amount of TPR and ZC3HC1 might simply have been lost in the course of the isolation procedure for erythrocytes, and especially in the course of isolating tissue nuclei; with such procedures being more time-consuming and laborious than the rapid manual isolation of an oocyte nucleus or the similarly straightforward preparation of the LNN fractions from cultured cells like XL-177. Such a potential loss might partly be due to certain differences in the post-translational modification status of ZC3HC1 in some cell types, which might cause easier detachment of ZC3HC1 from the NE during certain isolation procedures. (ii) Second, and in contrast to the proliferating XL-177 cells, in which most if not almost all NPCs appear to have an NB attached to them, with each finally assembled NB then possessing at least a similar minimal amount of TPR and ZC3HC1, we think that this does not necessarily need to hold true for every NPC of a blood or tissue cell nucleus. In these latter nuclei, we can instead also imagine some subpopulations of NPCs to have either less or far more TPR and ZC3HC1 appended to them than to other NPCs within the same nucleus. Furthermore, we do not exclude the possibility that in some distinct cell types all NPCs may have by default a smaller amount of TPR and ZC3HC1 appended to them, down to half the amount in other cell types. Moreover, in yet other types of terminally differentiated cells, some individual NPCs might even have no ZC3HC1 attached to them at all or lack both TPR and ZC3HC1 entirely. In fact, a wealth of former EM studies has been pointing at NPCs, for example, those of spermatocytes, that are seemingly devoid of so-called NPC-associated heterochromatin exclusion zones (HEZs), with such HEZs having been shown to mark the presence of an NB [18].

On the other hand, in many other types of heterochromatin-rich cells, some of their NPCs are known to be connected to HEZs that are particularly long, with such heterochromatin-flanked zones of a cylinder- or channel-like appearance projecting deep into the nuclear interior, beyond the dimensions of the NPC-attached NB [18, and references therein]. It is tempting to speculate that such HEZs are formed by arrangements of TPR and ZC3HC1, similar to those cylinder-like structures shown in Figure S7, and that the occurrence of such long cylinders at some NPCs, and their absence from others, correlates directly with the presence and absence of varying amounts of TPR and ZC3HC1. At least the situation in which the shorter versions of such HEZs are absent, as a result of eliminating TPR and the prototypic NB [18], and which we now know would come along with ZC3HC1 being absent too, would be equivalent to a subpopulation of nucleolus-abutting NPCs in the yeast *Saccharomyces cerevisiae*. There, such NPCs are known to be completely devoid of the budding yeast's homologs of TPR, namely Mlp1p

and Mlp2p [e.g., 19,20], and those NPCs also seem to lack exclusion zones of any kind [e.g., 21].

Currently, though, several of the scenarios relating to the vertebrates' tissue nuclei are just assumptions, also because occasionally noted variations in the intensities of staining for TPR and ZC3HC1 in IFM at the NEs of such nuclei, relative to the staining of their NPCs, might merely reflect incompleteness of labelling in these usually heterochromatin-rich cell nuclei, in which target accessibility for the bulky TPR and ZC3HC1 antibodies is likely to be limited. Because such IFM-based observations can therefore not be treated as evidence in support of the abovementioned notions, we feel that these issues, together with the range of questions relating to TPR and ZC3HC1 copy numbers and their arrangements in tissue nuclei, will need to be thoroughly addressed in future studies again, also once single-domain antibodies for TPR and ZC3HC1 will have become available. The latter should then allow for better targeting of these proteins in heterochromatin-rich environments.

Besides that, we had attempted to answer already one particular question in this broader context that we deemed especially important, namely whether the abovementioned somewhat lower mean amounts of NE-associated ZC3HC1 and TPR within the nuclei isolated from the adult brain might be the result of some gradual reduction with age. To address this issue, we had examined the mean amounts of TPR and ZC3HC1 relative to those of NUP107 and other proteins in populations of nuclei isolated in parallel from the cerebra of newborn and 1.5- and 2-year-old mice (our unpublished data). These source materials, in principle representing the same type of tissue, yet differing in age, had allowed for comparing (i) nuclei from cells that had just arisen from the highly proliferative cell divisions that occur during embryogenesis, with (ii) those nuclei of the adult cerebrum in which the majority of glial and neuronal cells had already been there in a non-proliferative state for up to year-long periods of time [e.g., 22–24]. This examination had resulted in finding ZC3HC1 similarly well detectable within the newborn and the adult mice's brain nuclei, and that its mean NE-associated amounts relative to those of NUP107 had remained very similar with advancing age. Moreover, the amount ratio of ZC3HC1:TPR too appeared to have remained rather similar, yet with some moderate increase in the NE-associated amounts of TPR relative to those of ZC3HC1 during ageing. In summary, these findings had allowed concluding that the, on average, somewhat lower overall amounts of NE-appended ZC3HC1 and TPR relative to those of NPC scaffold proteins like NUP107 did not necessarily reflect a general, age-related reduction.

Furthermore, we also regard it as noteworthy that an additional soluble pool of ZC3HC1 had been obtainable from the brain nuclei of young mice, where such ZC3HC1 might have either represented a subpopulation most easily detachable from the NE or perhaps some truly naturally-occurring soluble pool. In the adult brain nuclei, by contrast, such a soluble pool had not been similarly detectable. Conversely, hardly any TPR had been found existing in soluble form in the young nuclei, yet for this protein some soluble amounts had been detectable in adults. This finding and others of a similar kind eventually corroborated the idea that a certain surplus of one of these proteins cannot be appended to the NE when the other is not similarly available.

1.8. Supplemental Information 8. Destabilisation of ZC3HC1-NB interactions during cell fractionation.

In the past, a range of differently composed buffered solutions, in combination with other non-physiological conditions, have been commonly used for the enrichment and isolation of cell nuclei, and sometimes also for further enrichment of their NEs or NPCs. Some conditions, however, had already been known to result in the "*progressive differential dismantling of the pore complex*", while for others, it had been noted early on that "*the pore-attached material would require stabilisation by added bivalent cations*" [25, see also 26,27]. Later, once the NB was regarded as a structural entity, certain conditions were shown by either TEM or SEM to destabilise the entire NB or parts thereof indeed, while leaving the central parts of the NPC largely intact [e.g., 28,29].

Following up on such studies, we had first heuristically tested by series of trial-and-error experiments, and later in systematic detail, a range of procedures and conditions with regard to their effects on NB integrity in *Xenopus* and later also other amphibians' oocytes, using SEM for visualising the effects. Some of the procedures resulted in complete or partial NB disassembly, along with either complete, partial or no disintegration of the NPC proper. Accordingly, some procedures allowed for selective detachment of most or all of the NB's proteins, while others caused only one or other of them to be lost. Of these proteins, though, ZC3HC1 was the only one whose complete detachment commonly came along with at least one distinct subpopulation of TPR being detached as well. Conversely, we found that the same NB proteins remained durably NE-attached even during long-term treatments when applying NB-stabilising conditions, including also some, but not solely such, that more closely resemble those conditions the NE encounters in its physiological environment.

Later, we also systematically tested a wide range of conditions for subcellular fractionation and the isolation of the LNN-enriched materials of cultured amphibian and human cells. This eventually revealed that several basic principles underlying NB stability or destabilisation not only applied to the oocytes of *Xenopus laevis* and other non-hibernating amphibians but also to the proliferating cultured cells of human cell lines. Similar as for the manually isolated oocyte NEs, a combination of at least two non-physiological conditions would eventually cause ZC3HC1 to be rapidly and wholly detached from the cultured cells' LNN-enriched fractions. Among such NB-destabilising conditions were, e.g., the lack of adequate concentrations of divalent cations like Mg^{2+} within the one or other fractionation buffer and the standard mode of performing at least some of the fractionation steps at ice-cold temperatures. If not counterbalanced by other means, these combined conditions resulted not only in rapid detachment of essentially all ZC3HC1 but also of about half the total amount of NPC-associated TPR from the LNN-enriched fractions of different human cell types. However, somewhat different from the *Xenopus* oocyte NEs from the later stages of oogenesis, with their virtual absence of NE-associated chromatin, it was not possible, so far, to detach all TPR from the cultured cells' NEs without also causing some release of NPC components (our unpublished data).

Further experiments along this line then even indicated that ZC3HC1 occurs NB-associated in two distinct populations whose binding properties respond differently to changes of the physicochemical properties of the surrounding milieu. These findings further corroborated our impression that even rather subtle changes in buffer composition and other experimental conditions can result in the detachment of some or all of the ZC3HC1 polypeptides from the NE, together with distinct amounts of TPR.

In the course of these experiments, we had also noted that several experimental specifics that are characteristic for some of the common fractionation procedures used for vertebrate cells actually represent conditions that we had found causing ZC3HC1 to be detached from the NE. Therefore, we tested a range of published procedures used for isolating vertebrate nuclei in the past, among which were also protocols allowing for the enrichment of morphologically intact mammalian NPCs [e.g., 30]. One of the latter study's corresponding buffered solutions is actually referred to in the current study as an example

of an NB-destabilizing buffer which, in combination with the other destabilising condition of commonly performing the fractionation on ice, caused complete detachment of ZC3HC1 from the NPC- and nuclear lamina-enriched materials (Figure 3D). These findings might provide a possible explanation for why ZC3HC1 had escaped detection as an NB protein in the course of former mass spectrometry-based proteomics of NEs and purified mammalian NPCs that had been isolated via procedures that had destabilised ZC3HC1-TPR interactions. Since the corresponding ZC3HC1 ORF sequences, or sufficiently large error-free segments thereof, would have already been available [31,32] for peptide spectrum matches at that time, it seems that the corresponding proteins had indeed no longer been part of the materials then analysed by mass spectrometry.

While a detailed description of the different physicochemical parameters that can affect the NB's different components goes beyond the scope of the current study, we nonetheless deem it important to have exemplified how the stability of ZC3HC1's binding to the NB can be diversely affected in the course of different cell fractionation procedures (Figure 3D) and to have provided the corresponding experimental details in the main text's Material and Methods. In addition, the conditions having caused NB removal from the *Xenopus* oocyte NE materials presented in the Figure 1A3 and 1B1 are mentioned in the Supplemental Material and Methods section.

1.9. Supplemental Information 9. Reflections on co-immunoprecipitation of TPR and ZC3HC1 from the extracts of cultured cells and oocytes and from post-mitotic Xenopus egg extracts.

Rather than just precipitating small fragments of the NB or even larger parts of the NE from low-speed supernatants of ruptured cells in interphase, which would merely reflect what we had seen by iSEM anyhow and which we thus regarded not further informative, we wanted to make use of naturally-occurring soluble ZC3HC1 and TPR proteins, i.e., those that would be present in high-speed cell extract supernatants, and try to stimulate them to re-engage into interacting with each other. However, when having used the naturally-occurring soluble proteins of interphase cell extracts of cultured vertebrate cells, which generally contained either hardly any soluble TPR or soluble ZC3HC1, depending on the cell type (e.g., Figures S2 and S16, and further below), we had found the results not being sufficiently unambiguous, and the latter also pertained to some IP experiments conducted with the truly soluble proteins of oocyte nuclei. Some of our data had even led us to suspect that there might be some physiological reason for why small amounts of ZC3HC1 and TPR, if both would co-exist in a naturally soluble state in some specific cell type in interphase, might not quantitatively interact with each other and instead be barred from premature interactions. Furthermore, having initially also tried mitotic cell extracts, in which essentially all TPR and ZC3HC1 naturally occurs in a solubilised form, with not only TPR but also the native ZC3HC1 polypeptides then being phosphorylated at numerous sites [e.g., 33–35,36; our unpublished data], it had not come as a surprise that the mitotic versions of these proteins, in the presence of phosphatase inhibitors, could not be effectively co-immunoprecipitated either. Only later in our study did we realise that one could truly solubilise distinct subpopulations of NB-associated TPR polypeptides of cultured cells by different non-physiological means and then enable them to reassociate with soluble ZC3HC1 polypeptides released from the same cells, as will be shown in Figure S11.

By then, though, we had already been able to unambiguously co-immunoprecipitate TPR and ZC3HC1 from post-mitotic egg extracts (Figure 4A). While successful, this co-immunoprecipitation (co-IP) of TPR upon quantitative IP of ZC3HC1, and *vice versa*, was, however, not quantitative, and we can imagine this to have had several reasons. First, the assembly-competent egg extracts used for these IPs had been prepared from the eggs of heavyweight frogs, in which the ratios of soluble ZC3HC1 to soluble TPR sometimes appeared to differ between batches obtained from different frogs, and when compared to those ratios that apply to the cleansed oocyte NEs. Second, within such initially mitotic egg extracts, usually, not all TPR and ZC3HC1 polypeptides have already been sufficiently dephosphorylated again after having rendered the extract assembly-competent. In fact, the degree by which TPR and ZC3HC1 were still mitotically phosphorylated at distinct sites was found to vary between different preparations of assembly-competent egg extracts, and some of these mitotic phosphorylations impair TPR-ZC3HC1 interactions (our unpublished data). Third, TPR polypeptides appear to attract at least two pools of ZC3HC1, with one of these subpopulations being less tightly bound to TPR than the other, and some of these ZC3HC1 polypeptides can actually be seen gradually detaching again from the immunoprecipitated TPR (Figure 4A). However, while the characterisation of different populations of TPR-interacting ZC3HC1 polypeptides goes beyond the scope of the current study, we regard the data presented here as clearly demonstrating that ZC3HC1 and TPR can engage in specific physical interactions, even when still occurring as soluble proteins within a pool of thousands of others. These findings were later corroborated by further IP experiments using cell extracts of human HEK293T cells (see data within Figures S11 and S12).

1.10 Supplemental Information 10. Considerations regarding RNAi in cell lines of the allotetraploid organism Xenopus laevis.

In principle, RNAi-mediated knockdown (KD) of ZC3HC1 and other targets can be achieved in XL-177 cells, resulting in phenotypes similar to those upon RNAi of the corresponding target proteins in human cells (our unpublished data). However, it can be more difficult to efficiently silence certain target proteins in the cells of this particular frog species than in human cell lines. This also has to do with allotetraploidisation having occurred in *X. laevis* about 17-18 million years ago [37, see also 38], which in turn means that a large number of *X. laevis* proteins stem from two co-orthologous and still transcriptionally active genes, like it is the case for xlZC3HC1, while for others, like xlTPR, only the two alleles of a single-copy gene have survived the evolutionary processes since then. In contrast to xlTPR, a protein like xlZC3HC1 and several other NPC-associated proteins of interest to us are thus encoded by two distinct transcripts that exhibit notable sequence differences and both need to be destructed by RNAi. In these cases, one either has to find those few short nt segments, among the limited number of those whose sequences are identical in both transcripts, that can be efficiently targeted with only one double-stranded siRNA, or one has to identify two pairs of siRNAs that are both similarly well capable of conferring efficient elimination of their respective target transcripts, with both pairs not causing off-target effects. In addition to such complications, we also noted that it generally took more time in XL-177 cells to reach a degree of knockdown that was similar to that of the homologous target in human siRNA-transfected cells, in which such knockdown was more rapidly, often days faster, achievable.

1.11. Supplemental Information 11. A HeLa subline termed P2 and its use for CRISPR/Cas9n-editing.

For CRISPR/Cas9n-editing of HeLa cells, we chose a HeLa cell line of high passage number that we had isolated in the past when screening HeLa cell populations derived from HeLa CCL-2 for colonies exhibiting a range of desired properties. Such screens had aimed at (i) a higher degree of tolerance and robustness towards changes in cell culturing conditions and passaging procedures, including prolonged survival in acidified media as well as improved days-long transportability at RT under varying conditions with only atmospheric CO_2 available, (ii) a more narrow spectrum in chromosome number variations between the individual cells of a population and (iii) a more uniform cell morphology for cells within the same phase of the cell cycle, with particular emphasis on nuclear morphology; especially when comparing such subpopulations with the original HeLa CCL-2 cell line and several other established sublines derived from CCL-2. Concurrent control of transfectability with expression vectors and siRNAs, and of subsequent reporter gene expression and performance of the RNAi machinery, aimed at identifying those sublines in which such criteria and resulting performance were, at least, not notably impaired. In parallel, the newly obtained sublines continue to be regularly controlled not to have lost characteristic HeLa markers. Among these novel sublines was one that we termed "HeLa Pretty" or HeLa P (Cordes and Hase, unpublished), and a progeny thereof (HeLa P2; our unpublished data), which in the current study is only referred to as HeLa, and which is used for all CRISPR/Cas9n-editing of HeLa cells presented here. In its recent outsourced re-evaluation, including commissional karyotyping and microsatellite analyses (Dr U.A. Mau-Holzmann, University of Tübingen, Faculty of Medicine, Division of Cytogenetics), HeLa P2 was confirmed as unambiguously representing progeny of the original HeLa line. Of particular note, while the karyotype of HeLa P2 differed notably from the ones reported for several other HeLa sublines [e.g., 39–41], it was found to share close similarities with yet other HeLa strains, regarding their modal chromosome numbers and their range of chromosome numbers [42], and this held especially true for a high-passage-number strain of the HeLa subline Kyoto (HeLa K; RRID:CVCL_1922), which had been extensively analysed in the past [43]. Since HeLa P and HeLa P2 on the one hand and HeLa K on the other are not each other's progenitor or progeny, it is noteworthy that independent screening projects had resulted in the isolation of HeLa sublines that are similarly "user-friendly" and share a very similar karyotype which, though unbalanced, exhibits stability over many generations. On the other hand, some distinct chromosomal structural aberrations also allow for distinguishing HeLa P from HeLa K; yet none of the affected chromosome segments carries genes currently of interest to us, including, among others, *TPR* at 1q31.1, *ZC3HC1* at 7q32.2., and *NUP107* at 12q15. The same holds true for another HeLa strain, HeLa-EM2 [44], which was used for some of the experiments presented in Figure S14, where it is referred to as HeLa W. This subline too has been confirmed by karyotyping and microsatellite analyses to unambiguously be a progeny of the original HeLa line (our unpublished data).

While these different HeLa strains, apart from their many similarities, which include similar NB to NPC protein copy number relationships, also exhibit some differences concerning morphology and physiology, we regard these in the context of the current study as not relevant. A direct comparison of some of these HeLa sublines and some of their characteristics are to be presented elsewhere.

2. Supplemental Figures

A

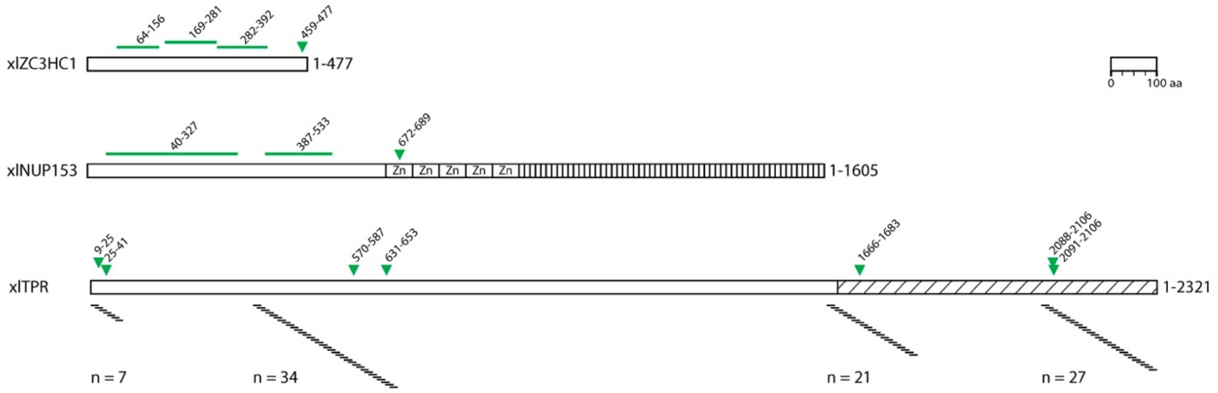

B

Examples of antibodies specific for the target region	TPR fragments								
	aa 1-383	aa 354-666	aa 650-986	aa 921-1195	aa 1176-1465	aa 1434-1632	aa 1595-1855	aa 1817-2062	aa 2024-2321
xlTPR (9-25)	**100**	5	0	0	6	0	0	0	0
xlTPR (25-41)	**100**	0	0	0	2	0	0	0	2
xlTPR (570-587)	1	**100**	0	1	1	1	0	0	4
xlTPR (1666-1683)	0	0	0	0	4	1	**100**	0	0
xlTPR (2088-2106)	0	0	0	0	1	0	0	0	**100**

Examples of antibodies with intramolecular cross-reactions	TPR fragments								
	aa 1-383	aa 354-666	aa 650-986	aa 921-1195	aa 1176-1465	aa 1434-1632	aa 1595-1855	aa 1817-2062	aa 2024-2321
xlTPR (1-17)	**71**	0	0	16	100	36	0	0	77
xlTPR (462-479)	0	**27**	2	0	25	100	0	0	1
xlTPR (2304-2321)	6	1	0	0	100	29	0	0	**91**

C

```
xlZC3HC1
Isoform 1     MATTCEEVLVSPVKSTAVTPLKIRELINEGIVCGERSGIGRKEASETPEENTAFEDLPGNNSYESTSKDAFFSRVESFSSLKWAGKPPEL   90
Isoform 2     MAAPCEDVLVSPVKSPAVTPLKIRELINEGIVSGERSGNGRKETAEIPEENNGFEDPPGNSSFESTTKDAFFSRVESFSSLKWAGKPPEL   90
              **: **:******** ****************.***** ****::* ****..*** ***.*:***:***********************

              CPLICAKYGWCNIECDMLKCSSCNAYLCASLQPILDFSKYKQRCVELQEALRKAHEKFCFWPDSPCPEHFWALLVTEPSSVLSDFVERFH   180
              CPLICAKYGWCNIECDMLKCSSCNAYLCASLQSVLDFSKYKQRCVELQEALRKAHEKFCFWPDSPCPDNFWALLVTEPSSILSDFVGRFH   180
              ******************************** :*********************************::***********:***** ***

              NLCHLEMQLPSLKHEDVKSMDITEDTVSRLLRLIEDELKPKEGREANSHSLPSDSLQVHISACILALCGWNTSFSSGSLCIINCPRCMRK   270
              NLCHLEMQLPSLKHEDLKSMDITEETVSRLLRLIEDELKPKEGKEDNSRSLATDSLQIHISACILALCGWNTSFSAGSLCIINCPRCMRK   270
              ****************:*******:******************:* **:** :****:*****************:**************

              VGLWAFQQLETVELDNSLSAPGTPVSPAEGHERSPLGFMSPSRRVTRSRDAEQSPAFAYGRTRSSDLLSPADSEAVRNRPVTRSMGQGEN   360
              VGLWAFQQLEAVELDNSLSAPGTPVSPGEGHERSPFGIMSPNRRVTRSRDTEQSPAFAYGRMRSSDLLSPADSEAIRNRPVTRSMGQGEN   360
              **********:****************.*******:*:***.********:********** *************:**************

              TGLGNELHSSPHRRAKRPRLCSSSSSDTSPRSCFDPLSQHRSWCPWVNVCQASGTNTMGSEIQEEARRKEYGWKEVLKVLLAEENSRTIS   450
              TGLGNELHSSPQRRAKRPRLCSSSSSDTFPRGCFDPLSQHRRWCPWVNVCKASGTSTVGMEIQEEASRKAYGWKEVLKVLLSEEE----S   450
              ***********:**************** **.********* ********:****.*:* ****** ** ***********:**:    *

              NPDTSSVPEKSHKVFRIFRQWQLAASS                                                                 477
              DPDISSVPEKSHKVFRIFRQWQMAATS                                                                 473
              :** ******************:**:*
```

S01 (1/5)

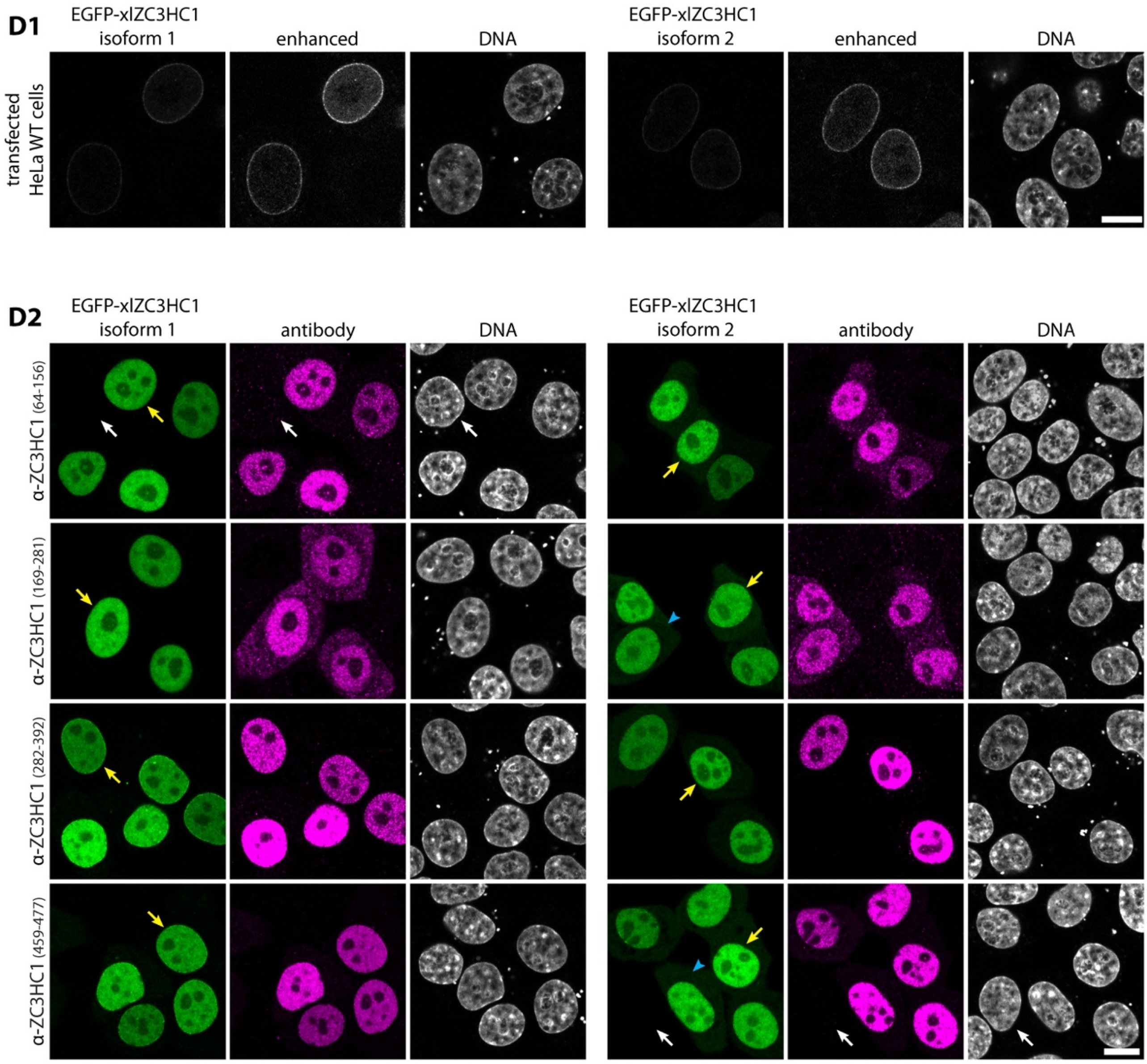
D1
EGFP-xlZC3HC1 isoform 1
enhanced
DNA
EGFP-xlZC3HC1 isoform 2
enhanced
DNA
transfected HeLa WT cells
D2
EGFP-xlZC3HC1 isoform 1
antibody
DNA
EGFP-xlZC3HC1 isoform 2
antibody
DNA
α-ZC3HC1 (64-156)
α-ZC3HC1 (169-281)
α-ZC3HC1 (282-392)
α-ZC3HC1 (459-477)

S01 (2/5)

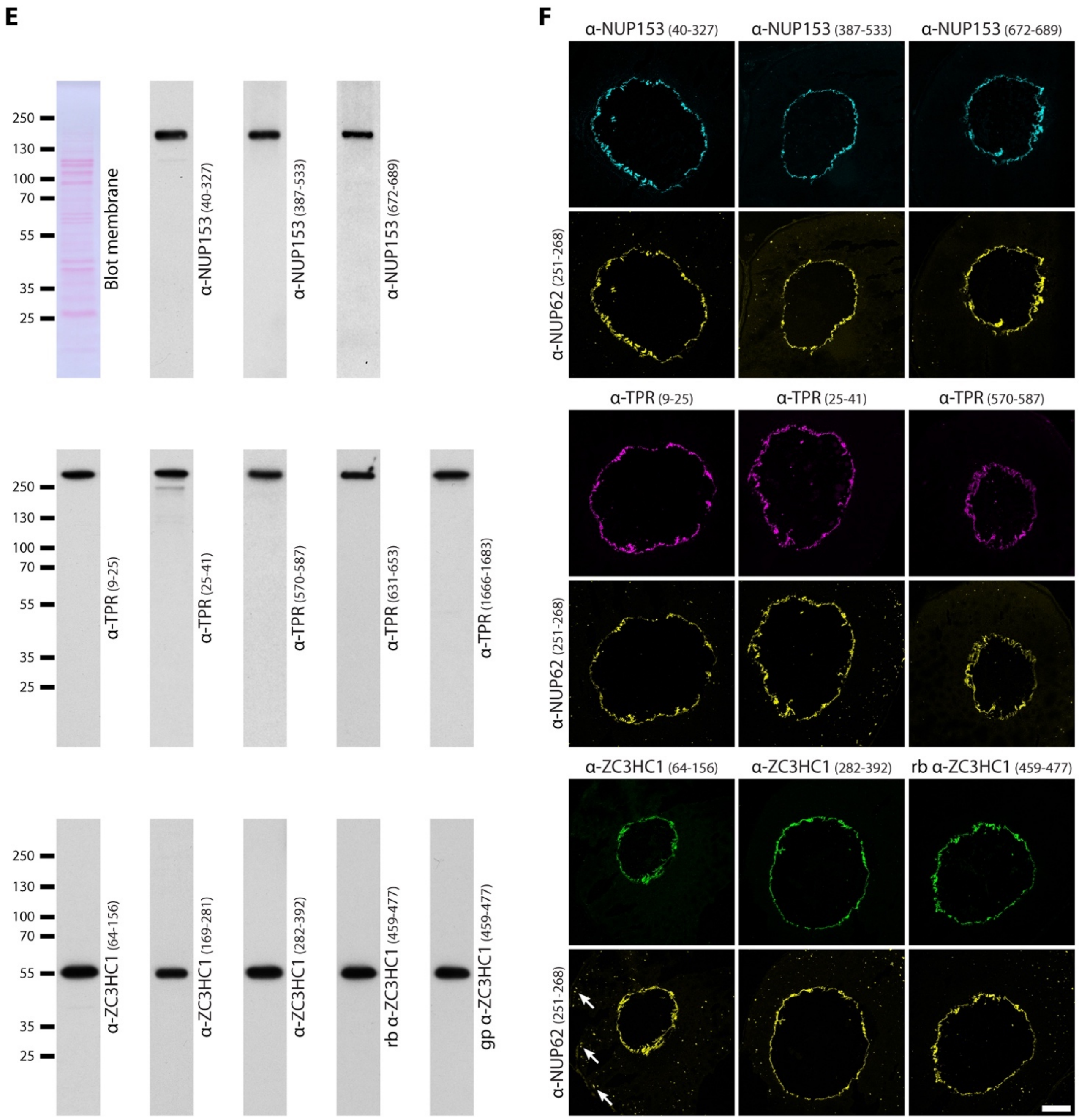
E
F
250
130
100
70
55
35
25
Blot membrane
α-NUP153 (40-327)
α-NUP153 (387-533)
α-NUP153 (672-689)
α-TPR (9-25)
α-TPR (25-41)
α-TPR (570-587)
α-TPR (631-653)
α-TPR (1666-1683)
α-ZC3HC1 (64-156)
α-ZC3HC1 (169-281)
α-ZC3HC1 (282-392)
rb α-ZC3HC1 (459-477)
gp α-ZC3HC1 (459-477)
α-NUP62 (251-268)

S01 (3/5)

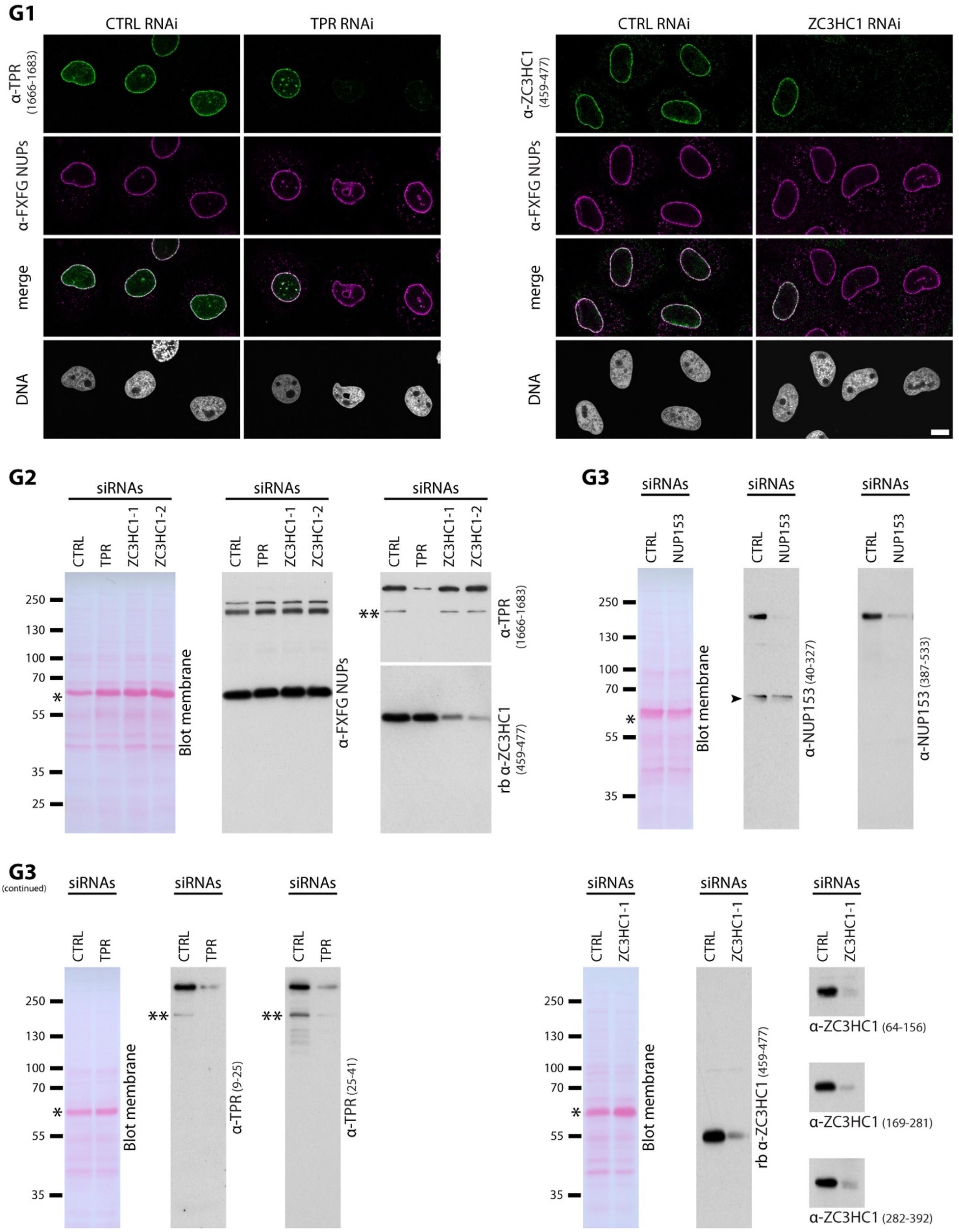
G1
CTRL RNAi
TPR RNAi
CTRL RNAi
ZC3HC1 RNAi
α-TPR (1666-1683)
α-ZC3HC1 (459-477)
α-FXFG NUPs
merge
DNA
G2
siRNAs
CTRL
TPR
ZC3HC1-1
ZC3HC1-2
Blot membrane
α-FXFG NUPs
α-TPR (1666-1683)
rb α-ZC3HC1 (459-477)
G3
NUP153
α-NUP153 (40-327)
α-NUP153 (387-533)
G3 (continued)
α-TPR (9-25)
α-TPR (25-41)
rb α-ZC3HC1 (459-477)
α-ZC3HC1 (64-156)
α-ZC3HC1 (169-281)
α-ZC3HC1 (282-392)

S01 (4/5)

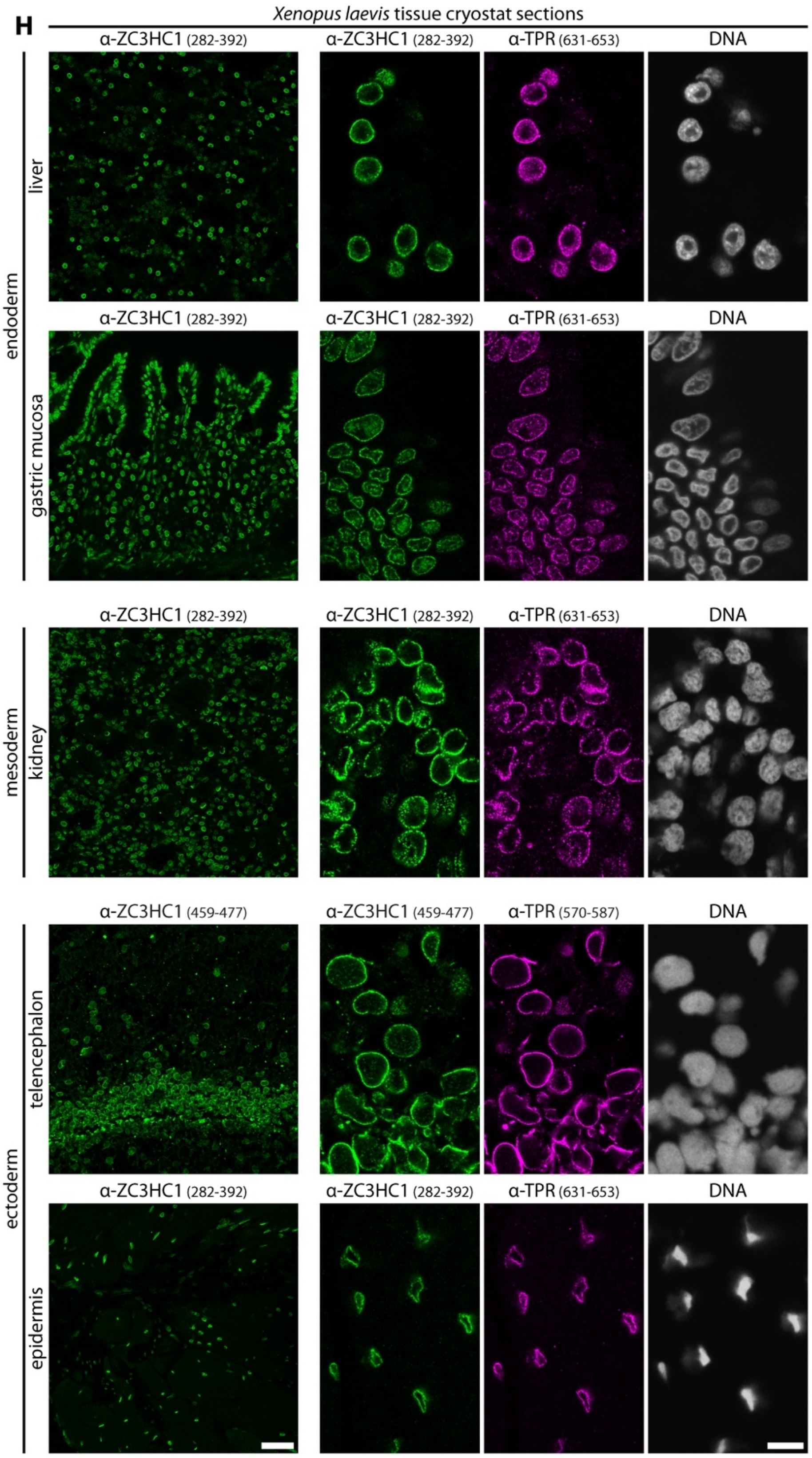

S01 (5/5)

Supplemental Figure S1. Characterisation of antibodies for *Xenopus* proteins ZC3HC1, TPR, and NUP153.

(A) Schematic depiction of each of the two orthologous proteins for ZC3HC1 and NUP153 in *Xenopus laevis*, an allotetraploid organism [e.g., 37,38], and of protein TPR, being the only remaining TPR orthologue in this frog. Merely the segments comprising the zinc fingers (Zn) and carboxy-terminal FXFG-repeat domain (dashed vertically) of xlNUP153, and the carboxy-terminal domain of TPR, the latter also predicted to be natively unfolded (dashed diagonally), are

highlighted. Regions of each protein for which those antibodies used in the current study had been purified, either via bacterially expressed recombinant polypeptides or with short, chemically synthesised peptides, are marked by green horizontal bars or arrowheads, respectively, with the corresponding ranges of amino acids specified. The ladders of short black bars beneath the scheme for TPR symbolise overlapping TPR peptides with a sequence overlap of 9 aa. These series of chemically synthesised peptides had been bead-immobilised and then used for the isolation of antibody subpopulations from sera that had been obtained after immunisations with recombinant polypeptides comprising larger parts of xlTPR. This procedure had been necessary because polyclonal populations of antibodies raised against several of the larger segments of TPR tended to include antibodies capable of intramolecular cross-reactions with other parts of TPR not used as antigens. Some of these antibodies had revealed such cross-reactions in IFM of different types of ectopically expressed TPR fragments within cultured cells, in conventional IB or dot-blotting of such TPR fragments, and eventually also in immuno-EM. In the current study, we hence only employed subpopulations of xlTPR antibodies with no conspicuous intramolecular cross-reactions, which we, therefore, regarded as specific for a single target site.

(B) Selection of representative results of ELISA experiments performed with a collection of rabbit peptide antibodies that had been isolated by the procedure described in S1A. The recombinant xlTPR polypeptides initially used for immunisations had been immobilised as targets. In the simple compilation of examples shown here, the highest interaction value for any of the different TPR fragments, obtained after side-by-side interaction with a given antibody, has been set to 100. Results representing a target-region-specific interaction are highlighted in shades of green, while those reflecting intramolecular cross-reactions are coloured in shades of magenta. Note that the selection of xlTPR peptide antibodies used for the current study (lettering in green) specifically reacted only with those parts of TPR that included their actual target epitopes. By contrast, other xlTPR peptide antibodies, represented by the examples shown here (lettering in magenta), exhibited pronounced cross-reactions with off-target parts of TPR. While many of these and other TPR antibodies exhibiting such intramolecular cross-reactions were nonetheless specific for protein TPR as a whole and, in principle, useful for conventional IFM and IB, they were not suitable for immuno-EM studies that aimed at determining the location of distinct parts of TPR at higher resolution. Note that some of the intramolecular cross-reactions did not appear explainable by a high degree of sequence similarity. In addition, the collections of antibodies isolated from different animals, even though having been immunised in parallel with the same recombinant polypeptide, differed to some extent with regard to some of the off-target sites recognised by their cross-reactive TPR antibodies (our unpublished data). In sum, irrespective of whether immunisations had been conducted in-house or at external facilities, intramolecular cross-reactions of antibodies raised against certain parts of TPR in different species were a common problem that needed to be solved prior to immuno-EM.

(C) Sequence alignment by Clustal Omega [45] of the two ZC3HC1 co-orthologues of *Xenopus laevis*. Sequence segments corresponding to recombinant polypeptides and a chemically synthesised C-terminal peptide used to purify antibody subpopulations are shaded in green. Several animals had been first immunised with the 477 aa-long xlZC3HC1 isoform 1 that we had expressed in and purified from insect cells, thereby having used the baculovirus expression system which was followed by boosts with aa 64-391 of isoform 1 that we had expressed in and purified from *E. coli*. Other animals were separately immunised with the C-terminal peptide that later was also used for peptide-antibody purification. Isolation of antibody subpopulations from the sera obtained after the immunisation with the recombinant polypeptides was then on the three bacterially expressed shorter segments of isoform 1 shown schematically depicted in S1A. Further note that the two isoforms of xlZC3HC1 only share 88% sequence identity and 92% sequence similarity. A range of reasons for why we nonetheless focussed primarily on raising antibodies against the isoform 1 can be summarised as follows. First, at the time point of our first identification of xlZC3HC1 by mass spectrometry (June 2007), as one of the proteins released from the NEs of *Xenopus laevis* oocytes upon NB disassembly, together with other NB proteins like, for example, xlTPR and xlMAD1, the only existing xlZC3HC1 sequence deposited in public databases as either refseq_rna nucleotide or nr_protein sequence was the one for xlZC3HC1 isoform 1. Aware of the fact that a protein in this allotetraploid frog might exist as either one or two co-orthologues, we had then inspected the *Xenopus laevis* ESTs available at that time, revealing a total of 28 EST sequences for xlZC3HC1 (August 2007), of which only three, representing two short non-overlapping cDNA segments, could not be clearly assigned to isoform 1 and which only later turned out to represent a second isoform indeed. These three EST sequences had not been taken into further account when raising the first collection of several xlZC3HC1 peptide antibodies in guinea pigs. The resulting antibodies, among which were some that should have cross-reacted with the potential second isoform, had then allowed for a seemingly specific immuno-localisation of xlZC3HC1 at distinct sites of the NB in *Xenopus* oocytes (our unpublished data). However, this first generation of xlZC3HC1 antibodies, raised in certain inbred strains of guinea pigs, had also led to some seemingly mutually exclusive findings in immuno-EM, in parts appearing incompatible with other data, and these findings had prompted us to adopt a range of measures. Among these were (i) the further development of the novel immuno-SEM and immuno-TEM procedures that are presented in the current study (see also comments in Figures S3 and S4), (ii) the preparation of new generations of xlZC3HC1 antibodies also in other species, including the rabbit antibodies presented here, (iii) RT-PCR using RNAs from stage VI oocytes as templates and subsequent cDNA synthesis not only of xlZC3HC1 but also of other NB proteins, in order to assemble their second isoform sequences, (iv) a closer analysis of the expression levels of xlZC3HC1 isoform 2 in *Xenopus* oocytes, thereby also taking novel EST sequence entries into public databases into account, (v) the assessment of the second isoform's

occurrence in also further isolations of *Xenopus* oocyte NEs analysed by mass spectrometry, and (vi) the quality-control of those antibodies raised against *X. laevis* proteins also by RNAi from then on. In sum, this allowed for confirming that ZC3HC1 isoform 2 is a protein that in *X. laevis* oocytes occurs in notably lower amounts than isoform 1, also in line with the abovementioned three ESTs for xlZC3HC1 isoform 2 still being the only database-deposited ones at later time points, compared to 61 ESTs for isoform 1 that had been deposited there by then (December 2012). At the NE of the *X. laevis* oocyte, we calculated the relative amounts of isoform 2 being rather low as well, ranging between 1% to 17% of the total amount of all xlZC3HC1 polypeptides at NB-containing NE preparations prepared for mass spectrometry and isolated from frogs differing in age and feeding status (n = 11). While isoform 2 is possibly present in slightly higher amounts in the eggs of *X. laevis*, where it was found to reach about one fifth of the cell's total amount of ZC3HC1 [46], we cannot tell so far whether it might occur in slightly higher relative amounts in the oocyte's soluble nuclear pool of xlZC3HC1 (see further below) and whether it might perhaps be less competitive than isoform 1 for binding to sites at the NE. This scenario could also hold true for some other NPC- and NB-associated proteins that occur as two co-orthologues in *X. laevis*. Clearly, however, both isoform 1 and 2 possess a prototypic signature characteristic for ZC3HC1 homologues all across the eukaryotic realm, as we will illustrate in a separate study. Furthermore, RNAi experiments performed in XL-177 cells, using siRNAs targeting only the one or the other of the two isoforms, or both together, as well as controls based on ectopic expression of isoform 2 polypeptides in human cells revealed that the populations of polyclonal xlZC3HC1 antibodies used for the immuno-EM data presented in the current study included such that recognised both xlZC3HC1 isoforms, as shown in the following.

(D) Testing of antibodies raised against xlZC3HC1 isoform 1 for cross-reactivity with xlZC3HC1 isoform 2.

(D1) Transient ectopic expression of EGFP-tagged xlZC3HC1 isoforms 1 and 2 in HeLa WT cells. cDNAs had been isolated from cDNA libraries generated by RT-PCR using RNAs from stage VI *Xenopus laevis* oocytes. Note that in cells expressing low amounts of these *Xenopus* proteins, with corresponding low signal yields also shown electronically enhanced for better visualisation, both isoforms were found located at the NE of the human cells. This indicates that not only isoform 1 but also isoform 2 is capable of binding to a vertebrate cell's NE and that both *Xenopus* isoforms can compete with their human counterpart for binding sites at the human interaction partner of ZC3HC1, despite only 53% sequence identity between xlZC3HC1 isoform 1 and hsZC3HC1. Bar, 10 μm.

(D2) IFM of HeLa cell populations including cells with and without ectopically expressed xlZC3HC1 isoform 1 and isoform 2. IFM was conducted with each of the four different rabbit polyclonal xlZC3HC1 antibodies used in the current investigation. Note that upon overexpression of both isoforms, resulting in these no longer bound only to the NE but mainly distributed across the nuclear interior instead (some examples marked by yellow arrows), all of the isoform 1 antibodies also targeted isoform 2. In addition, none of the xlZC3HC1 antibodies caused any conspicuous cross-reaction in those HeLa cells that had not been transfected with the xlZC3HC1 expression vectors (some examples marked by white arrows). As an aside, note that when comparing similar levels of expression, EGFP-ZC3HC1 isoform 2 generally exhibited a slightly more pronounced cytoplasmic localisation (some examples marked by blue arrowheads) than isoform 1, despite also possessing a seemingly intact NLS sequence (aa 372-381). The underlying cause for such subtle differences in the subcellular location of isoform 1 and 2 in human cells was not investigated in further detail. Bar, 10 μm.

(E) IB of manually isolated nuclei from *Xenopus* stage VI oocytes with antibodies for xlNUP153, xlTPR and xlZC3HC1 that were used in the current study for data with *Xenopus* material presented in the main Figures 1-4 as well as in the corresponding Supplemental Figures. Target regions are given in parentheses. Each lane was loaded with the total amount of proteins from the same number of nuclei. Immunolabelling was performed on the representative Ponceau S-stained membrane shown here and on replicates of the identical kind.

(F) Double-labelling IFM of cryostat sections of *Xenopus* stage VI oocytes with those novel antibodies for either xlNUP153, xlTPR or xlZC3HC1 that were used in the current study. In addition, the monoclonal antibody A225, specific for xlNUP62 [47], was used for reference staining of the NEs and ALPCs (some marked by arrows). Note that one of the xlZC3HC1 antibodies used for immuno-TEM (Figure 2) is not included here but presented in Figure S5B instead, where this antibody is shown to specifically label the NE on ultrathin sections of resin-embedded *Xenopus laevis* oocytes while being almost negative on corresponding *Xenopus* oocyte cryostat sections. As we had already noted it applying to some other antibodies too, this indicated that the epitopes targeted by some NPC and NB antibodies are hardly accessible in specimens for IFM when such epitopes are part of mostly intact three-dimensional structures, most of which are furthermore located deeper within the cryostat sections of several micrometres in thickness, and that such targets only become accessible and visible beyond background staining after having been exposed on the surface of ultrathin sections. Bar, 100 μm, same magnification for all micrographs.

(G) RNAi experiments in cultured *Xenopus laevis* cells of line XL-177, analysed by IFM and IB, in order to control for target specificity of antibodies, i.e., via the correlation between target protein knockdown (KD) and corresponding effects on immunolabelling in IB and IFM. Note that RNAi experiments of such kind in XL-177 cells were conducted at several time points of our study, with those performed at the beginning primarily based on available EST sequences at that time, while those repeated later made use of the then available sequence information for the full-length transcripts of both isoforms

for xlZC3HC1 and xlNUP153. Representative IFM and IB data shown here stem from cell populations that had been transfected with siRNAs for the transcript of the single-copy gene for xlTPR, or with siRNAs targeting both transcripts of the two co-orthologous genes for ZC3HC1 or those of the also co-orthologous NUP153 genes in *Xenopus laevis*. Transfections shown here had been performed with Lipofectamine RNAiMAX, and cells had been harvested 6 days post-transfection. Additional experiments performed with siRNAs specifically targeting only one of each pair of co-orthologous genes did not allow for visualisation of a sufficiently clear knockdown (data not shown), suggesting that their expression levels relative to each other might differ in XL-177 cells as compared to the oocyte, or that knockdown of the one isoform might induce upregulation of the other. Moreover, the data obtained with such allele-specific siRNAs also indicated that the antibodies raised against xlZC3HC1 isoform 1 targeted the other isoform, too (data not shown).

(G1) Selection of IFM micrographs of XL-177 cells that had been transfected with either non-target control siRNAs (CTRL) or siRNAs for TPR or ZC3HC1. Cells were double-labelled with representative antibodies for either xlTPR or xlZC3HC1, together with the monoclonal antibody mAb414, which binds to several FXFG nucleoporins located at the NPC, here shown for comparison. Images of the TPR and ZC3HC1 RNAi experiments were chosen to each include one representative cell as a reference that had remained non-transfected. Focal planes were aimed at being approximately perpendicular to the plane of the cells' NEs. Note that immunostaining of the NEs with the respective TPR and ZC3HC1 antibodies was notably reduced only upon RNAi-mediated KD of the corresponding target proteins, attesting antibody specificity. Similar results were obtained with the other IFM-suitable antibodies used in this study. Bar, 10 μm.

(G2) IB of total cell extracts from XL-177 cells that had been transfected either with non-target control siRNAs, with an siRNA for TPR, or separately with two different siRNAs that both target both isoforms of ZC3HC1. The Ponceau S-stained membrane shown here had first been incubated with mAb414, then recovered by quantitatively detaching the antibody through incubation at low pH, upon which it was cut in half and re-incubated with the indicated rabbit antibodies for xlZC3HC1 and xlTPR. Note that in former IB experiments using *Xenopus* oocyte extracts, mAb414 had been found to primarily react with xlNUP62 and xlNUP153, next to other FXFG nucleoporins [e.g., 29], with the third immuno-positive band seen here likely representing xlNUP214. The asterisk marks a prominent BSA band as a component of residual amounts of culture medium not wholly removed by washes, as hot protein sample buffer had been applied directly to the culture dishes in these cases. While of molecular mass similar to xlNUP62, this BSA band was confirmed not contributing to the prominent immunolabelling of xlNUP62 with mAb414. Note that specificity of TPR and ZC3HC1 antibodies was attested by evident knockdown of TPR and ZC3HC1, which was achieved without eliciting an apparent immediate effect on the cellular amounts of the here labelled FXFG nucleoporins.

(G3) IB of the total cell extracts of XL-177 like in S1G2, yet here only as pairs of lanes loaded with the extracts of cells that had been transfected with non-target control siRNAs next to the extracts of cells that had been treated with siRNAs for either NUP153, TPR or ZC3HC1. Immunolabelling was performed on the representative Ponceau S-stained membranes shown here and on replicates of the identical kind. For ZC3HC1 RNAi as well as for NUP153 RNAi, a single siRNA duplex each had been used for targeting the transcripts of both isoforms. The asterisk marks a prominent BSA band, described in S1G2. Membranes were incubated with those novel antibodies against xlZC3HC1, xlTPR and xlNUP153 that were used in the current study for immuno-EM and have not been used already for S1G2 or characterised and published earlier. The double asterisk marks a TPR degradation band, detectable in IB of XL-177 cell extracts with a wide range but not all of the xlTPR antibodies, with its intensity varying between preparations and even between immunoblots repeated with the same antibody. In contrast to antibody xlTPR (25-41), detection of this band with antibody xlTPR (9-25) required more prolonged exposure. The arrowhead marks a cross-reaction with a protein regarded unrelated to NUP153 within the total XL-177 cell extract and not detectable as a component of the total protein content of the *Xenopus* oocyte nuclei.

(H) IFM of cryostat sections of *X. laevis* liver, stomach, kidney, brain, and skin, representing tissues originating from the three germ layers. Overviews (left side) only show labelling with antibodies for xlZC3HC1, while the micrographs at higher magnification show such specimens also double-labelled together with antibodies for xlTPR. This then revealed that xlZC3HC1 and xlTPR colocalize at the NEs of these tissues' cells too. Bars, 50 μm (overview) and 10 μm, respectively. Furthermore, such NE-specific labelling not only showed that several of the rabbit and guinea pig antibodies raised against xlZC3HC1 and xlTPR were apparently target-specific in other types of *Xenopus* cells too, apart from oocytes and XL-177, but also that both proteins did not appear to have some other preferential subcellular location within different tissue cells.

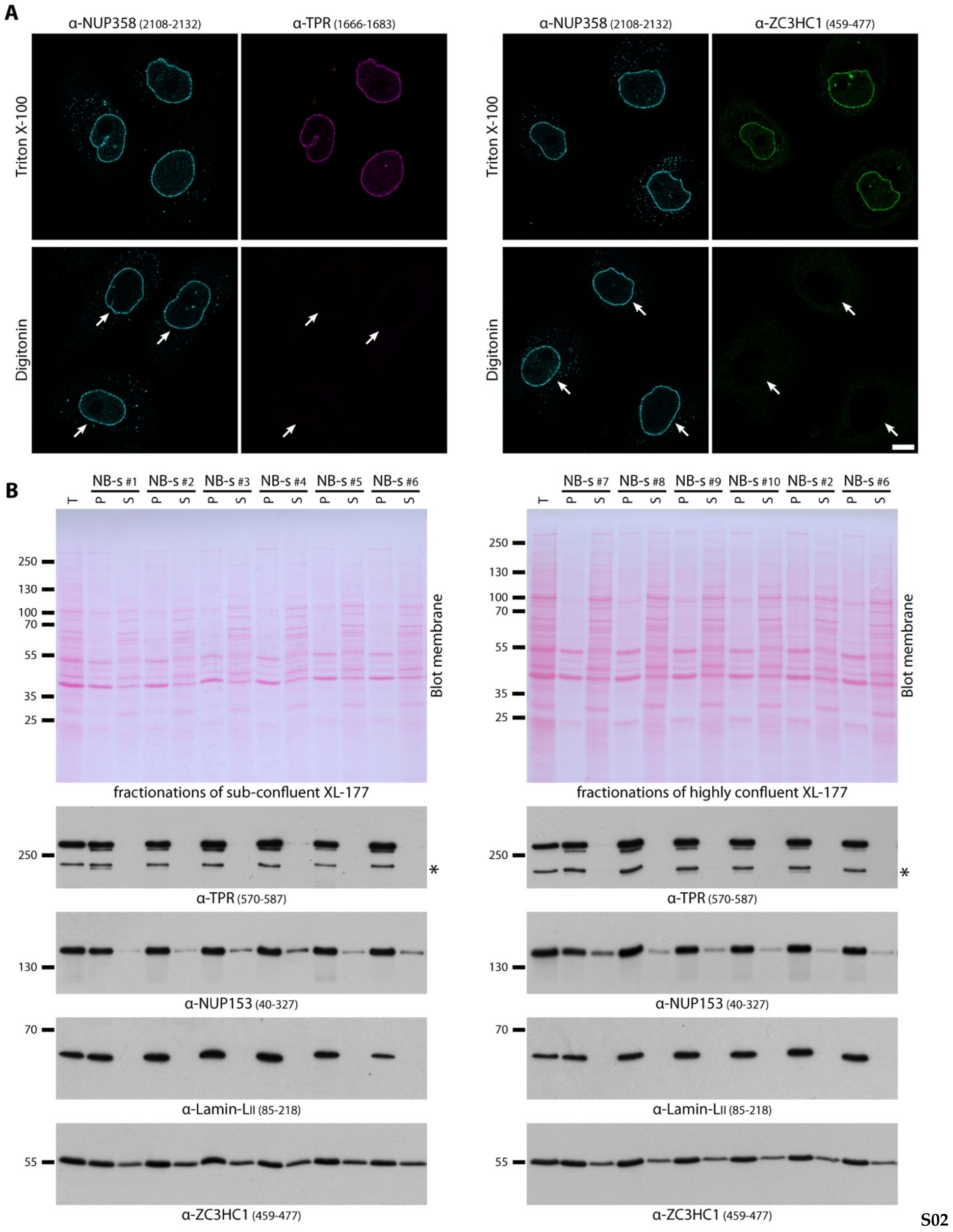

Supplemental Figure S2. Subcellular location of xlZC3HC1 in XL-177 cells.

(A) Accessibility of ZC3HC1 for antibodies in detergent-permeabilised XL-177 cells, as visualised by double-labelling IFM following permeabilisation with either TX-100 or digitonin. Specimens were labelled with antibodies for xlNUP358, a

nucleoporin appended to the NPC's cytoplasmic side, together with antibodies for either xlTPR or xlZC3HC1. Focal planes were aimed at being approximately perpendicular to the plane of the cells' NEs. Note that NE-staining for ZC3HC1 and TPR was visible only when the nuclear interior had been made accessible by NE permeabilisation with TX-100, while plasma membrane perforation with digitonin solely allowed for staining of NUP358. Arrows pointing rightwards or leftwards, respectively, mark corresponding cells in the two double-labelled specimens. Bar, 10 µm.

(B) IB of cell extracts obtained from sub-confluent and very densely packed confluent populations of XL-177 cells. Such cell populations had been split into suspension aliquots of similar cell numbers, further processed in parallel. Lanes were loaded with the non-fractioned cells' total cell proteins (T), the proteins in the solution obtained after treatment with TX-100 in different types of NB integrity-stabilising buffers (NB-s) and subsequent centrifugation at 20,000 g (S), and the non-soluble, lamina-NPC-NB (LNN)-enriched pellet fractions (P). Note that NB-s buffers #8 and #9 correspond to those used for LNN fractions shown in the Figure 3C and 3D, respectively, whose composition and the corresponding fractionation protocol is provided in the Material and Methods. For each one of the two cell populations, each of the loadings represented the corresponding fraction of the same number of cells, while cell numbers used for such fractionation differed between the sub-confluent and confluent populations, as did the exposure times of the two populations' corresponding sets of immunoblots. Immunolabelling with mAb X223, an antibody targeting the somatic *Xenopus* B-type lamin LII [48], and with antibodies raised against xlTPR, xlNUP153, and xlZC3HC1, was performed on the upper and lower parts of the membranes here shown stained with Ponceau S. This included re-incubations of these membranes with mAb X233 after quantitative removal of previously bound antibodies by incubations at low pH. The asterisk marks a TPR degradation band detectable in IB of XL-177 cell extracts with a wide range of TPR antibodies. Note that a soluble pool of ZC3HC1 was detectable both within the still proliferating XL-177 populations and those that had reached densely packed confluency, which in this adherent cell type comes along with a markedly decreased growth rate. Since hardly any soluble TPR and no soluble B-type lamin were detectable within the proliferating XL-177 cells, the largest proportion of soluble ZC3HC1 was regarded as non-mitotic and representing a natural pool of soluble ZC3HC1 in interphase. Even so, most of ZC3HC1 from such proliferating XL-177 cells was found to belong to the LNN-enriched fraction.

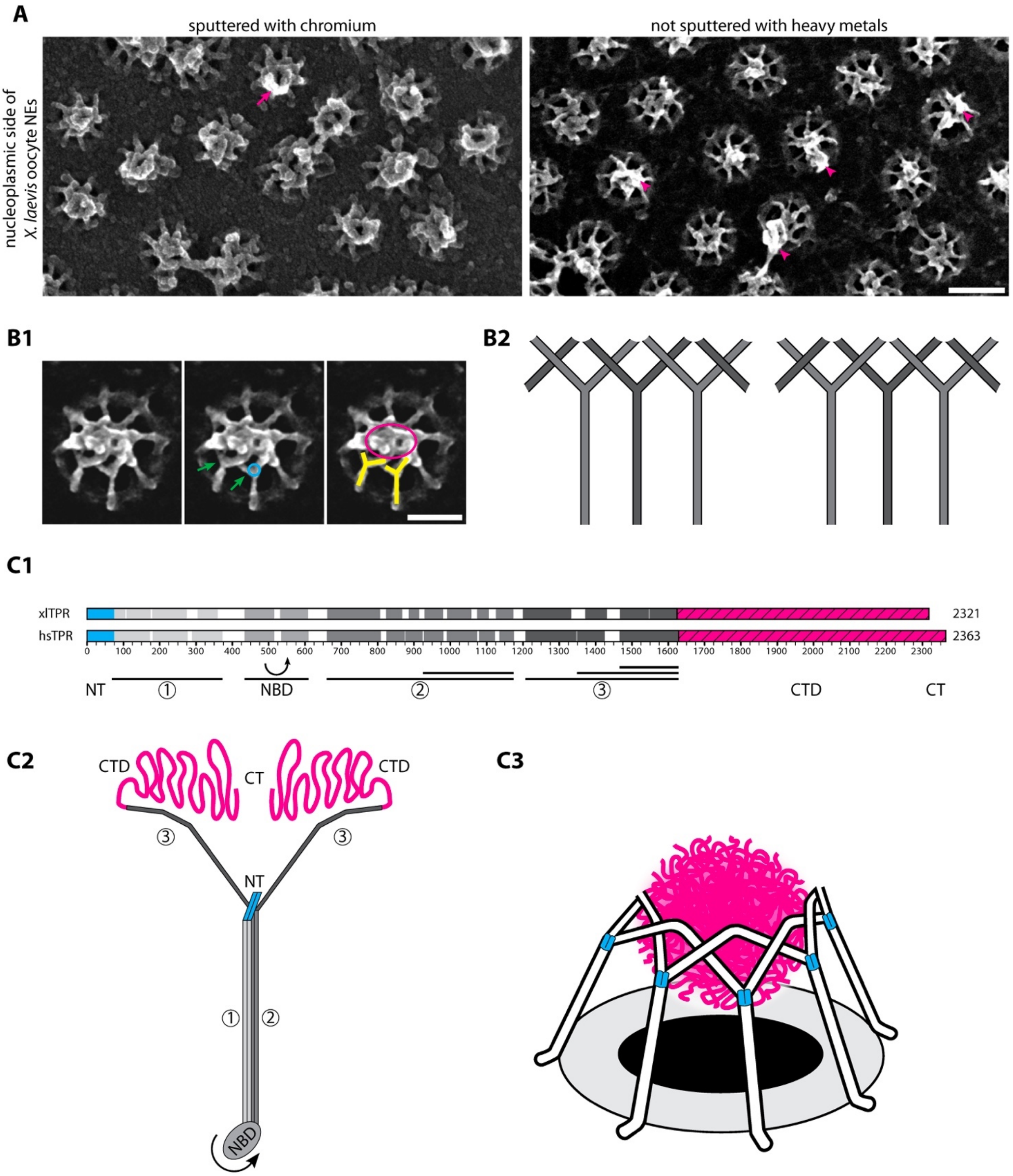
A
sputtered with chromium
not sputtered with heavy metals
nucleoplasmic side of
X. laevis oocyte NEs
B1
B2
C1
xlTPR
hsTPR
2321
2363
NT
①
NBD
②
③
CTD
CT
C2
CTD
CT
CTD
③
③
NT
①
②
NBD
C3

S03 (1/4)

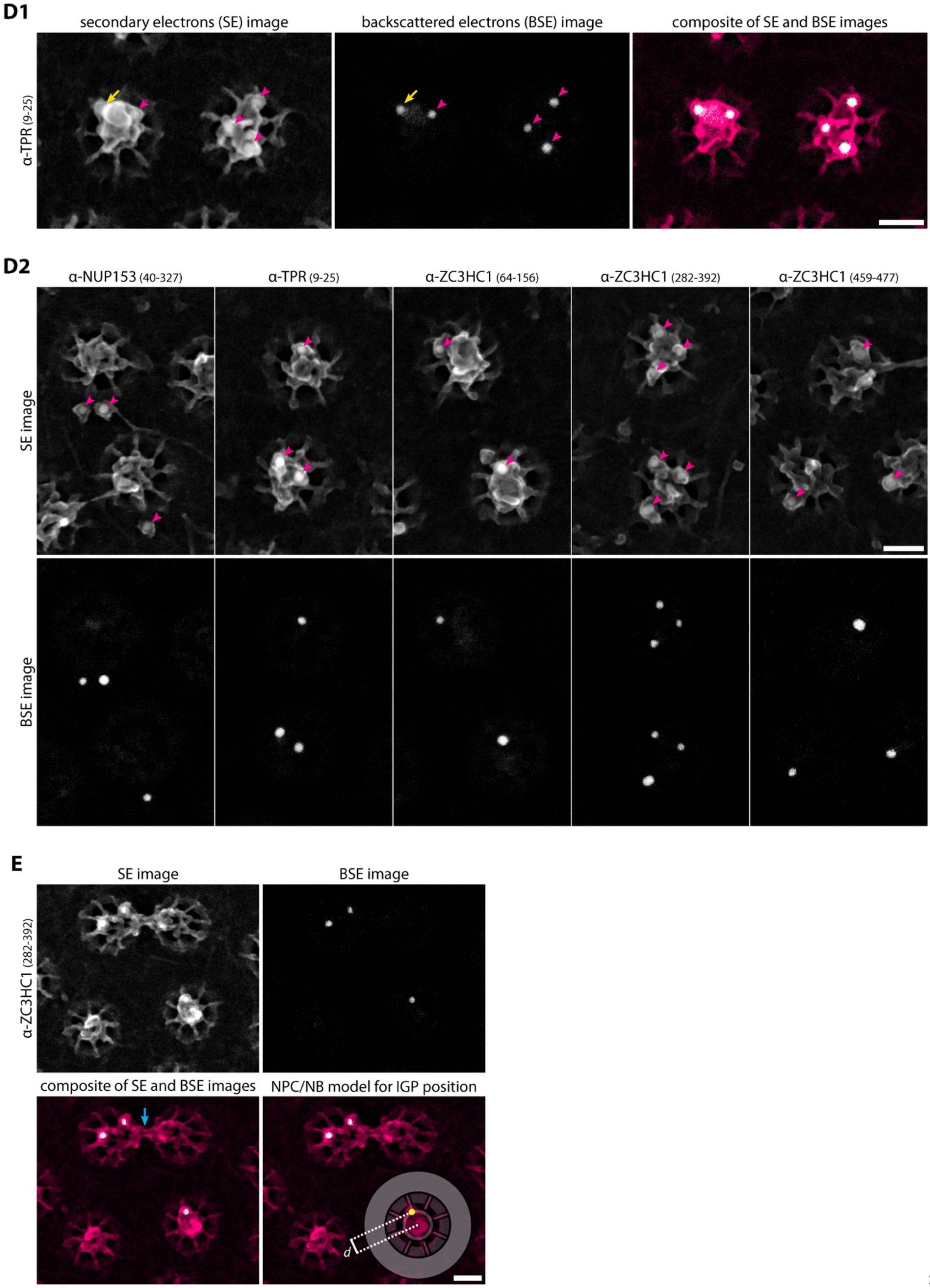
D1
secondary electrons (SE) image
backscattered electrons (BSE) image
composite of SE and BSE images
α-TPR (9-25)
D2
α-NUP153 (40-327)
α-TPR (9-25)
α-ZC3HC1 (64-156)
α-ZC3HC1 (282-392)
α-ZC3HC1 (459-477)
SE image
BSE image
E
SE image
BSE image
α-ZC3HC1 (282-392)
composite of SE and BSE images
NPC/NB model for IGP position
d

S03 (2/4)

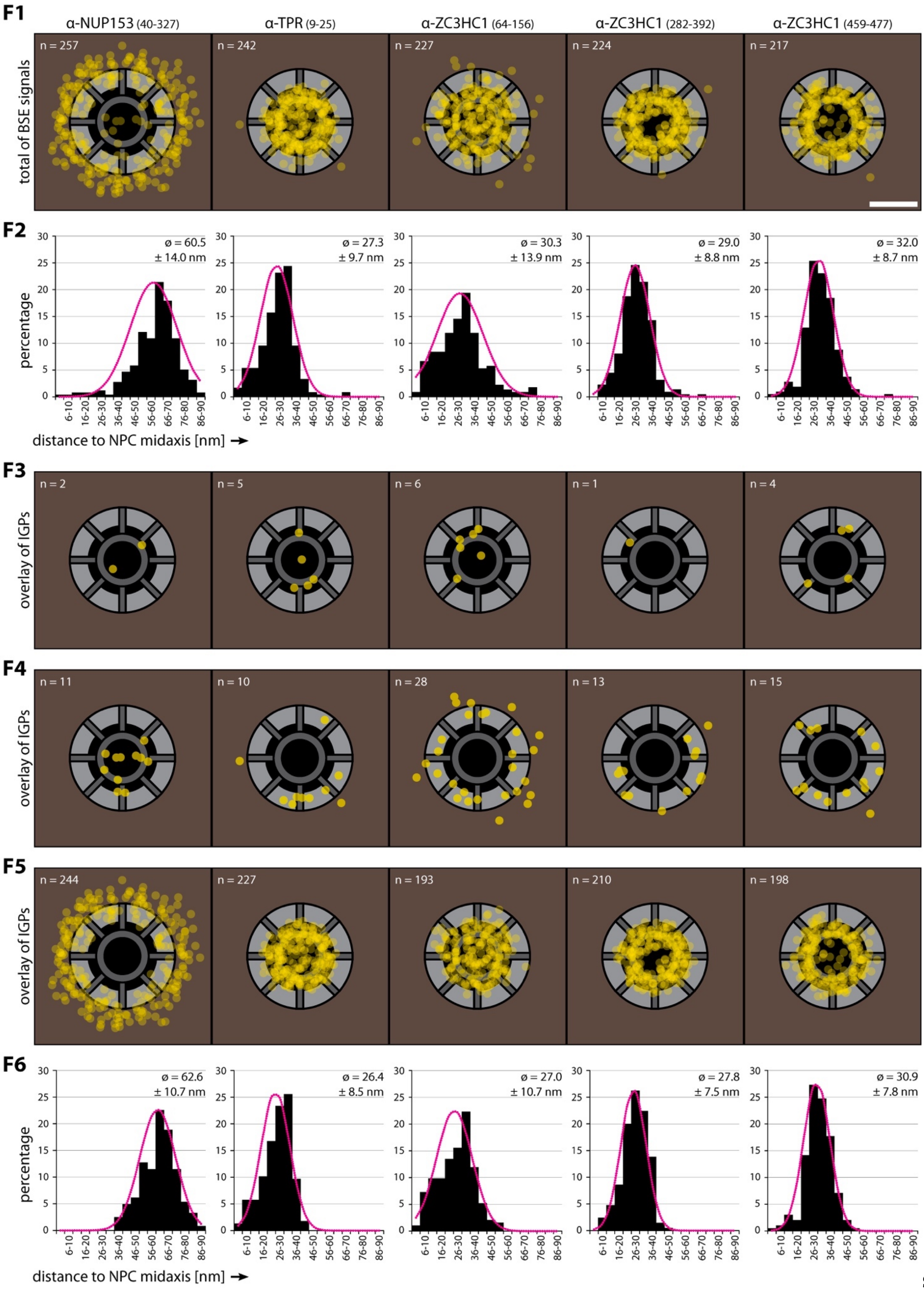
F1
α-NUP153 (40-327)
α-TPR (9-25)
α-ZC3HC1 (64-156)
α-ZC3HC1 (282-392)
α-ZC3HC1 (459-477)
total of BSE signals
n = 257
n = 242
n = 227
n = 224
n = 217
F2
percentage
ø = 60.5 ± 14.0 nm
ø = 27.3 ± 9.7 nm
ø = 30.3 ± 13.9 nm
ø = 29.0 ± 8.8 nm
ø = 32.0 ± 8.7 nm
6-10
16-20
26-30
36-40
46-50
56-60
66-70
76-80
86-90
distance to NPC midaxis [nm] →
F3
overlay of IGPs
n = 2
n = 5
n = 6
n = 1
n = 4
F4
overlay of IGPs
n = 11
n = 10
n = 28
n = 13
n = 15
F5
overlay of IGPs
n = 244
n = 227
n = 193
n = 210
n = 198
F6
percentage
ø = 62.6 ± 10.7 nm
ø = 26.4 ± 8.5 nm
ø = 27.0 ± 10.7 nm
ø = 27.8 ± 7.5 nm
ø = 30.9 ± 7.8 nm
distance to NPC midaxis [nm] →

S03 (3/4)

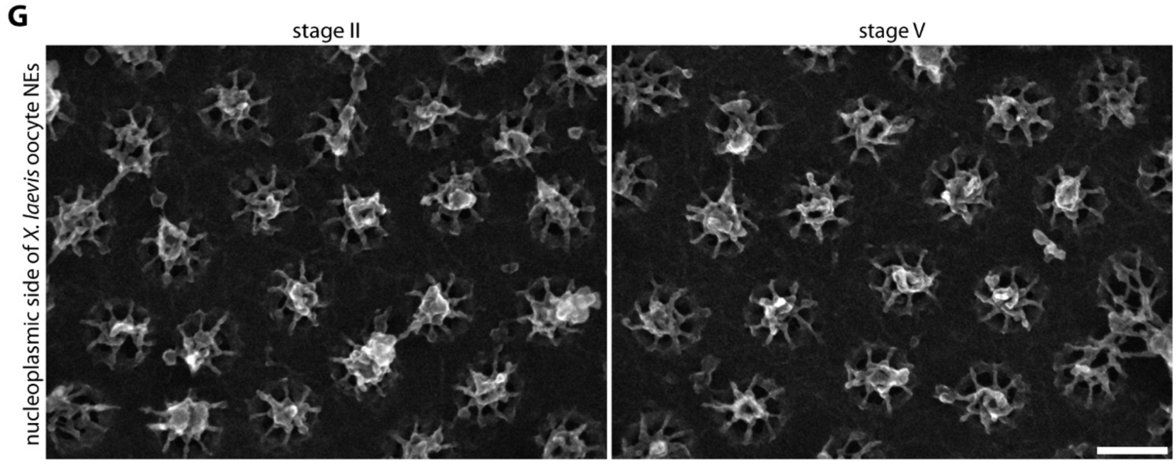

S03 (4/4)

Supplemental Figure S3. Background information, preconditions, procedural approaches, and resulting datasets for distance measurements between NPCs, NBs, and immunogold-labelled components visualised at *Xenopus* oocyte NEs by SEM.

(A) The appearance of *Xenopus* NEs and their NBs without and after conventional sputter-coating with chromium. Most of the standard procedures of preparing manually-isolated *Xenopus* NEs for inspection by SEM involve the coating of specimens with metals, such as chromium, as a generally considered necessity for high-resolution SEM. This is achieved either via sputtering or electron beam evaporation [e.g., 49–51]. However, it is actually possible to omit this particular step of metal deposition without provoking major charging artefacts due to electrons accumulating at the NE's surface. Instead, the non-coated NEs can be sufficiently conductive even without an additional metal coat and then unveil a more filigree NB, including details of its morphology commonly masked beneath an often more than 1.5 nm thick layer of surface-deposited metal. In the example presented on the left, the specimen had been sputter-coated with chromium up to a predefined final layer thickness of about 1.2 to 1.5 nm. By contrast, sputter-coating had been omitted for the sample on the right, with the example shown here displaying the upper limit of the degree of charging that we could occasionally note at the top of the NBs, where such charging likely affected NB-appended materials (some marked by arrowheads in magenta) that had collapsed onto the prototypic NB. However, such charging was sometimes even noted at the heavy-metal-coated NBs (an example marked with a magenta-coloured arrow). Bar, 100 nm.

(B) In addition to allowing for detecting IGPs in the SE modus (see below), the omission of heavy metal-coating also made it possible to more frequently detect additional structural features at the distal end of the NB. Such visualisation confirmed that the so-called TR is actually not a simple ring but rather a far more complex arrangement of branched, Y-shaped fibrillar elements, as observed and illustrated earlier, with those authors already then having depicted a "*proposed weave of the filament branches to make the basket ring*" [52].

(B1) Example of an NPC-attached *Xenopus* oocyte NB commonly regarded as prototypic. Each arrow in green points at one of those sites where each of the NB's rectilinear fibrils bifurcates at its distal end. Some of the more clearly visible Y-shaped filaments are shown overlaid with the schematic depiction of these structures in yellow. These are meant to be equivalent to the homomeric arrangement of those TPR polypeptides that constitute one of the NB's fibrils, as described in S3C below. The blue-coloured circle marks the proposed position of the TPR N-termini at one of the NB's fibrils, based on former data and conclusions outlined in S3C. The ellipsoid in magenta surrounds the approximate positions of the here mainly FA/GA-cross-linked carboxy-terminal domains of the TPR polypeptides, based on a former study [9] and our unpublished data. Bar, 50 nm.

(B2) Simplifying schematic depiction of the bifurcating parts of the NB fibrils that sometimes appeared to be interlaced in an alternating manner with the corresponding part of the directly neighbouring fibril (left row). In other cases, both of the bifurcating arms of a fibril were found located beneath the bifurcating parts of the two flanking NB fibrils (right side).

(C) TPR homomers as the scaffold-forming components of the NPC-attached, prototypic NB in *Xenopus* oocytes. In the past, we had found that parts of TPR's large, heptad-repeat (HR)-dominated domain, capable of forming coiled-coil homomers of mainly rectilinear, rod-like shape, are also capable of forming bifurcations which can occur at one or more sites of the domain's second half [8, and our unpublished data]. We had further noted that each of the first two thirds of the HR domain was not only capable of forming stable homodimers in parallel and in register but also that the first and the second third tended to interact with each other as well, while neither of them did so with the last third. Furthermore, in its monomeric form, this last third of the coiled-coil domain was found to only engage in weak homodimeric interactions in parallel, suggesting an anti-parallel arrangement instead [8, and our unpublished data]. Based on these and further observations, we formerly proposed an NB model in which homomeric arrangements of TPR are forming the scaffold of

the NB [9]. In this model, the last one-third of the rod would branch off toward the distal rod ends of adjoining TPR polypeptides that constitute the neighbouring basket fibrils, thereby forming the TR structure. The TPR polypeptides' carboxy-terminal domains, regarded as being to a large extent unstructured and as such highly flexible [8,18,53, and our unpublished data] would then project away from the TR, either deeper into the nuclear interior or towards the NPC, into the area bounded by the rectilinear NB fibrils. Now merging these results, we present part of an updated version of the 2004 model, here showing the NB commonly regarded as prototypic, in order to allow for correlating the immuno-EM localisation data presented in the current study with the different parts of the NPC-associated version of the NB built of TPR.

(C1) Schemes of *Xenopus laevis* and human TPR. The boxes in grey represent segments with consecutive copies of the HR motif for which 80% coiled-coil probability is predicted within windows of 28 aa by the PCOILS algorithm [54], with the different shades of grey corresponding to the grey tones also used for the model presented in S3C2 [for an earlier coiled-coil prediction of human TPR, see 8]. The segment in blue represents the N-terminal region, while the hatched segment in magenta depicts the C-terminal domain (CTD), which we regard as largely unstructured and natively unfolded. Bars either numbered 1-3 or labelled NBD together with an arrow bending backwards, as well as the blue- and magenta-coloured segments, correspond to those parts labelled and coloured accordingly in S3C2. Shorter bars in segments 2 and 3 are meant to illustrate shorter coiled-coil segments that are flanked by short, potentially hinge-like and bifurcation-forming sequence stretches, involving evolutionary-conserved prolines or other coiled-coil-disrupting features at these sites (data not shown).

(C2) Model for a TPR homodimer folded back onto itself. Based in parts on a model proposed earlier [9], the first of the first two thirds of TPR's rod domain, known to form homodimeric coiled coils, with the monomers therein being arranged in parallel and in register [8], is proposed to fold onto the second third, thereby resulting in an anti-parallel arrangement of these parts relative to each other, and to thereby expose the NPC-binding domain (NBD) of TPR. Eight of these homodimers each are then thought to bind to the outer and the inner of the two NUP107-containing Y-complex rings, respectively, with these two rings together forming the nuclear ring. The third HR-dominated segment of TPR's rod domain is then proposed to branch away from its correspondent in the NB fibril. Such branching might occur at either one or another of at least two possible sites within the second half of TPR's HR-domain, resulting in the homomeric assembly's Y-shaped bifurcation. The CTD of each monomer would then emanate from the outermost tip of the branched-off arm of such a Y-shaped structure, which actually is not to be confused with the NPC's Y-complex, while the N-termini of the dimeric or tetrameric TPR homomers are thought to be positioned close to the site of bifurcation.

(C3) Model of the prototypic NPC-anchored NB. Note that for reasons of clarity, only five of the NB's eight longitudinal fibrils are schematically depicted. Based on all of our currently available data, we suggest that homodimers of TPR represent the major architectural subunit of the NPC-anchored NB, with each of the NB's eight rectilinear fibrils made up of two of such homodimers, possibly engaging in homotetrameric interactions. The arrangement commonly referred to as the TR would be formed by the bifurcating parts of such NB fibrils that possibly interact with the branched-off parts of the over-next NB fibrils. However, it remains to be resolved whether the arrangements between the branched-off TPR segments include anti-parallel coiled-coils between monomeric parts or anti-parallel interactions between the TPR dimers or tetramers. The largely unstructured CTDs of the TPR polypeptides, by contrast, would together form a meshwork in a region largely confined laterally by the TR but nonetheless capable of extending both closer to the NPC and further away from it. We wish to remark that this simplified model does not claim to be complete, and it actually lacks yet other NB-resident proteins also contributing to certain features of the NB and its overall appearance (our unpublished data).

(D) Omission of metal-coating allows for the visualisation of immunogold particles (IGPs) via secondary electron (SE) detection. While sputter-coating of the NE usually also results in masking the majority of IGPs bound to an object of interest, requiring IGP positions to be conjectured from the detected backscattered electrons (BSE), it is possible to directly detect such IGPs in the SE modus when these are located on the surface of an object of interest and not buried beneath a layer of metal.

(D1) Exemplary micrographs of IGP-decorated NBs, here following immunolabelling with an antibody against TPR's N-terminus. Images were obtained with the SE and BSE detector (left and central image), respectively, and then superimposed onto each other, resulting in the assembly at the right, in which the SE image had been false-coloured in magenta. Note that four IGPs were visible in the SE modus (arrowheads in magenta) and could be assigned to four of the five BSE signal dots. The IGP that needed to be present in order to have allowed for the fifth BSE signal dot, but which nonetheless was not visible in the SE modus (yellow arrow), might have been buried beneath some materials of unknown kind that appeared to have collapsed onto the NB's terminal ring. Bar, 50 nm.

(D2) Raw data of those SE and BSE images presented in the main Figure 2A as merged images superimposed onto each other. The IGPs visible in the SE modus are marked by arrowheads. Bar, 50 nm.

(E) Procedure of aligning a merged SE and BSE image of an IGP-decorated NB to the scheme of an idealistic NPC and its NB. Superimposition of the exemplary micrographs obtained with the SE and BSE detector (first and second image in the

upper panel) resulted in the composite image in which the SE image has been false-coloured in magenta (first image in the lower panel). The scheme of an idealistic face-on view NPC-NB model, here surrounded by an additional grey-coloured ring with an outer radius of 90 nm, was then superimposed onto an IGP-decorated, morphologically intact NB of prototypic appearance (second image in the lower panel). The scheme was then rotated until the model's NB fibrils and the NR were best aligned with the corresponding parts of the authentic NPC and its NB located closest to the IGP. At this position of alignment, the distance *d* between the centre of the BSE signal to the medial, i.e., longitudinal axis of the NPC-NB was measured. By contrast, sideward-tilted NBs that were laterally interconnected with other NBs were excluded from such distance measurements (see also additional information provided together with Table S1). The blue-coloured arrow marks such an example of an interconnected pair of NBs, of which one was decorated with ZC3HC1 IGPs but excluded nonetheless. Bar, 50 nm.

(F) Distributions of BSE signals relative to an idealistic NPC and its NB in a face-on view. Data for each target epitope comprised at least 200 imaged BSE emission sites at NPCs with an intact NB and discernible edges of the nuclear and terminal rings. Such NPCs had been chosen randomly, except for having excluded NBs that were laden with notable amounts of additional fibrillar material that appeared to have collapsed onto them, and NBs that were laterally interconnected with neighbouring ones. Each BSE signal position was plotted onto the matrix after fine-tuning the alignment of BSE, SE and matrix images in such a way that the plot displayed the true-to-scale distance between BSE signal and nearest NB fibril and ring edge (for further details, see Figure S3E). Based on the NPC's and NB's eightfold rotational symmetry, some few BSE signal positions were rotated around the NB's medial axis by 45° or multiples thereof to allocate similar numbers of IGPs to each eighth part of the NB for better visibility.

(F1 and **F2)** Distributions of all BSE signals at stand-alone NBs, irrespective of whether assignable to a visible IGP or not, and corresponding diagrams. The bar in S3F1 represents 50 nm and also applies to S3F3-S3F5. The principles of presenting the signals' radial distances from the NB's longitudinal axis, in S3F2 and S3F6, are the same as in the main Figure 2C, except for having here included magenta-coloured standard distribution curves, calculated on the basis of all of each dataset's measurement values.

(F3) Sub-datasets of those BSE signals that could not be assigned to any IGPs visible in the SE modus.

(F4) Sub-datasets comprising only BSE signals that could be unambiguously assigned to an IGP but did not locate at the target protein's principal site of location, considered the NR for the NT-proximal part of NUP153 and the TR for TPR's NT and ZC3HC1.

(F5 and **F6)** Sub-datasets obtained when subtracting the datasets presented in S3F3 and S3F4 from the data in S3F1. Also, note that corresponding datasets presented in the main Figure 2 resulted from subtracting only the sub-datasets in S3F3 from their corresponding total datasets presented in S3F1.

(G) NEs shown here had been isolated on the same day from stage II and stage V oocytes of the same slim frog. Mounted onto the same silicon chip, they had then been processed for SEM together. While the NPCs and their attached NBs generally tend to be arranged in a slightly more densely packed fashion in stage II than in stage V, in line with findings of NPC densities in *Xenopus* oocyte NEs gradually decreasing in the course of oogenesis [e.g., 55, and our unpublished data], the overall morphology of the NB proper appeared being very similar in these stages of oogenesis, even though the diameter of the NPC proper has been reported to diminish to some extent between stage II and later stages of oogenesis [55]. Further note that among the features common to the NBs of both stage II and V oocytes are the two different arrangements by which the fibrils' bifurcations can interlace, as already illustrated in S3B2, and, in particular, the bifurcation of the NBs' fibrils at their distal ends, i.e., close to the TR. Bar, 100 nm, same magnification for both micrographs.

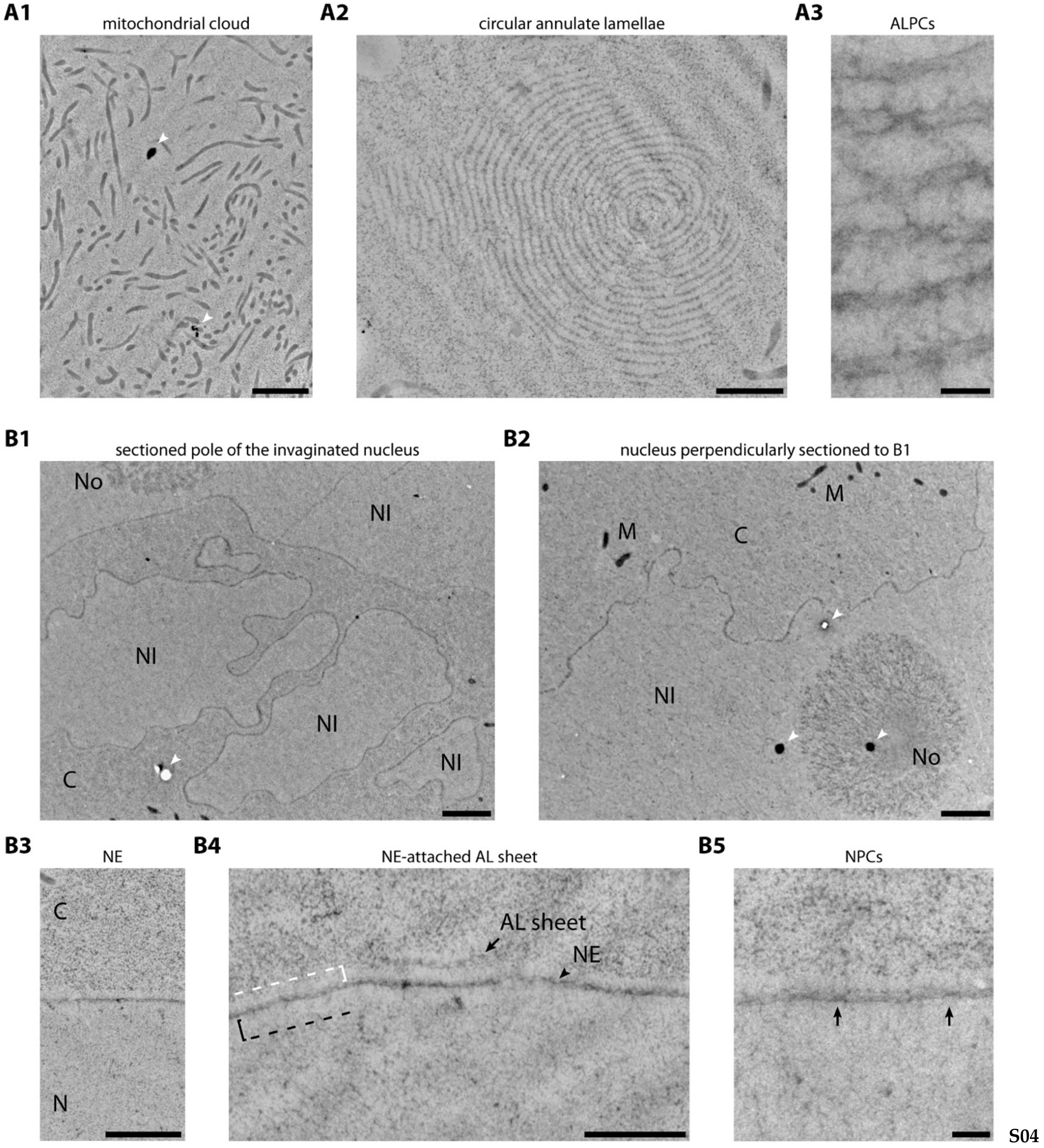

Supplemental Figure S4. The appearance of subcellular features of late-stage II and early-stage III oocytes, following high-pressure freezing, freeze-substitution in the absence of chemical fixatives, and embedding in the hydrophilic resin K4M.

Some of the epitopes in oocytes to be targeted by antibodies in the context of the current study did not sufficiently well tolerate the conditions characteristic for conventional post-embedding iTEM, namely the chemical fixation of samples at RT, with fixatives like FA and GA, and the subsequent embedding of such specimens in resins like, for example, LR White [see also 9]. Therefore, these epitopes made it necessary to develop a post-embedding protocol for *Xenopus* oocytes based on high-pressure freezing and freeze-substitution in the absence of chemical fixatives. Only this would allow for still profiting from the formerly discussed advantages of post-embedding over pre-embedding iTEM concerning the accuracy of target site localisation [e.g., 9,56]. In the following, ultrathin sections obtained from such high-pressure frozen and freeze-substituted late-stage II oocyte are shown as examples of their appearance before immunogold-labelling,

demonstrating uniform vitrification and illustrating some characteristic features in the cytoplasm (S4A) and at the NE (S4B).

(**A** and **B)** Transmission electron micrographs, representing mostly overview images at different magnifications of ultrathin sections of high-pressure-frozen and then K4M resin-embedded oocytes that had been freeze-substituted in the absence of cross-linking fixatives such as GA and OsO_4 in order to maintain antigen integrity. Note that the specimens' relatively low contrast was not only due to having omitted OsO_4 but also because of constraints in *en bloc*-staining with UA (see Information SI 6). Black and white objects and circles, some of which are marked by white arrowheads, were interpreted as possibly representing contaminating non-biological materials or holes within a section.

(**A1)** Image showing numerous mitochondria, crowded within a cytoplasmic area called the mitochondrial cloud, characteristic for the previtellogenic oocytes of *Xenopus laevis* [e.g., 57].

(**A2** and **A3)** A spiral-shaped version of an annulate lamella (AL) harbouring numerous AL pore complexes (ALPCs), and higher magnified examples of ALPCs in cross-section, as shown in S4A3. Such spirally wound AL are characteristic for the previtellogenic oocytes, while the often very much larger, sometimes gigantic AL in the full-grown immature oocytes of *Xenopus* and other amphibians occur as stacks of numerous sheets that can be dozens of micrometres long [e.g., 58–60]. As an aside, the wave-like shades of grey seen in this image were due to folding of the ultrathin section that had occurred upon water contact and spreading onto a grid, representing a common handling effect with ultrathin sections of oocyte materials embedded in the hydrophilic resin K4M. While such folding can be undone, for example, by "chloroform vapour flattening" or "heat wand flattening" [e.g., 61], this was not done with this particular section.

(B1 and **B2)** Parts of the highly invaginated NE of the previtellogenic oocyte, with S4B1 representing the top-on view of a spherical slice through a nucleus near its surface, with some parts of the nuclear interior (NI) appearing surrounded by cytoplasm (C), which due to being crowded with myriads of ribosomes here appears darker than the NI. Both compartments, though, are clearly separated from each other by the NE, which here appears even darker due to its high density of NPCs. In S4B2, the oocyte's nucleus was sectioned perpendicular to the plane of the section presented in S4B1. Whilst now showing the more familiar appearance of only two distinct compartments separated from each other, *videlicet* cytoplasm (C) and nuclear interior (NI), this section too illustrates the pronounced folding of the NE. In addition, a sectioned nucleolus (No) and a few mitochondria (M), which in the previtellogenic oocytes also occur outside of the mitochondrial cloud, can be seen.

(B3 to **B5)** Examples of two distinct zones adjacent to the NE's cytoplasmic (C) and nucleoplasmic (N) sides, with these zones visible at higher magnification. The zone at the cytoplasmic side, which largely excludes the ribosomes evenly distributed throughout the cytoplasm, was noted to have a width mainly varying between 70-90 nm, with a short stretch highlighted by the white half-bracket in S4B4. While this zone was barely or not at all visible in high pressure-frozen oocytes that had been freeze-substituted with hydrophobic resins in parallel, like HM20 and HM23, we regard it as being equivalent to the ribosome exclusion zone of similar width that has already been described in other vertebrate cell types processed for electron microscopy using other protocols. This zone appears to be established by filamentous proteins appended to the NPC's cytoplasmic side, and in vertebrates, NUP358 is one of the candidate proteins contributing to their formation [e.g., 18,62,63]. Similar but less broad ribosome-exclusion zones in other organisms, which lack NUP358, might be formed by other fibrillar NPC proteins [e.g., 64]. While the highly uniform distribution of the ribosomes throughout the cytoplasm, as exemplified in S4B3, already represented one of several arguments against the notion that exclusion zones might merely represent an artefact due to some alleged formation of ice crystals along the NE, the single AL sheet here seen adjacent to the NE in S4B4 (arrow) represented another argument in favour of a genuine exclusion zone at the oocyte NE's cytoplasmic side. Such NE-associated single AL sheets are characteristic for both the previtellogenic oocyte, as shown here, as well as the full-grown immature oocyte [e.g., 10,26]. The distance between the NE and such an AL sheet, which was found to be only slightly larger than the exclusion zone's width, was very similar to that also seen in chemically fixed specimens inspected by TEM [10] and by SEM of manually isolated oocyte nuclei when inspected at different tilt angles (data not shown). Therefore, in light of such seemingly rather unchanging spacing between the NE and the NE-associated AL sheet, irrespective of the mode of sample preparation, one could imagine this exclusion zone, respectively the proteins forming it, to be determining the seemingly set distance between the NE and the NE-associated AL sheets.

Apart from the zone at the NE's cytoplasmic side, a nucleoplasmic zone also adjacent to the NE, and distinct from the NI's appearance deeper within the nucleus, can be seen in images S4B3 to S4B5. With a short stretch again highlighted by the black half-bracket in S4B4, this zone was found to be mostly broader and of more varying width than the NE-adjacent cytoplasmic zone. We regarded this zone to include the NPC-appended NBs and sometimes additional fibrillar material, the latter occasionally appended to these NBs already in the early stages of oogenesis, where their amounts could notably vary at different sites along the NE, with those in the examples shown here already appearing substantial. In addition, two clearly discernible NPCs in cross-section are marked by arrows in S4B5. Bars, 2 µm (S4A1, S4A2, S4B1 and S4B2), 1 µm (S4B3 and S4B4), 200 nm (S4B5), and 100 nm (S4A3).

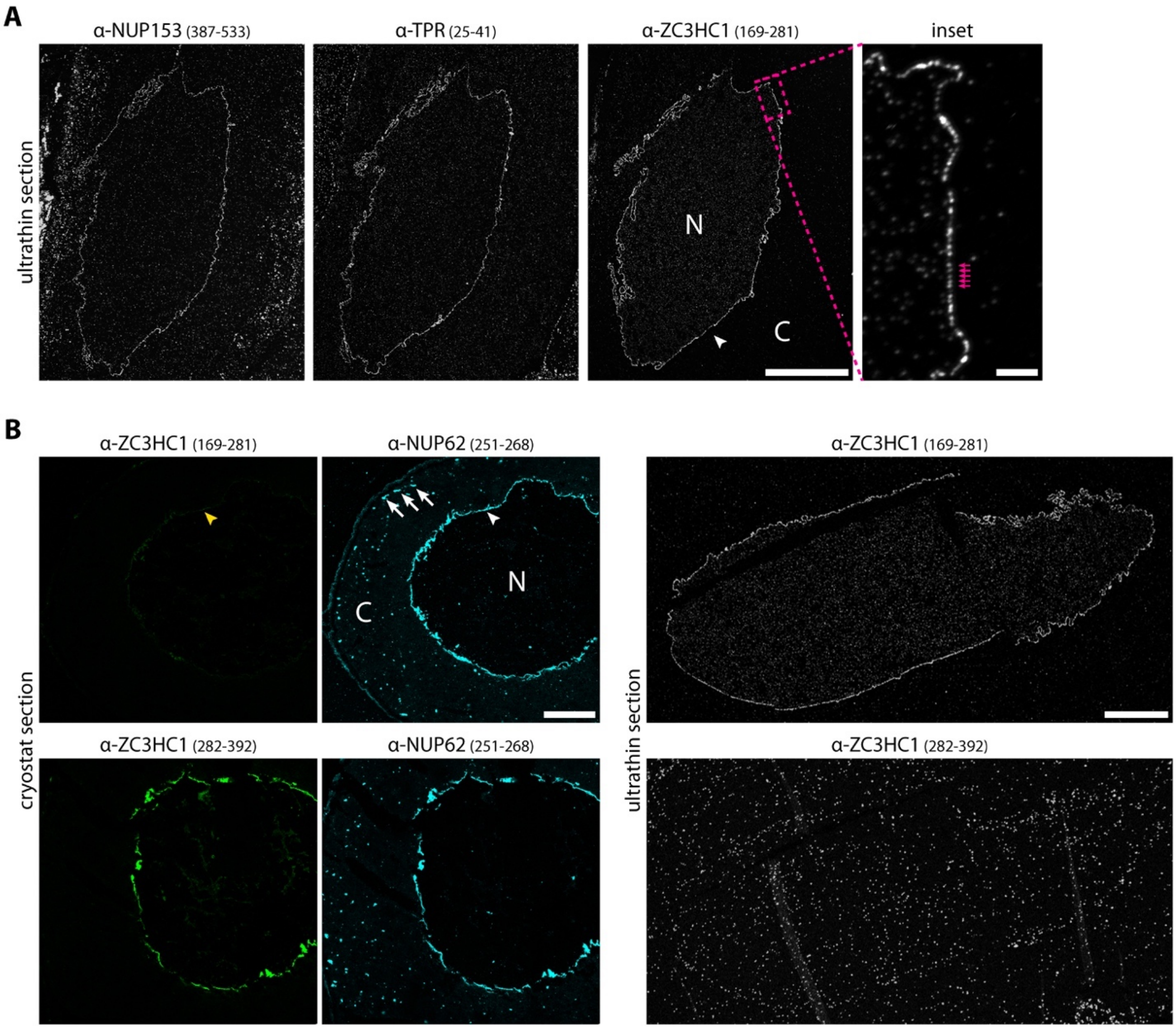
A
α-NUP153 (387-533)
α-TPR (25-41)
α-ZC3HC1 (169-281)
inset
ultrathin section
N
C
B
α-ZC3HC1 (169-281)
α-NUP62 (251-268)
α-ZC3HC1 (169-281)
N
C
cryostat section
ultrathin section
α-ZC3HC1 (282-392)
α-NUP62 (251-268)
α-ZC3HC1 (282-392)

S05 (1/2)

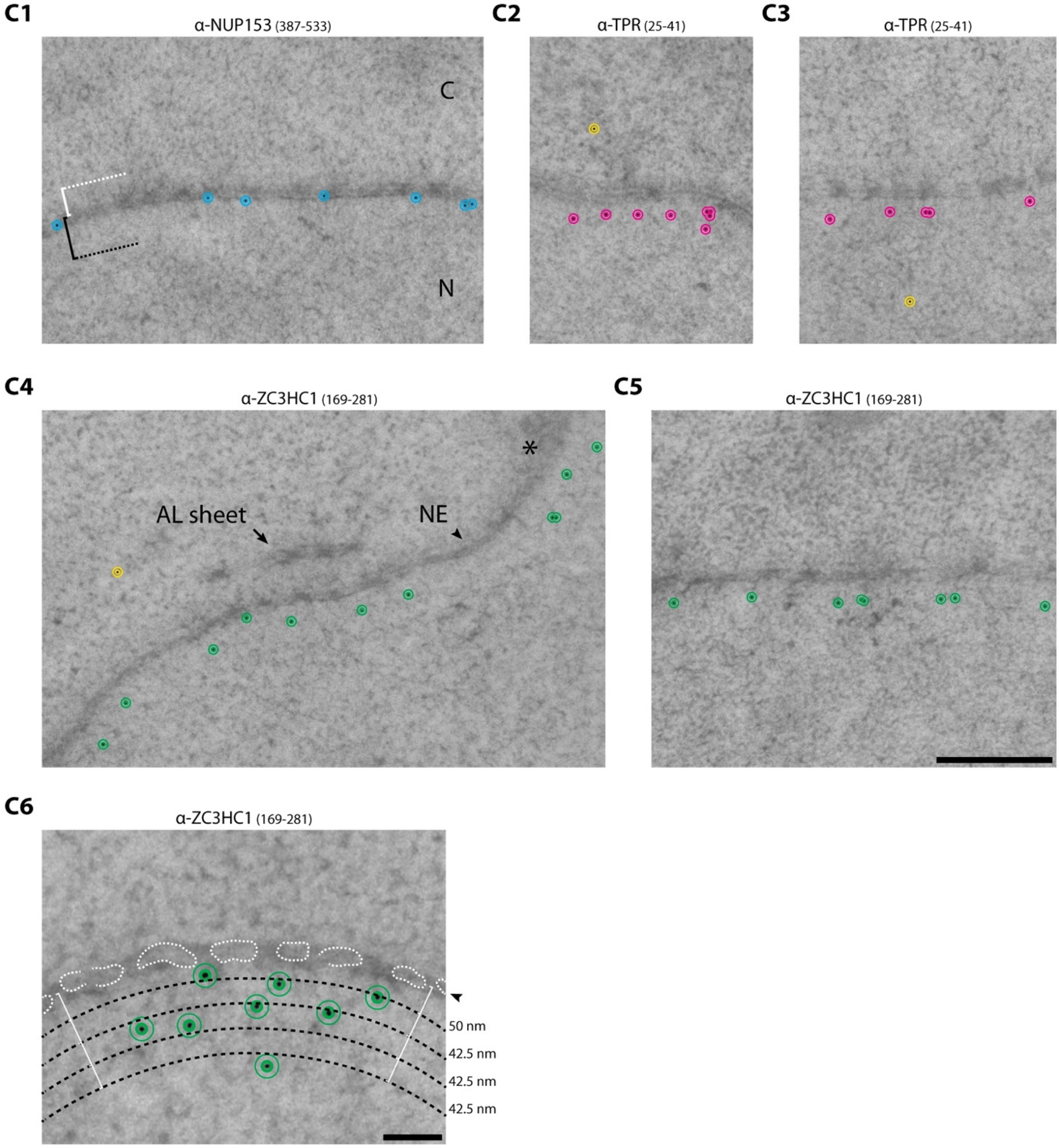

S05 (2/2)

Supplemental Figure S5. Suitability assessment of high-pressure frozen and freeze-substituted late-stage II oocytes for immunolabelling, using IFM for rapid screening of antibody performance on ultrathin sections and iTEM for the identical specimens' subsequent inspection with selected antibodies.

(A) IFM of ultrathin sections from the same group of K4M-embedded *Xenopus laevis* oocytes also used for Figure S4. Sections had been immobilised on glass coverslips and then immunolabelled with primary antibodies and fluorophor-conjugated secondary antibodies. The labelling protocol used was similar to the corresponding immunogold-labelling procedure for such ultrathin sections immobilised on copper grids, as exemplified further below, but omitting post-fixation with GA and post-staining with UA. Representative epifluorescence micrographs were from sections stained with collections of NUP153, TPR and ZC3HC1 antibodies, of which those presented here were then also used for the immuno-TEM data presented in the main Figure 2D-F. Cytoplasmic (C) and nuclear compartment (N) are marked, as is the NE (arrowhead). As an aside, note that rather elliptic shapes of the sectioned nuclei were not uncommon, given that the aluminium planchets used for HFP had cavity depths of about 150 µm, while the diameters of stage II oocytes range between 300 and 400 µm. Further note that the sections shown here had been labelled with only one antibody each. The

inset shows part of the NE from the ZC3HC1-labelled ultrathin section (magenta-coloured rectangle) at higher magnification, with some of the labelled NPCs marked by arrows. IFM on such ultrathin sections of resin-embedded oocytes allowed for rapidly screening antibodies and accessing their performance at different antibody concentrations prior to the more time-consuming procedure of immuno-TEM labelling and inspection of immunogold-labelled specimens. Bars, 50 µm and 2.5 µm for overview and inset, respectively.

(B) IFM of stage VI oocyte cryostat sections, and of ultrathin sections of a K4M-embedded late-stage II oocyte for direct comparison, with rabbit antibodies against xlZC3HC1 that either performed well or not on the one or other type of sectioned oocyte material. The cryostat-sectioned oocyte was double-immunolabelled with an xlNUP62-specific monoclonal antibody for visualising the NE. Note that some antibodies, like the one targeting aa 282-392 of xlZC3HC1 shown here as an example, hardly labelled the oocytes when embedded in K4M, even though the same antibodies labelled the cryostat-sectioned NE well for IFM (see also Figure S1F), and, equally well, the NBs of manually isolated NEs in iSEM, as shown in Figure 2A and Figure S3D2 and S3E. This indicated that the corresponding antigenic determinants had been altered or masked during the K4M freeze-substitution process and thus inaccessible for those antibodies. By contrast, other ZC3HC1 epitopes, like the ones located between aa 169-281, were well accessible on the ultrathin-sectioned K4M material, whilst they had been hardly accessible for antibodies on the several micrometre thick cryostat sections (yellow arrowhead), which had been air-dried and later FA-fixed prior to their use for IFM. Furthermore, these epitopes had also been poorly accessible when NBs had been fixed with FA prior to immunolabelling for iSEM (data not shown). By contrast, their good labelling then notable in post-embedding iTEM might have been due to some antigen-exposing step in the course of the freeze-substitution process, or the omission of FA, or a result of exposing these epitopes by the actual ultrathin sectioning. Cytoplasmic (C) and nuclear compartment (N) are exemplarily marked, as is the NE (white arrowhead) and some of the NUP62-positive AL (white arrows). Bars, 100 µm and 25 µm for cryostat and ultrathin sections, respectively.

(C1 to C5) Overview images of immunogold-labelled sections incubated with the same antibodies used for the IFM data presented in Figure S5A and the iTEM data presented in Figure 2D. In order to highlight the IGPs on these images, they were double-encircled, as in Figure 2D, in blue, green and magenta, for NUP153 (S5C1), TPR (S5C2 and S5C3) and ZC3HC1 (S5C4-6), respectively. Since the centre of an individual gold particle in indirect post-embedding immunogold-labelling can be located up to about 20 nm away from its actual target site in all directions when using IgGs as primary antibodies and 10 nm-gold-coupled $F(ab')_2$ fragments for secondary labelling, the outer coloured circles surrounding each IGP, with a diameter of 40 nm, are meant to encircle the areas in which one would expect each grain's actual target site to have been located. This explains why, for example, an IGP meant to have labelled a part of NUP153 located at the NPC's NR could even be found close to the NE midplane, as seen, for example, in one case in Figure 2D and two cases in S5C1. Furthermore, IGPs that are shown yellow-encircled are further away from the NE, outside the range of distances for which measurements are presented in the current study**.** Cytoplasm (C) and nucleoplasm (N), separated by the NE, are oriented toward the top and bottom. Like in Figure S4B4, the white half-bracket marks part of the zone of ribosome exclusion at the NE's cytoplasmic side, while a black half-bracket again marks part of the zone defined by NBs and possibly additional fibrillar material at the NE's nuclear side. An NE-associated single-sheet AL visible in one of the images is marked by an arrow, while the asterisk labels an NE segment mainly seen as a grazing section, i.e., mainly in a top-on view, whereas most of the NE visible in this image has been perpendicularly sectioned. The corresponding NE torsion of about 90°, separating the perpendicularly sectioned NE segment from the parallelly sectioned one, was located somewhere between the positions demarked by the arrowhead and the asterisk. Therefore, the few IGPs seen close to the asterisk did not reflect NE-to-IGP distances in Z but rather are IGPs only decorating slices parallel to the NE midplane, with the corresponding parts of the NPCs cut off as part of the preceding ultrathin section. However, immunolabelling of the other, largely perpendicularly sectioned NE segments revealed that those IGPs decorating NUP153 were mainly positioned directly at or very close to the nuclear side of the NE or NPC, while those IGPs for both TPR and ZC3HC1 were notably more distal. Bar, 500 nm, same magnification for S5C1-S5C5.

(C6) Example of the type of locally confined regions sporadically observed along the nuclear side of the NE already in late-stage II oocytes, in which decoration with TPR IGPs (data not shown) and ZC3HC1 IGPs could be found notably deeper within the nuclear interior. The patterns in which the IGPs were found distributed within such regions sometimes appeared regular, as highlighted by the dashed lines, the latter separated from each other in steps of about 42.5 nm (in this context, also see Figures S6C and S7B3). We could not in all cases exclude that some of these regions merely represented grazing sections close to the NE, with the IGP pattern then merely representing labelled NBs that had been sectioned perpendicularly to their longitudinal axis, like in the asterisk-marked region in S5C4. However, the NEs next to other regions with such distinct distribution of IGPs appeared to have been perpendicularly sectioned, supporting the notion that the labelled sites might well represent such truly located deeper within the nuclear interior. Here, to facilitate identification, the NE of this example is highlighted by white dashed lines. For further orientation, the two white lines, perpendicular to the midplanes of two cross-sectioned NPCs, and the curved dashed black lines in parallel to the NE, are provided as guides. We consider it likely that such regions correspond to those that have been recurrently described in conventional TEM studies of the *Xenopus* oocyte, in which NPC-associated accumulations of fibrillar materials projecting

deeper into the nuclear interior, and often appearing interwoven with each other, have been noted to occur especially in regions in which the oocyte's NE is more strongly curved [e.g., 4,5,10, and further references therein]. Bar, 100 nm.

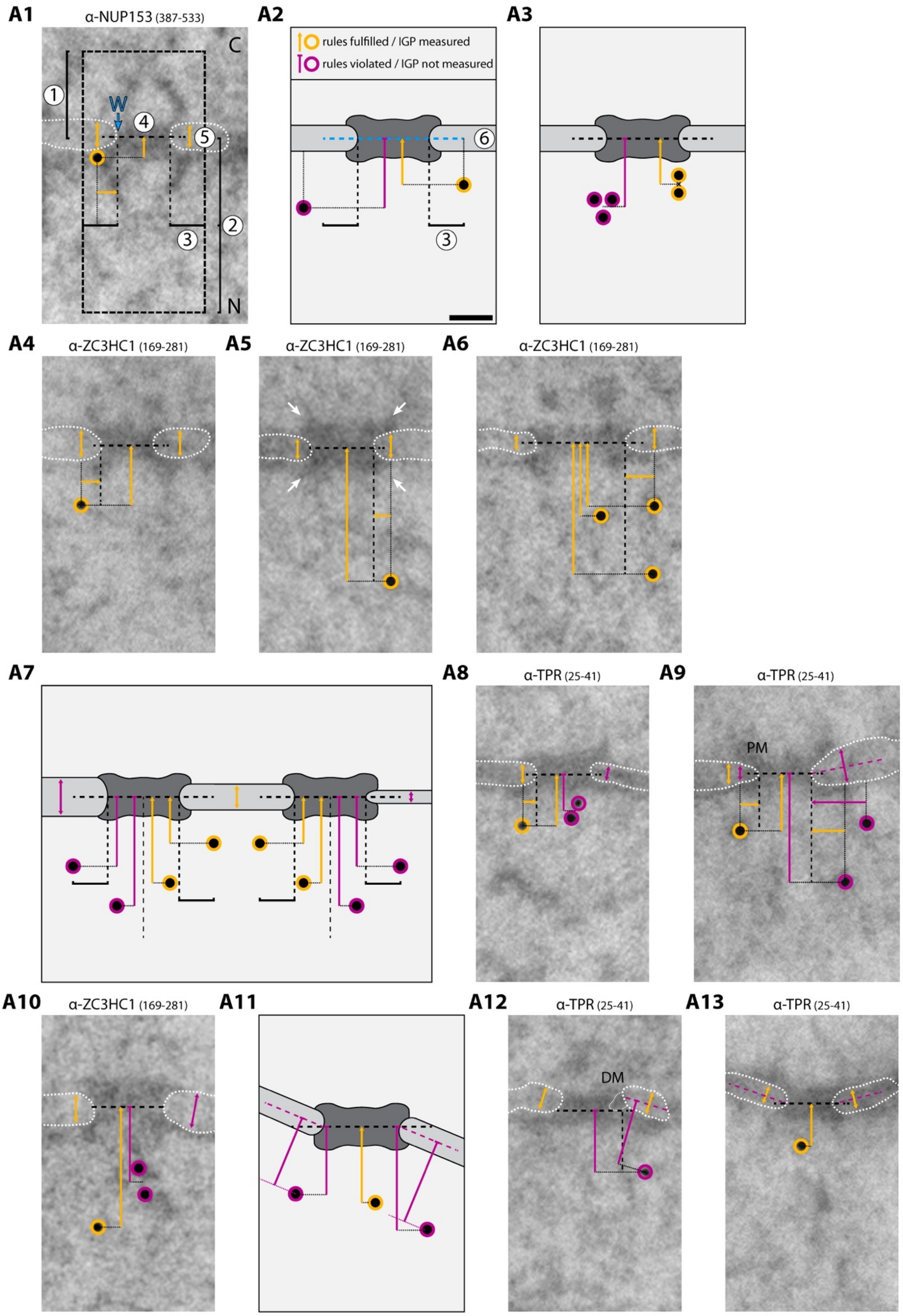
A1
α-NUP153 (387-533)
C
W
1
4
5
2
3
N
A2
rules fulfilled / IGP measured
rules violated / IGP not measured
6
3
A3
A4
α-ZC3HC1 (169-281)
A5
α-ZC3HC1 (169-281)
A6
α-ZC3HC1 (169-281)
A7
A8
α-TPR (25-41)
A9
α-TPR (25-41)
PM
A10
α-ZC3HC1 (169-281)
A11
A12
α-TPR (25-41)
DM
A13
α-TPR (25-41)

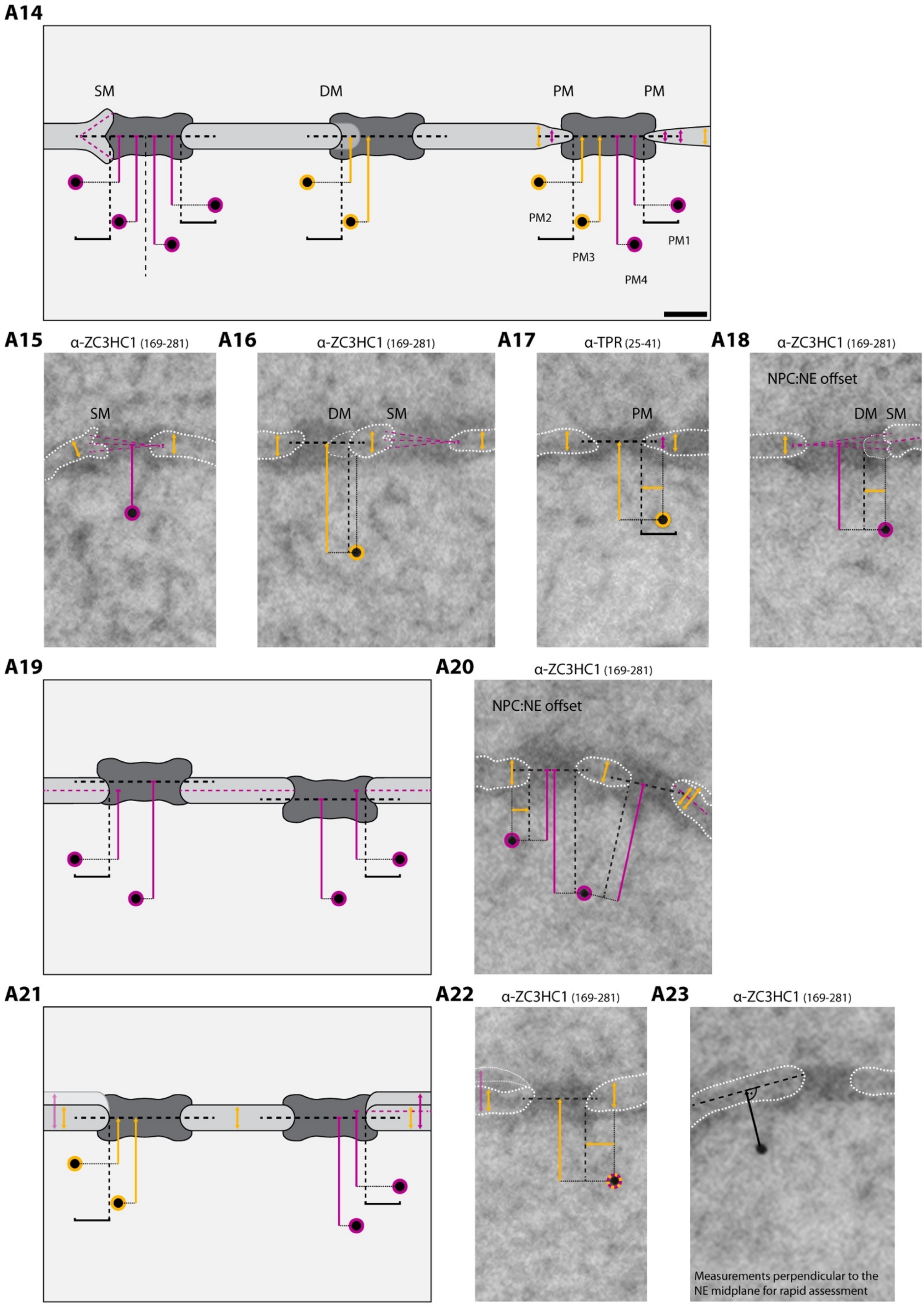
A14
SM
DM
PM
PM
PM2
PM3
PM4
PM1
A15
α-ZC3HC1 (169-281)
SM
A16
α-ZC3HC1 (169-281)
DM
SM
A17
α-TPR (25-41)
PM
A18
α-ZC3HC1 (169-281)
NPC:NE offset
DM
SM
A19
A20
α-ZC3HC1 (169-281)
NPC:NE offset
A21
A22
α-ZC3HC1 (169-281)
A23
α-ZC3HC1 (169-281)
Measurements perpendicular to the
NE midplane for rapid assessment

S06 (2/3)

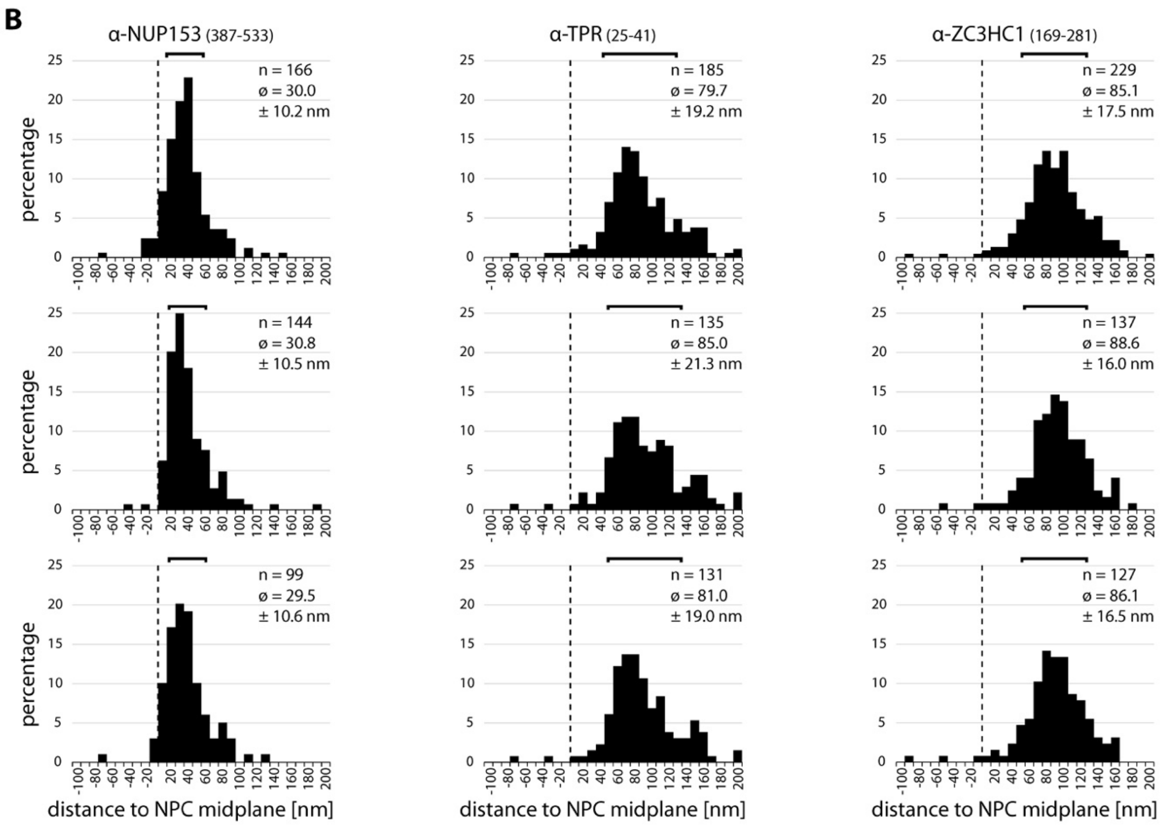
B
α-NUP153 (387-533)
α-TPR (25-41)
α-ZC3HC1 (169-281)
n = 166
ø = 30.0
± 10.2 nm
n = 185
ø = 79.7
± 19.2 nm
n = 229
ø = 85.1
± 17.5 nm
n = 144
ø = 30.8
± 10.5 nm
n = 135
ø = 85.0
± 21.3 nm
n = 137
ø = 88.6
± 16.0 nm
n = 99
ø = 29.5
± 10.6 nm
n = 131
ø = 81.0
± 19.0 nm
n = 127
ø = 86.1
± 16.5 nm
percentage
distance to NPC midplane [nm]

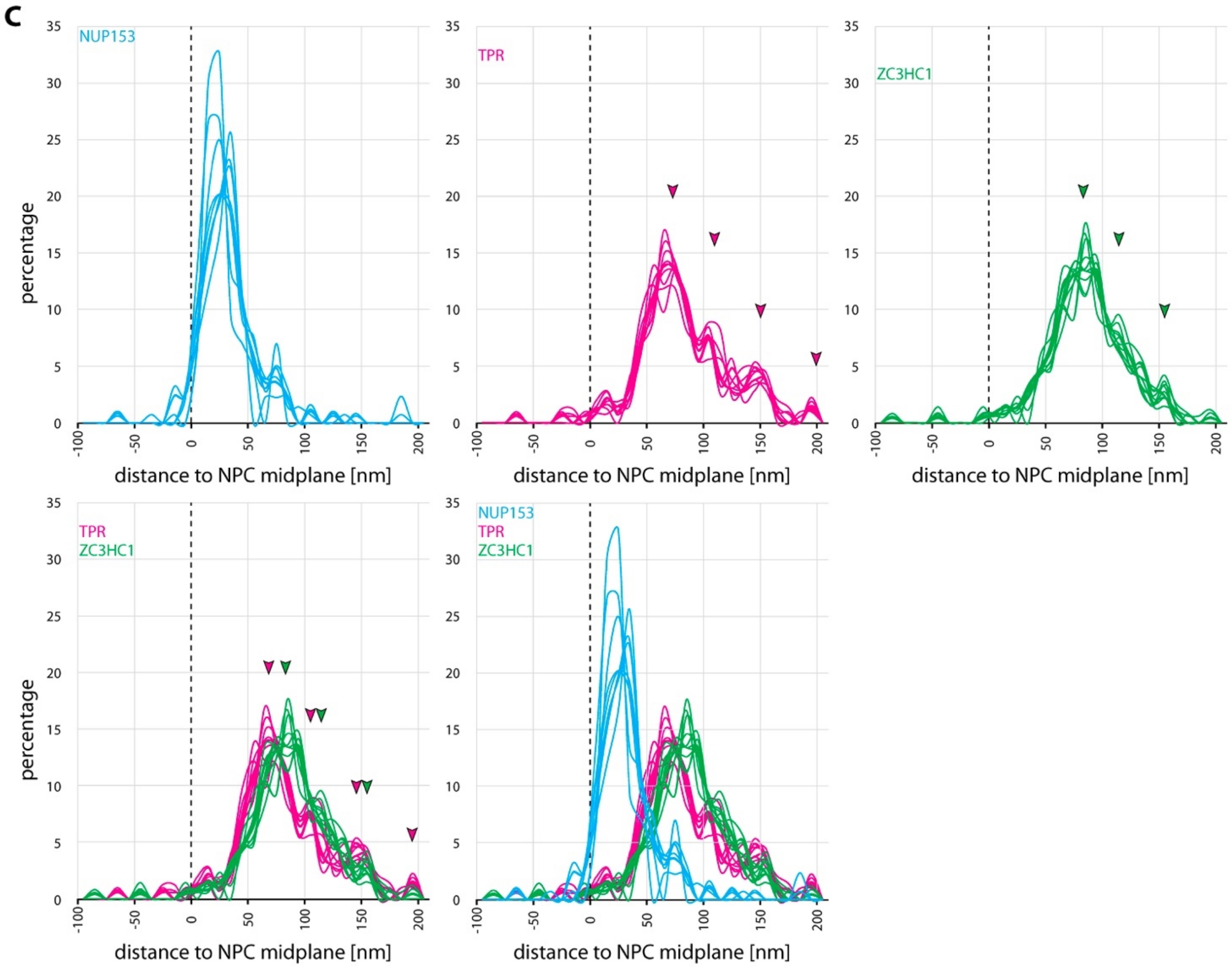
C
NUP153
TPR
ZC3HC1
percentage
distance to NPC midplane [nm]

Supplemental Figure S6. Guidelines for and results of distance measurements in post-embedding iTEM of K4M-embedded oocytes.

In post-embedding iTEM of resin-embedded specimens, the target epitopes need to be exposed on the surface of the ultrathin sections because the depth to which an antibody can penetrate into such a section, if at all and depending on the type of resin, is generally regarded as limited to a few nanometres at most. Thus, even though sectioning through an object of interest can sometimes expose epitopes inaccessible for antibodies in pre-embedding iTEM, it is generally more often the case that the density by which a given target is labelled with IGPs is far lower in post-embedding than in pre-embedding iTEM. Such low IGP density is especially evident when using single epitope-targeting antibodies specific for only one NPC or NB component [e.g., 9]. Even in an ideally sectioned NPC, i.e., when the section plane is perpendicular to the NE midplane but not necessarily perfectly diametric with regard to the NPC in top view, only epitopes corresponding to at most two-eights of the eight-fold rotationally symmetric NB will be exposed on the section surface incubated with antibodies (see also Information SI 5). However, such ideal section planes that allow for exposing a distinct epitope somewhere along two of the eight thin fibres of the NB proper will have to be regarded as rare in reality. This is likely one of the reasons why even some seemingly well-sectioned NPCs often are not labelled with IGPs at all, even when using high-affinity antibodies. The assessment of section quality is further complicated by issues regarding sample contrast in HPF specimens freeze-substituted with hydrophilic resins like K4M; an additional issue outlined in further detail in Information SI 6. Altogether, in the current study, this repeatedly led to a situation in which for one distinct target and its highly specific antibody, hundreds of EM micrographs had to be collected, with even far more sectioned NPCs, for even so obtaining comparatively low numbers of IGPs at nearly ideally sectioned NPCs.

In a project that aimed at determining the positions of various NB and NPC epitopes relative to each other in post-embedding iTEM, with only some of these data presented in the current study, we had realised that collecting sample sizes that we regarded sufficient for being diagnostically conclusive and statistically significant was neither technically nor economically feasible by the available means if only focussing on those IGP-decorated NPCs that we deemed perfectly sectioned and perfectly well heavy metal-stained, and that at the same time were only flanked by NE membranes of flawless, textbook-like appearance. Apart from this, we had to accept that we could not unambiguously distinguish between an NE segment exactly sectioned perpendicularly and others with only roughly perpendicular section planes, deviating from perpendicularity by a few degrees. Therefore, to allow for the acquisition of sufficiently large total sample sizes, and numbers of representative sample subsets that were nonetheless unambiguously evaluable, we had to accept a certain degree of imperfection regarding the section plane and appearance of those sectioned NPCs that were to be further evaluated. In order to ensure an unbiased data evaluation, this required strictly defining which degree of imperfection was still tolerable, in addition to setting up clear rules and strict guidelines for the tasks of immunogold-labelling, image acquisition, and data evaluation, including the distance measurements.

First, only antibodies performing sufficiently well in IFM of ultrathin sections of the same resin-embedded specimen were used for iTEM, as outlined in Information SI 5 and Figure S5. Next, these antibodies were further evaluated via iTEM. This included one person taking overlapping pictures along the entire length of the visible cross-sectioned NE that was to be imaged, irrespective of where an IGP was visible in the field of view. This meant that even if the only IGP visible was hundreds of nanometres away from the nearest NE segment, and even if there was no IGP to be seen in a field of view at all, still an image was taken. This allowed for assessing mean labelling densities along the NE and per visibly cross-sectioned NPC. The distances of each of all these IGPs to the midplane of the nearest cross-sectioned NE segment were then roughly measured to provide, in addition to the IFM data, a rough assessment of each antibody's labelling performance and specificity in post-embedding iTEM (for additional remarks in this context, see also S6A23 further below). Next, to avoid bias in the following more detailed evaluation of images and accurate measurements, the hundreds of images per specimen were coded by another person in a manner that assigning them to any antibody was no longer possible. Randomly mixed compilations of about 200 and more of such coded images, from labellings with different antibodies, were then evaluated by yet other persons, applying different levels of stringency for IGP distance measurements, based on the more or less stringent rules outlined further below, and performing all measurements at least twice. Decoding of images only occurred after the measurements had been completed. The values for those IGPs that were then detected to have been measured twice within each round of measurement, because of having been present on initially overlapping NE images, were then averaged, with only their resulting means thus used for a final dataset. Finally, several image compilations were then reshuffled and re-coded again and evaluated via the same procedure once more to assess the degree by which such measurements would reproducibly lead to similar values. The basic rules for how distances of IGPs were to be measured relative to the midplanes of the NPC and NE, some additional conditions that had to be met in order to allow for an IGP to be considered for lower-stringency datasets, and some of the generally valid exclusion criteria, are outlined and exemplified in S6A. The degree of variation between datasets based on either different levels of stringency or from reiterated measurements is then exemplified in S6B and S6C.

(A) IGP selection criteria and rules for measurements of IGP distances relative to the midplanes of the NPC and NE, explained schematically and with some exemplary micrographs that stem from the immunolabellings with the antibodies for NUP153-NT, TPR-NT and ZC3HC1. The NE, including the NPC wall-forming parts and certain NE peculiarities,

including such related to shape and width, are highlighted by different types of dashed white lines in order to facilitate understandability of the selection rules explained in the following. NE contrast was low because OsO_4 had been excluded from specimens to be used for immunolabelling, because *en bloc*-staining with uranyl acetate, as explained further above (e.g., Information SI 6) had yielded only weak to moderate contrast, due to specifics of the freeze-substitution protocol, and because immunolabellings and washes with aqueous solutions had further reduced the specimens' heavy metal contrast.

<u>Basic rules applying to near-perpendicularly sectioned NPCs exhibiting collinearity with the flanking NE:</u>

(A1) IGPs located up to 100 nm away from the perpendicularly sectioned NPC's or NE's midplane on the NPC's or NE's cytoplasmic side (C), and those up to 200 nm away on the nuclear side (N) were included in the datasets presented in the current study (see further below). The distances are marked as ① and ②, with corresponding black brackets drawn to scale. Only very few IGPs were sporadically found beyond these boundaries.

In addition to the abovementioned distances ① and ②, this area was further defined by the distance numbered as ③ in S6A1, which was set to be 40 nm. For all of the more stringent datasets, this represented the maximally acceptable distance between the grain centre of an IGP and the next closest NPC wall (W and blue arrow), along any axis parallel to the NE midplane, as long as this axis was within the area defined by ① and ②. Any IGP located beyond this horizontal boundary was not included in the datasets presented here (see also further below). The rationale for such boundary value was based on the thought that the distance, measured in parallel to the NE midplane, between the (i) NPC wall and (ii) the outer perimeter of the NPC's nuclear ring (NR) or cytoplasmic ring (CR), representing the NPC's outer boundaries in top view, plus the distance between (iii) an NPC or NB-associated epitope targeted by a primary IgG, and (iv) the grain centre of its corresponding IGP, with its ascribed mean diameter of 10 nm, would altogether only rarely exceed 40 nm when primary IgG and grain were merely interconnected via a secondary $F(ab')_2$ fragment. An IGP seen beyond this vertical boundary, without revealing a neighbouring NPC to which it could be assigned, might still have represented an IGP specifically bound to its genuine antigen, as part of an NPC or NB of which most would have then been removed as part of the ultrathin section just preceding the actual immunolabelled one. However, without being unambiguously assignable to a visible NPC, such IGPs were only included in some of the low stringency datasets.

In this context, we also regard the following noteworthy. While representative datasets in the current study comprise only IGPs that had been positioned within a distinct area surrounding a cross-sectioned and visible NPC, additional measurements that had accepted all IGPs next to a cross-sectioned NE, irrespective of whether these had been NPC-assignable or located beyond the vertical boundaries defined by ③, had resulted in IGP distribution patterns for NUP153, TPR and ZC3HC1 in which the positions of the one target's major IGP peak relative to the other targets' main IGP peaks were broadly similar to the here presented ones, as long as a clearly discernible NE midplane as reference point had been a measurement criterion (data not shown).

Another distance that directly affected the dimensions of the area within which IGPs were to be measured and that at the same time represented a selection criterion of its own is the one numbered ④ in S6A1, representing the distance between the two visible membrane walls of a cross-sectioned NPC. For the most stringent datasets, the shortest acceptable length for those NPCs that were non-diametrically sectioned was set to 50 nm, representing a relatively small section of the tube formed by these membranes. The maximal distance, then for the diametrically sectioned vertebrate NPC, which formerly had been set to 80-84 nm [e.g., 9,18], and which was also used for the first series of measurements on *Xenopus* oocyte materials (our unpublished data), was then set to 105 nm for the datasets finally presented in the current study, with this latter value based on cryo-EM data of the vertebrate NPC which only then had become available [65], and which provided an explanation for some of the seemingly unusually large pores in the NE that we had noted till then. An example almost reaching this value of 105 nm is presented in S6A6. Altogether, the distance values ①-④ allowed for creating a rectangular window (example with dashed black lines in S6A1) that surrounded the sectioned NPC. The long side of this rectangle had a fixed length (300 nm), while the short side could vary between 130 nm (2x40 nm plus 50 nm) and up to 185 nm (2x40 nm plus about 105 nm). In this rectangle, the distance between an IGP on the one hand and the NPC or the NE on the other was to be measured as a line parallel to the NPC medial axis, i.e., perpendicularly to the NPC's midplane or the midplane of the flanking NE, provided that the latter was collinear with that of the NPC.

Another distance-related selection criterion, which did not affect the dimensions of the abovementioned rectangle, is the one marked as ⑤. This criterion applied to the width of the NE, i.e., the distance between the inner and outer of its two phospholipid bilayers, just next to a cross-sectioned and IGP-decorated NPC.

In a former post-embedding iTEM study, we had set the width of an idealistic NE to 32 nm [9], followed by normalising IGP coordinates for a certain degree of NE width variations deemed tolerable. Among the reasons for such variations were (i) known artefacts as a consequence of chemical cross-linkers used back then, (ii) section planes not being precisely perpendicular to the NE midplane, and (iii) naturally occurring, i.e., natural variations in NE width. In the current study, though, we did not attempt to normalise for such variations but merely defined different ranges of widths, either more narrow or wider and as such representing either more or less stringent criteria, with categorically no IGPs outside such range to be measured. For the least stringent criteria, a wide range of widths from 20 nm to 40 nm was still tolerated, the latter value also influenced by the reported maximal distance of about 40 nm between the inner and outer of the NE's membranes, as it had been determined by cryo-electron tomography [65]. Thus, while apparent distances

between the two phospholipid bilayers forming the NE, as measurable on the micrographs, were found ranging from less than 15 nm to more than 40 nm, any IGPs located next to NE segments whose seeming width was not within this lowest stringency range of 20 nm to 40 nm, were categorically excluded from datasets discussed in the context of the current study. Actually, for most of the more stringent measurements, in order to further minimise the artificial broadening of the IGP distribution curves that was merely a consequence of not truly perpendicular section planes, we delimited the range to 26-38 nm; with the most stringent dataset only allowing for a range of 28-36 nm. Examples of such NE width variations are presented in S6A4-6.

While we regarded some of the less extreme width variations to reflect subtle ones indeed naturally occurring *in vivo*, we considered it also possible that some NE shrinkage and inflation might have occurred in the course of the freeze-substitution process, even though we had systematically screened various iTEM-compatible freeze-substitution conditions for such that would preserve the oocyte's natural appearance of the NE as much as possible. Apart from these explanations, though, which relate to true differences in NE width, much of the seeming NE width variations along the course of the sectioned NE was likely to simply reflect the result of the NE not being indeed perpendicularly sectioned all along its entire length. Such non-perpendicularity of sections was sometimes not detectable by merely inspecting a single micrograph, which also had to do with the abovementioned non-uniform NE staining with UA (see also Information SI 6), and was only evident upon analysis of consecutive sections or of tilt series of images from the same section. However, this kind of evaluation was not feasible for the large number of sectioned NPCs that had to be inspected in this study.

In most of the micrographs and schemes presented in S6A, the acceptable NEs are marked by double-headed arrows in yellow, while several kinds of deviations from an ideally sectioned NPC and NE, as shown further below, are marked by the same type of symbol in purple instead (see also S6A7 and further below), when treated as non-acceptable for datasets of higher stringency. Accordingly, examples of IGPs encircled in yellow are such whose values were still included in the more stringent datasets while those encircled in purple were omitted because of being located at NPCs or NEs that violated one or the other of the rules that needed to be complied with, as further described in the following.

(A2) The criteria ①-⑤, as outlined in S6A1, were complemented by the rule that, in order to rate an IGP-labelled NPC as complying with higher stringency criteria, the NPC midplane not only had to be unambiguously definable but also had to intersect the NPC's two visible membrane walls at or near the point where the diameter of the pore formed by the fused inner and outer NE membrane is smallest. In the following, this point is referred to as the turning point of the pore-forming NE membranes as seen in a cross-section. This, in turn, also meant IGPs were omitted from the more stringent datasets when either one or the other of the cut membrane walls flanking the sectioned NPC was not clearly discernible.

Another selection criterion for higher stringency datasets was collinearity of the cross-sectioned NPCs midplane and the midplanes of those parts of the NE that were directly flanking the sectioned NPC on both of its sides within the boundaries defined by ③, with such collinearity here marked as ⑥. To avoid information overload in S6A1, the common midplane of the schematically depicted NPC and its flanking NE, and the minimal outreach of the collinear midplane, are shown as a dashed horizontal line in blue, next to an exemplary IGP at the boundary defined by rule ③.

(A3) Further simple rules regarded the handling of rare IGP clusters, in which two or more grains were located next to each other closer than 25 nm between two grain centres. These were treated as bound to only a single target epitope, as such clusters were likely the consequence of not every IGP in the labelling solution actually having been singular, an observation not uncommon in immuno-EM. In fact, following the standard processes of coating the colloidal gold grains with antibodies, the subsequent quenching of the beads with other proteins, and the differential centrifugation steps then conducted for obtaining populations enriched in singular IGPs, minor numbers of IGPs therein can still occur as clusters of two or more gold grains. The latter might be bridged by the antibodies themselves, in the current study $F(ab')_2$ fragments capable of spanning up to 15 nm, or by the proteins used for the quenching process.

As schematically illustrated, we considered sporadic clusters consisting of two IGPs, provided all other rules were met, for some of the differently stringent datasets by including the mean of the two grains' distances as a single value. Note that this would also have held true for the two-grain cluster seen in S6A8 further below if this cluster would not have come along with a violation of the NE width rule. By contrast, IGPs of very rare clusters consisting of three or more grains were categorically not included in any of the measurements presented in the current study, irrespective of whether all other rules might have been met. On the other hand, when an NPC was labelled with two or more grains that were further apart than 25 nm, like in S6A6, each of these IGP distances was treated as a separate value.

(A4-A6) Examples of NE width variations commonly tolerated for here presented datasets. The positions of the NPC's cross-sectioned NR and CR are marked in S6A5 by white arrows. The positions at which NE width measurements were usually conducted, namely next to the membrane's NPC-adjacent arch where the cytoplasmic and nuclear parts of the NE membranes were, in the ideal situation, again parallel to each other (for exceptions, see below), are once again marked by the double-headed arrows in yellow.

Further rules applying to deviations from the norm:

(A7-A10) While one would expect an ideally cross-sectioned NPC to be flanked on both sides by sectioned NEs of very similar appearance, this was not always the case. Instead, the widths often differed to some extent, sometimes resulting in a situation in which the NE at the one side of the NPC met the NE width rules whilst the other did not. Therefore, we defined further rules for the evaluation of those IGPs that were located next to such NPCs and applied these in the context of lower and higher stringency measurements. These rules are illustrated in the scheme in S6A7 and exemplified by the micrographs in S6A8 to S6A10.

For those IGPs not positioned directly beneath the part of the NPC proper that was flanked by the NPC wall, but located beneath the NE instead, while still fulfilling the rule ③ regarding the IGP-NPC wall distance (see above), the NE width that was regarded as decisive, between a stretch of the inner and outer membrane arranged in parallel, was the one just next to the IGP. This meant that for some of the low-stringency measurements, such IGPs were accepted whose neighbouring NE had a width within the tolerable range, here illustrated by the four yellow encircled IGPs in the centre of the scheme, even though the NE width at the other side of the NPC did not. An IGP located next to the latter, here illustrated in purple on either the left or right side, would then still have been categorically excluded, either because the NE at this side would have been too narrow, as exemplified in S6A8, or too wide, as in S6A9 and S6A10. In cases in which the IGPs were located beneath the NPC proper, as illustrated in the middle of the scheme, the NE widths were measured at the approximate position of the outer perimeter of the NPC's membrane-adjacent NR and CR, that were often detectable due to their more electron-dense appearance. In those cases in which one of the widths did not meet the selection criteria, it was the width of the NE closest to the IGP that determined whether the IGP was to be considered or not.

Further rules regarded the handling of IGPs located next to NEs whose nuclear and cytoplasmic membranes were not parallel to each other. For example, when the NE still met the width rule at precisely this position but appeared notably bloated just beyond this site, thereby then clearly violating the NE width rule, the corresponding IGP was not considered for the current datasets. By contrast, in the case of "pointed-membrane" NEs (see further below), at which IGPs could also be located next to an NE whose cytoplasmic and nuclear membrane were not in parallel to each other, yet further rules applied (see further below).

(A11-A13) Furthermore, we noted that the midplanes of the NPC proper and the flanking NEs were often not collinear, either with regard to one or both sides of the NPC, even if the membranes were flanking a cross-sectioned NPC of prototypic appearance. Even in this latter case, we regarded lack of collinearity between the NPC midplane and the midplane of the neighbouring NE as a general exclusion criterion for any higher-stringency dataset when the IGP in question was not positioned perpendicularly above or beneath those parts of the NPC proper that were flanked by the NPC's membrane walls, but instead further away from the NPC, resulting in a location above or beneath the one or other NPC-flanking NE segment, and with this then coming along with the latter's midplane having a notable tilt angle relative to the adjacent NPC's midplane. This situation is here illustrated on the scheme's left and right side and also exemplified by the micrograph S6A12, with the latter, however, not only exemplifying the violation of the collinearity rule but also the so-called NPC:NE offset rule, explained further below. In addition, S6A12 also exemplifies the here non-excluding "double-membrane" (DM) rule, explained further below as well.

However, when IGPs were located above or beneath an NPC with a clearly definable midplane that intersected the flanking membrane walls at their turning point, as illustrated in the middle, non-collinearity between the NPC's midplane and those of the flanking NEs was not an exclusion criterion, as also exemplified by the micrograph in S6A13.

(A14-A18) Further rules applied to NE peculiarities occasionally observed in all of the datasets evaluated in the current study. Among these were cross-sections of the NPC's membrane wall that did not appear normally curved but exhibited odd shapes. We could not tell for sure how the one or other of these observed peculiarities had come about, whether they represented artefacts of the freeze-substitution process or some optical illusions of the ultrathin-sectioned material, but we had to decide how to handle IGPs located next to such NPCs.

In some cases, the NE appeared to be split into two small arches, a feature that we termed the "split membrane" (SM)-type of an NPC wall. This situation is illustrated on the scheme's left side and also exemplified by the micrograph S6A15, S6A16, and S6A18, with the latter representing a combination of two NE wall peculiarities, as explained further below. IGPs located next to an SM-type NPC wall were categorically excluded from being considered for any of the datasets of higher stringency, even when all other rules had been met, with the underlying rationale being that the NPC midplane would not be sufficiently strictly definable without the turning point of a single-arched NPC wall.

In other cases, the arch of the actual NPC wall of normal appearance seemed to be accompanied by a second, yet mostly fainter arch, located closer to the NPC medial axis, with this second arch being reminiscent of an NPC wall that had been superimposed onto the more electron-dense material of the NPC proper; a feature that we termed the "double membrane" (DM)-type. Illustrated at the scheme's central NPC, it is also exemplified by the micrographs S6A16 and S6A18, with the latter representing a combination of the DM and SM feature and of the NPC:NE offset rule explained further below. In such a case, we treated the SM criterion, or such an offset feature, as dominant, again resulting in the exclusion of the corresponding IGP. In general, we treated an IGP located next to a DM-type of NPC wall as measurable for datasets of moderate to higher stringency, as long as the NPC's other features complied with the other rules and as

long as the second arch had a notably fainter appearance than the main wall, which then nonetheless still had to comply with the criterion ④ rule for a non-diametric NPC section width of at least 50 nm.

Another NE peculiarity occasionally observed was the situation in which the sectioned NPC wall was not looking like an arch but had a somewhat more pointed appearance, which we termed the "pointed membrane" (PM)-type, illustrated at the scheme's right side and exemplified by the micrograph in S6A17. Like for the DM-type of NPC walls, we also treated an IGP located next to a PM-type of NPC wall as, in principle, measurable for moderate to higher stringency datasets, provided that the other rules were met. However, depending on the IGP's position relative to the NPC and the NE, yet further rules needed to be considered. For example, an IGP located beneath a pointed NE that did not reach the minimal NE width at least at the measurement boundary defined by criterion ③, illustrated in the scheme by scenario PM-1, was categorically not considered. On the other hand, as long as the NE width, gradually increasing from its tip to the criterion ③-defined boundary, met the width criterion ⑤ at this boundary and had reached parallelism of the cytoplasmic and nuclear NE membranes, a corresponding IGP was regarded measurable, here illustrated by PM-2 and exemplified in S6A9.

In those cases, in which the IGP was located perpendicular to the midplane of the sectioned NPC, but neither the one nor the other flanking NE segment met the width criteria at the criterion ③-defined boundary, such an IGP was again categorically excluded (not illustrated). On the other hand, if one of the flanking NEs met the width criteria, it depended on whether the IGP was positioned closer to the permissible or non-permissible NE, in order to be regarded measurable for a lower stringency dataset or not at all, here illustrated by PM-3 and PM-4 respectively.

(A19-A20) IGPs were not considered for the higher stringency datasets when there appeared to be an apparent offset between the corresponding NPC and its flanking NE segments or when an offset could not be excluded unambiguously, as illustrated in S6A19 and exemplified in S6A20. In addition, a potential offset could also be noted in S6A12, and to some extent, also in S6A10, S6A18 and S6A22. Even in those cases in which NPC and NE midplanes were still in parallel to each other, they were then no longer collinear, with the NPC instead appearing displaced relative to the NE's midplane, towards the NE's one or other side, perhaps reflecting some effect of non-perpendicular sectioning. Sometimes, this even came along with either the sectioned NPC's CR or the NR, or parts thereof, no longer being visible in the micrographs.

<u>Further potential distortions of the NE:</u>

(A21-A22). In some cases, the position of the cytoplasmic membrane of the NE was ambiguous because of two bands of membrane-like appearance occurring in parallel to each other, as illustrated in S6A21 and exemplified in S6A22, and also visible in S6A20. As long as one of these bands was notably weaker than the other, the NE width rule was applied to the more dominant one. This allowed for measurement between the latter and the NE's inner membrane (yellow double-headed arrow on the left) that yielded a distance value that in principle was regarded admissive for a low stringency dataset while ignoring the other distance (light purple-coloured double-headed arrow). By contrast, in cases in which both membranes appeared similarly pronounced, as illustrated on the right side, the corresponding IGP-decorated NPCs were not considered for any of the current study's datasets.

Furthermore, note that the IGP in S6A22, shown encircled in dashed yellow and purple, represents a typical example for those grains whose assignment to a distinct dataset of only low or moderately higher stringency was not always unequivocal, representing a fluent transition between the different categories of stringency and reflecting some variation in the rigorousness of rule application in the course of the iteratively conducted evaluations of the coded datasets (in this context, also see S6C).

<u>Initial assessment measurements:</u>

(A23) For rapid measurements, performed as part of the initial assessment of antibody performance prior to the above-described more detailed evaluation of then coded image compilations, only a few rules were applied. First, the distance between any IGP and the closest NE segment was to be measured, irrespective of whether a cross-sectioned NPC was visible at this position or not, as long as this part of the NE did not merely represent a grazing or low-angle cross-section but had been transversely sectioned, yet not necessarily perpendicularly, at an angle that allowed for recognising the imaged NE as the boundary between the cytoplasm and nuclear interior. Central to these measurements though was the strict perpendicularity (here indicated as a right angle with dot) of the distance vector to the closest midplane through either the NE or an NPC, often resulting in situations in which a neighbouring NPC's midplane was ignored, as exemplified in this micrograph. When not applying any NE width or other selection criteria, such crude measurements, while allowing for confirming an antibody's suitability for iTEM, were found to result in IGP distribution patterns that were notably broader than those that stemmed from the more rule-based evaluations. Bars in S6A2 and S6A14, 50 nm, same magnification for all micrographs in S6A.

(B) Examples of variations in datasets of distance measurements of IGPs relative to a reference point in post-embedding iTEM. The results, representing the distances of IGPs relative to the NE or NPC midplane (dashed vertical line), stem from three different measurements at different points in time, all based on rules outlined in S6A, but reflecting an increase in stringency in the IGP selection from the first row to the third row. The data were obtained from the same set of images that had been coded and shuffled before the analysis and only decoded again after having completed all three different

measurement processes. The histograms include all IGPs that were detected up to 100 nm and 200 nm away from the cross-sectioned NPCs at their cytoplasmic (negative values) and nuclear sides (positive values) and that had complied with the rules for each stringency level, with each bar representing the percentage of IGPs located within a window of 10 nm width and with n standing for the number of gold grains per dataset. Provided values of mean distances and SD values are based on the middle 68% of all measured distance values for each target (contingents demarked by brackets). Note that ZC3HC1 and TPR-NT IGPs were always found enriched in a similar region at the nuclear side of the NPC, irrespective of the mode of measurement and that the distribution peak for the ZC3HC1 IGPs was always slightly further away from the NPC-NE midplane than the corresponding peak for TPR-NT. The mean values might overvalue to some extent the distance of each protein's first main peak from the NPC's midplane because additional subpopulations of nuclear ZC3HC1 and TPR appeared located further away from the NE, with their IGP distributions partially overlapping with those representing the prototypic NB. In addition, the width of the main bell curves also needs to be interpreted with some caution for other reasons mentioned in Information SI 6. Further note that even for the antibodies targeting the NUP153 segment 387-533, there were IGPs decorating sites further away from the NPC proper. Also, note that the bar diagrams shown in the central Figure 2D present the mean of the percentage values for the corresponding distance windows of these three measurements for each antigen. Further measurements on these images, re-coded again for such purpose, and on other compilations of coded images of specimens labelled with the same antibodies, then, though, applying only one or the other level of stringency, did not result in any major deviations from the findings presented here.

(C) Line diagrams corresponding in part to the data presented in S6B but illustrating these results in a different manner. Each thin line reflects a dataset acquired by either applying differently stringent criteria regarding IGP selection and measurement, or iterations of measurements on compilations of re-coded images that have been conducted at different time points, in order to assess the degree of data reproducibility in the course of independent measurements, here representing 12 datasets each per target. The graphs for NUP153, TPR and ZC3HC1 are presented in blue, magenta and green, respectively. Note that the different measurements conducted for a given target yielded similar line profiles.

In addition to each protein's major IGP peak, essentially all measurements pointed at further populations of IGPs for TPR, ZC3HC1 and NUP153 located farther away from the NPC. Since these additional peaks represented the sum of IGPs detected at numerous cross-sectioned NPCs, there are, in principle, two main explanations for the gradual decline in IGP numbers per peak, the further these are located away from the NPC. First, the decline might reflect a gradual diminishment of TPR and ZC3HC1 copy numbers at generally every NPC. Alternatively, only a few NBs in the stage II oocytes might possess such additional TPR and ZC3HC1 polypeptides as part of additional fibrillar arrangements that are appended to the NPC-attached prototypic NB and project deeper into the nuclear interior. We consider this latter scenario the most probable one, as NB-like structures are known to occur appended to the TR of some but not all of the NPC-attached NBs in the *Xenopus* oocyte, as exemplified in Figure S7.

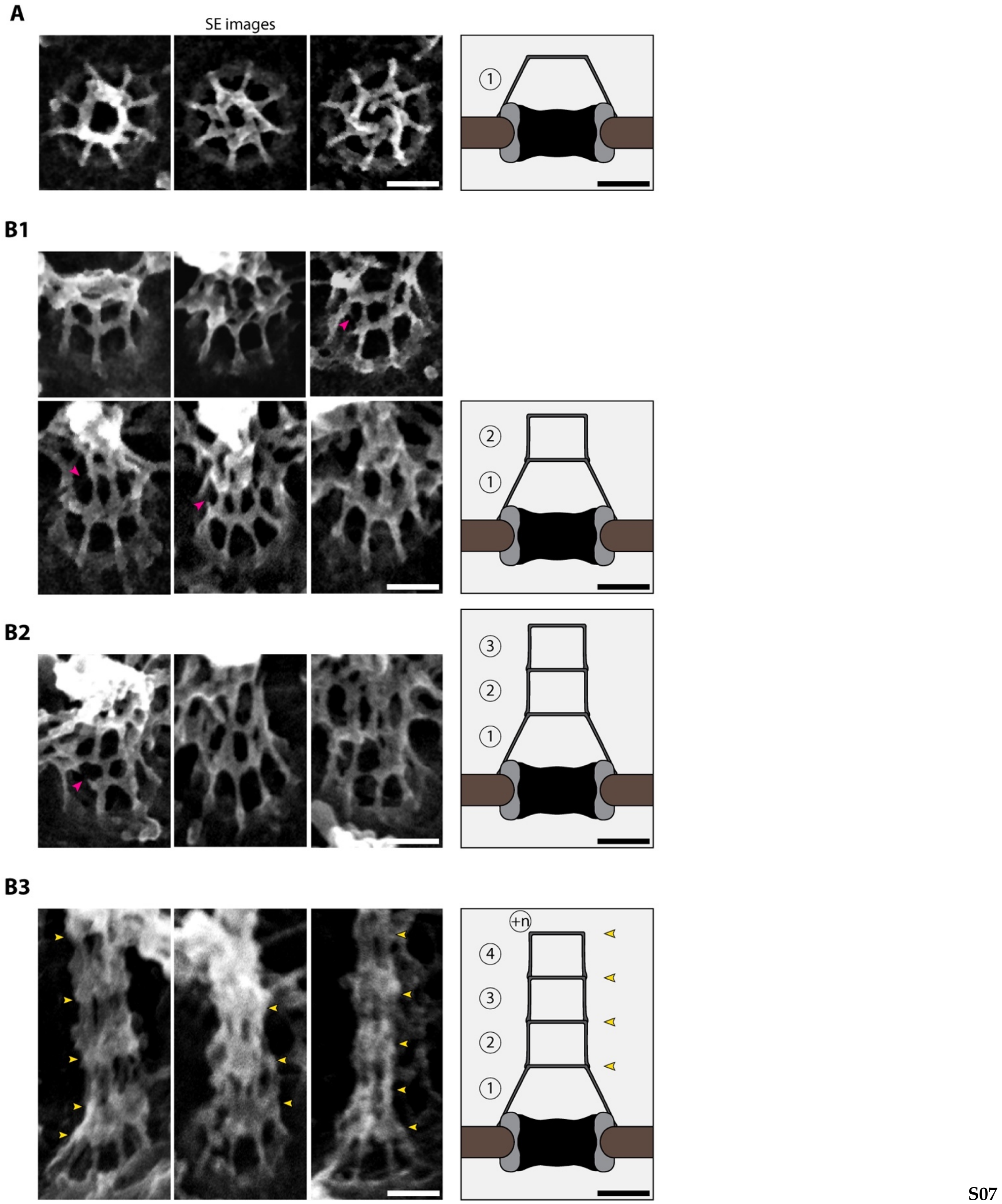

Supplemental Figure S7. Additional NB-like structures stacked on top of the NB proper.

In addition to the NPC-anchored NBs of prototypic appearance, we sometimes found additional fibrillar structures of NB-like appearance appended to the TR, sometimes even stacked one on top of the other, forming cylindrical extensions. Such appendices on top of the NB proper had been reported already in the past. They had been described as cylindrical extensions with regularly transverse fibrils [1], as cylinder arrays of NPC-attached coaxial filaments [4] containing TPR [10], as a cable-like system with periodic structures [3,66,67], as circular arrays of intranuclear filamentous bundles [5],

and as funnel-forming second baskets [68]. Furthermore, we can imagine the NB-attached nuclear envelope lattice (NEL), an NB-attached fibrous sheet with a regular-repeating substructure identified in the oocytes of the newt *Triturus cristatus* [2], to be mostly composed of those proteins that also form the NB-attached cylindrical extensions. These abovementioned arrangements appended to the TR of the NB proper are clearly distinct in appearance from other types of fibrils and fibres occasionally found in contact with some NBs, among which are also actin filaments [e.g., 6,7,28], with actin occurring in vast amounts within the *Xenopus* oocyte's nucleus due to the lack of exportin 6, its nuclear export receptor, which is not expressed in this particular cell type [69].

The following SEM images allow for comparing some variations in the appearance of the NPC-attached NBs with features of the NB-like structures (NBLS) stacked onto them. Even though the current procedure of visualising these structures by SEM neither allows to resolve specific structural details in satisfying detail, and even though this approach of visualising the NB and its appendices does not allow to exclude potential artefacts, like for example, some shrinkage of the NB and the NB-attached cylindrical extensions, which might come about (i) by using cross-linking fixatives like OsO_4, FA and GA, and (ii) by more pronounced beam damage when omitting heavy metal-coating, we regard these SEM data as informative. We interpret them in such a way that these NBLS occasionally seen appended to the prototypic NBs are likely the reason for the observed appearance of additional minor peaks of IGPs for TPR and ZC3HC1 in iTEM of resin-embedded oocytes, with such presumed correlation illustrated in Figure 2G.

(**A** to **B3**) SEM images of individual, NPC-anchored *Xenopus laevis* oocyte NBs (S7A) and of stacked-on NBLS (S7B1-3). The simplified schematic depictions of the NB and of the additional NB-like arrangements on top of the NPC-attached NB's TR correspond to the scheme in Figure 2G.

In S7A, the NPC-attached NBs appeared to lack additional fibrillar structures emanating from the TR. Also, note the often visible bifurcation of the NB fibrils at their terminal end, i.e., in the TR region. Differences in the diameters of the inner perimeters of the TRs shown here might, to some extent, reflect differences in angle sizes by which the fibrils bifurcate, possibly ranging from Y-shaped to almost T-shaped conformations.

In S7B1 and S7B2, additional NB-like entities are shown stacked on top of the NPC-attached NB proper. Magenta-coloured arrowheads point at some fibrils that appeared missing or ruptured. Such absence of one or several of these delicate fibrils often resulted from the lengthy focussing for image acquisition of such NBLS, generally taking more time than for solitary prototypic NBs. During such prolonged irradiation with the electron beam, we often saw fibrils being ruptured in real-time.

In S7B3, note that some NPC-attached NBs even have more NBLS appended to them, stacked one on top of the other. Yellow arrowheads here mark the periodic repetitions of these structures. However, the larger the number of these additional NB-like units, the more difficult it generally was to visualise them by SEM *in toto*, i.e., all together as a cylindrical entity. Among several technical reasons, it was, in particular, the absence of heavy metal-coating, normally allowing for conductivity and the flowing-off of electrons, that had caused rapid surface charging and beam-induced damage of such extended structures. This revealed a drawback of omitting heavy metal-coating as part of the protocol that we had established for iSEM. Bars, 50 nm.

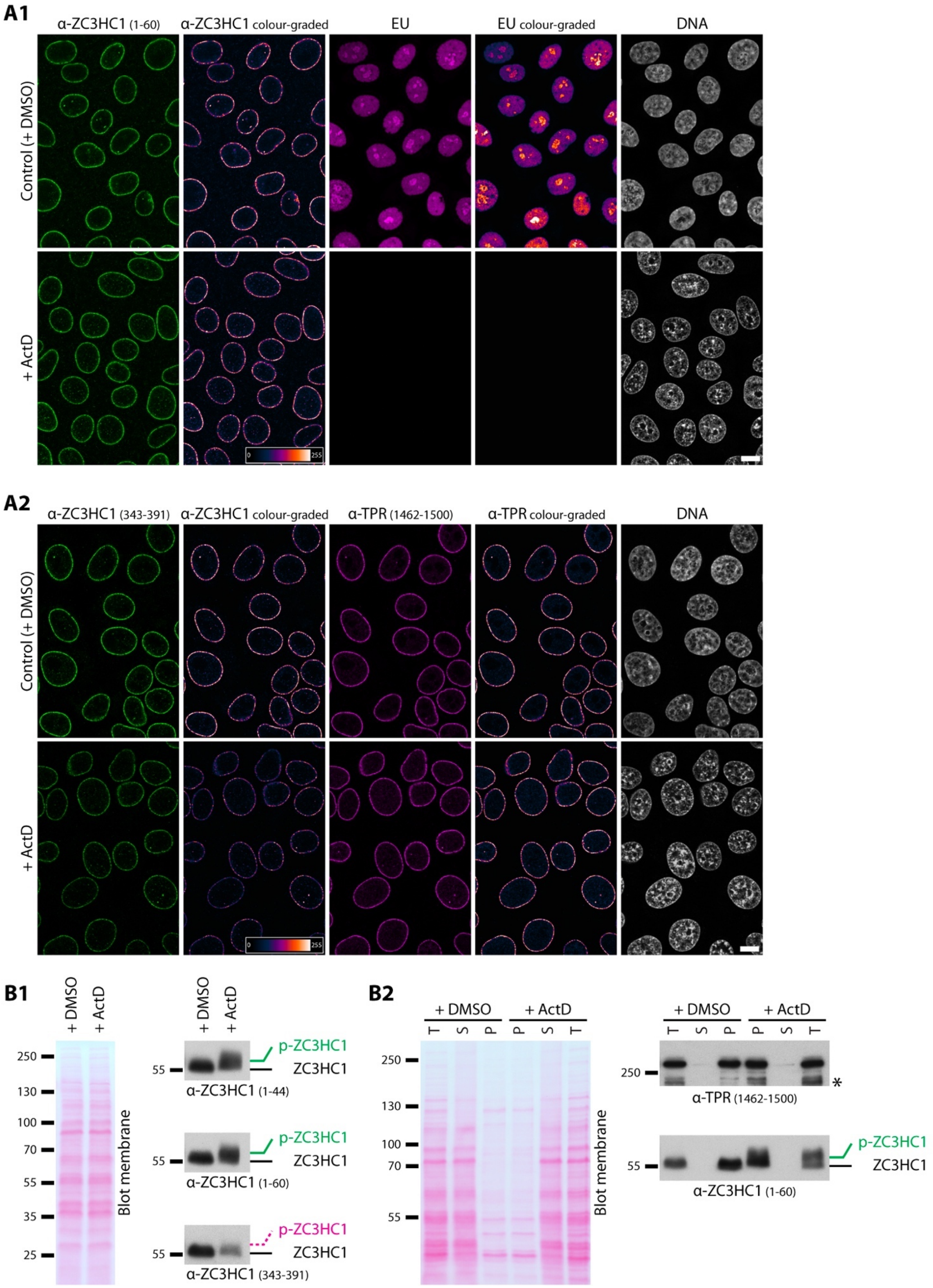
A1
α-ZC3HC1 (1-60)
α-ZC3HC1 colour-graded
EU
EU colour-graded
DNA
Control (+ DMSO)
+ ActD
0
255
A2
α-ZC3HC1 (343-391)
α-ZC3HC1 colour-graded
α-TPR (1462-1500)
α-TPR colour-graded
DNA
Control (+ DMSO)
+ ActD
0
255
B1
+ DMSO
+ ActD
250
130
100
70
55
35
25
Blot membrane
p-ZC3HC1
ZC3HC1
α-ZC3HC1 (1-44)
α-ZC3HC1 (1-60)
α-ZC3HC1 (343-391)
B2
+ DMSO
+ ActD
T S P P S T
250
130
100
70
55
Blot membrane
T S P P S T
α-TPR (1462-1500)
*
α-ZC3HC1 (1-60)
p-ZC3HC1
ZC3HC1

S08

Supplemental Figure S8. IFM and IB of ZC3HC1 upon shutdown of gene transcription in human tumour cells.

For comparing the subcellular ZC3HC1 localisation in transcriptionally active and silent HeLa cells by IFM, cells had been treated with DMSO or with transcription inhibitor Actinomycin D (ActD), the latter inhibiting especially transcription by RNA Pol I but also by RNA Pol II, as well as with 5,6-Dichloro-1-β-D-ribofuranosylbenzimidazole (DRB) for primarily inhibiting RNA Pol II [e.g., 70]. Since the results regarding ZC3HC1 immunolabelling at the NE were similar upon treatment with DRB and ActD, only the latter are presented in the following.

(A) IFM of ZC3HC1 in HeLa cells that had been treated with DMSO (control) or 5 µg/ml ActD for altogether 4 hours. To monitor any residual transcriptional activity within the inhibitor-treated cell populations and to compare this with the controls, cells had been fed, after the first 3 hours of the treatment with either DMSO or ActD, for another hour with then added alkyne-modified ribonucleoside 5-ethynyl uridine (EU), in order to allow for its incorporation into nascent RNAs. This, in turn, was followed by cell fixation and IFM. Specimens were subsequently analysed in parallel, using identical microscope settings. The micrograph of fluorescence of Alexa Fluor 488-conjugated EU in S8A1 is shown colour-converted to magenta while labelling of ZC3HC1 with Cy3-coupled secondary antibodies is shown converted to green, in order to allow for all IFM data for ZC3HC1 in this study to be presented in the same colour. All labellings are also shown colour-graded to display differences in pixel intensities via a colour look-up table. Bars, 10 µm.

(A1) Effective shutdown of transcriptional activity with ActD, also accompanied by some chromatin rearrangements as visualised by DNA staining of the fixed specimens, did not cause any generally noticeable effects on the staining for ZC3HC1 at the NE when using some of the ZC3HC1-specific antibodies, here exemplified by a monoclonal antibody that targets an epitope that we mapped being located between aa 1 and 60 of the 502 aa-long ZC3HC1 protein. When analysing collections of coded images acquired with the same microscope settings and randomly collected from several coverslips with DMSO- and ActD-treated specimens that had been immunolabelled with such a ZC3HC1 antibody, subsequent decoding of such images revealed that it was not possible to sort these images into groups representing only the one or other treatment (data not shown).

(A2) With another subset of ZC3HC1 antibodies, here exemplified by a ZC3HC1 antibody against aa 343-391, with its epitope located within a region predicted to represent an entirely disordered loop of ZC3HC1, the intensity of staining for ZC3HC1 at the NE often appeared moderately reduced within the transcriptionally silenced cells, as compared to the untreated or DMSO-treated cells inspected in parallel. In this case, collections of coded pictures from specimens immunolabelled with such a ZC3HC1 antibody could be sorted into two categories that, after decoding, represented either only the DMSO- or the ActD-treated cells (data not shown). By contrast, immunolabelling of the same cells for TPR, here with an antibody targeting part of its rod domain, revealed no differences between the signal intensities at the control and ActD-treated cells' NEs. Similar results regarding TPR and ZC3HC1 were also obtained when using HCT116 cells instead of HeLa (our unpublished data).

In addition to the abovementioned data, which had indicated that the decrease in NE-associated signal intensity for ZC3HC1, as observed with some but not other ZC3HC1 antibodies, did not reflect a detachment of notable amounts of this protein from the NE, we investigated this issue in further detail by IB. This revealed that treatment with transcription inhibitors and other drugs could result in NE-associated ZC3HC1 polypeptides being phosphorylated at distinct sites, causing some of the epitopes usually recognised by some of the ZC3HC1 antibodies to then be partially masked. While further details related to this topic will need to be presented elsewhere, some of the immunoblots exemplifying such post-translational modification of ZC3HC1 upon treatment with ActD are already shown in the following.

(B) IB of cell fractions obtained from populations of cells that had been treated with DMSO or ActD, revealing that indeed no conspicuous amounts of ZC3HC1, despite its site-specific phosphorylation, were released into a soluble pool after 4 hours of a transcriptional shutdown.

(B1) IB of total extracts from HeLa cells treated with DMSO or ActD for 4 hours. Incubations with three different antibodies for ZC3HC1 were on three stripes of the same membrane, with only one of these stripes here shown stained with Ponceau S, representing replicates of identical loadings of DMSO and ActD extracts into pairs of lanes. Note that while the pattern of Ponceau S-stained proteins did not notably differ between the total extracts of DMSO- and ActD-treated cells, conspicuous amounts of ZC3HC1 were detectable within the extracts of the ActD-treated cells as having been post-translationally modified (green lettering), which subsequently was ascertained to represent phosphorylations at several amino acids (our unpublished data). While two of the ZC3HC1 antibodies against the N-terminus (upper two rows) bound to the non-phosphorylated and the multisite-phosphorylated protein, the latter was barely targeted and thus hardly detectable by a third ZC3HC1 antibody against aa 343-391 (magenta lettering).

(B2) IB of fractions obtained by extraction with TX-100 in NB-s buffer and subsequent centrifugation at 20,000 g, resulting in a supernatant (S) and an LNN-enriched pellet (P) fraction. Total cell proteins (T) were loaded for comparison. Immunolabellings for TPR and ZC3HC1 were performed on the upper and lower halves of the Ponceau S-stained membrane shown here. The asterisk marks a common degradation product of TPR. Note that those versions of ZC3HC1

that had become phosphorylated (green lettering) upon ActD treatment largely remained bound to the pelleted, LNN-enriched materials. Further note that despite such pronounced ActD treatment-induced phosphorylation of ZC3HC1, the TPR polypeptides too remained near-quantitatively associated with the pellet fraction, demonstrating that certain positions of the ZC3HC1 amino acid sequence can be phosphorylated without causing complete disruption of distinct ZC3HC1-TPR interactions.

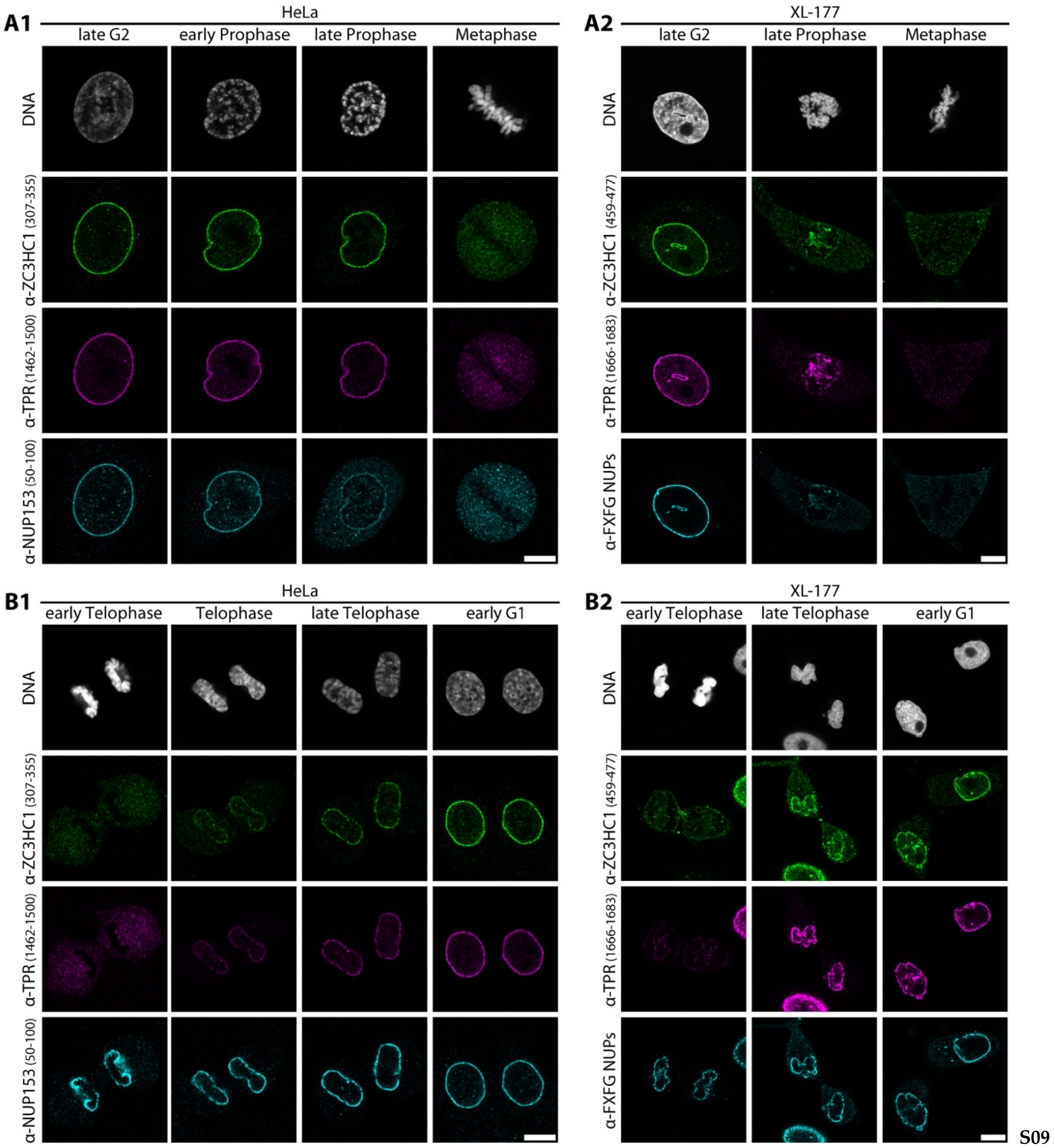

Supplemental Figure S9. Disassembly during entry into mitosis and post-mitotic reassociation of ZC3HC1 and TPR to the newly assembled NE in proliferating cells.

(A) In former studies describing NB disassembly, TPR had been shown to detach from the NE only relatively late in prophase, just prior to or along with NPC scaffold proteins like NUP107. By contrast, other NPC proteins, for example almost all of the NPC-attached NUP98 polypeptides and one of seemingly two populations of NPC-associated NUP153, had been found to be released into solution already much earlier [e.g., 71–73]. In the present study, the detachment of ZC3HC1 from the NE in mitosis was found to occur concomitantly to TPR's release into solution.

(A1 and A2) IFM of HeLa (S9A1) and XL-177 cells (S9A2) in cell cycle stages ranging from late G2 to metaphase. Cells had been triple-labelled with antibodies for either hsTPR or xlTPR and for either hsZC3HC1 or xlZC3HC1, together with antibodies for hsNUP153 or with mAb414 for comparison, the latter reactive with several FXFG-repeat nucleoporins. Note that a major proportion of ZC3HC1, like most of TPR, remained located at the nuclear periphery during NE breakdown

until late in prophase, with this being particularly obvious in HeLa and also other lines of human tumour cells (our unpublished data). By contrast, a relatively larger proportion of NUP153, which in interphase occurs in 32 copies per NPC [14], could already be seen detached from the NPC at this time point, with only smaller amounts having remained located at the NE up to the prophase-to-prometaphase transition. While NUP153 is required for NB formation and has direct and indirect roles in the recruitment and attachment of TPR to those NPCs that are assembled after mitosis and in interphase, respectively [29,71,74–78], its early detachment from the NE in prophase was in line with the majority of NUP153 polypeptides not being required anymore for keeping TPR at the NPC, once it has been anchored to it [78,79,80, and our unpublished data]. Bars, 10 µm.

(B) Former studies had shown that TPR reassociates with NPCs only late towards the end of telophase and early in G1, paralleling the onset of chromatin decondensation [e.g., 71,81,82]. In the present study, reattachment of ZC3HC1 to the newly assembled NPCs was found to occur similarly late and concomitantly to TPR's reassociation.

(B1 and **B2)** IFM of HeLa (S9B1) and XL-177 cells (S9B2) in cell cycle stages ranging from early telophase to early G1. Cells had been triple-labelled like in S9A. Note that reattachment of ZC3HC1 to the NE mostly occurred late in telophase, then largely concomitantly to the reattachment of TPR. By contrast, some of the FXFG-repeat nucleoporins, like NUP153, reassociated with the NE already during late anaphase [see also 71,81,82]. Bars, 10 µm.

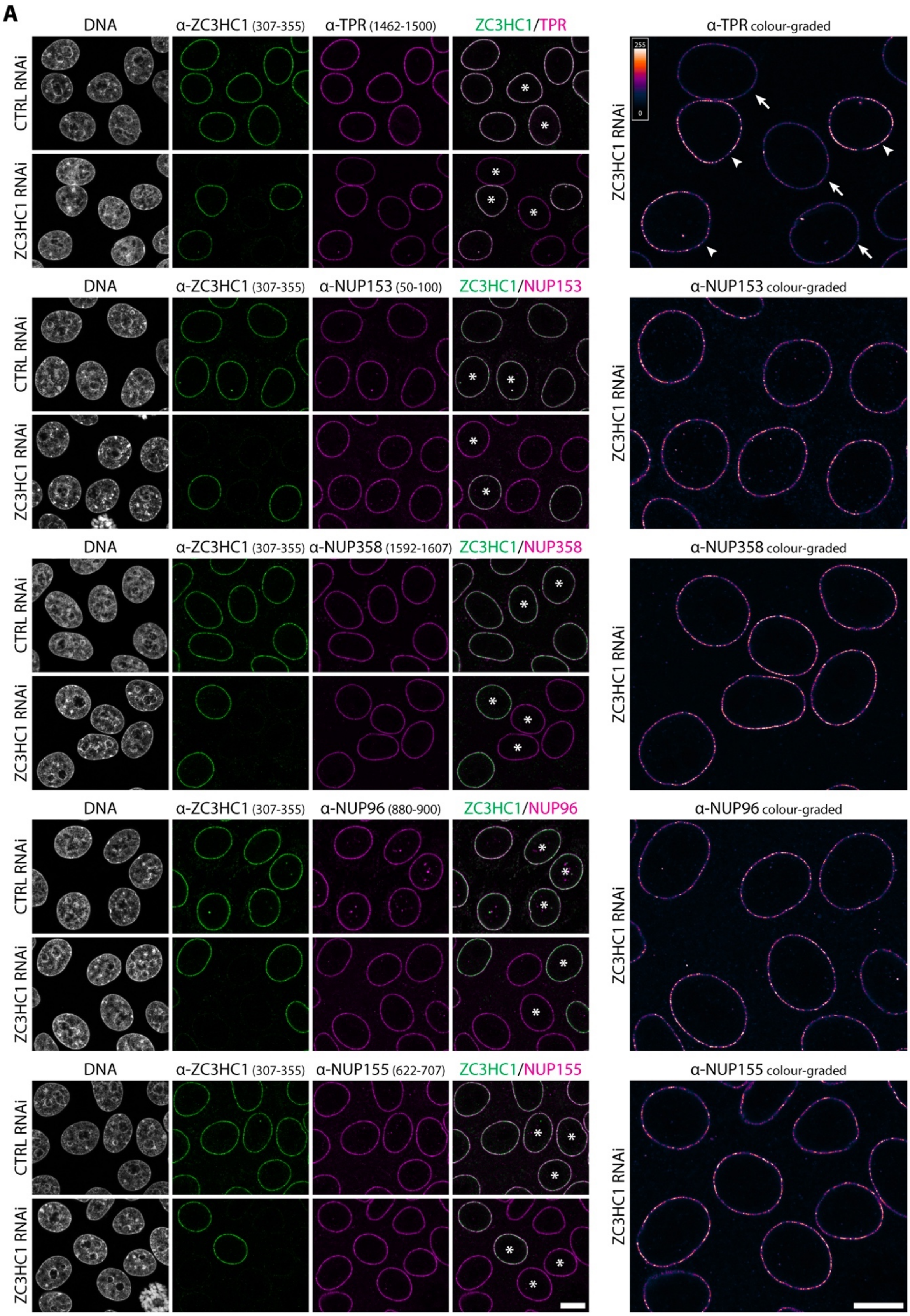
A
DNA
α-ZC3HC1 (307-355)
α-TPR (1462-1500)
ZC3HC1/TPR
α-TPR colour-graded
CTRL RNAi
ZC3HC1 RNAi
255
0
α-NUP153 (50-100)
ZC3HC1/NUP153
α-NUP153 colour-graded
α-NUP358 (1592-1607)
ZC3HC1/NUP358
α-NUP358 colour-graded
α-NUP96 (880-900)
ZC3HC1/NUP96
α-NUP96 colour-graded
α-NUP155 (622-707)
ZC3HC1/NUP155
α-NUP155 colour-graded

S10 (1/2)

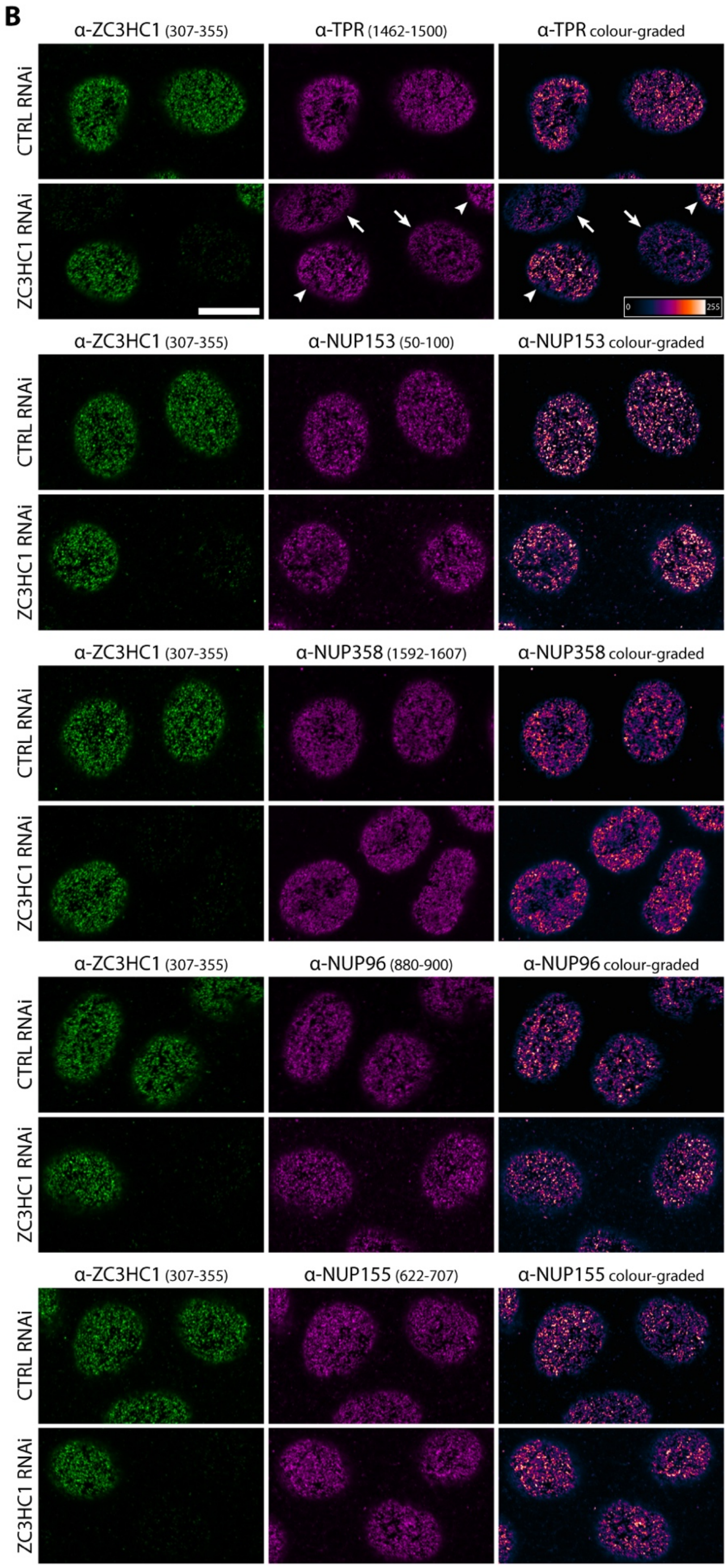

S10 (2/2)

Supplemental Figure S10. IFM of several NPC components in ZC3HC1-deficient HeLa cells.

Cell populations shown here had been transfected with non-target control siRNAs and with siRNAs targeting ZC3HC1. Double-labelling was with antibodies for ZC3HC1 in combination with such for TPR or for an NPC protein

located either at the NPC's cytoplasmic side (NUP358), at its nuclear side (NUP153), on both sides of the NPC as a component of the NPC's nuclear and cytoplasmic ring (NUP96) or within the NPC's inner ring [NUP155; e.g., 83,84]. Cells were treated with TX-100 before fixation to allow for better accessibility of antibodies, especially to epitopes of the inner ring, with all specimens treated in the same manner in order to allow for direct comparison. As an aside, note that such treatment resulted in the loss of the soluble pool of TPR from the ZC3HC1-deficient nuclei.

(A) IFM with the focus on the equatorial plane of the cell nuclei. Pairs or triplets of cells marked with asterisks are shown at a different plane of focus in S10B further below. Some cells that had remained non-transfected within the ZC3HC1 siRNA-transfected populations are shown as reference, having been similarly intensely stained as the cells transfected with control siRNAs (CTRL). In addition to the micrographs showing monochrome immunolabellings of the ZC3HC1 RNAi specimens with TPR and NUP antibodies on the left side, the same images are shown enlarged and colour-graded on the right side in order to display differences in pixel intensities via a colour look-up table. Note that bright NE-staining for NUP358, NUP153, NUP96 and NUP155 did not appear affected by ZC3HC1 deficiency. By contrast, compared to TPR in non-transfected cells (marked by arrowheads), the signal intensity for NE-associated TPR in the ZC3HC1-deficient cells (arrows) appeared reduced by about half. Bars, 10 µm.

(B) IFM with the plane of focus on the cells' nuclear surfaces. Note that the density as well as the intensity of the punctate signals, as seen after labelling with the NUP358, NUP153, NUP96 and NUP155 antibodies and representing NPCs in both the ZC3HC1-positive and ZC3HC1-deficient cells, remained largely unaffected in the absence of ZC3HC1. Accordingly, NPC density having remained unaffected in the ZC3HC1-deficient cells (marked by arrows) was also evident when having labelled them with TPR antibodies and compared them with neighbouring ZC3HC1-positive cells (arrowheads). While it was less obvious in this plane of focus, when merely inspecting the monochromatic images, that ZC3HC1-deficiency was causing a reduction in signal intensities for TPR, this was more evident in the same micrograph's colour-graded version, as shown here too. Bar, 10 µm.

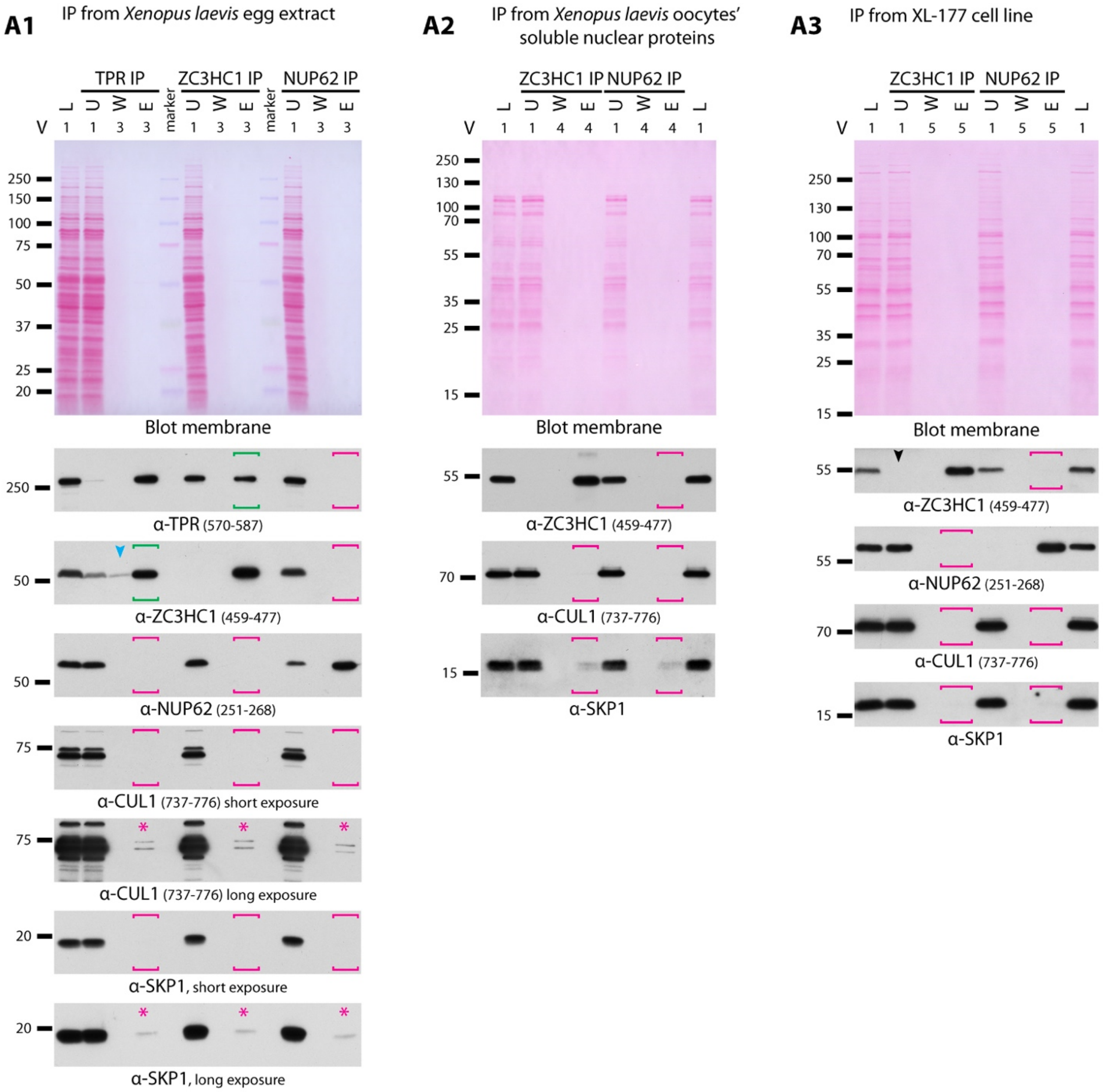
A1
IP from Xenopus laevis egg extract
TPR IP
ZC3HC1 IP
NUP62 IP
L U W E marker U W E marker U W E
V 1 1 3 3 1 3 3 1 3 3
250
150
100
75
50
37
25
20
Blot membrane
α-TPR (570-587)
α-ZC3HC1 (459-477)
α-NUP62 (251-268)
α-CUL1 (737-776) short exposure
α-CUL1 (737-776) long exposure
α-SKP1, short exposure
α-SKP1, long exposure
A2
IP from Xenopus laevis oocytes' soluble nuclear proteins
ZC3HC1 IP
NUP62 IP
L U W E U W E L
V 1 1 4 4 1 4 4 1
250
130
100
70
55
35
25
15
Blot membrane
α-ZC3HC1 (459-477)
α-CUL1 (737-776)
α-SKP1
A3
IP from XL-177 cell line
ZC3HC1 IP
NUP62 IP
L U W E U W E L
V 1 1 5 5 1 5 5 1
250
130
100
70
55
35
25
15
Blot membrane
α-ZC3HC1 (459-477)
α-NUP62 (251-268)
α-CUL1 (737-776)
α-SKP1

S11 (1/9)

.

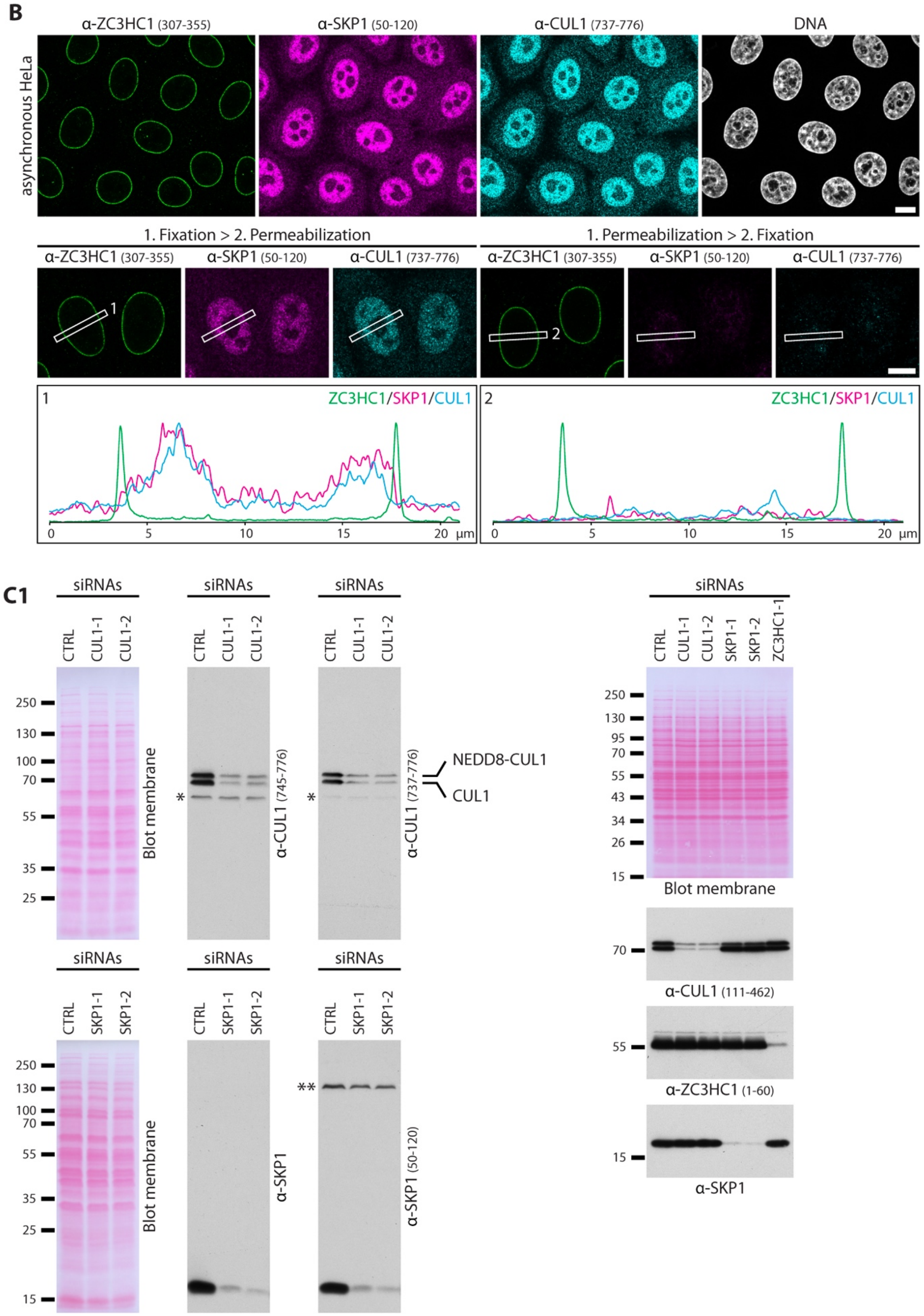

B
α-ZC3HC1 (307-355)
α-SKP1 (50-120)
α-CUL1 (737-776)
DNA
asynchronous HeLa
1. Fixation > 2. Permeabilization
1. Permeabilization > 2. Fixation
α-ZC3HC1 (307-355)
α-SKP1 (50-120)
α-CUL1 (737-776)
ZC3HC1/SKP1/CUL1
0 5 10 15 20 µm
C1
siRNAs
CTRL
CUL1-1
CUL1-2
SKP1-1
SKP1-2
ZC3HC1-1
Blot membrane
α-CUL1 (745-776)
α-CUL1 (737-776)
NEDD8-CUL1
CUL1
α-CUL1 (111-462)
α-ZC3HC1 (1-60)
α-SKP1
α-SKP1 (50-120)
250 130 100 95 70 55 43 35 34 26 25 15

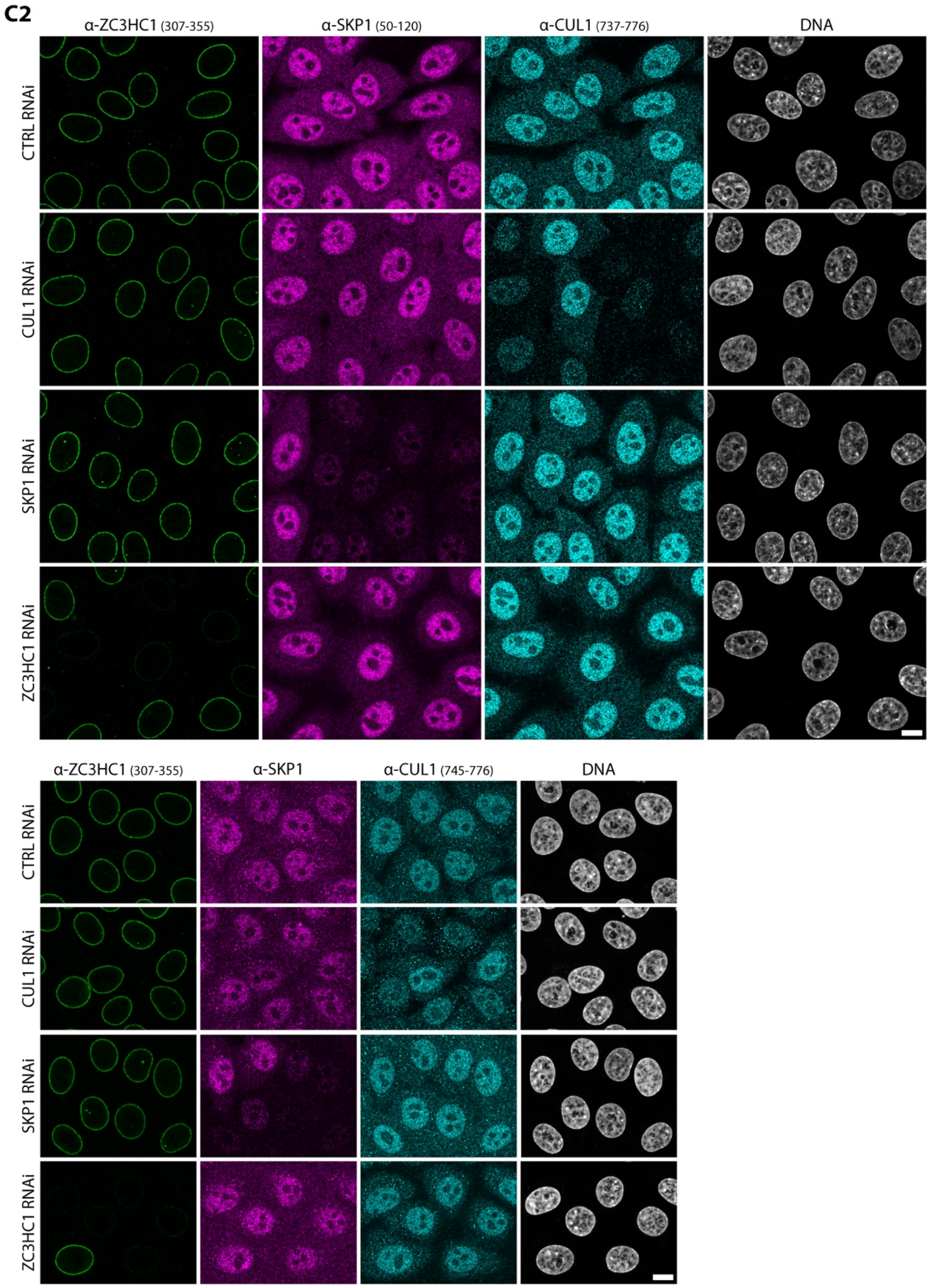
C2
α-ZC3HC1 (307-355)
α-SKP1 (50-120)
α-CUL1 (737-776)
DNA
CTRL RNAi
CUL1 RNAi
SKP1 RNAi
ZC3HC1 RNAi
α-ZC3HC1 (307-355)
α-SKP1
α-CUL1 (745-776)
DNA
CTRL RNAi
CUL1 RNAi
SKP1 RNAi
ZC3HC1 RNAi

S11 (3/9)

D1

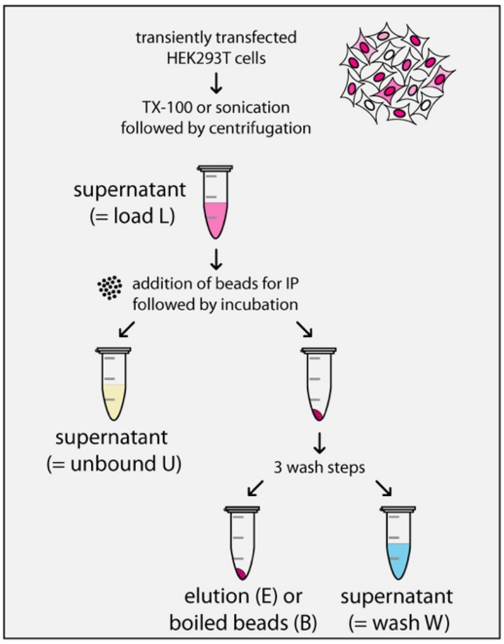

D2 IP with anti-FLAG mAb M2:

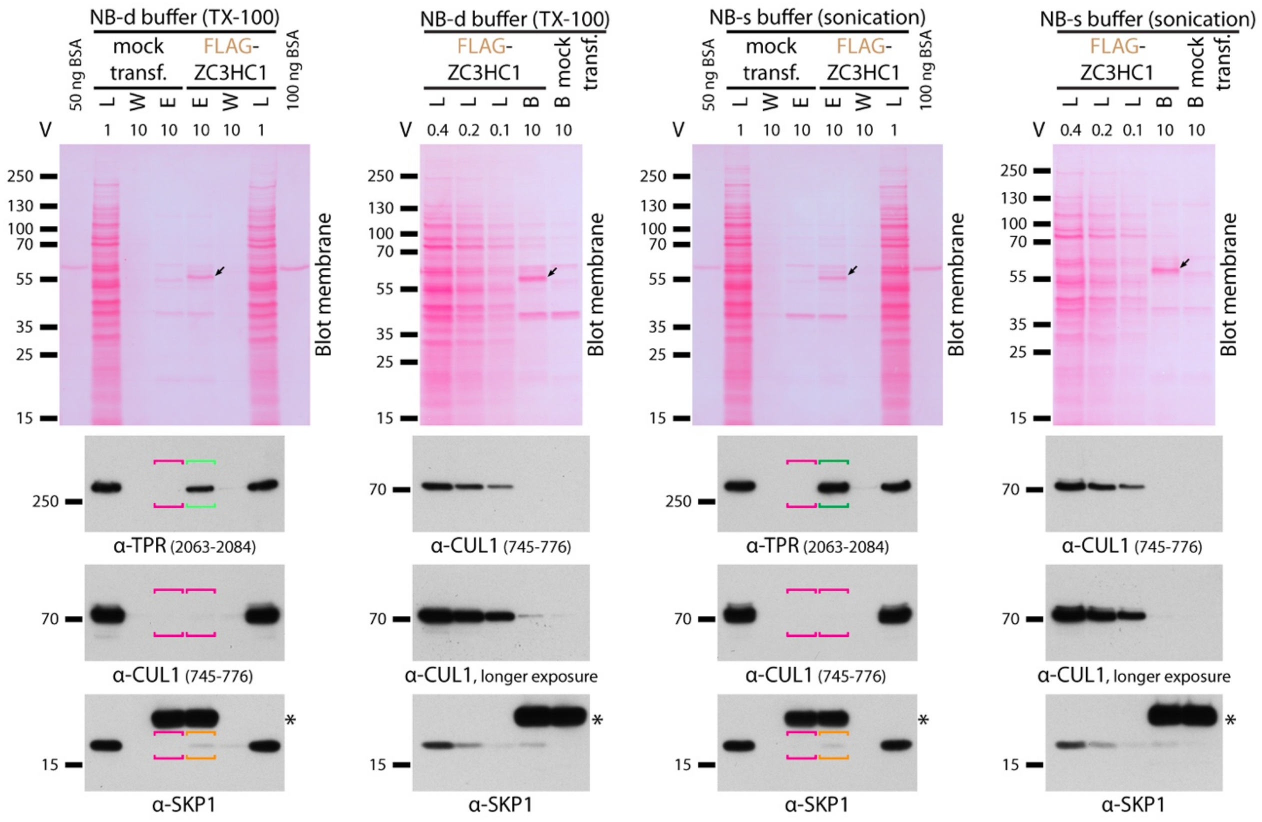

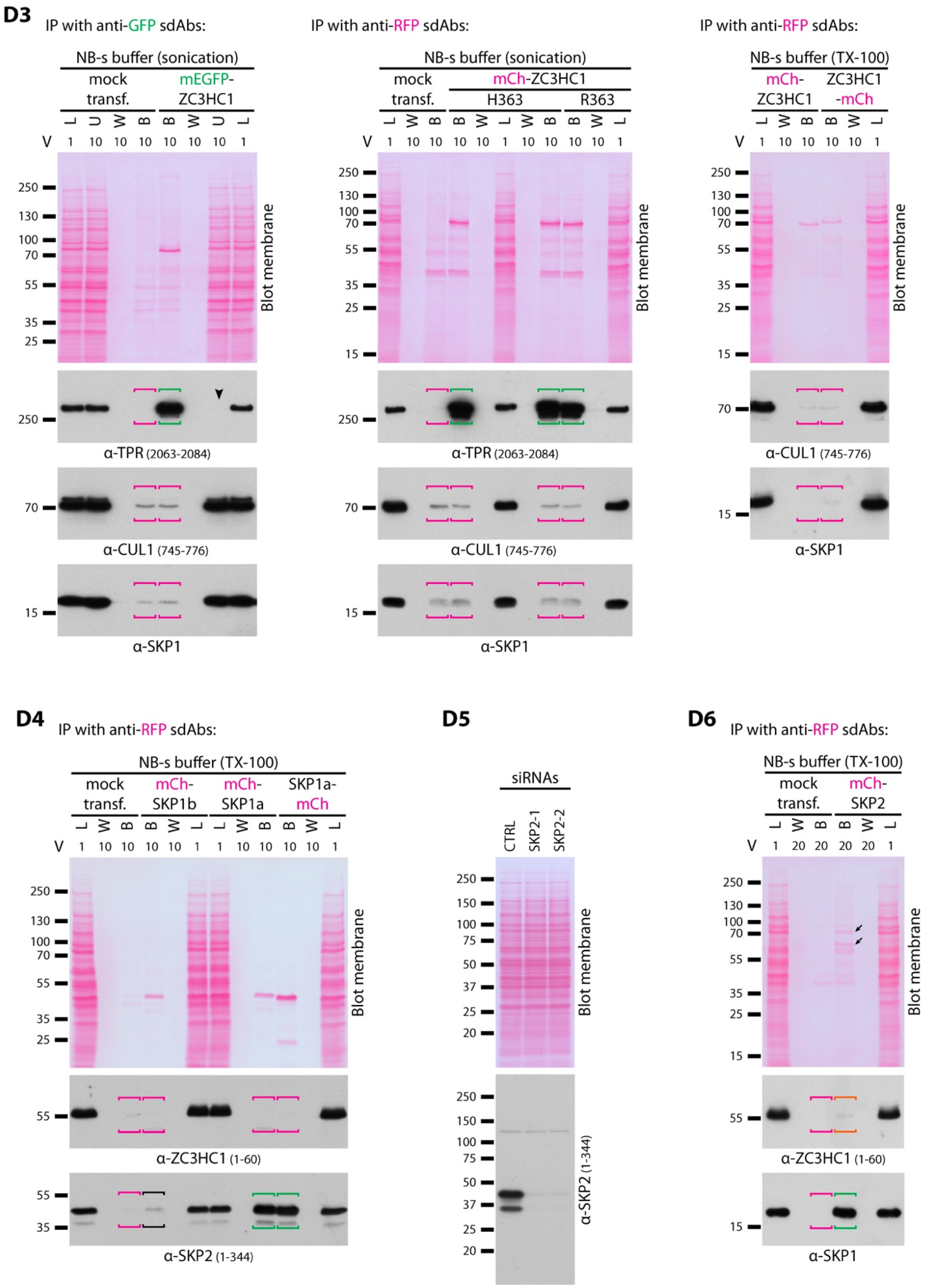
D3
IP with anti-GFP sdAbs:
NB-s buffer (sonication)
mock transf.
mEGFP-ZC3HC1
L U W B B W U L
V 1 10 10 10 10 10 10 1
Blot membrane
α-TPR (2063-2084)
α-CUL1 (745-776)
α-SKP1
IP with anti-RFP sdAbs:
NB-s buffer (sonication)
mock transf.
mCh-ZC3HC1
H363
R363
L W B B W L W B B W L
V 1 10 10 10 10 1 10 10 10 10 1
Blot membrane
α-TPR (2063-2084)
α-CUL1 (745-776)
α-SKP1
IP with anti-RFP sdAbs:
NB-s buffer (TX-100)
mCh-ZC3HC1
ZC3HC1-mCh
L W B B W L
V 1 10 10 10 10 1
Blot membrane
α-CUL1 (745-776)
α-SKP1
D4
IP with anti-RFP sdAbs:
NB-s buffer (TX-100)
mock transf.
mCh-SKP1b
mCh-SKP1a
SKP1a-mCh
L W B B W L L W B B W L
V 1 10 10 10 10 1 1 10 10 10 10 1
Blot membrane
α-ZC3HC1 (1-60)
α-SKP2 (1-344)
D5
siRNAs
CTRL
SKP2-1
SKP2-2
Blot membrane
α-SKP2 (1-344)
D6
IP with anti-RFP sdAbs:
NB-s buffer (TX-100)
mock transf.
mCh-SKP2
L W B B W L
V 1 20 20 20 20 1
Blot membrane
α-ZC3HC1 (1-60)
α-SKP1

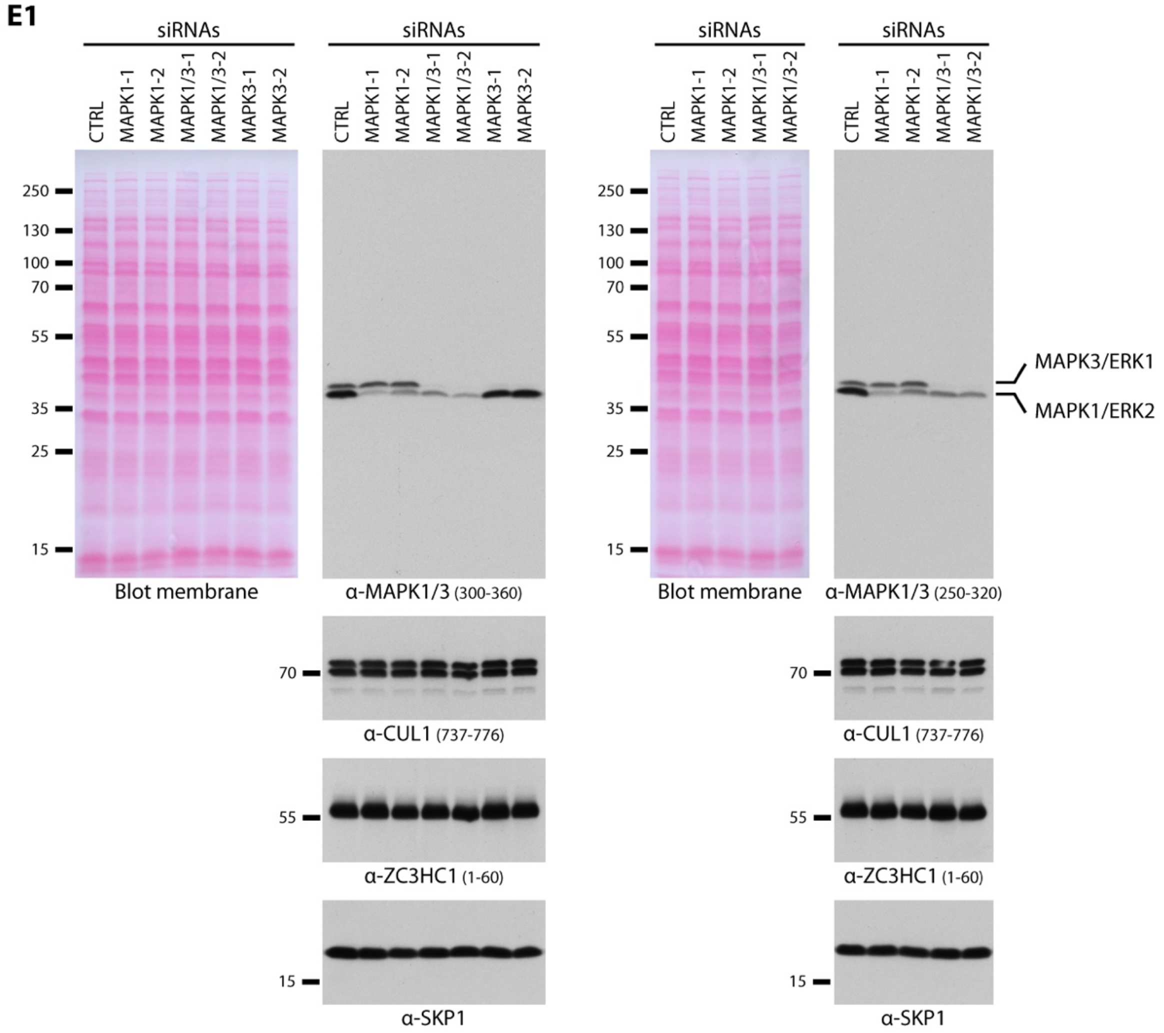
E1
siRNAs
CTRL
MAPK1-1
MAPK1-2
MAPK1/3-1
MAPK1/3-2
MAPK3-1
MAPK3-2
250
130
100
70
55
35
25
15
Blot membrane
α-MAPK1/3 (300-360)
α-CUL1 (737-776)
α-ZC3HC1 (1-60)
α-SKP1
α-MAPK1/3 (250-320)
MAPK3/ERK1
MAPK1/ERK2

S11 (6/9)

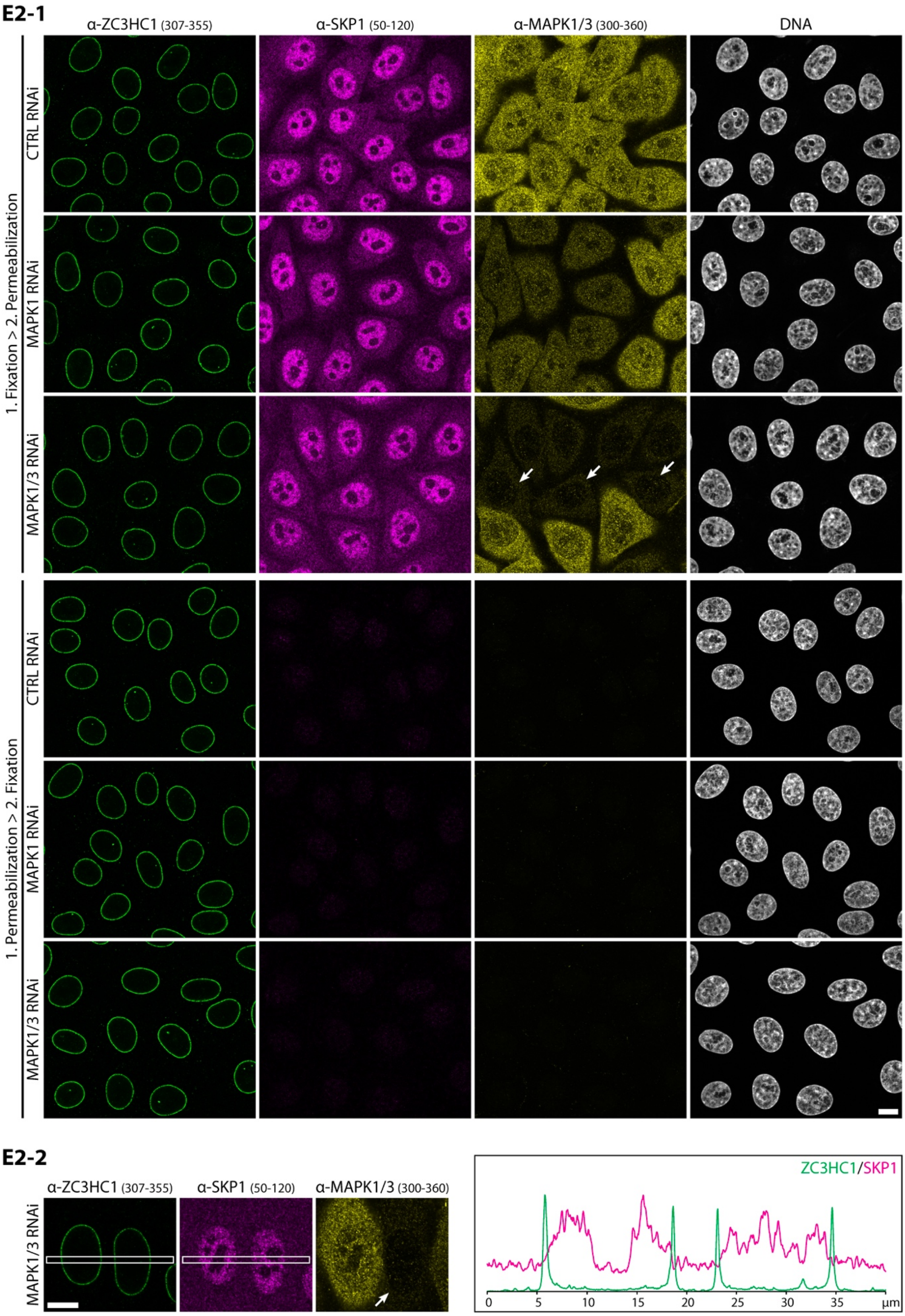
E2-1
α-ZC3HC1 (307-355)
α-SKP1 (50-120)
α-MAPK1/3 (300-360)
DNA
1. Fixation > 2. Permeabilization
CTRL RNAi
MAPK1 RNAi
MAPK1/3 RNAi
1. Permeabilization > 2. Fixation
CTRL RNAi
MAPK1 RNAi
MAPK1/3 RNAi
E2-2
α-ZC3HC1 (307-355)
α-SKP1 (50-120)
α-MAPK1/3 (300-360)
MAPK1/3 RNAi
ZC3HC1/SKP1
0
5
10
15
20
25
30
35
μm

S11 (7/9)

E2-3

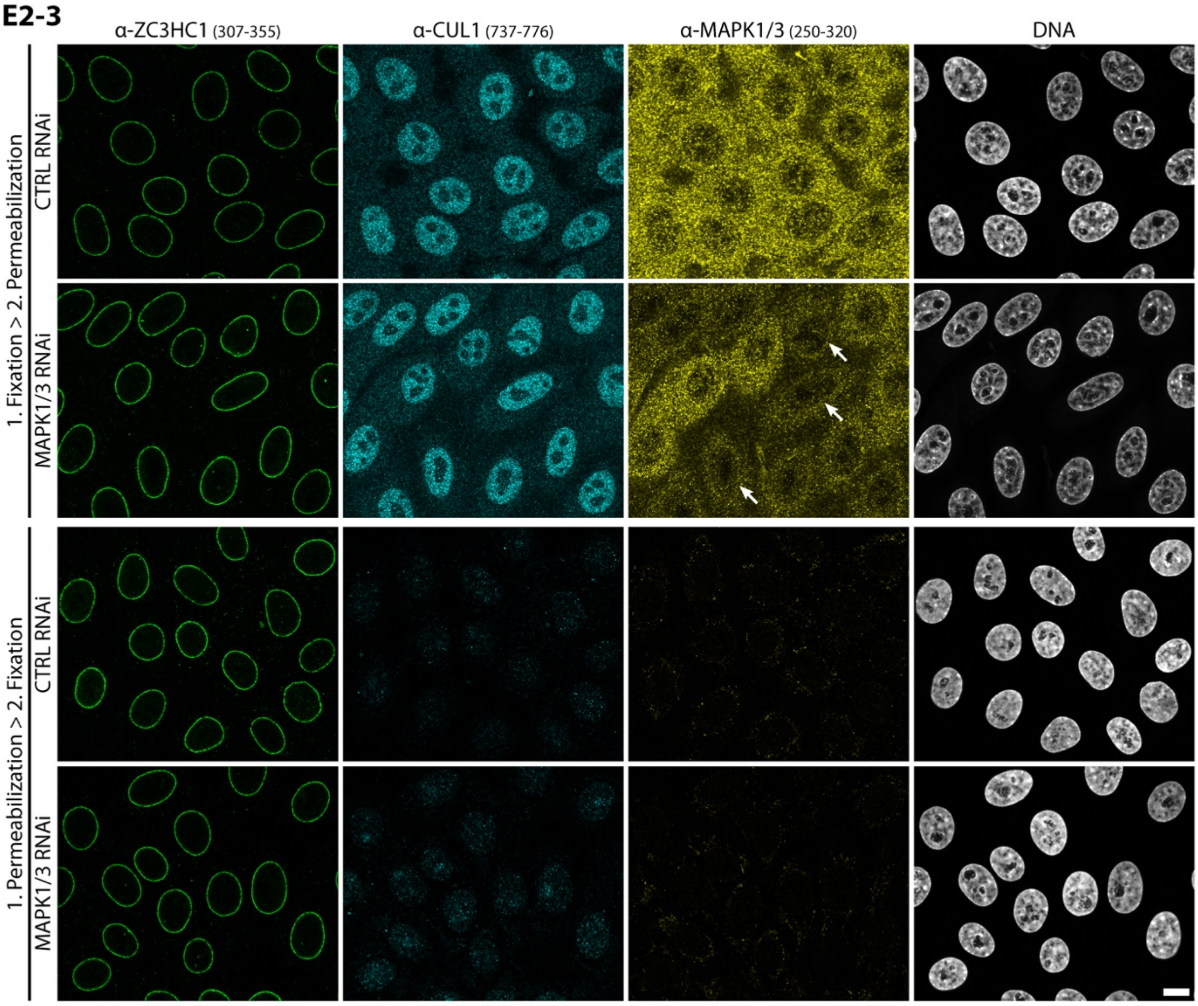
α-ZC3HC1 (307-355)
α-CUL1 (737-776)
α-MAPK1/3 (250-320)
DNA
1. Fixation > 2. Permeabilization
CTRL RNAi
MAPK1/3 RNAi
1. Permeabilization > 2. Fixation
CTRL RNAi
MAPK1/3 RNAi

F1

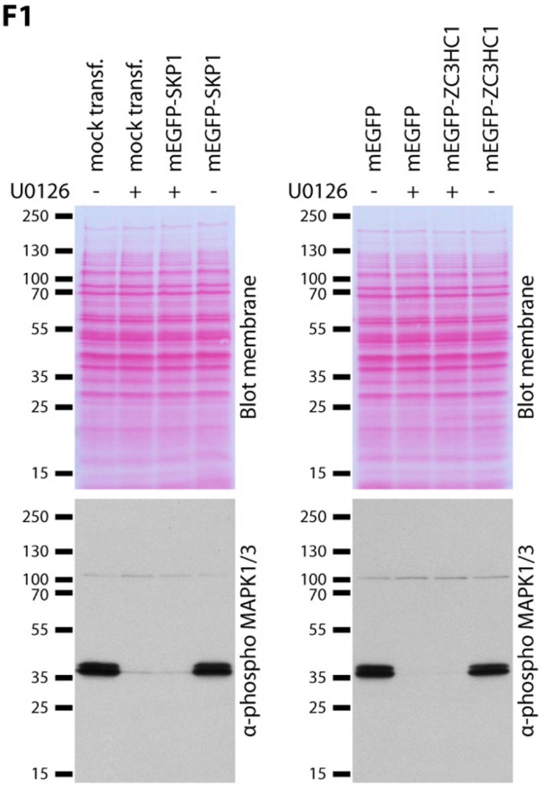
mock transf.
mock transf.
mEGFP-SKP1
mEGFP-SKP1
U0126 - + + -
mEGFP
mEGFP
mEGFP-ZC3HC1
mEGFP-ZC3HC1
U0126 - + + -
250
130
100
70
55
35
25
15
Blot membrane
α-phospho MAPK1/3

F2

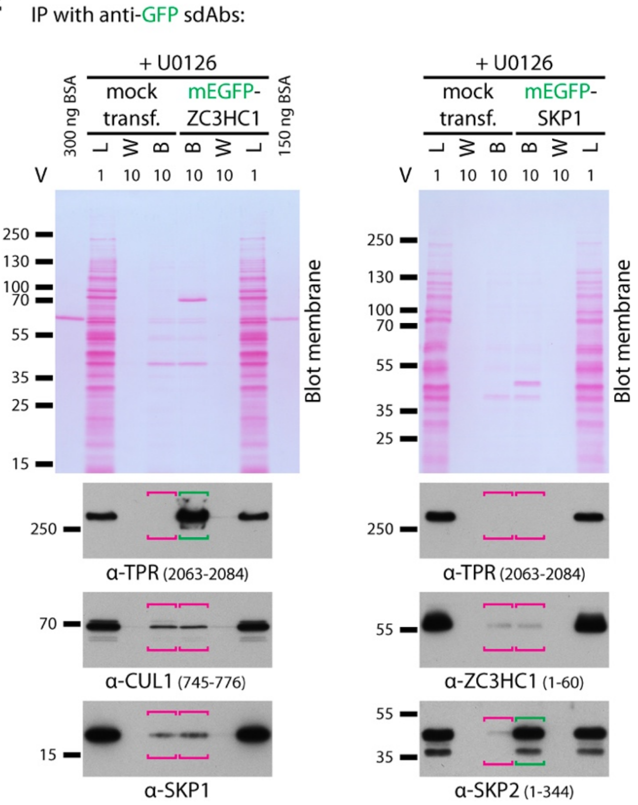
IP with anti-GFP sdAbs:
+ U0126
mock transf.
mEGFP-ZC3HC1
300 ng BSA
150 ng BSA
L W B B W L
V 1 10 10 10 10 1
+ U0126
mock transf.
mEGFP-SKP1
L W B B W L
V 1 10 10 10 10 1
Blot membrane
α-TPR (2063-2084)
α-CUL1 (745-776)
α-SKP1
α-TPR (2063-2084)
α-ZC3HC1 (1-60)
α-SKP2 (1-344)

G1

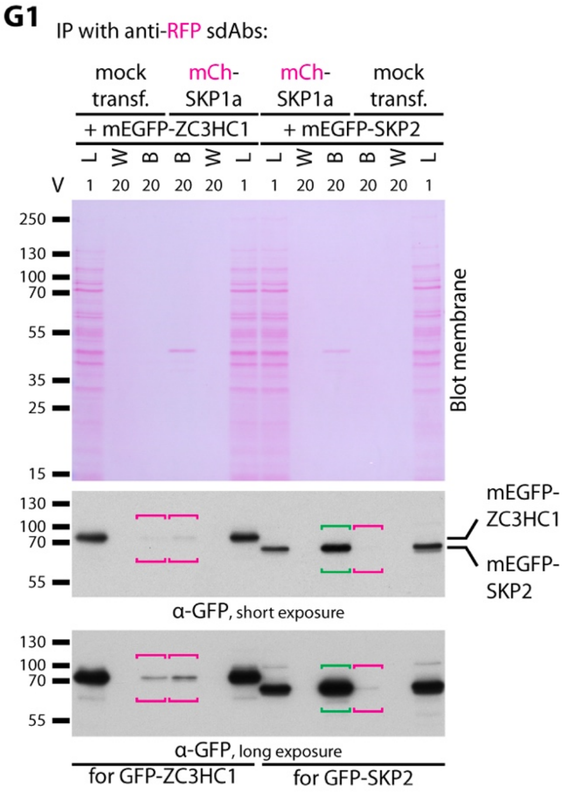

G2

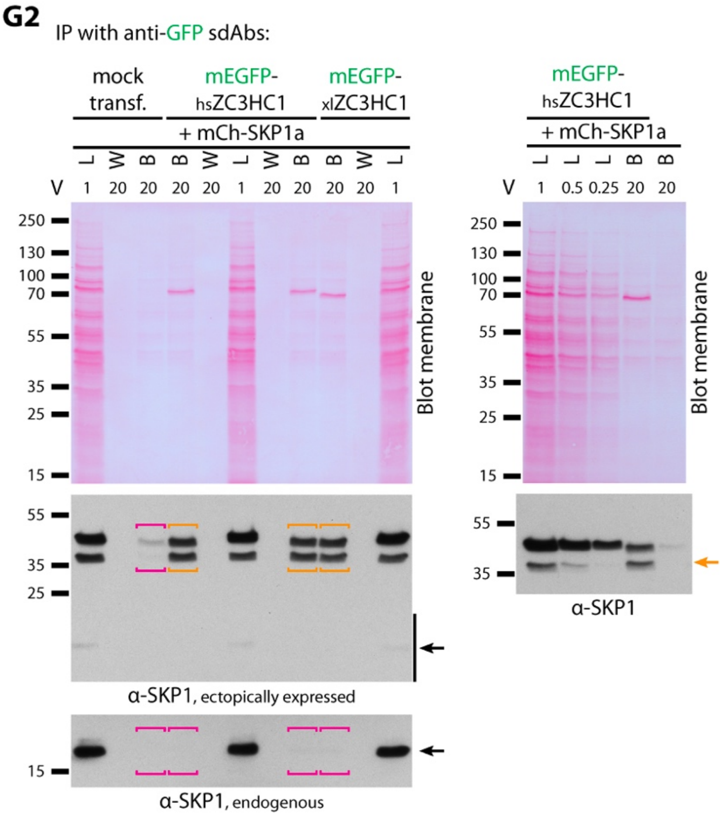

G2 (continued)

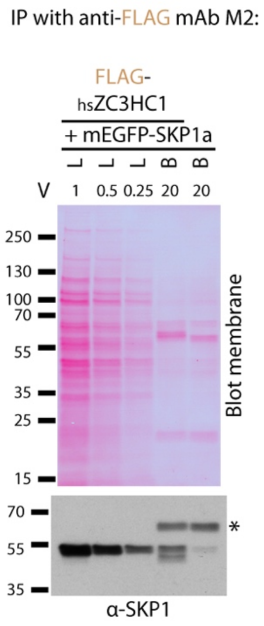

G3

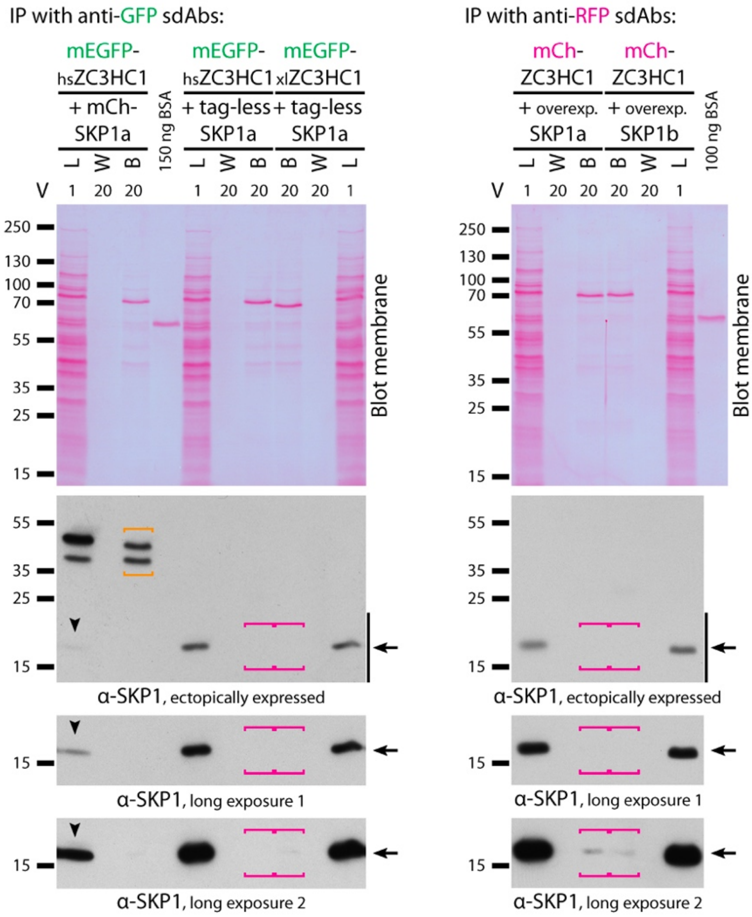

Supplemental Figure S11. ZC3HC1 in interphase neither being part of an SCF complex nor a binding partner of SKP1 under normal circumstances *in vivo*.

Among the proteins initially identified as NB components in *Xenopus* oocytes, ZC3HC1 was one whose reported binding partners and function turned out not to be compatible with various datasets accumulated in the course of our study. In fact, ZC3HC1 had formerly been described as a primarily SKP1-interacting protein, with SKP1 reportedly keeping ZC3HC1 tethered to the SCF complex all throughout interphase in various types of cells, with such SCF complex described as functioning in cell cycle regulation [33,85–89], and with all this commonly regarded being the cellular interactome and function of ZC3HC1 to date (e.g., https://www.uniprot.org/uniprot/Q86WB0; https://www.ensembl.org/Homo_sapiens/Gene/Summary?db=core;g=ENSG00000091732;r=7:130018287-130051451; https://www.ncbi.nlm.nih.gov/gene/51530; https://www.genecards.org/cgi-bin/carddisp.pl?gene=ZC3HC1). Thus, addressing apparent conflicts between these published data and some of our findings, we aimed to find out whether one could reconcile the diverging datasets.

In the early stages of our study, we had actually considered it well possible that ZC3HC1 might have several functions at different places within the cell, with those at the NE distinct from those that ZC3HC1 might have somewhere else, including possibly such as a component of an SCF complex. Even though already back then all ZC3HC1 that we had been looking at in non-cycling, terminally differentiated cells, like those from *Xenopus* brain and other organs, appeared to be located at the NE, we considered this reconcilable with the notion that a ZC3HC1-SCF complex would simply not be required in cell types in which there might be less need for controlling the levels of proteins involved in cell cycle progression. Furthermore, having detected pools of soluble nuclear ZC3HC1 in several other cell types by then, and having found that such soluble ZC3HC1 usually existed as polypeptides not bound to TPR, we initially had considered it possible that this soluble nuclear ZC3HC1, instead of binding to TPR, would bind to SKP1 and then be part of an SCF complex with some role in cell cycle regulation. Especially in the oocyte, this scenario did not appear unlikely to us at first, in view of the existence of a conspicuous nuclear pool of soluble ZC3HC1 in this cell type, representing a cell on the verge of ultimately racing through numerous cell cycles during embryogenesis. Moreover, once we had detected small but notable pools of soluble ZC3HC1 also in some cultured cell lines, like XL-177, we deemed it possible that these ZC3HC1 polypeptides were part of soluble SCF complexes. Since the mass spectrometric analyses of the protein complement of manually isolated *Xenopus* oocyte NEs had not pointed to the existence of an SCF complex at the oocyte's NE (see also further below), we could envision a scenario in which binding of a soluble ZC3HC1 polypeptide to either soluble SKP1 or to NPC-anchored TPR would occur in a mutually exclusive manner. While we initially deemed it possible that such a ZC3HC1-SCF complex could play some role in cell cycle regulation, we merely regarded it as not comprehensible why such an SCF complex should be required for the degradation of CCNB1, for cell biological reasons that are outlined in detail in the discussion of the current study.

However, while ZC3HC1 had been described as a protein that all throughout interphase would only be part of an SCF complex, from which it would only dissociate at the beginning of mitosis in order to be then degraded [e.g., 33,85,87], no published data allowed for actually estimating how much of the cells' natural content of ZC3HC1 would indeed be part of such an SCF complex. In fact, at the beginning of our project, the data provided as evidence for a close interaction of ZC3HC1 and SCF complex stemmed from two studies, presenting IP and affinity-chromatographic data [33,86], which were only later complemented by further IP results [87–90]. However, none of these studies had allowed for assessing how much of the presumed interaction partner, relative to its amount in the starting materials, had been co-isolated with the actual target protein. In addition, specificity of such co-IPs had not been a topic in some of the studies, in which control IPs without the actual IP target protein or with unrelated antibodies had not been included [86–88]. Furthermore, while the issue of a naturally occurring interaction between the cells' endogenous ZC3HC1 and SCF polypeptides had been addressed in some individual experiments [33,89,90], most of these studies had used vast amounts of the one or other kind of overexpressed tagged protein for attracting endogenous polypeptides of the presumed interaction partner [33,86,88-90], and the seemingly most striking interactions had actually been observed after having overexpressed both interaction partners as recombinant proteins together in mammalian cells [33,87].

Starting from our reflections on the early studies that had reported ZC3HC1 being part of an SCF complex, and later also conducting additional series of experiments, then taking information into account from further studies reportedly confirming the existence of a ZC3HC1-containing SCF complex, we sought in an unbiased manner for any piece of experimental evidence that might confirm the existence of a genuine ZC3HC1-SCF complex anywhere in any of the different cell types investigated, and that might allow for reconciling former data with ZC3HC1 being an NB protein. With this in mind, and well aware that any conclusion of ours in this matter must neither be expressed carelessly nor inconsiderately, we aimed at systematically addressing this issue by not only testing different experimental conditions but also by grouping the interaction experiments to be performed into three main categories. These categories were to reflect the different types of experiments that had been conducted in the former studies.

(1) The first group included all those approaches that focussed on any naturally occurring interaction between the endogenous ZC3HC1 and SCF polypeptides within different cell types.

(2) In the second category, tagged versions of ZC3HC1, of SKP1, and of a genuine F-box protein for comparison were to be individually expressed in cells and then used for attracting the endogenous polypeptides of the one or other interaction partner.

(3) Finally, in the third group, potential interaction partners were to be ectopically expressed within cells as pairs of recombinant polypeptides to study interactions between them.

Together, these approaches aimed at providing different pieces of information required for eventually answering two central questions, namely (i) whether there might be a naturally occurring interaction detectable between SCF components and those ZC3HC1 polypeptides within a cell that naturally occur in a soluble state and are not bound to the NE, and (ii) whether even NB-associated ZC3HC1 polypeptides might engage in such interactions under certain conditions.

First, we investigated whether those native ZC3HC1 polypeptides that belong to a natural soluble pool might interact with components of the SCF complex, like SKP1 and CUL1. To this end, we analysed whether these proteins would be co-immunoprecipitated together with ZC3HC1 from three different types of *Xenopus laevis* cell extracts in which ZC3HC1 had been found occurring in such a naturally soluble state. These were the (1) post-mitotic egg extracts that were capable of nuclear assembly, yet with the proteins therein nonetheless being in a still soluble state, (2) the soluble nuclear proteins of the G2-arrested oocyte, known by then to harbour conspicuous amounts of SCF components and soluble ZC3HC1, the latter sometimes being even more abundant there than at the NE, and (3) the soluble pool of proteins from XL-177 cells in interphase, which hardly contained any soluble TPR but a small yet genuine pool of soluble ZC3HC1, recurrently notable during XL-177 cell fractionations (see also Figure S2B).

In the end, however, also after having tested for this purpose different buffer conditions, as well as different ZC3HC1 antibodies including such that did not target the alleged SKP1 binding interface of ZC3HC1, none of these experiments, a selection of which is presented further below in S11A, revealed any specific interaction between ZC3HC1 and the genuine SCF complex proteins SKP1 and CUL1. Furthermore, similar experiments performed with cell extracts from some of those human cell lines, like HCT116 and HEK293T, in which a small soluble pool of ZC3HC1 existed in interphase, did not provide evidence for a specific interaction between ZC3HC1 and either SKP1 and CUL1 (data not shown). However, with hardly any soluble TPR naturally occurring in interphase in these cells, the positive control results, namely the co-IP of TPR, did not appear sufficiently impressive to us when the cell extracts used for such IP experiments had been prepared under conditions that maintained NB integrity. Such preparation under NB-s conditions had actually been driven by the intention to inspect only the genuine soluble pool of ZC3HC1 and not have this pool mixed with additional amounts of ZC3HC1, released by non-physiological conditions from the NB together with certain amounts of TPR.

However, since we regarded convincing co-IPs as positive controls for the ZC3HC1 IP experiments with somatic cell extracts as indispensable at some point, we addressed this issue by solubilising distinct amounts of the NB-associated TPR polypeptides in some of the cell fractionation experiments. Such solubilisation in a variety of ways (see further below) resulted in elevated amounts of truly soluble TPR within the corresponding cell extracts, in which they were then in principle available as binding partners for ZC3HC1 in some of the later IP experiments (see further below).

As additional experiments within category 1, we looked for evidence in support for an interaction between the endogenous ZC3HC1 polypeptides that occur attached to the NB polypeptides on the one hand and the endogenous SKP1 and CUL1 polypeptides on the other. Our decision to conduct such searches primarily in human cells from then on had several reasons. Firstly, the mass spectrometric analyses of *Xenopus* oocyte NEs had already pointed at only trace amounts of NE-associated SKP1 in this cell type (one exclusive spectrum count each in only four out of 22 datasets for *X. laevis*, and none in four datasets for *X. tropicalis* oocyte NEs) and hardly any CUL1 (one exclusive spectrum count in one out of the 22 datasets for *X. laevis*, and none in the *X. tropicalis* datasets), with the few SKP1 and CUL1 peptides detected not even correlating with the presence or absence of the NBs. Conversely, thousands of exclusive spectrum counts in total had been obtained from the same oocyte NE preparations for a reference protein, like, for example, NUP107. Secondly, many of the former cell culture-based experiments that had led to the portrayal of a ZC3HC1-containing SCF complex had been conducted with materials obtained from human cell lines like HeLa and HEK293T. And finally, working with human cells from then on had the advantage of more easily achieving RNAi-mediated KD of one or the other target protein of interest (see also Information SI 10).

First, we inspected whether colocalisation between ZC3HC1, SKP1 and CUL1 might be detectable at the NEs of such human cells. However, despite testing several target-specific SKP1 and CUL1 antibodies (for a selection, see S11C1) and different IFM protocols, the SCF components were not found to colocalise with the NE-associated ZC3HC1, as exemplified in S11B. Next, we also inspected whether the RNAi-mediated KD of SKP1 or CUL1 would affect the localisation of ZC3HC1 at the NE, also in view of the former reporting that only the alleged interaction with the SCF complex would prevent all cellular ZC3HC1 from being destabilised and degraded [87]. However, when SKP1 or CUL1 were knocked down by RNAi, this had no effect on the amount and localisation of ZC3HC1 at the NE, as shown in S11C2.

In summary, none of the different experiments of category 1, addressing putative interactions between the endogenous proteins, provided any evidence in support of a naturally occurring interaction in interphase between the soluble or the NB-associated polypeptides of endogenous ZC3HC1 on the one hand and endogenous SKP1 or CUL1 on the other.

The experiments of category 2 included ectopically overexpressing recombinant ZC3HC1 in human cells to obtain an excess of soluble ZC3HC1 polypeptides and investigate whether these, once all binding sites at TPR polypeptides would have been saturated, might perhaps then be capable of interacting with endogenous SCF complex components. In

addition, we conducted the same type of experiments with recombinant SKP1, looking for an interaction with endogenous ZC3HC1.

As one of the positive controls for successful interaction with SKP1, we chose SKP2, as a genuine F-box protein representative, whose interaction with SKP1 was well established [e.g., 91–96]. The involvement of SKP2 was meant to address the questions of (i) how the interaction of the ectopically expressed SKP1 with the endogenous F-box protein SKP2 would compare to the interaction between such recombinant SKP1 and the endogenous polypeptides of the alleged F-box protein ZC3HC1, and (ii) how the attraction of endogenous SKP1 by ectopically expressed SKP2 would compare to any attraction of endogenous SKP1 by the ectopically expressed ZC3HC1.

Furthermore, since we also needed a positive control demonstrating successful interaction with ectopically expressed ZC3HC1, and since TPR was the only ZC3HC1 interaction partner known to us by then, the IP experiments with the recombinant ZC3HC1 were performed with cell extracts that had been prepared in different ways. Some of these extracts were actually prepared in parallel and then used side by side for the same series of IP experiments.

Once again, such extracts could be grouped into three categories, of which the second and third category extracts provided recurrently reproducible TPR co-IPs as positive control results that we regarded as sufficiently convincing. Among these extracts were those in which certain amounts of the normally NB-appended TPR polypeptides were instead present in solubilised form.

(1) Cell extracts of the first category were generally obtained under NB-stabilising (NB-s) conditions. This included permeabilisation of cells in NB-s buffers, which in the past had allowed for maintaining the interaction between all endogenous TPR and ZC3HC1 at the NE, with non-ionic detergents included in concentrations just as low as required for quantitatively releasing all soluble material from the nuclear interior. Among these solutions were also those NB-s buffers that had already been used for the IP experiments with the *Xenopus* cell extracts.

In those cases in which SKP1 or SKP2 had been ectopically expressed within the human cells, the final cell extracts obtained by such procedures once again contained, as non-mitotic proteins, only those ZC3HC1 polypeptides and those trace amounts of TPR, if at all, that naturally occur in interphase. Only when ZC3HC1 had been ectopically expressed, the endogenous ZC3HC1 polypeptides, which then had had to compete with the recombinant versions for binding sites at the NE, occurred in somewhat larger soluble amounts. In some of these experiments, additional small amounts of soluble TPR were noted too, with the latter possibly having been prevented from being correctly recruited to or stably incorporated into the NB (our unpublished data), in the presence of an excess of recombinant ZC3HC1 that exceeded the natural cellular ZC3HC1 content in HEK293T cells by up to three orders of magnitude (see further below).

(2) The other two major types of cell extracts, by contrast, harboured large non-mitotic pools of soluble TPR, with such TPR having been released from the NBs by different physicochemical means. In the one type of such cell extracts, the cells had been permeabilised with detergents in the one or other NB-destabilising (NB-d) buffer. While certain NB-d buffers caused subsequent aggregation and precipitation of some NB proteins like TPR (our unpublished data), other NB-d buffers allowed for the solubilisation of about half or more of the total amount of NPC-associated TPR, with most or all of the NE-bound ZC3HC1 then having been solubilised as well. Among the tested buffers and solutions causing such different effects was also the buffer that apparently had been used for the cell permeabilisation and IP experiments described earlier (e.g., [33,86,87,97]; for almost identical conditions, see [89], and [90]).

Even though some of the NB-d buffers notably varied in composition, all had in common that they lacked sufficient concentrations of divalent cations. Cell extracts obtained in this way were either used directly for IP experiments, which generally resulted in no or notably attenuated interaction between TPR and ZC3HC1, or they were first re-supplemented with salts to make buffer conditions more similar again to an NB-s buffer, and thus to physiological conditions within a cell. Especially when incubated at RT, this latter approach then allowed for renewed, often quantitative interaction between TPR and ZC3HC1 in such extracts.

(3) For extracts of the third category, we used an NB-s buffer with ample concentrations of $MgCl_2$ and without any detergent. The cells and their nuclei were ruptured by short pulses of sonication, in a manner that resulted in the total sample's mean temperature having increased by maximally only 2°C, determined with a microprobe thermometer. This sonication procedure, established by systematic optimisation, neither resulted in any major disintegration of the NPCs nor in fragmentation of the NEs into primarily small-sized parts (our unpublished data). However, even though the cells were kept in an NB-s buffer for this purpose, we had found this approach to solubilise distinct subpopulations of NB-associated TPR and ZC3HC1 polypeptides, which then at low temperatures remained in solution also upon centrifugation at 200,000 g (our unpublished data). Nonetheless, such solubilised TPR and ZC3HC1 polypeptides apparently had also remained capable of binding to each other, evident once the extracts were incubated at RT.

Since the rupturing of cells by brief sonication allowed for omitting the use of detergents, scenarios could be avoided in which such a detergent might perhaps have interfered with some kind of interaction between ZC3HC1 and the SCF core complex components, especially since also non-ionic detergents like TX-100 were known to affect distinct types of protein-protein interactions [e.g., 98,99].

Clearly, however, irrespective of which recombinant protein had been ectopically expressed, the cell extract preparations applying category 2 and 3 protocols resulted in artificially large amounts of soluble endogenous ZC3HC1, present in addition to the naturally occurring small pool of soluble ZC3HC1, and this was taken into consideration when later interpreting the corresponding IP results (see further below).

In addition to performing such IP experiments with cell extracts obtained in different ways, and with the IP bead incubations performed in different buffers, we also tested different types of tags, appended to either the N- or C-terminus of ZC3HC1, SKP1 and SKP2.
(1) Since NIPA/ZC3HC1 ectopically expressed in human cells in the former studies, in order to be used for co-IP of endogenous SKP1 or CUL1 [86,88–90], had always been equipped with the FLAG-tag [100], we used this tag as one of several tags for our experiments as well, allowing for the IP with FLAG-tag-specific antibodies.
(2) In addition, we transfected cells with expression vectors for ZC3HC1 tagged with various fluorescent proteins (FP), here exemplified by either the monomeric FP mCherry or by a non-homodimerising version of EGFP. Furthermore, the ectopically expressed versions of SKP1 and SKP2 were tagged with such FP proteins as well. This allowed for making use of the nanotrap technology for fluorescent fusion proteins [101], representing a highly effective way of isolating proteins and subcellular complexes with the help of high-affinity single-domain antibodies (sdAbs).

Deliberately using the "nano-trapping" beads and the IgG-coated beads for conventional IPs in amounts in which their binding capacity only moderately exceeded a target protein's total loaded amounts, we nonetheless achieved quantitative immunodepletion of each of the different targets. Furthermore, we only conducted a few and very brief bead washes, lasting in total only a few minutes, always using the same buffer as for the actual incubation of the beads with the extracts, thereby minimising the risk of losing any potential specific binding partner. Since most of our experiments were performed with NB-s buffers, this meant that in most IP experiments the washing step buffers too resembled the physiological conditions within a cell more closely than other buffers sometimes used for IP experiments. Only in some early IP experiments in which incubations were performed in the absence of detergent, we used the same NB-s buffer supplemented with 0.02% Tween-20 for the washing steps, which we later, though, controlled to not negatively have affected any of the true or alleged interactions between ZC3HC1 and the other proteins investigated in the current study.

Finally, we used several different human cell lines to ectopically express FLAG- and FP-tagged polypeptides. For all systematic side-by-side experiments, though, we primarily used HEK293T cells, exploiting this cell line's extremely good transfectability and because it had also been used for some of the former studies' IP experiments.

In these experiments, the FLAG-tag-specific IgGs and the high-affinity sdAbs, including one specifically targeting different variants of Green Fluorescent Protein (GFP) and another one specific for a Red Fluorescent Protein (RFP) like mCherry, allowed for reliably purifying the tagged polypeptides from the cell extracts, followed by analysing the immunoprecipitated material for co-enriched proteins by IB (examples presented further below) and mass spectrometry (our unpublished data). In the end, however, while all of the different IP conditions had allowed for isolating vast amounts of either recombinant ZC3HC1 or recombinant SKP1 in a very efficient manner, these experiments too, as demonstrated by a selection of representative datasets presented in S11D, had not provided evidence in support of the notion that ZC3HC1 and an SKP1- and CUL1-containing SCF complex are natural binding partners. By contrast, the ectopically expressed SKP1 had turned out capable of binding endogenous SKP2 and, *vice versa*, the recombinant version of SKP2 had attracted endogenous SKP1.

In particular, in none of the NB-s buffers had endogenous CUL1 been co-sedimented with any of the FP-tagged ZC3HC1 polypeptides above background levels, the latter representing minor amounts similarly detectable together with (i) the fluorescent proteins (FP) expressed and immunoprecipitated on their own (data not shown), and even together with (ii) beads that had been incubated with HEK293T cell extracts containing no ectopically expressed protein at all, as exemplified in S11D2, S11D3, and S11F. Only with the FLAG-tagged version of ZC3HC1 did we sometimes note some trace amounts of CUL1, just hardly detectable above background levels, among the immunoprecipitated material, as exemplified in S11D2. However, as described further below, we calculated these few barely detectable CUL1 polypeptides to represent at most less than 50 copies per HEK293T cell, equivalent to less than 0.2% of the approximated total content of endogenous CUL1 in a HEK293T cell, in contrast to millions of FLAG-tagged ZC3HC1 polypeptides per cell. In other words, only about one CUL1 polypeptide per 100,000 copies of recombinant FLAG-ZC3HC1 was found to have sporadically ended up among the immunoprecipitated material while the equivalent of some ten-thousands of soluble CUL1 polypeptides per cell had remained in solution.

Different from CUL1, minute amounts of SKP1 were more often found to have been co-sedimented together with the amassment of ectopically expressed ZC3HC1 polypeptides, irrespective of whether the IPs had been conducted in the presence of the NB-s buffers or the formerly used IP buffers. While this was observed for basically all FP-tagged versions of ZC3HC1, it was generally most pronounced, as exemplified in S11D2, when having conducted such IP experiments with IgGs and the FLAG-tagged version of ZC3HC1. However, even then, the number of co-sedimented SKP1 polypeptides was calculated to correspond to 700-900 copies per cell, equivalent to about 1% of a HEK293T cell's approximated total content of endogenous SKP1. This, in turn, meant that per 5000 copies of tagged ZC3HC1, only about one SKP1 polypeptide had been co-sedimented, while again the equivalent of several tens of thousands of soluble SKP1 polypeptides per cell had remained in solution.

Furthermore, knowing by then that ZC3HC1 naturally occurs in two variants, due to a single amino acid polymorphism at residue 363, which can either be an arginine or a histidine [e.g., 102–109], we also tested both of these variants with regard to their binding to SKP1 and CUL1 in parallel. This, however, did not reveal any apparent difference between the two ZC3HC1 variants either, both of them being similarly unattractive for endogenous SKP1 and CUL1, as exemplified in S11D3.

In apparent contrast to the results with regard to SKP1 and CUL1, the IP of ZC3HC1, in any of its ectopically expressed differently tagged versions, when having used any of the NB-s buffers for the bead incubations, had always come along with conspicuous, often even quantitative co-IP of those soluble endogenous TPR polypeptides that were present in the one or other type of HE293T cell extracts and did not stem from mitotic cells. In some of these experiments, the NB-detached and then soluble TPR amounts represented at least 20,000 copies per cell, all of which could be co-immunoprecipitated again together with the tagged ZC3HC1 polypeptides. Examples of such quantitative co-IP are presented in S11D3, confirming the interaction between TPR and ZC3HC1.

While the IP of the tagged versions of ZC3HC1 had been noted to come along with co-sedimentation of some trace amounts of endogenous SKP1, we were even more interested in finding out whether an excess of tagged SKP1 would be capable of attracting the naturally occurring small amounts of soluble endogenous ZC3HC1. However, this was not the case. Irrespective of whether the immunoprecipitated SKP1 had been tagged at its N- or C-terminus, or which of the different FPs had been appended, no naturally occurring soluble ZC3HC1 polypeptides were found co-sedimented in any of these IP experiments. In all but one experiment, not even trace amounts were detectable above the anyhow hardly visible background. The one case with a trace of ZC3HC1 just above background turned out, after having performed corresponding dilution series for subsequent calculations, to represent, at most, two copies of soluble ZC3HC1 per HEK293T cell.

Also, no evidence for an interaction between endogenous ZC3HC1 polypeptides and ectopically expressed SKP1 came from experiments in which we had ectopically expressed and immunoprecipitated the canonical and the shorter isoform of SKP1 next to each other, as exemplified in S11D4, with neither the longer nor shorter SKP1 isoform having been capable of attracting the endogenous ZC3HC1 polypeptides. By contrast, while the shorter SKP1 isoform could also barely bind SKP2, the cells' endogenous SKP2 polypeptides were readily co-immunoprecipitated with the canonical version of SKP1, as also exemplified in S11D4.

As additional control experiments, we ectopically expressed and immunoprecipitated tagged versions of SKP2 as the genuine F-box protein representative. While similarly tagged ZC3HC1 had not been capable of attracting any conspicuous amounts of endogenous SKP1, the latter were found to be readily co-immunoprecipitated with the tagged SKP2, as exemplified in S11D6. Of further note, though, and in contrast to no ZC3HC1 generally detectable upon IP of tagged SKP1, the IP of SKP2 also resulted in some co-sedimentation of endogenous ZC3HC1, in trace amounts, but above background level nonetheless. Further analyses revealed that such traces of ZC3HC1 could represent copy numbers equivalent to up to 10 polypeptides per HEK293T cell which might have engaged in some kind of interaction with the recombinant SKP2. However, only knowing at that time that SKP1 is in principle capable of homodimerising [e.g., 95,110,111], we could not tell until then whether the observed co-sedimentation of traces of ZC3HC1 represented a direct interaction with SKP2, or rather an indirect one via the co-immunoprecipitated SKP1 polypeptides, with one SKP1 molecule binding via its F-box-binding interface the F-box of SKP2 and perhaps the homodimer's other interface sporadically binding a ZC3HC1 polypeptide. However, a most recent study reporting that SKP1 dimerisation results in concealing its F-box protein binding site [112] now allows for proposing a scenario in which the co-IP of SKP1 can only occur via heterodimer formation between SKP1 and SKP2, thereby occupying the F-box binding interface of SKP1. This, in turn, would mean that any ZC3HC1 sporadically bound to SKP1, and not to SKP2, would need to adhere to some other part of SKP1 that is not its F-box binding domain.

Apart from the so far described experiments of our search for some evidence in support of the reported relationship between ZC3HC1 and the SCF complex, we also considered the findings of a study in which (i) the kinase ERK2/MAPK1 had been reported to dissociate the alleged interaction between ZC3HC1 and SKP1, respectively the SCF complex, and in which (ii) RNAi of MAPK1 had been stated to notably stabilise a strong interaction between ZC3HC1 and the SCF [113]. Since MAPK1 is known to be constitutively active in numerous tumour cell lines also in interphase [e.g., 114,115], we initially could indeed not rule out a scenario in which missing colocalisation between ZC3HC1 and SCF complex components at the NE might simply be due to some ongoing MAPK1 activity that resulted in permanently destabilising and disrupting some maybe existing interaction between the NB-resident ZC3HC1 and the SCF complex components SKP1 and CUL1.

To also address this notion, as shown in S11E, we knocked down MAPK1 alone, or together with ERK1/MAPK3, by RNAi, and then reinvestigated the localisation of SKP1 and CUL1, relative to that of ZC3HC1. Again, however, we could not detect any colocalisation of ZC3HC1 and SCF components in MAPK1/3-deficient cells, as exemplified by a selection of IFM micrographs presented in S11E2.

Furthermore, it had also been reported [88] that suppressing MAPK1/3 activity by incubating cells with U0126, an inhibitor of MEK1/2 (MAP2K1/2) acting upstream of MAPK1/3 [116], would as well result in stabilising and even enhancing the degree of binding between overexpressed ZC3HC1 and the SCF complex, the latter represented in that study only by CUL1 though [88]. Again, however, we could neither observe any colocalisation of SKP1 or CUL1 with ZC3HC1 by IFM in different cell lines treated with U0126 (our unpublished data), nor did we find ZC3HC1 and the SCF core components specifically co-immunoprecipitated from the cell extracts of such U0126-treated cells in any considerable amounts. In fact, very similar to those IP experiments performed with extracts of cells not treated with U0126, the IPs of N-terminally tagged versions of ZC3HC1 came along with no or, if at all, only trace amounts of endogenous SKP1 hardly above background levels within the precipitated material, along with essentially no CUL1 at all. Similarly, upon the IP of

vast amounts of N-terminally tagged SKP1 from such U0126-treated cell extracts, once again, not even trace amounts of endogenous ZC3HC1 beyond background levels were detected within the immunoprecipitate, with representative immunoblots shown in S11F2.

In summary, and like for the different experiments of category 1, none of the experiments of category 2, which had addressed putative interactions between an ectopically overexpressed protein and the endogenous version of its presumed binding partner, had provided any convincing evidence in support of some naturally occurring interaction in interphase between ZC3HC1 and either SKP1 or CUL1.

Based on copy number calculations already briefly referred to above, the results of these category 2 IP experiments allowed for drawing even further conclusions when first considering the following additional pieces of information. (i) For most of these IP experiments, we had determined total cell numbers and transfection efficiencies of the cell populations to be used for cell extract preparations. Then, after immunoprecipitation, we calculated the ectopically expressed proteins' total amount of mass, e.g., by comparing its immunoprecipitated amounts, visible as stainable bands after SDS-PAGE and Western blotting, with serial dilutions of known amounts of reference proteins, like BSA, which we had loaded onto the same gel. This allowed for calculating that in most of these experiments, the average number of ectopically expressed polypeptides per transfected cell had amounted to several million copies at the time point of cell harvest. (ii) In the course of our study, mass spectrometric data became available for HEK293 cells that allowed for comparing the relative amounts between essentially all proteins within this cell type, ranging between lower [117] and notably higher relative amounts [118] determined for those proteins that were of interest in the context here. Furthermore, for NPC protein NUP107, the absolute copy number per NPC had been very accurately determined in HEK293 cells [14], allowing for this number to be used as a definite reference value. (iii) Furthermore, nuclear dimensions and NPC density in HEK293 were known as well [14; our unpublished data], and (iv) subcellular fractionation of HEK293 cells allowed us to determine the relative amounts of those NUP107 polypeptides that were located at the NPCs, at the ALPCs, and at other sites within this cell type (our unpublished data). Altogether this not only allowed for closely approximating the total number of NUP107 polypeptides per HEK293 cell, but also, when correlating this with the most recent mass spectrometric dataset for HEK293T [118], to approximate the total polypeptide numbers per HEK293T cell for other proteins as well, like for ZC3HC1, SKP1, and the total of all F-box proteins within this cell type. For ZC3HC1, we calculated its approximate total copy number not to exceed $1{\cdot}10^4$ within a HEK293T cell in the G1-phase, while copy numbers for SKP1 were correspondingly determined to reach at most $9{\cdot}10^4$ copies, and the total of all F-box and F-box-like proteins within this cell type to not exceed $1.4{\cdot}10^5$ copies.

These numbers then allowed for the following conclusions. First, millions of ZC3HC1 polypeptides ectopically expressed within a HEK293T cell had not been capable of successfully competing with the cell's genuine F-box proteins for the F-box-binding interface of the endogenous SKP1 polypeptides, even though the F-box proteins' total copy number was at least one order of magnitude lower than that of the recombinant ZC3HC1. Secondly, millions of recombinant SKP1 polypeptides expressed within a HEK293T had not been able to compete with TPR for its binding to ZC3HC1 successfully, with this neither having been the case *in vivo* nor *in vitro*, as outlined in the following.

Apart from the IP results, the unsuccessful competition *in vivo* had also been unveiled by the fact that the excess of overexpressed SKP1 had not resulted in any higher numbers of soluble ZC3HC1 polypeptides within the transfected cells as compared to the non-transfected ones, when these had been fractioned side-by-side under conditions maintaining NB integrity. This, in turn, meant that the SKP1 polypeptides ectopically expressed within the transfected cell, being at any time point in excess over those endogenous ZC3HC1 polypeptides that were newly synthesised during the same time as well, could not prevent such new ZC3HC1 polypeptides from binding to those NBs that were also newly assembled within this same time frame. Furthermore, essentially unchanged soluble amounts of endogenous ZC3HC1 also meant that the excess of recombinant SKP1 had not been able to displace any ZC3HC1 from the NBs within the living cells either.

Not being capable of competing *in vitro* either had been evident in those experiments in which major amounts of NB-bound TPR and ZC3HC1 within the SKP1-overexpressing cells had been artificially solubilised, either by sonication or by using NB-destabilising buffers, and had then been allowed to interact with each other again. Even though copy numbers of the recombinant SKP1 polypeptides also then still exceeded those of TPR and ZC3HC1 within such cell extracts by at least one to two orders of magnitude in different experiments, this excess of SKP1 had not been able to prevent the in principle immediately available endogenous ZC3HC1 from binding to TPR again.

Apart from having revealed that ZC3HC1 was not capable of competing with true F-box proteins for SKP1-binding and that SKP1 similarly could not compete with those proteins naturally interacting with ZC3HC1 either, our category 2 experiments had also led us to other findings of potential interest. For example, in the course of these experiments, we had also noted that some commercial CUL1 antibodies exhibit cross-reactions with CUL1-unrelated proteins, as revealed by CUL1 RNAi experiments, and that such proteins can adhere to the IgG- and sdAbs-coated beads. To avoid data misinterpretation, it was thus particularly important to control target-specificity of all CUL1 and all SKP1 and SKP2 antibodies used in the current investigation by RNAi experiments. In addition, we had also noted that the handling of commercially available beads coated with anti-FLAG mouse IgGs, to be used for the FLAG-ZC3HC1 IPs, required particular caution in order to avoid data misinterpretation following subsequent IB with mAbs, as will also be exemplified in S11D2.

However, at that time, in search still for some evidence that might point at an interaction between ZC3HC1 and SKP1, we set out to conduct the experiments of category 3, which focussed on the co-expression of tagged versions of both ZC3HC1 and SKP1, thereby aiming at vast amounts of both proteins that would then no longer need to compete with other endogenous proteins for binding sites on the alleged binding partner. In other words, the ectopically expressed ZC3HC1 would then not have to compete with the cell's endogenous pool of F-box proteins for just a limited number of endogenous SKP1 polypeptides but would now encounter an excess of "free" SKP1 instead, as the copy number of available F-box proteins would no longer be large enough to saturate the F-box binding sites of SKP1. In fact, those IP experiments in the former studies that appeared to have resulted in the most efficient co-IP of SKP1 with ZC3HC1 had been those in which both proteins had been ectopically co-expressed [33,87].

Experiments of co-transfecting the cells with pairs of expression vectors encoding differently FP-tagged versions of both proteins were complemented by various controls. These included (i) the co-expression of differently tagged versions of SKP1 and SKP2, (ii) the expression of only one tagged version of ZC3HC1 or SKP1 together with the corresponding other fluorescent protein on its own, and (iii) the use of extracts from HEK293 cells that had not been transfected with an expression vector, for mock IP experiments always performed in parallel. Subsequently, either anti-RFP/mCherry or anti-GFP/YFP sdAbs or anti-FLAG IgGs were used for the actual IPs.

The outcome of these category 3 experiments can be summarised as follows. When the ectopically expressed SKP1 polypeptides were the target for IP, the tagged versions of ZC3HC1 were again not co-sedimented beyond background levels at all or in only minute amounts. By contrast, when having ectopically co-expressed SKP1 and SKP2 in parallel, tagged in the same manner as when co-expressing SKP1 and ZC3HC1 polypeptides, SKP2 was readily co-immunoprecipitated upon IP of SKP1, as exemplified in S11G1.

However, when any of the N-terminally tagged versions of ZC3HC1 were immunoprecipitated, i.e., the GFP-, the mCherry- or the Flag-tagged versions, this too resulted in notable amounts of the recombinant SKP1 having been co-immunoprecipitated. Even though still far from being quantitative, with the co-sedimented amounts only representing between less than 0.5% and about 1% of the total cellular amount of the full-length tagged SKP1, these percentages represented tens of thousands of tagged SKP1 polypeptides per cell that had ended up bead-associated together with the immunoprecipitated millions of tagged ZC3HC1 polypeptides per cell. Of particular note, this degree of co-sedimentation was even observed after having immunoprecipitated tagged versions of the *Xenopus laevis* homolog of ZC3HC1, which lacks amino acids formerly reported as essential for interaction with SKP1. Nonetheless, the amounts of recombinant SKP1 co-sedimented upon IP of *Xenopus* or human ZC3HC1 were essentially indistinguishable, as shown in S11G2.

Finally, since truncated versions of the tagged SKP1 polypeptides appeared more efficiently co-immunoprecipitated than the full-length chimeric protein, with the enrichment of such shorter polypeptides amounting to about 5% in some experiments, we wondered whether untagged versions of recombinant SKP1 might represent better binding partners for the ectopically expressed ZC3HC1 polypeptides. We, therefore, performed additional ZC3HC1 IP experiments after having expressed the tagged versions of ZC3HC1 together with tag-free versions of SKP1. However, this did not result in any co-sedimentation of SKP1, or only in hardly detectable trace amounts, as exemplified in S11G3.

Based on these and all earlier results, we finally concluded that ZC3HC1 is not a protein that is a common natural component of an SCF complex and that the interactions observed between certain amounts of the ectopically expressed tagged versions of SKP1 and ZC3HC1 are otherwise explainable, as also addressed in the Supplemental Discussion.

(A) Immunoprecipitation (IP) of naturally occurring soluble forms of xlZC3HC1 from *Xenopus* cell extracts, in search for an interaction with endogenous xlSKP1 or xlCUL1.

(A1) IP of xlZC3HC1 from 250,000 g supernatants of *Xenopus laevis* egg extracts competent of post-mitotic nuclear assembly. The IPs, the stained membrane and the immunoblots for xlZC3HC1, xlTPR, and xlNUP62 are identical to those already presented in Figure 4A. The IP results for TPR and NUP62 are here shown as controls. Additional immunoblots now also presented for xlSKP1 and xlCUL1 were on the lowest part of the Ponceau S-stained membrane shown here and on an identical duplicate, respectively. Lanes had been loaded with an aliquot of the total soluble cell proteins not yet treated with the magnetic immunoaffinity beads (L, for load), with the proteins that had remained unbound after incubation with such beads (U), those released during the third of three successive washing steps (W), and the proteins obtained after final elution (E). Loadings in L and U represented the same volume fraction of the respective samples' total amount (1 V), while the loadings in lanes W and E represented three-fold higher relative amounts (3 V). Cases in which a protein had been co-immunoprecipitated with the IP's actual target protein are framed with brackets in green, while cases in which no co-IP had occurred are accentuated by brackets in magenta. For the blue arrowhead, see Figure 4A. Note that while a subpopulation of TPR polypeptides had been co-immunoprecipitated together with ZC3HC1, and while a large proportion of the extract's total content of ZC3HC1 had similarly been co-immunoprecipitated together with TPR, neither SKP1 nor CUL1 had been co-sedimented together with ZC3HC1. Traces of SKP1 and CUL1 (labelled by asterisks) that had remained attached to the immunoaffinity beads of all IP experiments in similarly minute amounts were only notable after prolonged exposure times (asterisks in magenta).

(A2) IP of the soluble nuclear pool of xlZC3HC1 from the 20,000 g supernatant of manually isolated and then mechanically disrupted *Xenopus* oocyte nuclei. Having used the same supernatant, which in this case barely contained any soluble

NUP62 and similarly only minute amounts of soluble TPR (not shown), a control IP had been conducted in parallel, using magnetic beads as for S11A1 that were coated with antibodies against xlNUP62, in IgG amounts identical to those that had been used for preparing the anti-xlZC3HC1 beads. Lanes were loaded with an aliquot of the total of the proteins present in the nuclei's low-speed supernatant after 20,000 g centrifugation and before incubation with the magnetic immunoaffinity beads (L), with the proteins that had remained unbound after incubation with the beads (U), those released during the third of three successive washing steps (W), and the proteins obtained after final elution (E). Loadings in L and U represented the same volume fraction of the respective samples' total amount (1 V), while the loadings in lanes W and E represented four-fold higher relative amounts (4 V). Immunolabelling for ZC3HC1, CUL1 and SKP1 was on the upper and lower parts of the Ponceau S-stained membrane shown here and on an identical duplicate, respectively. Brackets in magenta mark those cases in which no co-IP had occurred. Note that while essentially all soluble ZC3HC1 from the oocyte's nuclear fraction had been immunoprecipitated, this had not been accompanied by any specific co-IP of SKP1 and CUL1, with only minor amounts of latter similarly attached to the immunoaffinity beads for ZC3HC1 and NUP62.

(A3) IP of the soluble pool of xlZC3HC1 from the 20,000 g supernatant of detergent-permeabilised XL-177 cells. Detergent extraction had been performed in a manner not affecting NB integrity in *Xenopus* oocytes and not causing any conspicuous release of TPR from the NEs of XL-177 cells. We, therefore, regarded the relatively small amount of ZC3HC1 in this type of TPR-free supernatant to primarily have represented a naturally occurring soluble state of ZC3HC1 in this cell line (see also Figure S2B). IP of xlNUP62, using the same supernatant, which here contained a relatively large amount of soluble NUP62, possibly also including solubilised AL materials, had been conducted as a control in parallel. Lanes were loaded with the cells' soluble proteins, obtained after a 20,000 g centrifugation of the detergent-extracted cells, prior to incubation with the magnetic immunoaffinity beads (L), the proteins that had remained unbound after bead incubation (U), those released during the third of three successive washing steps (W), and the proteins obtained after final elution (E). Loadings in T, P, L and U represented the same volume fraction of the respective samples' total amount (1 V), while the loadings in lanes W and E represented five-fold higher relative amounts (5 V). Immunolabelling for ZC3HC1, NUP62, CUL1 and SKP1 was on the upper and lower parts of the Ponceau S-stained membrane shown here and on identical duplicates, respectively. Brackets in magenta mark those cases in which no co-IP had occurred. Note that while essentially all soluble ZC3HC1 that had been released into the XL-177 cell's 20,000 g supernatant had been immunoprecipitated (black arrowhead), as it also held true for all soluble NUP62, this had not been accompanied by any specific co-IP of SKP1 and CUL1.

(B to F) Investigations on the alleged interaction between ZC3HC1 and the SCF complex in mammalian cells.

(B) Triple-labelling IFM of HeLa cells in interphase with antibodies for ZC3HC1 and SCF components. The upper panel of micrographs shows an overview of cells that had been fixed and then permeabilised with TX-100 before immunolabelling. Like for all other specimens to be labelled with CUL1 antibodies for IFM in this study, the FA-fixed and permeabilised cells had subsequently been treated with methanol (-20°C for 10 min), in order to render epitopes recognised by these CUL1 antibodies better accessible, followed by their transfer back into PBS, prior to immunolabelling. Pairs of cells shown in the lower panel on the left side had been fixed with FA before cell permeabilisation with detergents, while those on the right had been briefly permeabilised with detergent before fixation, which removed most of the SKP1 and CUL1 polypeptides present in such tumour cells. The immunolabelled cells shown in this lower panel were then imaged with reduced excitation laser power. The areas marked by rectangles, with the focus on the nuclei's equator, were analysed by ImageJ, allowing for plotting and merging the line profiles for ZC3HC1 with those for SKP1 and CUL1. Note that these analyses did not indicate any colocalisation between the NE-associated ZC3HC1 on the one hand and SKP1 or CUL1 on the other. We found this to hold true all throughout interphase, also after having tested other IFM protocols (our unpublished data). Further note that selections of epifluorescence micrographs shown here were representative for series of very similar results obtained over the course of several years during which these experiments were repeated several times, also with the intention of addressing the issue with different commercial SKP1 and CUL1 antibodies once they became available. In the end, a wide range of commercial antibodies raised in different species had been tested, without having produced any evident labelling for either SKP1 or CUL1 at the NB (data not shown). Bars, 10 μm.

(C) SKP1 and CUL1 RNAi experiments in HeLa cells, analysed by IB and IFM.

(C1) IB of whole-protein extracts from HeLa cells that had been transfected with control siRNAs (CTRL), different pairs of SKP1 or CUL1 siRNAs, or ZC3HC1 siRNAs for further comparison. Single asterisks mark a cross-reaction with a yet unknown protein, labelled with both CUL1 antibodies but apparently unaffected by CUL1 RNAi. Similarly, the double-asterisk marks a cross-reaction of one of the SKP1 antibodies that remained unaffected by SKP1 RNAi. Immunolabelling for the immunoblots shown on the left was performed on uncut membranes comprising the entire length of gel-electrophoretic sample separation, like the representative membranes here shown stained with Ponceau S, to further illustrate each of the antibodies' degree of target-specificity, apart from their target-verification by RNAi. The CUL1-positive band labelled NEDD8-CUL1 represents those CUL1 polypeptides to which the ubiquitin-like protein NEDD8 had apparently been conjugated within the cells, allowing for enhanced cullin-based E3 ligase activity [e.g., 119], with the

degree of neddylation here found especially pronounced in HeLa and less in HEK293T cells (see further below). The upper and lower part of the Ponceau S-stained membrane shown on the right side was first incubated with ZC3HC1 and SKP1, followed by recovering the upper part by quantitatively detaching the antibody through incubation at low pH, upon which it was re-incubated with an antibody for CUL1. Note that the knockdown of neither SKP1 nor CUL1 had notably affected the cellular amount of ZC3HC1 relative to the amount of total cell proteins loaded per lane, demonstrating that ZC3HC1 did not require interaction with SKP1 and the SCF complex in order to be protected from degradation. This finding was at variance with former conclusions of NIPA/ZC3HC1 in interphase cells being protected from degradation only because of its alleged interaction with the SCF complex [e.g., 87].

(C2) Triple-labelling IFM of ZC3HC1 and SCF components in cells that had been treated with either control siRNAs or such targeting ZC3HC1, SKP1, or CUL1, and then harvested at day 3 post-transfection. A few non-transfected cells are shown as a reference. SKP1 and CUL1 presented in the upper and lower micrograph assemblies were each detected with two distinct antibodies yielding similar results. Note, in particular, that knockdown of SKP1 and CUL1 had not affected the presence and immunolabelling intensity of ZC3HC1 at the NE. Similarly, RNAi-mediated depletion of ZC3HC1 had no notable effect on subcellular distribution and labelling intensity of SKP1 or CUL1 either. Bars, 10 µm.

(D) IB of materials obtained from IP experiments with cells extracts containing differently tagged versions of ectopically expressed ZC3HC1, SKP1, or SKP2. Extracts of such cells had been obtained either (i) by sonication in the absence of detergent in NB-s buffers or (ii) by extraction with 0.125% TX-100 in NB-s buffers or (iii) by extraction with 1% TX-100 in an NB-destabilising buffer of identical composition to the corresponding buffer used in former IP studies [33,86,97]. Examples of IPs presented here included FLAG-tagged ZC3HC1 immunoprecipitated with FLAG-tag-specific antibodies and monomeric GFP- or mCherry-tagged ZC3HC1, SKP1, or SKP2 immunoprecipitated with GFP- or RFP-specific sdAbs. For the examples presented here, lanes for SDS-PAGE were always loaded with at least (i) the cell extracts with which the beads had been loaded (L) prior to their incubation with the FLAG-IgG- or sdAb-beads, (ii) the proteins released during the third of three successive washing steps (W), and (iii) the proteins that had remained bound to the beads from which they were released either by quantitative elution with 100 mM glycine pH 2.5 (E), as in the case of some of the FLAG-IP experiments, or by incubation and heating in protein sample buffer (B). In some cases, the proteins that had remained unbound after bead incubation (U) were loaded for comparison. In addition, a distinct amount of BSA was loaded next to the actual IP experiments, as it is here shown on some of the stained membranes, in order to allow for an approximation of the amount of immunoprecipitated recombinant protein.

Furthermore, while cell numbers used for different IP experiments not performed in parallel to each other had differed to some extent, the cell numbers for each cohering set of experiments, consisting of control IPs next to the IPs of target proteins, had been identical, with materials loaded in lanes L and U corresponding to about the same number of cells (1V), while those in lanes W and B corresponded to either 10 (10V) or 20 times (20V) higher numbers. Incubations with antibodies for ZC3HC1, CUL1, and SKP1, and with those for TPR and SKP2 as positive controls, were on the lower and upper parts of the Ponceau S-stained membranes shown here and on duplicates with identical loadings. Green-coloured brackets highlight cases of unambiguous, specific co-IP of a protein with the IP's actual target protein. Lime-green-coloured brackets in S11D2 mark incomplete co-IP of TPR upon incubation in NB-d buffer and black brackets in S11D4 minor amounts of SKP2 co-sedimented with SKP1's short isoform. Brackets in orange frame other cases in which minute beyond-background-level amounts of co-sedimented materials were visible, yet sometimes only after prolonged exposure. No co-sedimentation having occurred beyond background levels is marked by brackets in magenta.

(D1) Simplified schematic depiction of the procedures leading to the IP of tagged versions of ZC3HC1 and other proteins, here exemplified for a mCherry-tagged target following (i) its ectopic expression in HEK293T cells, (ii) the fractionation of such cells by sonication or extraction with the detergent TX-100, (iii) the incubation of the resulting cell extract with immunoaffinity beads, (iv) the separation of unbound proteins and beads, (v) the bead washes and (vi) the final release of the immunoprecipitated proteins into solution.

(D2) IB of materials obtained from IP experiments with anti-FLAG IgG-coated immuno-magnetic beads, following ectopic expression of FLAG-tagged ZC3HC1, and subsequent cell extract preparations and incubations under different conditions. A certain amount of the bead-coupled IgG's heavy and light chains, found to be released from the beads together with the immunoprecipitated ZC3HC1, is marked by asterisks. While varying in amounts, depending on how the bound proteins were eluted from the beads, such heavy and light chain release was also observed in the absence of DTT and ionic detergents and even when having washed the beads rigorously and mock-eluted them with glycine at pH 2.5 before their actual use in some of the IP experiments (data not shown). Such mouse IgG polypeptides, here represented by their asterisk-marked light chains, self-evidently reacted with secondary anti-mouse IgGs, as it had here happened during immunodetection of SKP1 with one of the SKP1 mAbs commonly used for this purpose. Apart from this, note that IP of the tagged ZC3HC1, marked by black arrows on the images of the Ponceau S-stained membranes, had not resulted in any co-sedimentation of endogenous CUL1 when having performed IP experiments in NB-s buffers. Trace amounts of CUL1, hardly above background levels, were if at all only detectable after having performed such IP experiments in an NB-d buffer in the presence of 1% TX-100. Note further that also hardly any of the cells' endogenous SKP1 polypeptides had been co-sedimented together with the immunoprecipitated FLAG-ZC3HC1 polypeptides. However, in contrast to CUL1,

such minute amounts of co-sedimented SKP1 were observed in the IP material of essentially all IP experiments performed with FLAG-ZC3HC1, irrespective of whether an NB-d or an NB-s buffer had been used for the incubation. These amounts varied between clearly less than 0.5% and about 1% of the cells' soluble pool of SKP1. The latter value was, in fact, deducible from the serial dilutions shown on the right side of the two corresponding IP datasets presented. These were from those experiments performed in parallel, in which co-sedimentation of endogenous SKP1 next to the IP of FLAG-ZC3HC1 had reached the highest detectable level in the current study.

Clearly different from these nonetheless minute amounts of SKP1, though, IP of FLAG-ZC3HC1 had resulted in obvious co-IP of soluble TPR. This was especially pronounced in those cases in which the soluble endogenous TPR polypeptides, present in the cell extracts obtained by sonication, had been allowed to interact again with ZC3HC1 in an NB-s buffer. However, even in the presence of the NB-d buffer, some TPR could also be found co-sedimented in varying amounts with FLAG-ZC3HC1, with the result shown here representing the upper limit of such amounts co-sedimented with ZC3HC1 under NB-destabilising conditions. However, with regard to this latter finding, we deem it necessary also to note that we regard certain TRIS-buffered solutions, like the NB-d buffer used here, as disadvantageous for certain experiments with TPR, as they can affect TPR's solubility (our unpublished data). This knowledge had actually kept us from already regarding a result based on such TRIS-containing buffer as evidence for a successful co-IP of TPR.

(D3) IB of materials obtained from IP experiments with anti-GFP or anti-RFP sdAb-coated agarose beads, following ectopic expression of monomeric EGFP- or mCherry-tagged versions of ZC3HC1, subsequent cell extract preparations under different conditions, and incubations and IPs under identical conditions. Note that none of the IPs of these differently tagged versions of ZC3HC1 had resulted in any co-sedimentation of endogenous CUL1 beyond background levels, and similarly, no or hardly any endogenous SKP1 either. Note that this result for the commonly used H363 variant of ZC3HC1 also held true for the R363 variant. By contrast, IP of all of these FP-tagged versions of ZC3HC1, here exemplified by mEGFP-ZC3HC1, had allowed for quantitative co-IP of all the solubilised TPR polypeptides initially present in interphase cell extracts (L) and then absent therefrom (position marked by arrowhead) after incubation (U), as long as such extracts had been prepared in an NB-s buffer.

(D4) IB of materials obtained from IP experiments with anti-RFP sdAb-coated agarose beads, following ectopic expression of mCherry-tagged versions of SKP1, and subsequent cell extract preparations and incubations under NB-stabilising conditions. These results were also representative of the IPs of mEGFP-tagged versions of SKP1 and those for which also differently prepared extracts had been used. The SKP1 isoforms used for the experiments presented here included both the N-terminally and the C-terminally-tagged canonical, i.e., 163 aa-long isoform of SKP1 (here labelled SKP1a), and the N-terminally tagged version of the less common, 160 aa-long isoform (here SKP1b). Note that ZC3HC1 had not been co-sedimented in any amounts above background upon the IP of any of these recombinant SKP1 versions. By contrast, IP of both the N- and C-terminally-tagged canonical isoform of SKP1 had come along with endogenous SKP2 polypeptides having been readily co-immunoprecipitated, whereas such an interaction with SKP2 had hardly occurred with the shorter version of SKP1 (black bracket).

(D5) IB of whole-protein extracts from HeLa cells that had been transfected with control siRNAs or different pairs of SKP2 siRNAs in order to demonstrate target specificity of the SKP2 antibodies used for IB.

(D6) IB of materials obtained from IP experiments with anti-RFP sdAb-coated agarose beads, following ectopic expression of mCherry-tagged versions of SKP2, and subsequent cell extract preparations and incubations under NB-stabilising conditions, with these results being representative also of the IPs of mEGFP-tagged versions of SKP2. Note that ectopic expression of SKP2 in our hands generally had resulted in somewhat lower expression levels than those observed for the other tagged proteins, prompting us to load relatively more of the immunoprecipitated material. Nonetheless, such ectopically expressed SKP2 (arrows) was well capable of interacting with endogenous SKP1, resulting in co-IP of the latter upon IP of SKP2. On the other hand, endogenous ZC3HC1 had been co-sedimented in trace amounts just above background levels only, here marked by orange brackets. The IB of such co-sedimented ZC3HC1 next to serial dilutions of the loaded cell extracts (data not shown) revealed that such trace amounts represented, at most, less than 1% of the cells' naturally occurring soluble ZC3HC1 polypeptides.

(E) RNAi of ERK2/MAPK1 and ERK1/MAKP3, and its effects on the subcellular distribution of ZC3HC1 relative to that of SCF components.

(E1) IB of total cell extracts from HeLa cells that had been transfected with non-target control or different MAPK1 and MAPK3 siRNAs, alone or in combination, and then harvested at day 3 post-transfection. Immunolabelling for MAPK1/3, using two different antibodies both targeting MAPK1 and MAPK3, was performed on uncut membranes comprising the entire length of gel-electrophoretic sample separation, like the representative one shown here stained with Ponceau S, in order to further illustrate target-specificity of the antibodies, apart from their target-verification by RNAi. Immunolabelling for ZC3HC1, CUL1 and SKP1 was performed on additional membranes with identical loadings prepared in parallel. As an aside, note that despite the near-complete loss of MAPK3 and the pronounced reduction of MAPK1, upon RNAi of each MAPK alone or both together, the total amounts of the other immunolabelled proteins had not been notably affected.

(E2-1 to **E2-3)** IFM of ZC3HC1 and SCF components in HeLa cells that had been transfected with either control or MAPK1 siRNAs alone or had been co-transfected with siRNAs targeting MAPK1 and MAPK3 and then harvested at day 3 post-transfection. The cells were triple-labelled with an antibody for ZC3HC1, a second one for either SKP1 (S11E2-1 and S11E2-2) or CUL1 (S11E2-3), and a third one targeting both MAPK1 and MAPK3. The micrographs presented here show only some representative examples. Like in S11B further above, the cells presented in S11E2 had either been fixed before or after permeabilisation with detergent, with the latter procedure again resulting in the removal of most of the SKP1, CUL1 and MAPK1/3 polypeptides. Note the nearly quantitative loss of almost all immunostaining for MAPK1/3 in those cells shown in S11E2-1 and S11E2-3 (some marked by white arrows) that had been transfected with the combination of MAPK1 and MAPK3 siRNAs and then immunolabelled with one of the two commercial MAPKK1/3 antibodies. Note further that such MAPK1/3-deficiency had not resulted in any then more obvious NE-labelling for SKP1 (S11E2-1). Similarly, the absence of MAPK1/3 had not resulted in any apparent NE-association of CUL1 either (S11E2-3).

Furthermore, efficient MAPK1/3 knock-down causing neither SKP1 nor CUL1 to then colocalise with ZC3HC1 at the NE was evident when inspecting exemplifying pairs of cells like those shown in S11E2-2, comprising one cell in which both MAPK1 and MAPK3 had been knocked down by RNAi (here also marked by a white arrow) while the other had remained non-transfected. With the focus on the equator of both nuclei, the area marked by the rectangle was analysed by ImageJ (similar to the images in S11B), resulting in the line profiles for ZC3HC1 and SKP1 in the presence and absence of MAPK1/3. Bars, 10 µm.

(F) IB of materials from IP experiments performed with the extracts of U0126-treated cell populations.

(F1) IB of total cell extracts obtained from HEK293T cells that had been transfected with the indicated expression vectors and then had or had not been treated with U0126 at a concentration of 10µM for 2 hours prior to the cells' harvest. Immunolabelling of the membrane shown here was performed with an antibody specific for the phosphorylated versions of MAPK1/3, reactive for MAPK1 phosphorylated at T185 and Y187, and for MAPK3 phosphorylated at T202 and Y204. Note that after 2 hours of incubation in the presence of U0126, only minor amounts of MAPK1/3 were still phosphorylated. As an aside, standard SDS-PAGE for the immunoblots shown here did not allow for separating MAPK1 from MAPK3 similarly well as in S11E1.

(F2) IB of materials obtained from IP experiments with high-affinity sdAbs, following ectopic expression of differently tagged versions of recombinant proteins, as described for corresponding experiments in S11D, yet here also having used the extracts of cells treated with U0126 at a concentration of 10 µM for 2 hours before the cells' harvest. In contrast to experiments presented in S11D and S11G, these extracts of U0126-treated cells had only been obtained by rupturing the cells by sonication in detergent-free NB-s buffer, which allowed for demonstrating the co-IP of endogenous TPR together with the immunoprecipitated recombinant versions of ZC3HC1. Lanes shown here were loaded with (i) aliquots of these supernatants, stemming from the same cells that in S11F1 were shown to have been successfully treated with U0126, and here representing the actual loading material (L) prior to their incubation with the sdAb-beads, (ii) the proteins released from the beads during the third of three successive washing steps (W), and (iii) those proteins that had remained bound to the beads (B). Materials loaded in lanes L corresponded to about the same number of cells (1V), while those in lanes W and B corresponded to 10 times (10V) higher numbers. Incubations with antibodies for either ZC3HC1, CUL1, or SKP1, and with those for TPR and SKP2 as positive controls, were on the lower and upper parts of the Ponceau S-stained membranes shown here and on duplicates with identical loadings. Classification of the differently coloured brackets corresponds to that in S11D. Note that IP of tagged SKP1 from such U0126-containing cell extracts, once again in large amounts as evident from the Ponceau S-stained membrane, had not resulted in co-sedimentation of the endogenous ZC3HC1 polypeptides in levels above background. By contrast, IP of the same recombinant SKP1 had come along with the co-IP of conspicuous amounts of endogenous SKP2. Further note that IP of ZC3HC1 from the U0126-containing cell extracts had resulted once again in co-IP of endogenous TPR, whereas no CUL1 and hardly any endogenous SKP1 beyond background levels had been co-sedimented. Note also that the results here shown for the full-length ZC3HC1 variant H363 were once again essentially the same as those obtained with the ZC3HC1 variant R363 (data not shown).

(G) IB of materials obtained from IP experiments with cell extracts containing recombinant ZC3HC1, SKP1, and SKP2 in different combinations, after having ectopically co-expressed each two of these proteins as pairs of differently tagged polypeptides or as pairs consisting of a tagged version of the one and an untagged version of the other protein. Extracts of such cells had been obtained either by sonication in the absence of detergent or by extraction with TX-100 in NB-s buffers, yielding similar results, yet with representative examples presented in the following only stemming from IP experiments performed with extracts prepared with the same type of NB-s buffer containing 0.125% TX-100 (S11G1) or 0.15% TX-100 (S11G2 and S11G3). Lanes shown here were loaded with (i) the actual loading material (L) prior to the incubation with the sdAb-beads, (ii) the proteins released during the third of three successive washing steps (W) and (iii) those proteins that had remained bound to the beads (B). Materials loaded in lanes L corresponded to about the same number of cells (1V), while those in lanes W and B corresponded to 20 times (20V) higher numbers. Incubations with antibodies for SKP1, next to some incubations with a GFP antibody to detect the correspondingly tagged protein, were on parts of the Ponceau S- or MemCode-stained membranes shown here, with the latters' images again colour-converted to red. Classification of the differently coloured brackets corresponds to that in S11D.

(G1) IB of materials from IP experiments with cell extracts containing tagged versions of the canonical, 163 aa long isoform of SKP1 (labelled as SKP1a) that had been co-expressed either with differently tagged versions of ZC3HC1 or SKP2. Additional immunoblots were performed in parallel as controls, with (i) the extracts of non-transfected cells and with (ii) the extracts of cells that had been ectopically expressing only one of the tagged proteins, the latter then not being the actual target for the bead-coupled sdAbs used for such mock IPs. Only a selection of these IPs and controls is presented here. Note that IP of the recombinant SKP1 had been accompanied by co-IP of a notable amount of recombinant SKP2, here detected via its GFP tag. By contrast, trace amounts of co-sedimented recombinant ZC3HC1 were again hardly above background levels and only detectable upon prolonged exposure.

(G2) IB of materials from IP experiments with cell extracts containing tagged versions of human or *Xenopus laevis* ZC3HC1 and tagged versions of the canonical, 163 aa long isoform of SKP1. Loading of the immunoprecipitated human ZC3HC1 material was in duplicate to allow for direct lane-to-lane comparison with the control and with the immunoprecipitated *Xenopus* ZC3HC1. Note that IP of both the human and amphibian ZC3HC1 homolog had come along with some co-sedimentation of SKP1, with an apparent preference for the subpopulation of shorter versions of the chimeric FP-SKP1. Such a preference for FP-SKP1 polypeptides lacking either part of their FP or SKP1 sequences was also noted upon the IP of other tagged versions of ZC3HC1 (not shown). Further note that the amounts of SKP1 co-sedimented with human and *Xenopus* ZC3HC1 were essentially the same, even though xlZC3HC1 lacks the proline corresponding to P171 of hsZC3HC1, formerly reported essential for SKP1-binding [33], while human and *Xenopus* SKP1, on the other hand, are identical in sequence. The dilution series presented on the upper right and lower left, with material from two separate IP experiments, allowed for deducing that the amounts of the full-length, tagged SKP1 that had been co-sedimented upon IP of either GFP- or FLAG-tagged ZC3HC1 corresponded to about 0.5% and 1%, respectively, of the total amounts of the full-length versions of the two experiments' ectopically expressed FP-tagged SKP1 present in the loaded extracts. Further note that co-sedimentation of fragments of such recombinant SKP1 polypeptides, one of which is marked by an arrow in orange (right upper dilution series), appeared relatively more pronounced, here corresponding to about 4% of the same fragment's total amount in the loading material. By contrast, endogenous SKP1, on the lower part (black bar) of the same immunoblot shown after shorter and longer exposure times and here marked by black arrows, had not been co-sedimented. As an aside, note that the asterisk (lower left dilution series) marks small amounts of mouse IgG heavy chains released from the anti-FLAG magnetic beads used for this experiment. These heavy chains were immunolabelled with the anti-mouse secondary antibodies used to visualise the mEGFP-SKP1 polypeptides here immunodetected with a mouse monoclonal antibody.

(G3) IB of materials from a selection of IP experiments that had been performed with cell extracts containing differently tagged versions of human or *Xenopus* ZC3HC1, together with the tag-free recombinant version of the canonical, 163 aa long isoform of SKP1 (labelled SKP1a), or the tag-free recombinant version of the shorter SKP1 isoform (labelled SKP1b), or the tagged version of SKP1a for comparison. Arrowheads point at SKP1 polypeptides representing the endogenous pool of SKP1 in the tagged SKP1-containing cell extracts, in which endogenous SKP1 protein levels were the same as in non-transfected HEK293T cells (data not shown), allowing for comparing these amounts with those of the ectopically expressed tag-less SKP1 polypeptides, marked by arrows, in the neighbouring loadings. Like in S11G2, the lower parts of the immunoblots marked by black bars are also shown after long exposure times. Note that while IP of the recombinant human ZC3HC1 polypeptides had come along with some co-sedimentation of the tagged SKP1 polypeptides, with a preference for some of the shorter versions thereof, neither the one nor the other of the tag-free and full-length SKP1 isoforms had been co-sedimented beyond background levels. Similarly, the IP of the *Xenopus* ZC3HC1 polypeptides had not resulted in co-sedimentation of the tag-less SKP1 polypeptides either.

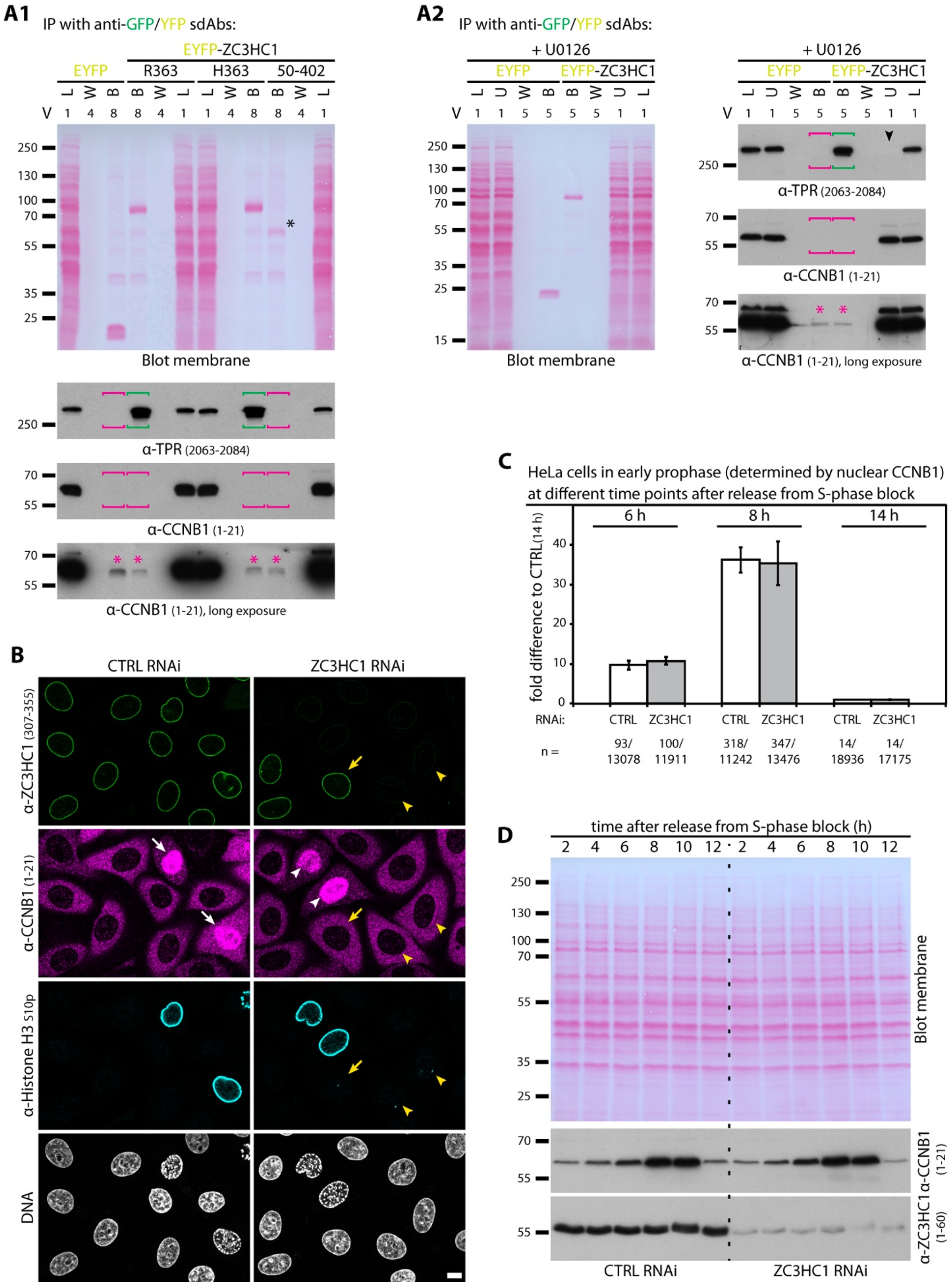
A1
IP with anti-GFP/YFP sdAbs:
EYFP
EYFP-ZC3HC1
R363
H363
50-402
Blot membrane
α-TPR (2063-2084)
α-CCNB1 (1-21)
α-CCNB1 (1-21), long exposure
A2
IP with anti-GFP/YFP sdAbs:
+ U0126
α-TPR (2063-2084)
α-CCNB1 (1-21)
α-CCNB1 (1-21), long exposure
B
CTRL RNAi
ZC3HC1 RNAi
α-ZC3HC1 (307-355)
α-CCNB1 (1-21)
α-Histone H3 S10p
DNA
C
HeLa cells in early prophase (determined by nuclear CCNB1)
at different time points after release from S-phase block
6 h
8 h
14 h
fold difference to CTRL(14 h)
RNAi: CTRL ZC3HC1 CTRL ZC3HC1 CTRL ZC3HC1
n = 93/13078 100/11911 318/11242 347/13476 14/18936 14/17175
D
time after release from S-phase block (h)
2 4 6 8 10 12 2 4 6 8 10 12
Blot membrane
α-CCNB1 (1-21)
α-ZC3HC1 (1-60)
CTRL RNAi
ZC3HC1 RNAi

Supplemental Figure S12. Evidence for ZC3HC1 neither binding CCNB1 nor playing a role in directly regulating the cellular levels of CCNB1 in proliferating cells.

The main function of ZC3HC1 as an SKP1-interacting protein and component of the SCF complex was reported to be the binding of CCNB1 polypeptides that manage to untimely leak into the nucleus in interphase, with ZC3HC1 then promoting their destruction. This, in turn, was reported to prevent premature mitotic entry and cell cycle arrest in prometaphase, especially in tumour cells like HeLa [33,85,86,88, see also 89]. CCNB1 in mitotic cells, on the other hand, was explained to not be degraded because ZC3HC1 would disassemble from the SCF complex [33,85,86] and would be rapidly and quantitatively degraded during mitotic exit. Synthesis of new ZC3HC1 polypeptides would only start again later in G1 [87] while in growth-arrested cells, the amount of the alleged mitotic entry regulator ZC3HC1 was described as being minimal [33]. Accordingly, the elimination of ZC3HC1 by RNAi in proliferating cells was reported to go along with a notable, especially nuclear enrichment of CCNB1 even in G1- and S-phase, and premature mitotic entry. In addition, such ZC3HC1 deficiency was described as being accompanied by prometaphase arrest in most cells [33,85]. Even though we eventually found it not possible to confirm this connection between ZC3HC1 and the SCF complex (see above), we investigated whether ZC3HC1 might nonetheless interact with CCNB1 and affect its cellular fate.

However, immunoprecipitations of endogenous ZC3HC1 and of recombinant, FP-tagged hsZC3HC1 that had been overexpressed in HEK293T cells did not result in any specific co-isolation of CCNB1, despite various attempts that also included variations in extraction buffer compositions. As part of these investigations, we also addressed the possibility that the naturally occurring amino acid substitution at aa position 363 of ZC3HC1 (H363R, e.g., [102]; R363H, e.g., [103]) might affect the relationship between CCNB1 and ZC3HC1. Since it was likely [120], but to us not certain, that the former studies had been conducted with the ZC3HC1 version harbouring the arginine residue at aa 363 [33,86], we also used an additional expression vector for ZC3HC1 that coded for the R363H substitution and then performed the IP experiments with both the H363 and R363 versions of ZC3HC1 in parallel. Again, however, CCNB1 was not found co-immunoprecipitated together with either ZC3HC1 variant. A selection of these findings is presented as immunoblots in S12A1 and S12A2. All of them were in contrast to former reports, in which an excess of GST-hsZC3HC1 had been shown to bind apparently minor amounts of overexpressed, Myc-tagged CCNB1 from HeLa cell extracts [86], and IP of CCNB1 had been presented as resulting in some co-isolation of tagged hsZC3HC1, which had been overexpressed in murine NIH 3T3 cells [33]. In our IP experiments, however, using high-affinity single-domain antibodies (sdAb), neither FP-ZC3HC1 nor ZC3HC1-FP were found to attract CCNB1, even when cells had been treated with MAPK1 siRNAs (our unpublished data) or with U0126 (an example is shown in S12A2). We performed such latter treatments to address the issue of a reported stabilisation of the alleged ZC3HC1-SCF complex [88], which we, however, were not able to confirm either (Figure S11E,F). Moreover, within those materials that had been co-isolated together with the immunoprecipitated FP-ZC3HC1 or ZC3HC1-FP proteins and then subjected to mass spectrometric analysis, we detected an abundance of TPR peptides but not any peptides derived from CCNB1 (our unpublished data).

However, while CCNB1 was known to essentially occur only within the cytoplasm until just before the onset of prophase, even the excess of ZC3HC1 occurring upon overexpression was located mainly within the nucleus. Therefore, we initially regarded it as still imaginable that those amounts of both proteins that might have had a chance to encounter each other within the cell could have been too low to have allowed for the detection of an interaction by IB. Therefore, we repeated the IP experiments also with mutant versions of ZC3HC1 that remained located exclusively within the cytoplasm in interphase. Our design of these mutants was based to some extent on one of the abovementioned former studies in which the alleged CCNB1 binding domain of NIPA/ZC3HC1 had been reported as located between aa 353-402, and in which all deletion mutants still possessing this particular segment had been presented as binding CCNB1 as good as or even better than the full-length NIPA/ZC3HC1 polypeptide [86, and Supplemental Figure 1 therein]. Furthermore, while the NLS of ZC3HC1 had been formerly described as comprising aa 396-402 [120], we found that the arginine residue at position 404 was essential for nuclear import too. Therefore, deletion mutants that lacked aa 404 were cytoplasmic, which in turn allowed for piling up amounts of ZC3HC1 polypeptides within the cytoplasm (our unpublished data) without having to remove or substitute any amino acid between 353-402, i.e., within the alleged, formerly reported, CCNB1-binding domain of NIPA/ZC3HC1.

Following overexpression of such cytoplasmically located ZC3HC1 deletion mutants in HEK293T cells, the EYFP-tagged ZC3HC1 polypeptides were again immunoprecipitated from the cell extracts, using buffer conditions and high concentrations of TX-100 as described earlier [33,86, specified in 97]. In addition, we also applied far less stringent conditions, using NB-s buffers of near-physiological salt concentrations and far less or even no detergent at all, when cells were ruptured by sonication instead. Nonetheless, none of these experiments (exemplified by S12A1) revealed any co-isolation of CCNB1 together with the cytoplasmic versions of ZC3HC1 harbouring the entire region formerly described as the CCNB1 interaction domain.

In addition to such protein interaction studies, we had also investigated in the early phase of our study whether ZC3HC1 deficiency might affect the subcellular distribution of CCNB1 since the main function assigned to ZC3HC1 as an alleged SCF complex component had been reported to be the binding and destruction of CCNB1 polypeptides that manage to untimely leak into the nucleus during interphase [33,85,86]. Moreover, such findings were recurrently underscored [87,88,113] while the current investigation was ongoing. However, before our establishing of CRISPR/Cas9n-edited

ZC3HC1 KO cell lines later in our study, which only then allowed for ultimately clarifying this issue (see Figure 6 and Figure S15D), our early studies regarding the subcellular distribution of CCNB1 in the absence of ZC3HC1 had to be based on RNAi experiments.

At that time, the elimination of ZC3HC1 by RNAi in proliferating cells had been reported to come along with a gradual enrichment of CCNB1 in the nucleus already from S-phase on, and consequently result in premature mitotic entry. However, these former experiments had all relied on only a single ZC3HC1 siRNA pair [33] which apparently was the only one used in later studies too [e.g., 89,113]. Moreover, the conclusions regarding a nuclear accumulation of CCNB1 in the absence of ZC3HC1 were based exclusively on the IB of subcellular fractions and CCNB1 immunoprecipitated from them. To re-approach this issue of the subcellular distribution of CCNB1 at a higher degree of resolution, i.e., at the single-cell level, we studied this by IFM of asynchronous and cell cycle-synchronised populations of ZC3HC1-positive and deficient cells, upon having treated these cells with control siRNAs and different pairs of ZC3HC1 siRNAs (examples shown in S12B). However, this did not reveal any CCNB1 accumulation within the nuclei of ZC3HC1 RNAi cells, compared to the control RNAi cells and those cells within the ZC3HC1 siRNA-treated populations that had remained non-transfected. Nuclear accumulation of CCNB1 was only observed in cells at the verge of exiting G2 and entering prophase, as also evident by characteristic morphological changes of the nucleus, and verified by co-staining with an antibody against phosphorylated serine 10 of histone H3, as a hallmark for the onset of mitosis [e.g., 121–123].

Additionally, such exact timing and effectiveness of nuclear import of the short-lived, 48 kDa CCNB1 protein also demonstrated that the import pathway remained unperturbed in the ZC3HC1-deficient cells. Moreover, since it was already known that CCNB1 polypeptides, prior to their nuclear accumulation in late G2, are actively excluded from the nuclear interior by NES-mediated nuclear export [e.g., 124–126], the nuclear exclusion of CCNB1 in the ZC3HC1-deficient cells in G2 indicated that nuclear export of such protein cargos was still functioning as well. Finally, since the cyclin B1 mRNA was also known to be short-lived in G1 [127], and since ZC3HC1-deficient cells were apparently well capable of synthesising the CCNB1 protein nonetheless, this pointed at renewed synthesis, correct processing of the multi-intron CCNB1 pre-mRNA, and final export of the mRNA also in the absence of ZC3HC1.

Furthermore, our IFM studies on cell cycle-synchronised cell populations performed till then had not revealed any apparent differences regarding the time points at which the control and ZC3HC1-deficient cells were entering mitosis after the release from a thymidine block. However, to analyse this in a more quantitative manner, we evaluated large numbers of IFM micrographs of cultures of control and ZC3HC1-deficient cells harvested at several time points after release from a thymidine-mediated arrest in S-phase. Images from all specimens were taken from several predefined approximate positions near the centre and the perimeter of coverslips, yet without any further bias regarding area selections. Specimens to be evaluated in such a manner were stained with DAPI visualising the different stages of mitosis, with antibodies for ZC3HC1 in order to distinguish between the transfected and non-transfected cells in the ZC3HC1 RNAi experiments, and with antibodies for CCNB1 in order to count those cells in which CCNB1 had been purposefully imported into the nucleus, which happens by importin β /KPNB1-mediated nuclear import at the G2 to M transition [128–130]. These analyses, too, did not reveal any notably earlier onset of mitosis in the populations of ZC3HC1-deficient cells compared to populations that had been treated with control siRNAs and harvested in parallel, as presented in S12C. By contrast, with a few other ZC3HC1 siRNAs, the mitotic onset was even found to be moderately delayed when compared to those cells treated with control siRNAs (our unpublished data), which, however, might reflect some off-target effects caused by these siRNAs. We regard this conclusion as legitimate because the degree of knockdown achieved with these ZC3HC1 siRNAs was not at all better than with the other ZC3HC1 siRNAs not causing such minor additional phenotypes (our unpublished data). Clearly, however, none of our ZC3HC1 RNAi experiments revealed an onset of mitosis that, on average, occurred conspicuously earlier than in cells treated with different control siRNAs.

These IFM studies were complemented by the IB of CCNB1 from total cell extracts obtained from cell cycle-synchronised cell populations, which again had been first transfected with control or ZC3HC1 siRNAs and later harvested at different time points after the release from a thymidine block. This revealed that the gradual accumulation of CCNB1 over time and the total amounts at each time point did not notably differ between the control and ZC3HC1-RNAi populations, as exemplified in S12D.

(A1 and **A2)** IB of materials obtained from IP experiments with sdAb-coated agarose beads, performed as described for corresponding experiments in Figure S11. For all experiments presented here, a population of HEK293T cells had been transfected with a corresponding expression vector for EYFP alone, acting as the background control. For S12A1, additional populations of cells had been transfected with expression vectors for EYFP-ZC3HC1 R363, for EYFP-ZC3HC1 H383 and for an EYFP-ZC3HC1 mutant version that only comprises aa 50-402 and that is incapable of nuclear import and binding to TPR. The total cellular amounts of this primarily cytoplasmic ZC3HC1 mutant (here marked by an asterisk in black) were generally lower than those observed upon overexpression of the WT protein or many other ZC3HC1 mutants. However, whether this might reflect more rapid degradation of ZC3HC1 50-402 or has other reasons was not clarified. For S12A2, the additional cell population had been transfected with the expression vector for EYFP-ZC3HC1 R363, and both populations had then been treated with 10 μM U0126 for 2 hours before their harvest. Lanes shown here were loaded with (i) aliquots of the supernatants, also containing some TPR, obtained after having ruptured the cells in the absence of detergent by sonication in NB-s buffer, with these supernatants representing the actual loading materials (L) prior to their

incubation with the sdAb-beads, (ii) the proteins that had remained unbound (U) after incubation with the beads (here only shown for S12A2), (iii) those proteins released from the beads during the third of three successive washing steps (W) and finally (iv) those that had remained bound to the beads (B). Material loaded in lanes L and U corresponded to about the same number of cells (1V), while those in lanes W corresponded to 4 times (4V) and 5 times (5V), and those in B to 8 times (8V) and 5 times (5V) higher numbers, in S12A1 and S12A2, respectively. Incubations with antibodies for CCNB1 and with those for TPR as a positive control were on the lower and upper parts of the Ponceau S-stained membranes shown here. Cases in which TPR had been co-immunoprecipitated, as the binding partner of the sdAb-bound full-length and not mutated ZC3HC1 polypeptides, are framed with brackets in green. Those in which no co-IP had occurred are accentuated by brackets in magenta. Note that CCNB1 was not found specifically co-isolated with the immunoprecipitated ZC3HC1 in any experiment, with asterisks in magenta marking trace amounts of CCNB1 that could be seen non-specifically attached to all of these sdAb-beads only upon prolonged film exposure. By contrast, the soluble TPR polypeptides had been quantitatively co-depleted from those cell extracts from which EYFP-ZC3HC1 had been immunoprecipitated, as illustrated by its arrow-marked absence in the unbound material shown in S12A2. As an aside, it has no profound meaning that the data shown here represent those obtained with an EYFP-tag, with its tendency to homodimerise weakly, and not the IP data for the proteins also available as tagged with the monomeric GFP version, as shown in S11. Instead, it just reflects the fact that IP experiments for S11 have been far more often repeated over time, both before and after having conducted all experiments for S12, eventually resulting in a broader range of conditions and tags having been tested for S11, in order to conclusively clarify specific points of controversy regarding the alleged relationship between ZC3HC1 and the SCF complex.

(B) IFM of cell cycle-synchronised HeLa cells that had been transfected with non-target control siRNAs (CTRL) or siRNAs targeting ZC3HC1. Three days post-transfection, the cells presented were harvested in the late G2 and early M-phase and then immunolabelled for ZC3HC1 and CCNB1, and with an antibody targeting the phosphorylated serine 10 of histone H3 [H3 phospho-S10; 121], as another hallmark for the onset of mitosis. Control and ZC3HC1-deficient cells demarked by white arrows and arrowheads, respectively, were in early prophase, evident because of (i) chromosome condensation having begun, (ii) phosphorylation of S10 of histone H3 having by then occurred all along the nuclear periphery, and (iii) nuclear accumulation of CCNB1. Note that in the ZC3HC1-positive and ZC3HC1-deficient cells marked by yellow arrows and arrowheads, respectively, first signs of histone H3 S10 phosphorylation were visible, earmarking them as cells that have been fixed only minutes before the onset of nuclear import of CCNB1. Nonetheless, even at this very late time point in G2, the nuclei of ZC3HC1-deficient cells appeared similarly devoid of CCNB1 as they were in the presence of ZC3HC1. Bar, 10 μm.

(C) Diagrams summarising the proportion of HeLa cells in early prophase at 3 days post-transfection with non-target control or ZC3HC1 siRNAs. Specimens harvested at different time points (here exemplified by 6, 8 and 14 h) after the release from a thymidine block were stained with DAPI and labelled for IFM with antibodies for CCNB1 and ZC3HC1. Fluorescence micrographs were acquired from random areas from all across the coverslips, using a 20x objective. Image evaluation was then done by first inspecting only the coded micrographs showing the CCNB1-labelled, as well as DAPI-stained cells of the control and ZC3HC1 RNAi populations, thereby counting all cells as well as only those in which nuclear accumulation of CCNB1 was evident, the latter being the most easily assessable criterion for mitotic entry. Inspection of the corresponding immunolabelling for ZC3HC1 was only done subsequently, i.e., on the then no longer coded images, which allowed for identifying very few cells within the ZC3HC1 siRNA-treated population that had remained non-transfected and at the same time were positive for nuclear CCNB1. We subtracted this small number of CCNB1-positive cells otherwise wrongly ascribed to the ZC3HC1-deficient population, resulting in the presented datasets, with ZC3HC1-depleted cells on the one side versus populations treated with the control siRNAs on the other. Bars represent the standard deviation of three separate evaluations performed by two persons. Numbers n stand for those G2/M cells in which nuclear accumulation of CCNB1 had been obvious, over the total number of inspected cells at each of the time points, with the y-axis representing the fold-difference to the normalised reference point, which in this case was the 14 h value of the control population set to 1. Note that even though we found a minimally higher number of KO than control cells positive for nuclear CCNB1 accumulation at the 6 h time point of this experiment, this result was clearly at variance with formerly reported findings. There, early populations of control cells entering mitosis after release from an S-phase arrest had been depicted as outnumbered by more than tenfold higher numbers of ZC3HC1-deficient cells already in mitosis at the same time point [33]. However, we were not able to find evidence in support of these former results, neither within the here presented data nor by additional cell cycle synchronisation experiments of similar but not identical set-up (data not shown), all of which resulted in findings being similar for both control and ZC3HC1-depleted cell populations. Therefore, we eventually regarded the outcome of our experiments as best summarised by the conclusion that ZC3HC1-deficiency in these cells does not result in a conspicuously earlier onset of mitosis.

(D) IB of loadings of similar amounts of total cell extracts obtained from cell cycle-synchronised populations of HeLa cells that had been transfected with non-target control or ZC3HC1 siRNAs. The cells had been harvested about three days post-transfection at different time points after the release from a thymidine block. Immunolabelling for CCNB1 and ZC3HC1 was performed on the Ponceau S-stained membrane shown here and on a replicate with identical loadings. Note that

gradual CCNB1 accumulation over time, and its total amounts at corresponding time points, did not notably differ between the control and ZC3HC1 siRNA-transfected cell populations. As an aside, and in contrast to former findings [33], also note that the cellular ZC3HC1 amounts did not appear to be largely degraded and to decline late in mitosis precipitously. Instead, towards the end of mitosis and in early G1, here represented by the 12 h time point at which CCNB1 levels were notably lower again, the total amounts of ZC3HC1 did not appear to be notably reduced as compared to those in G2 and earlier in mitosis.

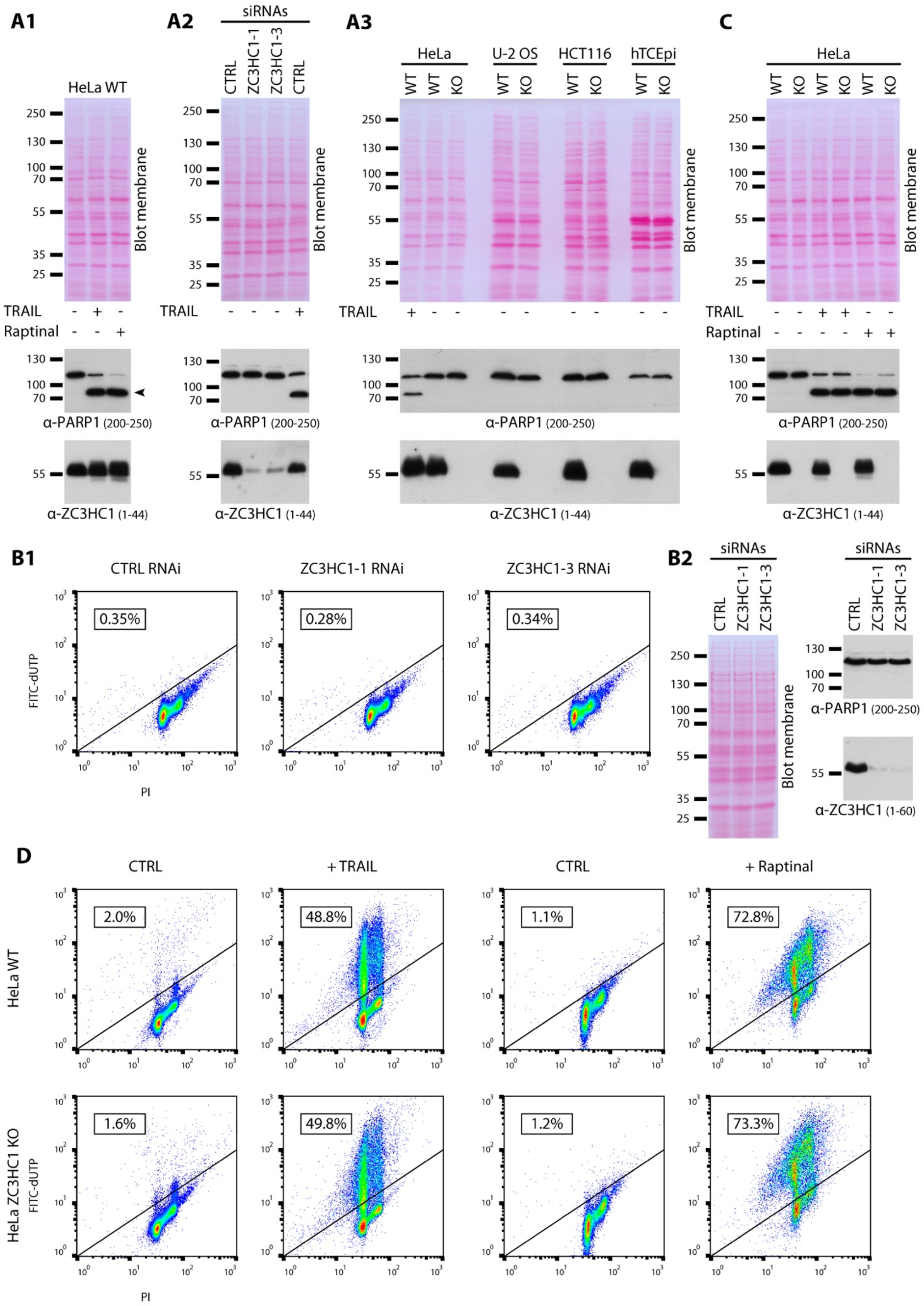

A1
HeLa WT
Blot membrane
TRAIL
Raptinal
α-PARP1 (200-250)
α-ZC3HC1 (1-44)
A2
siRNAs
CTRL
ZC3HC1-1
ZC3HC1-3
A3
HeLa
U-2 OS
HCT116
hTCEpi
WT
KO
C
HeLa
B1
CTRL RNAi
ZC3HC1-1 RNAi
ZC3HC1-3 RNAi
0.35%
0.28%
0.34%
FITC-dUTP
PI
B2
α-ZC3HC1 (1-60)
D
CTRL
+ TRAIL
+ Raptinal
HeLa WT
HeLa ZC3HC1 KO
2.0%
48.8%
1.1%
72.8%
1.6%
49.8%
1.2%
73.3%

Supplemental Figure S13. ZC3HC1 deficiency neither triggers apoptosis in different human cell types nor enhances apoptotic phenotypes upon induction of apoptosis in HeLa cells.

Hitherto, ZC3HC1 knockdown by RNAi in HeLa cells had been described as effectively inducing pronounced apoptosis in the majority of cells, associated with marked caspase-3 activation and DNA fragmentation. This had been proposed to reflect enhanced sensitivity of tumour cells towards ZC3HC1 deficiency [113]. By contrast, we had not observed notably higher numbers of apoptotic cells within populations in which we had knocked down ZC3HC1 by RNAi until then. In fact, having tested collections of ZC3HC1 siRNAs, obtained from different companies for comparison, for knocking down ZC3HC1 in several human cell lines in the early phase of our study, we had found that most of these siRNAs, only a selection of which is presented in Table S3, notably diminished ZC3HC1 mRNA and protein levels without concurrently eliciting apoptotic or non-apoptotic cytotoxic phenotypes. For example, nuclear morphology and appearance of chromatin staining in the ZC3HC1-deficient cells did not suspiciously differ from that in corresponding control cells treated with non-target siRNAs, with the sporadic occurrence of pyknosis and nuclear fragmentation appearing to be similarly rare as in the presence of ZC3HC1.

Moreover, these findings were also in line with our data for TPR in the absence of ZC3HC1. Even though we had noted by then that cellular TPR amounts could be diminished to some extent upon ZC3HC1 RNAi in different human cell lines (see, e.g., Figure 5B) and even though it had been known since long that TPR could be a substrate of the effector caspase-3 [131,132], we could exclude caspase-3 activity as the reason for TPR's reduction in the absence of ZC3HC1 (our unpublished data). In addition, labelling of cells with several antibodies for markers of DNA damage and ongoing DNA repair, like, e.g., γH2AX and 53BP1 had not revealed any increased staining in the ZC3HC1-deficient cells either (our unpublished data).

Furthermore, only a few ZC3HC1 siRNAs had caused a noticeable off-target effect, including an siRNA whose usage resulted in a moderate increase in the number of dead cells. However, such effects not related to the knockdown of the actual target protein had already been commonly observed by then for the one or other siRNA among larger collections of same-target siRNAs [18,133–137, also discussed, e.g., ,138,139].

Nonetheless, despite not having seen any evidence until then that might have hinted at some role in apoptosis, we decided to investigate in further detail whether the absence of ZC3HC1 might directly trigger apoptosis to some extent, particularly in HeLa cells, and whether we might have overlooked some subtle effects so far. These more systematic investigations were first conducted with cells in which we had efficiently knocked down ZC3HC1 with some of those siRNAs that had not caused any notable off-target effects in earlier experiments. Later, after having established cell lines with all *ZC3HC1* alleles disrupted by CRISPR/Cas9n technology (see further below), these too were examined accordingly.

Monitoring of apoptosis was done in various ways, inspecting several of the characteristic hallmarks of an apoptotic cell, yet with our main focus on two of them. First, we studied the fate of the protein poly-(ADP-ribose) polymerase 1 (PARP1), which is a long-known target of apoptotic cleavage [140] and which represents a substrate especially susceptible to rapid degradation by the effector caspase-3 and caspase-7 in HeLa cells [e.g., 131]. In fact, both of these effector caspases, and the initiator caspases for the extrinsic and intrinsic apoptotic pathways, caspase-8 and caspase-9, respectively [e.g., 141], were known by then to exist in sufficient amounts in HeLa [e.g., 142], and both of the main apoptotic pathways had been shown to be functional in this cell line [e.g., 131,132] and in one of its sublines, originally called HeLa P (see Information SI 11) and commonly used in the current study.

In addition, we also studied whether DNA degradation might be more common in the absence of ZC3HC1, which was investigated via "terminal deoxynucleotidyl transferase dUTP nick end labelling" (TUNEL). This approach allowed for the detection of DNA fragments by labelling the 3′-hydroxyl termini of double-strand DNA breaks generated during apoptosis [143]. In the current study, a single-step TUNEL assay was used in which DNA breaks were labelled with FITC-dUTP, followed by flow cytometry analysis. In the following, a selection of such experiments and their representative data are presented.

(A1) Immunoblots demonstrating proper functioning of both the extrinsic and intrinsic apoptotic pathways in the HeLa subline used in the current study. Lanes were loaded with similar amounts of total HeLa cell extracts from proliferating populations and such that had been treated in parallel with the tumour necrosis factor-related apoptosis-inducing ligand (TRAIL/Apo2L), at a concentration of 250 ng/ml for 4 hours, in order to trigger apoptosis via the extrinsic pathway and reveal characteristic apoptotic cleavage of the 113 kDa PARP1 protein [140], and its resulting prominent caspase-3 and caspase-7 cleavage product of 89 kDa (arrowhead). Similarly, cells of experiments conducted in parallel to those with TRAIL were treated with 10 μM Raptinal for 4 hours, in order to trigger apoptosis via the intrinsic pathway that leads to PARP1 cleavage too [e.g., 131,144,145]. Incubations with antibodies for ZC3HC1 and PARP1 were on different parts of the Ponceau S-stained membrane shown here, with this also applying to S13A2 and S13A3.

As an aside, we had initially obtained results that had also allowed for arguing against an anti-apoptotic role for ZC3HC1 in HeLa cells, after having used several other small molecules that trigger apoptosis via the intrinsic pathway (our unpublished data), including, for example, Staurosporine [e.g., 131]. Later, however, having realised that Raptinal, a novel most effective reagent for inducing intrinsic pathway apoptosis [145], allowed for a much more rapid and quantitative degradation of PARP1 in HeLa cells than any of the other reagents we had used until then, we repeated all

apoptosis experiments with Raptinal. This was also done because we had noticed by then that lengthy treatments of different types of cells, including HeLa, with certain types of drugs inhibiting or triggering different pathways, including apoptosis, had led to notable changes in the phosphorylation status of ZC3HC1 and caused pleiotropic effects not directly related to the actually targeted pathway (our unpublished data), with this, in turn, having sometimes complicated data interpretation.

(A2) IB of similar amounts of total cell extracts from HeLa populations that had been transfected with non-target control siRNAs (CTRL) or with different pairs of ZC3HC1 siRNAs and then harvested at day 3 post-transfection. In addition, one batch of cells treated with control siRNAs was additionally treated with TRAIL (CTRL + TRAIL) at 250 ng/ml for 4 hours before harvesting to allow for the emergence of the PARP1 degradation product as the reference. Similar results were obtained with the almost diploid cell line HCT116 (our unpublished data). Note that in the cell extracts of the ZC3HC1 siRNA-treated populations no degradation of PARP-1 was notable, despite efficient knockdown of ZC3HC1.

(A3) IB of total cell extracts from HeLa WT and ZC3HC1 KO strains, next to those from other WT cell lines and their corresponding ZC3HC1 KO strains, once these KO strains, here already shown for comparison, had been generated by CRISPR/Cas9n technology later in this study (see below). Note that PARP1 integrity in the absence of a proapoptotic stimulus did not appear affected by complete KO of ZC3HC1 in these cell lines.

(B1) Levels of DNA fragmentation, as determined by TUNEL assay, within populations of cells that had been transfected with non-target control siRNAs or with different pairs of ZC3HC1 siRNAs and then harvested at day 3 post-transfection. The flow cytometry gating data for HeLa cells treated with control or ZC3HC1 siRNAs are presented as two-parameter density plots, with y-axis values reflecting the degree of FITC-dUTP incorporation and the x-axis DNA staining with propidium iodide (PI), which applies for all plots shown in S13B1 and S13D. Note that this did not reveal any notably increased degree of DNA fragmentation, which otherwise would have manifested itself as an increase in FITC-labelling. These findings were in contrast to a previous study in which the majority of HeLa cells treated with a ZC3HC1 siRNA had been found to be TUNEL-positive [113].

(B2) Portions of the cell populations used for the TUNEL assay presented in S13B1 were used for IB of total cell extracts to control for efficient ZC3HC1 knockdown indeed having occurred.

(C) IB of similar amounts of total HeLa WT and KO cell extracts from proliferating populations, as well as populations treated in parallel either with 250 ng/ml TRAIL or with 10 μM Raptinal for 4 hours each. Note that the degree of PARP1 degradation upon induction of apoptosis via the extrinsic or intrinsic pathway was found to not notably differ between the HeLa WT and ZC3HC1 KO strains.

(D) TUNEL assay experiments conducted like in S13B, yet here with HeLa WT and ZC3HC1 KO cell populations, after treatment as for S13C. Note that the levels of DNA fragmentation, notably increased upon activation of the extrinsic and intrinsic pathway of apoptosis, as reflected by a conspicuous increase in FITC fluorescence, were similar in the presence and absence of ZC3HC1 in HeLa cells.

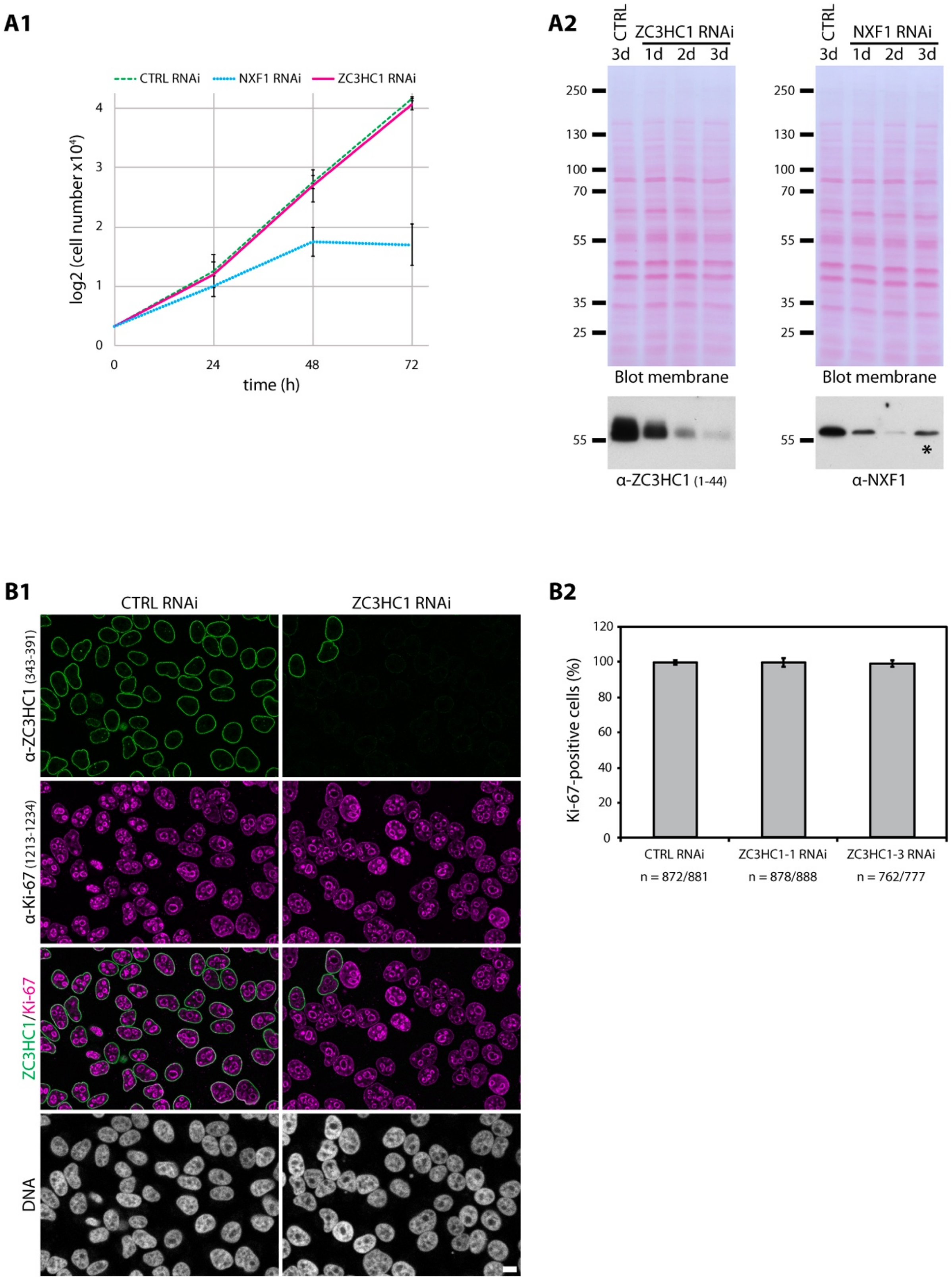
A1
CTRL RNAi
NXF1 RNAi
ZC3HC1 RNAi
log2 (cell number x10⁴)
time (h)
0
24
48
72
A2
CTRL 3d
ZC3HC1 RNAi
1d
2d
3d
NXF1 RNAi
250
130
100
70
55
35
25
Blot membrane
α-ZC3HC1 (1-44)
α-NXF1
*
B1
CTRL RNAi
ZC3HC1 RNAi
α-ZC3HC1 (343-391)
α-Ki-67 (1213-1234)
ZC3HC1/Ki-67
DNA
B2
Ki-67-positive cells (%)
120
100
80
60
40
20
0
CTRL RNAi
n = 872/881
ZC3HC1-1 RNAi
n = 878/888
ZC3HC1-3 RNAi
n = 762/777

S14 (1/4)

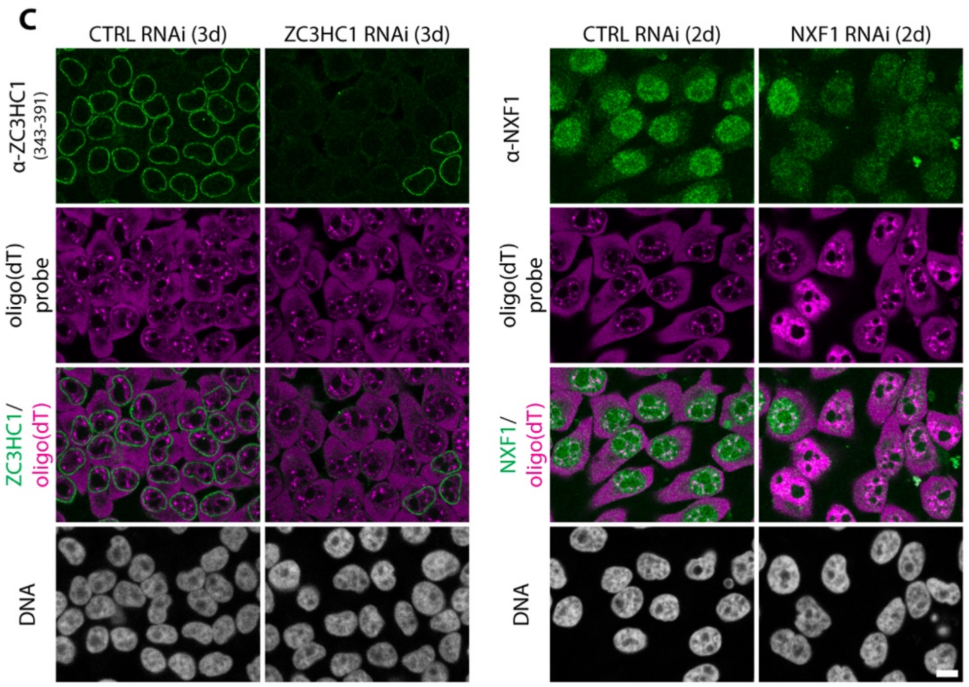
C
CTRL RNAi (3d)
ZC3HC1 RNAi (3d)
CTRL RNAi (2d)
NXF1 RNAi (2d)
α-ZC3HC1 (343-391)
α-NXF1
oligo(dT) probe
ZC3HC1/ oligo(dT)
NXF1/ oligo(dT)
DNA

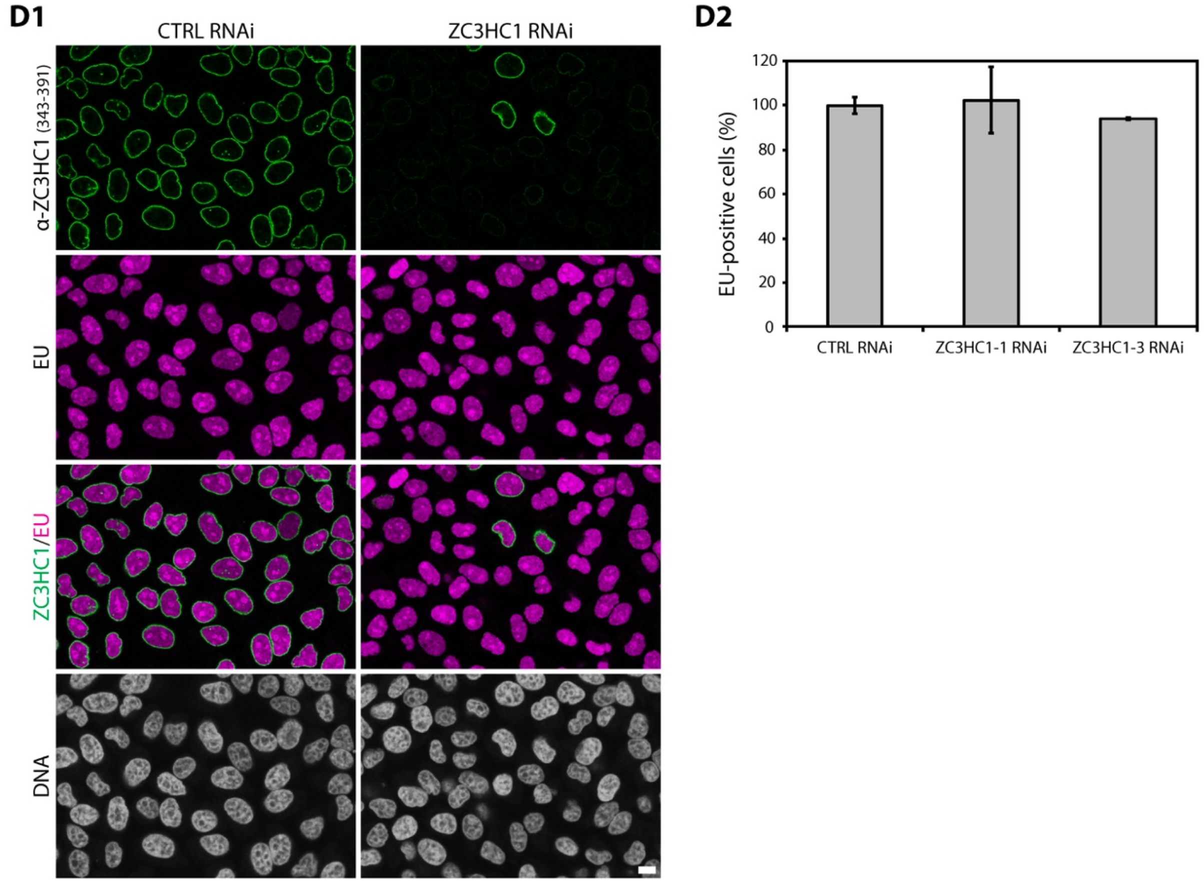
D1
CTRL RNAi
ZC3HC1 RNAi
α-ZC3HC1 (343-391)
EU
ZC3HC1/EU
DNA
D2
EU-positive cells (%)
120
100
80
60
40
20
0
CTRL RNAi
ZC3HC1-1 RNAi
ZC3HC1-3 RNAi

S14 (2/4)

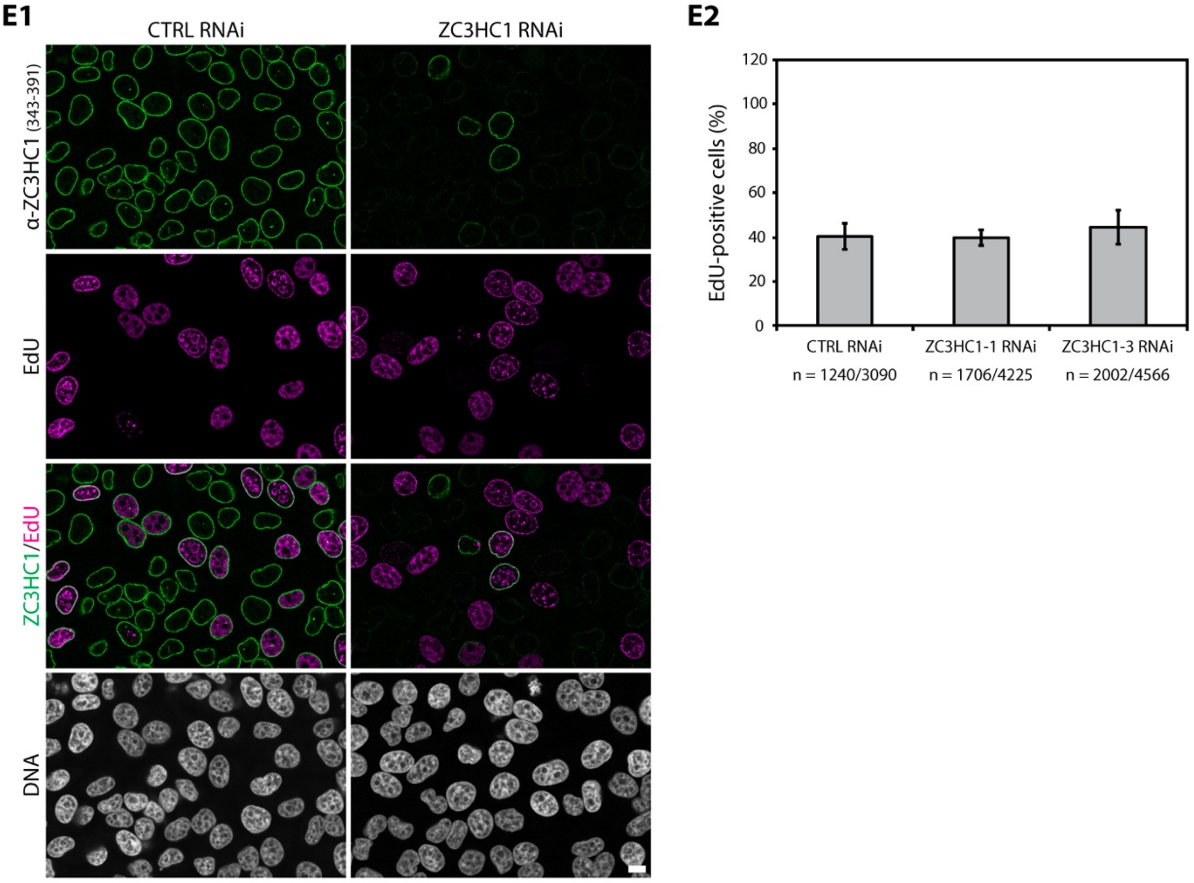
E1
CTRL RNAi
ZC3HC1 RNAi
α-ZC3HC1 (343-391)
EdU
ZC3HC1/EdU
DNA
E2
EdU-positive cells (%)
120
100
80
60
40
20
0
CTRL RNAi
n = 1240/3090
ZC3HC1-1 RNAi
n = 1706/4225
ZC3HC1-3 RNAi
n = 2002/4566

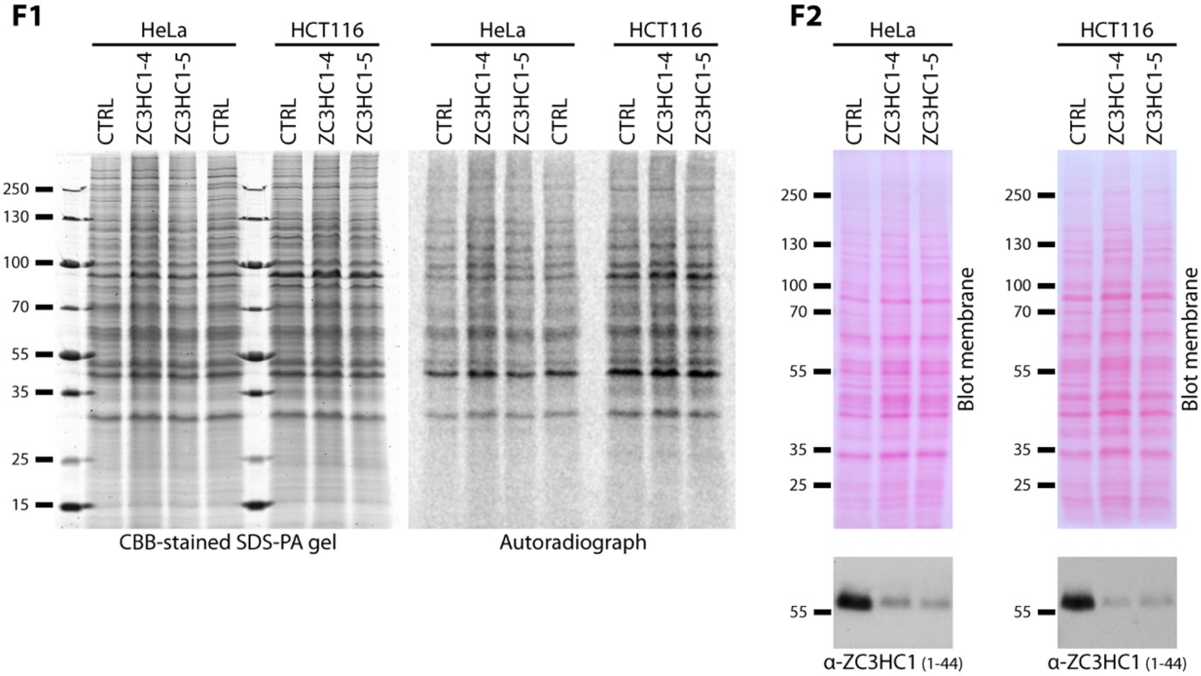
F1
HeLa
HCT116
HeLa
HCT116
CTRL
ZC3HC1-4
ZC3HC1-5
CTRL
250
130
100
70
55
35
25
15
CBB-stained SDS-PA gel
Autoradiograph
F2
HeLa
HCT116
Blot membrane
α-ZC3HC1 (1-44)

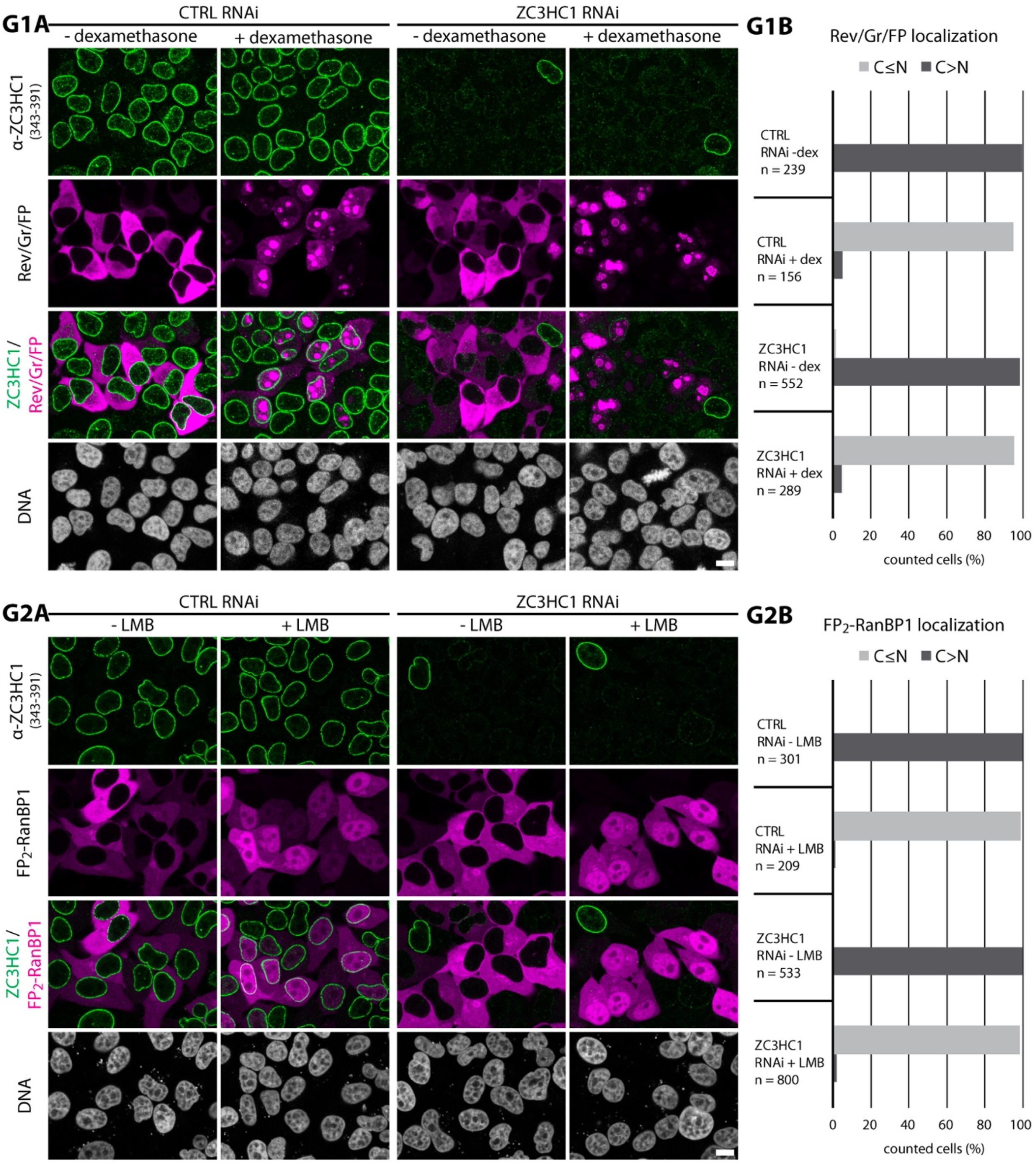

S14 (4/4)

Supplemental Figure S14. ZC3HC1 is not required for cellular housekeeping activities of cultured cells in interphase, with cell culture expansion, subcellular distribution of poly(A)+ RNA, bulk translational activity, bulk transcriptional activity, rates of replication, and the bulk of nucleocytoplasmic transport remaining largely unperturbed in ZC3HC1-deficient cell populations.

At the outset of the current study, reports existing back then had described ZC3HC1 as a nuclear protein essential for cell cycle regulation and proper progression through mitosis of cultured cells, with NIPA/ZC3HC1 deficiency reportedly causing mitotic arrest in prometaphase [33,85,86]. Initially guided by these reports, we had started to address the question as to whether this would-be essential role for ZC3HC1 might also be connected to its location at the NE in interphase, where we had found this protein mainly positioned. Since already some of our early findings, like the presence of conspicuous amounts of ZC3HC1 at the NEs of terminally differentiated and no longer proliferating cells, appeared difficult to reconcile with a concept of ZC3HC1 only being an essential cell cycle regulator, we decided to investigate in more detail whether and how ZC3HC1 deficiency in proliferating cells might affect cellular performance. At that time, before the arising of the CRISPR/Cas9n-technology as a routine procedure, which only then made it possible to conduct

experiments with ZC3HC1 KO cell lines too (e.g., Figure 6), we first performed experiments in which cellular deficiency of ZC3HC1 had been achieved by its RNAi-mediated knockdown.

Some of the earliest of these RNAi experiments already indicated that ZC3HC1 was not required for cell population viability and growth, at least not during the span of time during which cellular ZC3HC1 levels were diminished by RNAi, as will be exemplified in S14A. Furthermore, we had also noted by then that ZC3HC1 deficiency did not impair steady nuclear import of some natural cargos of large mass, such as the 359 kDa-large proliferation marker protein Ki-67 (S14B), and this also held true for at least some cargos of unusually extended size, exemplified by the long rod-shaped, 536 kDa homodimer of TPR itself, which possesses only one NLS per monomer [8,146] and which too apparently was imported into the nucleus in the absence of ZC3HC1, as deducible from results shown in Figure 5. Moreover, having searched for phenotypes that might nonetheless reveal themselves during such a time span, as perhaps gradually increasing effects in parallel to the steadily decreasing cellular amounts of ZC3HC1, we had also found the subcellular distribution of poly(A)+ mRNAs not to have been notably affected (S14C), suggesting that nuclear export of the bulk of such mRNAs did not depend on ZC3HC1 either.

However, even though these experiments had not hinted at ZC3HC1 being an essential protein, we still wondered at that point whether the gradual diminishment of the cellular amounts of ZC3HC1 during a period of only a few days might merely not yet have allowed for phenotypes to manifest themselves conspicuously enough. In fact, we initially did not consider it entirely unlikely that cellular ZC3HC1 amounts might first need to fall below some threshold level for some time before eventually having an impact on cell culture growth and allowing for distinct phenotypes to emerge. However, our attempts to study siRNA-transfected adherent populations of proliferating human tumour cells at time points much later than after three to four days after the initial transfection with siRNAs had turned out to be problematic in our hands at that time. These attempts, which included the splitting and re-seeding of the siRNA-transfected cell cultures, as a necessity for avoiding overcrowding since these cells were not exhibiting contact-inhibition, and their re-transfection with siRNAs, had come along with a range of side-effects also noted with non-target control siRNAs. In retrospect, these can be regarded as having been due to distinct transfection protocol specifications that were common at the beginning of our study. However, since none of these unspecific side-effects had been notable at three days after an initial single round of transfection with siRNAs, when RNAi-mediated diminishment of the cellular ZC3HC1 levels had temporarily reached a minimum, we decided to use this point in time to perform a series of experiments that would provide snapshots of the ZC3HC1-deficient cells' functional capabilities directly at the time point when such ZC3HC1 deficiency was most pronounced. Focussing on some of the essential tasks that need to be fulfilled within a normally proliferating cell, this then included looking at replication, transcription, translation and further aspects of nucleocytoplasmic transport, including nuclear export of proteins. A representative selection of such experiments is compiled in S14D to S14G. Altogether, the results revealed ZC3HC1 as apparently being a protein dispensable for the main housekeeping tasks.

Most of the experiments presented in this Figure S14 were conducted with a HeLa subline called HeLa EM2 [44] that we here call HeLa W. Others were performed with a HeLa subline called HeLa P2, in the following only referred to as HeLa. Both HeLa sublines were unambiguously confirmed by karyotyping and microsatellite analyses to represent progeny of the original HeLa cell line (for further details, see Information SI 11).

(A) Monitoring of HeLa cell population growth following transfection with ZC3HC1 siRNAs.

(A1) Expansions of adherent populations of HeLa W cells, from the time point of having transfected the cells in parallel with representative siRNAs specifically targeting ZC3HC1, with non-target control siRNAs (CTRL), and, for further comparison, with siRNAs targeting the mRNA export factor NXF1/TAP. The siRNAs used here were representative of a collection of siRNAs that we had found not exhibiting any notable off-target effects (our unpublished data). Data points, representing the rate of increase in cell numbers relative to the starting populations, are the mean results from three separate time-course experiments. Of note, treatment with ZC3HC1 siRNAs, known to be capable of already notably reducing cellular ZC3HC1 levels one day after transfection, did not remarkably impair proliferation of the corresponding populations, even after three days, but instead allowed for largely unperturbed cell culture expansion. By contrast, NXF1 RNAi resulted in slowing down population growth.

(A2) IB of total extracts from HeLa W cells that had been transfected with non-target control siRNAs and harvested after three days, and from cells transfected with ZC3HC1 or NXF1 siRNAs and harvested after one, two and three days. Loadings were adjusted to similar total protein amounts per lane. Incubations with antibodies for ZC3HC1 and NXF1 were on the two membranes here shown stained with Ponceau S. In the case of NXF1 RNAi, some re-emergence of NXF1 was sometimes noted after about three days post-transfection (asterisk), which we considered primarily representing the initially small population of non-transfected and thus still proliferating cells whose numbers were gradually increasing.

(B) Monitoring of proliferation marker protein Ki-67 in ZC3HC1-deficient HeLa cell populations. Complementing the early population growth experiments in S14A, we also wanted to assess whether the cells might gradually exit the cell cycle into G0 only from beyond a certain level of ZC3HC1 deficiency on, perhaps only reaching G0 several days post-transfection. In fact, since it had been formerly reported that NIPA/ZC3HC1 expression would be cell cycle-dependent and only minimal once the cells would have exited the cell cycle [33], we wondered whether such reported gradual

decrease in the cellular ZC3HC1 amounts might be a consequence of or rather a cause for cell cycle exit. To gain further insight, we harvested asynchronous populations of HeLa W cells at three days post-transfection with either non-target control siRNAs or ZC3HC1 siRNAs, in order to study by IFM whether the by then highly reduced levels of ZC3HC1 might have come along with some gradual loss of marker proteins for cell proliferation.

(B1) IFM of cells double-immunolabelled for ZC3HC1, in order to distinguish between ZC3HC1-positive and ZC3HC1-deficient cells, and for the proliferation marker protein Ki-67, a 359 kDa protein of short half-life that is known to be present in all cell cycle phases of the cycling cell, but absent in quiescent ones that have exited the cell cycle [e.g., 147–150]. The micrograph of the ZC3HC1 siRNA-treated cell population includes a few cells that have remained non-transfected and are shown as a reference. Note that such immunolabelling for Ki-67 indicated that cell cycle exit events within the populations of ZC3HC1-deficient cells at day 3 post-transfection were very rare, with cells no longer immunopositive for Ki-67 only sporadically detectable (not shown), both within the ZC3HC1 RNAi and control cell populations. Bar, 10 µm, same magnification for all micrographs.

(B2) Diagrams displaying the percentage fraction of cells within a population that were positive for Ki-67 at day 3 post-transfection with non-target control siRNAs or two different pairs of ZC3HC1 siRNAs. Numbers n stand for the Ki-67-positive cells over the total numbers of inspected cells. Specimens had been double-immunolabelled with Ki-67 and ZC3HC1 antibodies, followed by the random acquisition of images and subsequent inspection of the ZC3HC1-positive and ZC3HC1-deficient cells for presence or absence of Ki-67. Altogether, the findings underscored the conclusion that ZC3HC1-deficiency in these experiments did not promote cell cycle exit. Moreover, this type of experiment had also been performed with HCT116 cells, which resulted in a very similar outcome (our unpublished data).

The presence of the short-lived Ki-67 in the majority of ZC3HC1-deficient cells also provided evidence for efficient synthesis and ongoing nuclear protein import of such a large-sized protein at day 3 post-transfection with ZC3HC1 siRNAs. Furthermore, in the course of these growth experiments, we had also noted that there are no conspicuously higher numbers of cells going into apoptosis after transfection with ZC3HC1 siRNAs, as compared to those treated with the non-target control siRNAs. Nonetheless, the issue of whether the absence of ZC3HC1 might trigger apoptosis or intensify apoptotic phenotypes was investigated in further detail (see Figure S13).

(C) Fluorescence microscopy of the subcellular distribution of poly(A)+ RNA in ZC3HC1-deficient cells. In order to monitor the subcellular distribution of the bulk of mRNAs in ZC3HC1-deficient asynchronous HeLa W populations, poly(A)+ RNA was detected via hybridisation with a fluorophore-labelled oligo(dT) probe at day 3 post-transfection with ZC3HC1 or non-target control siRNAs. For further comparison, cells transfected in parallel with siRNAs against NXF1 were harvested already two days post-transfection to avoid losing the majority of NXF1-deficient cells due to cell death and detachment from the coverslip at three days post-transfection. The cells were then analysed by IFM, correlating oligo(dT) distribution with the absence or presence of ZC3HC1, as visualised with antibodies. The micrographs of the ZC3HC1 and NXF1 siRNA-treated population of cells include a few cells that had remained non-transfected and are shown as a reference. Note that poly(A)+ distribution within the ZC3HC1-deficient cells had remained largely unperturbed, being virtually indistinguishable from overall appearance in the controls, indicative that ZC3HC1 is largely dispensable for bulk export of mRNAs in these human cell lines. By contrast, RNAi of the mRNA export factor NXF1 had resulted in an apparent nuclear accumulation of poly(A)+ RNA. Bar, 10 µm.

(D) Visualisation of transcriptional activity and quantification of transcriptionally active cells within populations of ZC3HC1-deficient cells.

(D1) In order to monitor transcriptional activity in ZC3HC1-deficient HeLa W cells at day 3 post-transfection with ZC3HC1 siRNAs, in comparison to cells treated with non-target control siRNAs, asynchronous cell populations were incubated with the fluorophore-conjugated nucleoside analogue 5-ethynyl-uridine (EU) for one hour and then analysed by IFM, correlating the EU signals with absence or presence of ZC3HC1, visualised by antibodies. As usual, in addition to maintaining identical microscope settings for the control and ZC3HC1 RNAi specimens, the chosen micrograph of the ZC3HC1 siRNA-treated population of cells included a few cells as a reference that had remained non-transfected. Note that transcriptional activity appeared to have remained similar in the ZC3HC1-deficient and control cells. Bar, 10 µm.

(D2) In addition, overall fluorescence intensities were quantified spectrophotometrically. To this end, microplate reader measurements for the EU-coupled fluorophore (Alexa Fluor 488) were normalised to DAPI fluorescence to correct for occasional minor differences in cell numbers between wells. Diagram bars represent the mean of three measurements of separate wells for each pair of siRNAs, with the mean EU-A488/DAPI ratios for the ZC3HC1 RNAi populations displayed relative to the mean control value set to 100%. Note that transcriptional activities did not markedly differ between control and ZC3HC1-deficient cell populations.

(E) Visualisation of replicating cells and quantification of cells in S-phase within populations of ZC3HC1-deficient cells.

(E1) In order to monitor replication and the percentage of cells in S-phase in ZC3HC1-deficient HeLa W cells at day 3 post-transfection with ZC3HC1 siRNAs, in comparison to cells treated with non-target control siRNAs, asynchronous cell populations were incubated with the Alexa Fluor 488-conjugated nucleoside analogue 5-ethynyl-2′-deoxyuridine (EdU)

for one hour and then analysed by IFM, correlating the EdU signals with absence or presence of ZC3HC1, visualised with antibodies. As usual, in addition to maintaining identical microscope settings for the control and ZC3HC1 RNAi specimens, the chosen micrograph of the ZC3HC1 siRNA-treated population of cells included a few cells as a reference that had remained non-transfected. Note that similar characteristic patterns of early, intermediate and late replication of chromosomal DNA were observed in both ZC3HC1-deficient and control cells. Bar, 10 µm.

(E2) Diagrams displaying the percentage fraction of EdU-positive cells within a population at three days post-transfection with non-target control or ZC3HC1 siRNAs. In order to determine the proportion of cells within asynchronous populations that had incorporated EdU after a one-hour-long incubation, randomly acquired images were analysed, thereby determining the number n of EdU-positive cells relative to the respective total number of cells in each dataset. In addition to EdU incorporation, cells had been immunolabelled for ZC3HC1 to identify those cells within the ZC3HC1-siRNA-treated populations that had remained non-transfected. This allowed for subtracting a small number of EdU counts assigned to ZC3HC1-positive cells, resulting in the presented datasets, with only ZC3HC1-deficient cells on the one side versus those treated with control siRNAs on the other. Bars represent the mean of three separate evaluations performed in parallel. Note that the proportion of cells being Edu-positive within the populations of ZC3HC1-deficient cells and within those treated with the non-target siRNAs was very similar, which self-evidently meant that the proportion of cells being in S-phase within such asynchronous populations was very similar too.

(F) Translational activity within populations of ZC3HC1-deficient cells.

(F1) In order to monitor translational activities in ZC3HC1-deficient HeLa W cell populations at day 3 post-transfection with ZC3HC1 siRNAs, in comparison to cells treated with non-target control siRNAs, asynchronous populations of ZC3HC1-positive and -deficient HeLa and HCT116 cells were incubated for 3 hours in medium containing ^{35}S-methionine, followed by separating total cell proteins on an SDS-polyacrylamide (SDS-PA) gel by SDS-PAGE, and subsequent staining with Coomassie Brilliant Blue (CBB). This was followed by drying the gel and exposing it to an X-ray film, with the autoradiograph shown here. Note that translational activity at the time point of harvest, reflected by the degree of ^{35}S-methionine incorporation as shown by the autoradiograph, relative to the total amount of proteins as shown in the CBB-stained SDS-PA gel, did not differ notably between the control and ZC3HC1-diminished cell populations.

(F2) IB for ZC3HC1, using the same radioactive total cell extracts as for S14F1, demonstrating that ZC3HC1 knockdown had indeed occurred. As an aside, note that while protein banding patterns of HeLa and HCT116 cell extracts are generally of very similar appearance when electrophoretically separated by the SDS-PAGE system [151] commonly used in the present study, recurrent analysis of the corresponding cell lines by karyotyping and microsatellite analysis unambiguously confirmed these cell lines being of HeLa and HCT116 origin.

(G) Monitoring of nuclear import and export of different kinds of reporter proteins in ZC3HC1-deficient cells. Circumstantial evidence had already indicated at this time point that ZC3HC1 residing at the TR of the NB was not required for nuclear import and release of large import cargos, like Ki-67 (Figure S14B1) or TPR (Figure 5), from the NB into the nuclear interior. Furthermore, we had also been able to conclude that nuclear export and import of CCNB1 was functioning well in the absence of ZC3HC1 (Figure S12). Nonetheless, to further scrutinise whether ZC3HC1 might play a role in nucleocytoplasmic transport, we made use of established GFP-based reporter systems developed by others for studying nuclear import and export of average-sized proteins, namely an approximately 64 kDa chimeric protein comprising a GFP-tagged HIV-1 Rev protein fused to the hormone-responsive element of the glucocorticoid receptor [Rev/Gr/GFP, 152] and a shuttling GFP-GFP-RanBP1 (GFP_2-RanBP1) fusion protein of about 75 kDa [153]. While constitutively expressed, the actual translocation of these reporter proteins either into or out of the nucleus can be elicited or impaired at any time point, and thus also several days after the initial transfections with siRNAs. Therefore, these reporters allowed for assessing current pathway performance at time points when an ultimate level of target protein knockdown had already been reached. In the following, the fluorescence of Rev/Gr/GFP and of GFP_2-RanBP1 is shown false-coloured in magenta (Rev/Gr/FP in S14G1A and FP-RanBP1 in S14G2A, respectively) while images of ZC3HC1 labelled with Cy3-coupled secondary antibodies are false-coloured in green.

(G1) Hormone-induced nuclear import of the chimeric Rev/Gr/GFP protein was not impaired by ZC3HC1 deficiency. HeLa W cells transfected first with siRNAs were transfected 55 hours later with the expression vector pXRGG. Again 17 hours later, i.e., at three days post-transfection with the siRNAs, the cells were treated either with or without 1 µM dexamethasone for 60 min at 37°C before fixation.

(G1A) Cells were then studied by fluorescence microscopy, in combination with IFM using ZC3HC1 antibodies to distinguish ZC3HC1-positive from ZC3HC1-deficient cells. Images showing ZC3HC1-deficient cells were selected to include also one of the rare non-transfected cells to allow for assessing the degree of knockdown in the other cells. Note that while the reporter protein resided within the cytoplasm in the absence of hormone, it near-quantitatively translocated upon steroid treatment into most of the ZC3HC1-deficient and control cells' nuclei. Bar, 10µm.

(G1B) Diagrams displaying the percentage fraction of cells in which the Rev/Gr/GFP reporter protein, in the absence or presence of dexamethasone and ZC3HC1, was found primarily located in one or the other subcellular compartment.

Numbers n stand for the total of transfected cells within randomly photographed areas of the respective coverslips that have been evaluated per experiment.

(G2) Nuclear export of GFP_2-RanBP1, inhibitable by CRM1/XPO1-inhibitor leptomycin B (LMB), was not impaired by ZC3HC1-deficiency. Cells transfected first with siRNAs were 55 hours later transfected with the expression vector pK-GFP_2-RanBP1. Again 17 hours later, the cells were treated either with or without 20 nM LMB for 60 min at 37°C before fixation.

(G2A) Cells were then studied by fluorescence microscopy in combination with IFM for ZC3HC1. Note that the GFP-tagged shuttling protein RanBP1, naturally exhibiting a steady-state cytoplasmic localisation, accumulated within the nucleus upon LMB treatment, revealing ongoing nuclear protein import and indicating functioning CRM1-mediated export that could be stalled by LMB, independent of whether ZC3HC1 was absent or not. As an aside, note that the DAPI-stained foci seen outside of the cells in S14G1A and S14G2A represent plasmid DNA that had either not been taken up by the cells or had not been transported into the nuclei. Bar, 10 µm.

(G2B) Diagrams displaying the percentage fraction of cells in which the GFP_2-RANBP1 reporter protein, in the absence or presence of LMB and ZC3HC1, was found primarily located in one or the other subcellular compartment. Numbers n stand for the total of transfected cells within randomly photographed areas of the respective coverslips that have been evaluated per experiment.

Of particular note, all these findings based on ectopically expressed reporter proteins not only indicated that ZC3HC1 was largely dispensable for nuclear import and export of such distinct proteins but also that ZC3HC1 was not required for the export of any of the corresponding reporter protein-encoding transcripts, all of which stemmed from intron-less cDNA sequences and first needed to be exported from the nucleus before allowing for subsequent reporter protein synthesis.

S15 (1/6)

A1

human *ZC3HC1* gene
7q32.2 (33,166 bases)

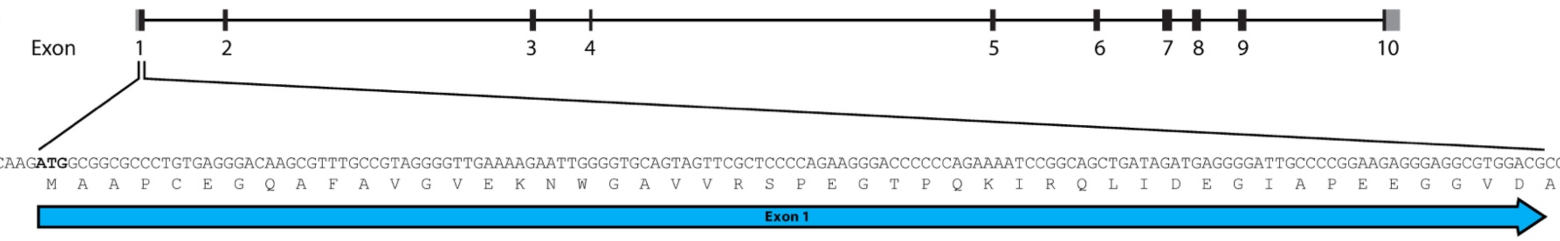

A2

dsDNA target sites of sgRNAs of pair 1

```
                                                                                                               CUUUUAGGCCGTCGACUAUC
5′   CGGCAGCGGACCAAGATGGCGGCGCCCTGTGAGGGACAAGCGTTTGCCGTAGGGGTTGAAAAGAATTGGGGTGCAGTAGTTCGCTCCCCAGAAGGGACCCCCCAGAAAATCCGGCAGCTGATAGATGAGGGGATTGCCCCGGAAGAGGGAGGCGTGG
3′   GCCGTCGCCTGGTTCTACCGCCGCGGGACACTCCCTGTTCGCAAACGGCATCCCCAACTTTTCTTAACCCCACGTCATCAAGCGAGGGGTCTTCCCTGGGGGGTCTTTTAGGCCGTCGACTATCTACTCCCCTAACGGGGCCTTCTCCCTCCGCACC
                                                                                                                                      GAGGGGAUUGCCCCGGAAGA
```

dsDNA target sites of sgRNAs of pair 2

```
                           ACACUCCCUGUUCGCAAACG
5′   CGGCAGCGGACCAAGATGGCGGCGCCCTGTGAGGGACAAGCGTTTGCCGTAGGGGTTGAAAAGAATTGGGGTGCAGTAGTTCGCTCCCCAGAAGGGACCCCCCAGAAAATCCGGCAGCTGATAGATGAGGGGATTGCCCCGGAAGAGGGAGGCGTGG
3′   GCCGTCGCCTGGTTCTACCGCCGCGGGACACTCCCTGTTCGCAAACGGCATCCCCAACTTTTCTTAACCCCACGTCATCAAGCGAGGGGTCTTCCCTGGGGGGTCTTTTAGGCCGTCGACTATCTACTCCCCTAACGGGGCCTTCTCCCTCCGCACC
                                                                         GCAGUAGUUCGCUCCCCAGA
```

A3

```
Wildtype        CGGCAGCGGACCAAGATGGCGGCGCCCTGTGAGGGACAAGCGTTTGCCGTAGGGGTTGAAAAGAATTGGGGTGCAGTAGTTCGCTCCCCAGAAGGGACCCCCCAGAAAATCCGGCAGCTGATAGATGAGGGGATTGCCCCGG-AAGAGGGAGGCGTG
HeLa P2 KO #1   CGGCAGCGGACCAAGATGGCGGCGCCCTGTGAGGGACAAGCGTTTGCCGTAGGGGTTGAAAAGAATTGGGGTGCAGTAGTTCGCTCCCCAGAAGGGACCCCCCAGAAAATCCGGCAGCTGATAGATGAGGGGATTGCCCCGGGGAGAGGGAGGCGTG
HeLa P2 KO #2   CGGCAGCGGACCAAGATGGCGGCGCCCTGTGAGGGACAAGCGTTTGCCGTAGGGGTTGAAAAGAATTGGGGTGCAGTAGTTCGCTCCCCAGAAGGGACCCCCCAGAAAATCCGGCAGCTGATAGATGAGG----------AG-AAGAGGGAGGCGTG
HeLa P2 KO #3   CGGCAGCGGACCAAGATGGCGGCGCCCTGTGAGGGACAAGCGTTTGCCGTAGGGGTTGAAAAGAATTGGGGTGCAGTAGTTCGCTCCCCAGAAGGGACCCCCCAGAAAATCCGG----------------GGATTGCCCCGG-AAGAGGGAGGCGTG

Wildtype        CGGCAGCGGACCAAGATGGCGGCGCCCTGTGAGGGACAAGCGTTTGCCGTAGGGGTTGAAAAGAATTGGGGTGCAGTAGTTCGCTCCCCAGAAGGGACCCCCCAGAAAATCCG________________________________GCAGCTGATAGA
HCT116 KO      CGGCAGCGGACCAAGATGGCGGCGCCCTGTGAGGGACAAGCGTTTGCCGTAGGGGTTGAAAAGAATTGGGGTGCAGTAGTTCGCTCCCCAGAAGGGACCCCCCAGAAAATCCGGCAGCTGATAGATGAGGGGATTGCAGCTGATAGCAGCTGATAGA

Wildtype        CGGCAGCGGACCAAGATGGCGGCGCCCTGTGAGGGACAAGCGTTTGCCGTAGGGGTTGAAAAGAATTGGGGTGCAGTAGTTCGCTCCCCAGAAGGGACCCCCCAGAAAATCCGGCAGCTGATAGATGAGGGGATTGCCCCGGAAGAGGGAGGCGTGG
U-2 OS KO #1    CGGCAGCGGACCAAGATGGCGGCGCCCTGTGAGGGACA-------------------------------------GTAGTTCGCTCCCCAGAAGGGACCCCCCAGAAAATCCGGCAGCTGATAGATGAGGGGATTGCCCCGGAAGAGGGAGGCGTGG
U-2 OS KO #2    CGGCAGCGGACCAAG--------------------------------------------------TTCCTCCCCAGTAGTTCGCTCCCCAGAAGGGACCCCCCAGAAAATCCGGCAGCTGATAGATGAGGGGATTGCCCCGGAAGAGGGAGGCGTGG

Wildtype        CGGCAGCGGACCAAGATGGCGGCGCCCTGTGAGGGACAAGCGTTTGCCGTAGGGGTTGAAAAGAATTGGGGTGCAGTAGTTCGCTCCCCAGAAGGGACCCCCCAGAAAATCCGGCAGCTGATAGATGAGGGGATTGCCCCGGAAGAGGGAGGCGTGG
hTCEpi KO       CGGCAGCGGACCAAGATGGCGGCGCCCTGTGAGGGACAAGCGTTTGCCGTA-------------------------------------------GGACCCCCCAGAAAATCCGGCAGCTGATAGATGAGGGGATTGCCCCGGAAGAGGGAGGCGTGG
```

A4

```
                Exon 1                                            Exon 2                                    Exon 3
Wildtype        MAAPCEGQAFAVGVEKNWGAVVRSPEGTPQKIRQLIDEGIAPEEGGVDA   KDTSATSQSVNGSPQAEQPSLESTSKEAFFSRVETFS     SLKWAGKPFELSPLVCAKYGWVTVECDMLKCSSCQAFLCASLQPAFDFDR
HeLa P2 KO #1   MAAPCEGQAFAVGVEKNWGAVVRSPEGTPQKIRQLIDEGIAPGRGRRGR   EGHVCHIPVS.
HeLa P2 KO #2   MAAPCEGQAFAVGVEKNWGAVVRSPEGTPQKIRQLIDE    EKREAWTR   RTRLPHPSQLMDHPKRNNLHWNLQAKKPSLAEWKHFL     L.
HeLa P2 KO #3   MAAPCEGQAFAVGVEKNWGAVVRSPEGTPQKIR      GLPRKREAWTR   RTRLPHPSQLMDHPKRNNLHWNLQAKKPSLAEWKHFL     L.

Wildtype        MAAPCEGQAFAVGVEKNWGAVVRSPEGTPQKIRQLIDEGIAPEEGGVDA   KDTSATSQSVNGSPQAEQPSLESTSKEAFFSRVETFS     SLKWAGKPFELSPLVCAKYGWVTVECDMLKCSSCQAFLCASLQPAFDFDR
HCT116 KO       MAAPCEGQAFAVGVEKNWGAVVRSPEGTPQKIRQLIDEGIAADSS.

Wildtype        MAAPCEGQAFAVGVEKNWGAVVRSPEGTPQKIRQLIDEGIAPEEGGVDA   KDTSATSQSVNGSPQAEQPSLESTSKEAFFSRVETFS     SLKWAGKPFELSPLVCAKYGWVTVECDMLKCSSCQAFLCASLQPAFDFDR
U-2 OS KO #1    MAAPCEGQ.
U-2 OS KO #2    no start codon

Wildtype        MAAPCEGQAFAVGVEKNWGAVVRSPEGTPQKIRQLIDEGIAPEEGGVDA   KDTSATSQSVNGSPQAEQPSLESTSKEAFFSRVETFS     SLKWAGKPFELSPLVCAKYGWVTVECDMLKCSSCQAFLCASLQPAFDFDR
hTCEpi KO       MAAPCEGQAFAVGPPRKSGS.
```

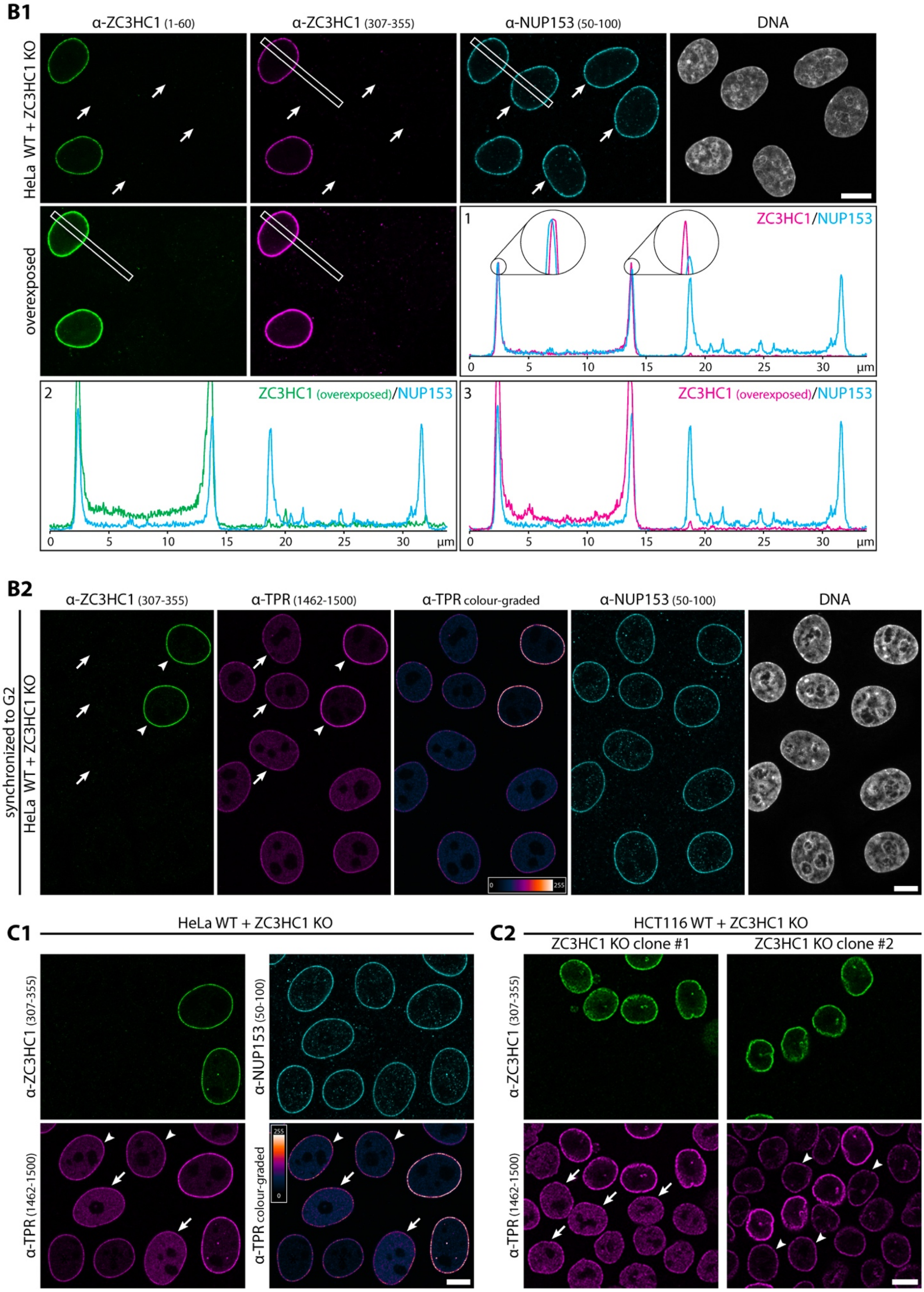
B1
α-ZC3HC1 (1-60)
α-ZC3HC1 (307-355)
α-NUP153 (50-100)
DNA
HeLa WT + ZC3HC1 KO
overexposed
1
ZC3HC1/NUP153
2
ZC3HC1 (overexposed)/NUP153
3
ZC3HC1 (overexposed)/NUP153
µm
B2
α-ZC3HC1 (307-355)
α-TPR (1462-1500)
α-TPR colour-graded
α-NUP153 (50-100)
DNA
synchronized to G2
HeLa WT + ZC3HC1 KO
C1
HeLa WT + ZC3HC1 KO
α-ZC3HC1 (307-355)
α-NUP153 (50-100)
α-TPR (1462-1500)
α-TPR colour-graded
C2
HCT116 WT + ZC3HC1 KO
ZC3HC1 KO clone #1
ZC3HC1 KO clone #2

S15 (2/6)

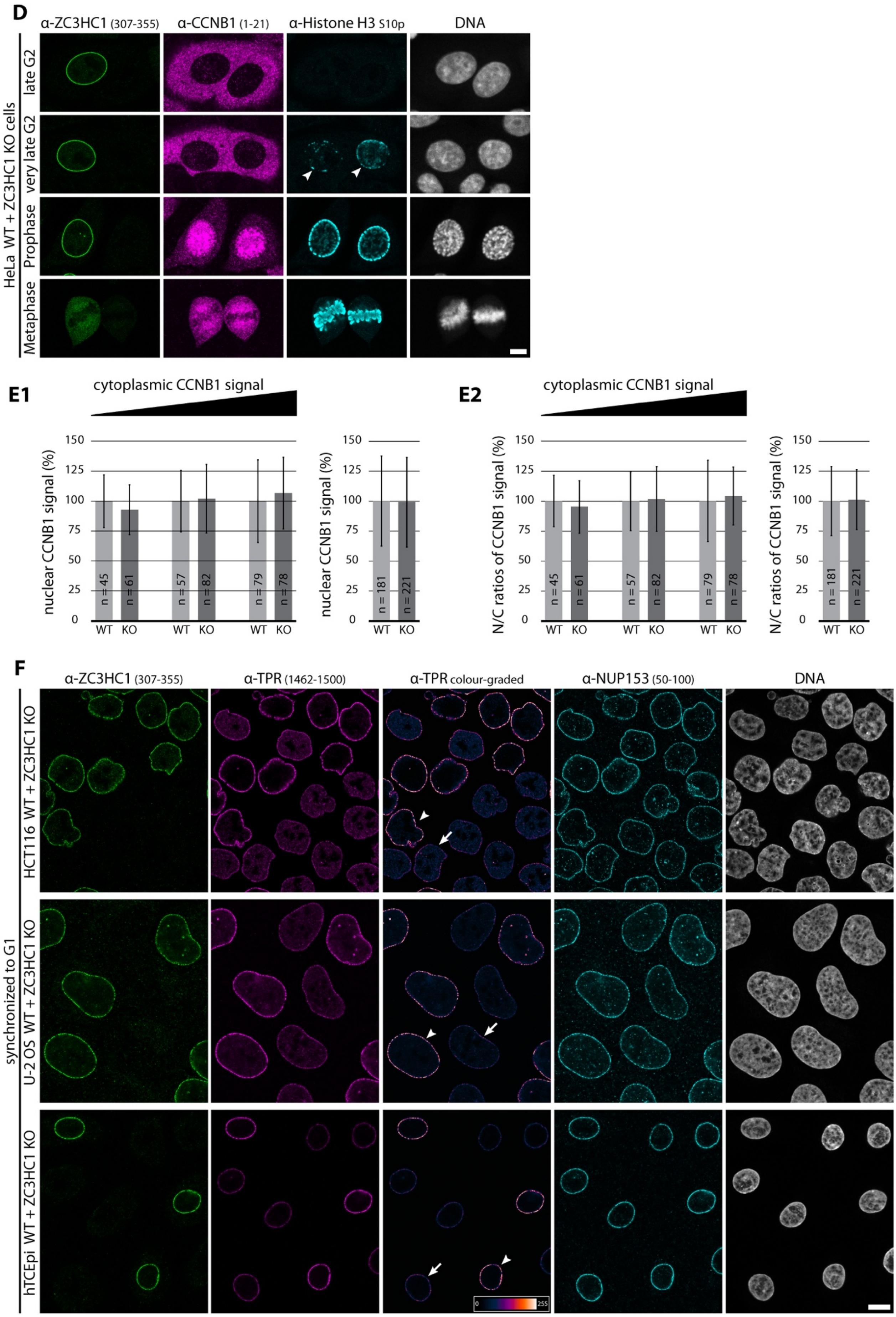
D
α-ZC3HC1 (307-355)
α-CCNB1 (1-21)
α-Histone H3 S10p
DNA
HeLa WT + ZC3HC1 KO cells
late G2
very late G2
Prophase
Metaphase
E1
cytoplasmic CCNB1 signal
nuclear CCNB1 signal (%)
n = 45
n = 61
n = 57
n = 82
n = 79
n = 78
n = 181
n = 221
WT KO
E2
N/C ratios of CCNB1 signal (%)
F
α-ZC3HC1 (307-355)
α-TPR (1462-1500)
α-TPR colour-graded
α-NUP153 (50-100)
DNA
synchronized to G1
HCT116 WT + ZC3HC1 KO
U-2 OS WT + ZC3HC1 KO
hTCEpi WT + ZC3HC1 KO
0
255

S15 (3/6)

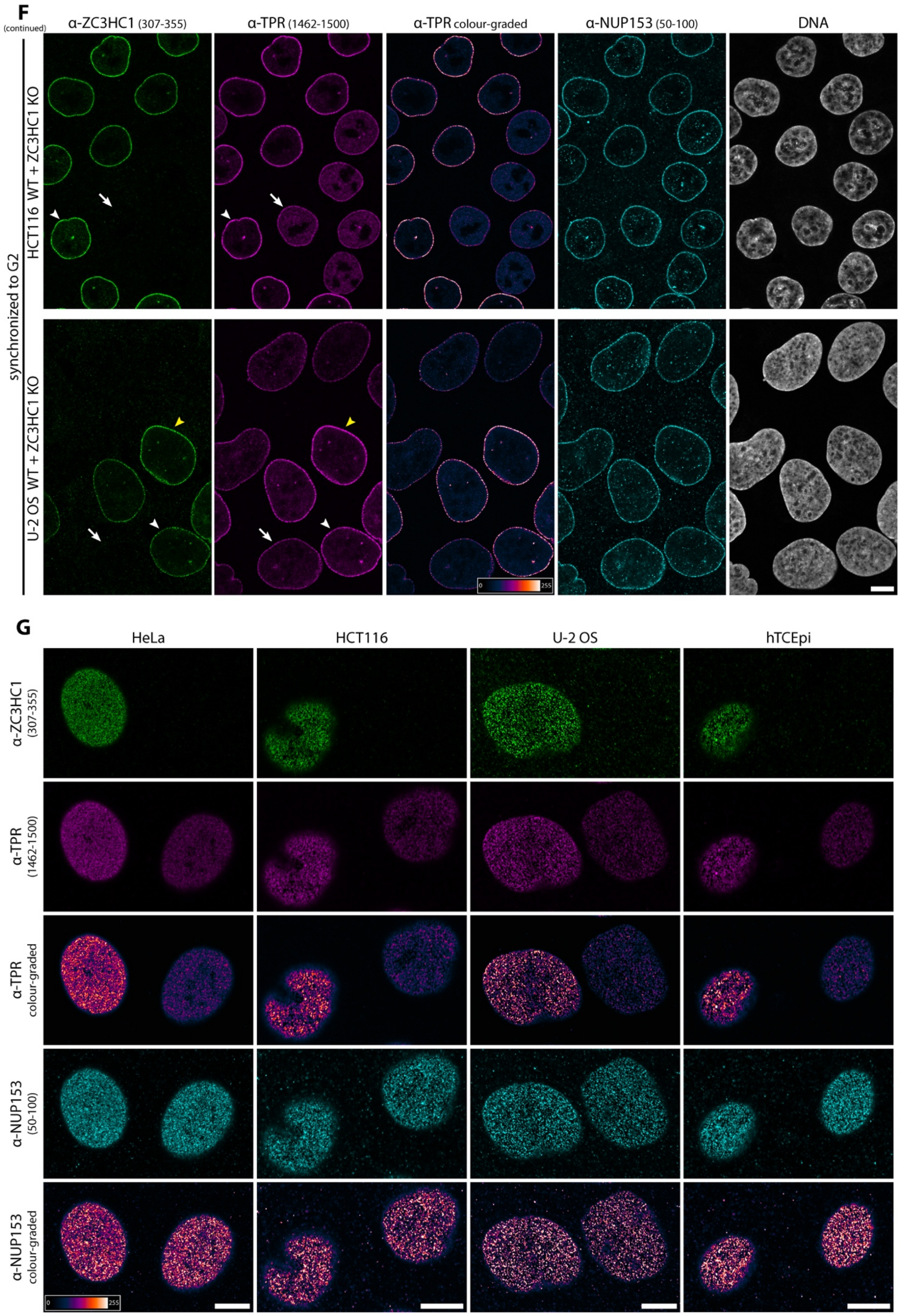
F
(continued)
α-ZC3HC1 (307-355)
α-TPR (1462-1500)
α-TPR colour-graded
α-NUP153 (50-100)
DNA
synchronized to G2
HCT116 WT + ZC3HC1 KO
U-2 OS WT + ZC3HC1 KO
0
255
G
HeLa
HCT116
U-2 OS
hTCEpi
α-ZC3HC1 (307-355)
α-TPR (1462-1500)
α-TPR colour-graded
α-NUP153 (50-100)
α-NUP153 colour-graded
0
255

S15 (4/6)

H

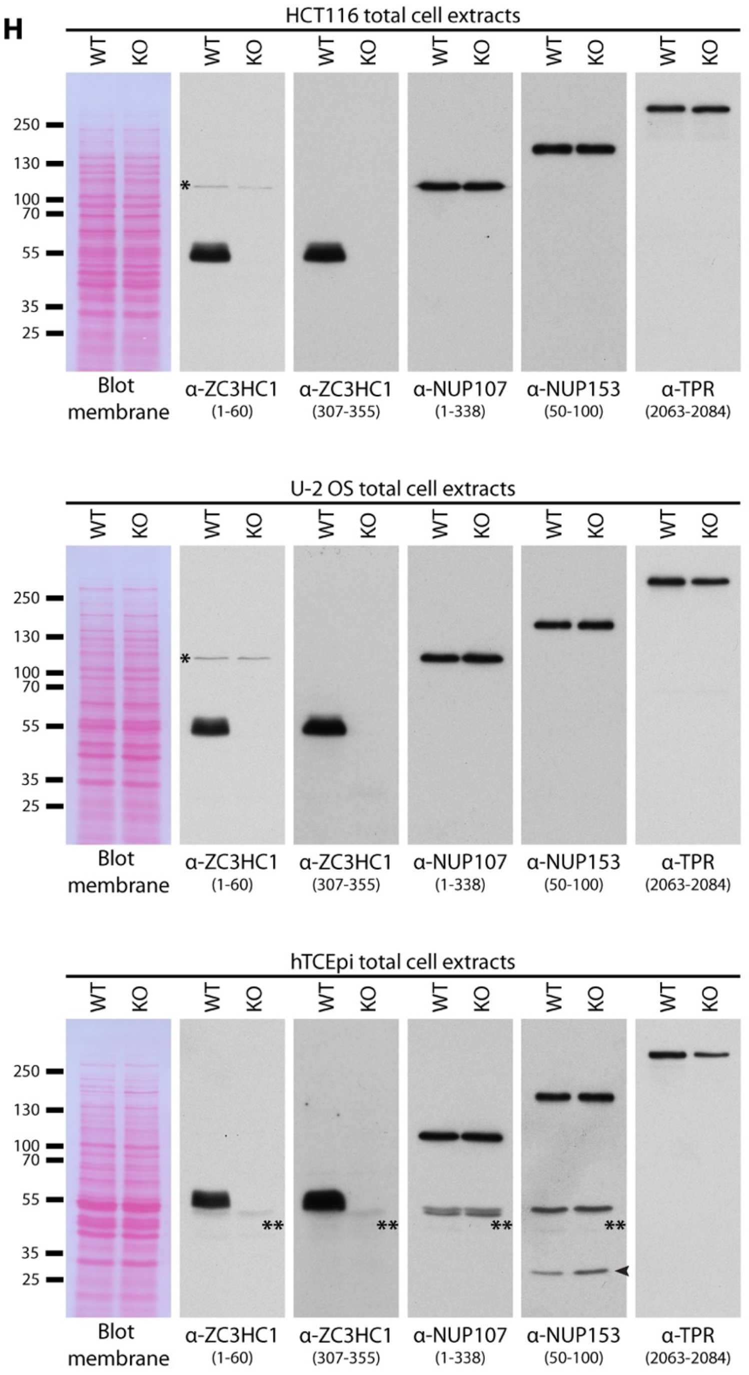
HCT116 total cell extracts
WT KO WT KO WT KO WT KO WT KO WT KO
250
130
100
70
55
35
25
*
Blot membrane
α-ZC3HC1 (1-60)
α-ZC3HC1 (307-355)
α-NUP107 (1-338)
α-NUP153 (50-100)
α-TPR (2063-2084)
U-2 OS total cell extracts
WT KO WT KO WT KO WT KO WT KO WT KO
250
130
100
70
55
35
25
*
Blot membrane
α-ZC3HC1 (1-60)
α-ZC3HC1 (307-355)
α-NUP107 (1-338)
α-NUP153 (50-100)
α-TPR (2063-2084)
hTCEpi total cell extracts
WT KO WT KO WT KO WT KO WT KO WT KO
250
130
100
70
55
35
25
**
**
**
**
Blot membrane
α-ZC3HC1 (1-60)
α-ZC3HC1 (307-355)
α-NUP107 (1-338)
α-NUP153 (50-100)
α-TPR (2063-2084)

I

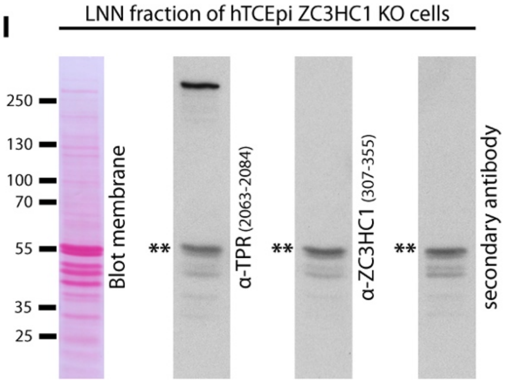
LNN fraction of hTCEpi ZC3HC1 KO cells
250
130
100
70
55
35
25
Blot membrane
**
α-TPR (2063-2084)
**
α-ZC3HC1 (307-355)
**
secondary antibody

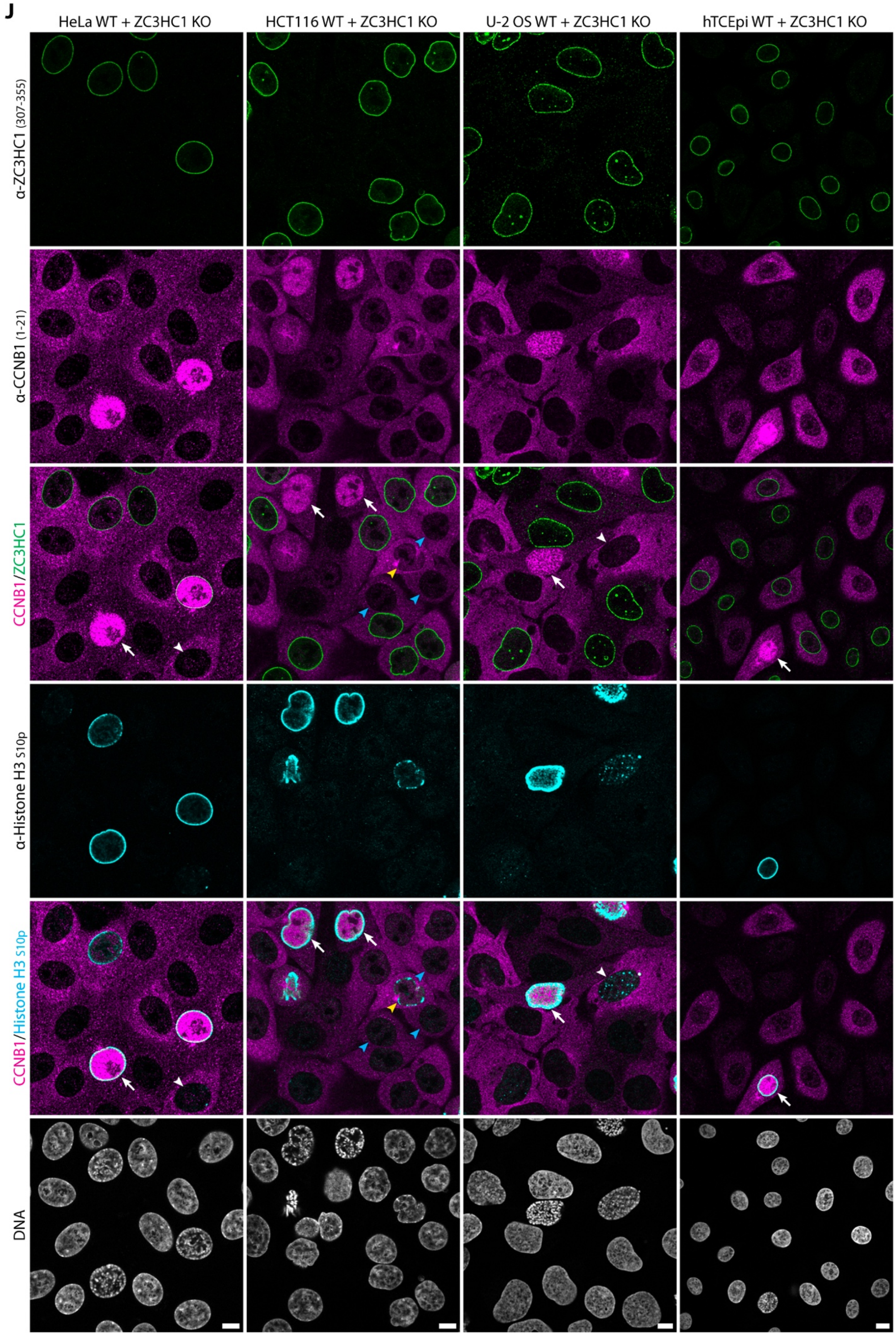
J
HeLa WT + ZC3HC1 KO
HCT116 WT + ZC3HC1 KO
U-2 OS WT + ZC3HC1 KO
hTCEpi WT + ZC3HC1 KO
α-ZC3HC1 (307-355)
α-CCNB1 (1-21)
CCNB1/ZC3HC1
α-Histone H3 S10p
CCNB1/Histone H3 S10p
DNA

S15 (6/6)

Supplemental Figure S15. Characterisation of human cell lines of mesodermal, endodermal, and ectodermal origin following CRISPR/Cas9n-mediated *ZC3HC1* gene knockout.

For *ZC3HC1* gene disruption in HeLa cells, we used the subline HeLa P2 generally referred to in the text as HeLa (see Information SI 11). Furthermore, ATCC cell lines U-2 OS and HCT116 on the one hand, and Evercyte cell line hTCEpi on the other, which in contrast to HeLa P2 had not been subcloned for upgraded properties, represented cultures of high and low passage numbers, respectively, at the time point they were used for CRISPR/Cas9n-editing. All cell lines were recurrently controlled for maintaining characteristic, cell line-specific features. Furthermore, chromosome copy numbers, and the integrity of chromosomes of particular interest to us, were controlled by karyotyping, also conducted in the course of commissional re-evaluation of the cell lines elsewhere (University of Tübingen, Faculty of Medicine, Division of Cytogenetics; Dr U.A. Mau-Holzmann). In the case of chromosome 7, harbouring *ZC3HC1* at 7q32.2, both the near-diploid HCT116 and the diploid hTCEpi cells were confirmed possessing two intact copies of this chromosome, while the hypotriploid HeLa P2 cells possess three of them; with none of these cell lines revealing any partial 7q-arm translocations. By contrast, yet in a sense similar to former and most recent observations (e.g., https://www.lgcstandards-atcc.org/en/Products/All/HTB-96.aspx#characteristics; [154–156]), indicating notable karyotypic diversity among subclones from U-2 OS-derived cell lines and complex chromosomal rearrangements characteristic for this cell line, many cells of those cell passages of the hyper-triploid line U-2 OS used in our study were found to possess seemingly only one largely intact copy of chromosome 7. The other copy, or copies of chromosome 7, appeared rearranged or fragmented in such a manner that the integrity and fate of their 7q-arm and possible fragments thereof remained ambiguous to us. This finding was taken into account when analysing the ZC3HC1 disruptions in U-2 OS with special care. Since sequencing of the CRISPR/Cas9n-edited exon 1 region of *ZC3HC1* unambiguously resulted in two distinct sequences, we assume that two *ZC3HC1* alleles exist in the U-2 OS subline used in the current study, both of which had been targeted for disruption.

Furthermore, the homozygous ZC3HC1 KO cell lines presented in the current study, i.e., those in which all *ZC3HC1* alleles had been verifiably disrupted, were karyotyped as well, largely confirming findings made with each line's corresponding WT progenitor version. Before that, these lines had been characterised by genomic PCR and sequencing (S15A), IFM (Figure 6 and S15B,C,F,G), and IB (Figure 6 and S15H). Further note that each one of the isolated KO cell lines had then been passaged at least 50 times without showing signs of increased apoptotic rate or any other conspicuous change in fitness.

(A) Characterisation of the *ZC3HC1* exon 1 locus in CRISPR/Cas9n-edited ZC3HC1 KO cell lines.

(A1) Schematic depiction of the human *ZC3HC1* gene and the nucleotide and deduced aa sequences corresponding to exon 1, which in the current study was the target of CRISPR/Cas9n-conferred disruption, using the Cas9 double-nickase procedure for this purpose [157].

(A2) DNA target sites within *ZC3HC1* exon 1 and corresponding single-guide RNAs (sgRNAs). Two pairs of sgRNAs were used independently from each other for introducing indels into the first *ZC3HC1* exon. Sequences of these sgRNAs are shown in light blue lettering, next to those of their dsDNA target site regions, here shown as boxed sequences. The two protospacer adjacent motifs (PAM) of each pair of sgRNAs are highlighted in magenta-coloured lettering. For further details, see the Material and Methods section. Also note that both pairs were often used in parallel since we had noted that the efficiencies of introducing indels either via the one or the other pair of sgRNAs differed between some of the cell lines.

(A3 and **A4)** Genomic sequence comparison between the intact WT and the disrupted alleles of the finally obtained ZC3HC1 KO cell lines (S15A3), and the therefrom deduced amino acid sequences (S15A4), reflecting the potential remnants of truncated ZC3HC1 polypeptides in the different KO cell lines. Sequences provided in magenta-coloured lettering in S15A3 represent nucleotides that have either been inserted, like in the case of HCT116, deleted (-), or otherwise exchanged compared to the WT sequence. The magenta-coloured sequences in S15A4 represent aa that are not part of the genuine ZC3HC1 sequence but the result of rearrangements, insertions and frameshifts caused by CRISPR/Cas9n-editing. Note that such mutant sequences were deduced from sequenced plasmids with inserted PCR products; the latter obtained by PCR amplification of the genomic regions flanking the site of *ZC3HC1* disruption. For each of the *ZC3HC1* cell lines, the corresponding PCR product-containing plasmids had been isolated from varying numbers of single colonies of transformed *E. coli* in order to reach a greater than 99% probability of having each allele represented among them at least once. As an aside, the numbers of such colonies sequenced for the almost diploid cell line HCT116 and the diploid non-tumour cell line hTCEpi were several-fold higher than those that were sufficient for reaching statistically significant information for HeLa and U-2 OS. The reason was that in the HCT116 and hTCEpi KO cell lines, we could only detect one distinct type of indel per cell line, even though two slightly different indels would have been conceivable also for each of these two cell lines' two *ZC3HC1* alleles. However, extensive sequence analyses in combination with the other IB and IFM data obtained (Figure 6, and see below) eventually allowed for concluding that in each of the homozygous HCT116 and hTCEpi KO cell lines, both of their ZC3HC1 alleles had been effectively disrupted by only one type of indel.

(B) IFM of HeLa WT and ZC3HC1 KO cells, complementary to Figure 6A.

(B1) HeLa WT cells had been grown together with cells from a stable HeLa strain in which all *ZC3HC1* alleles had been disrupted by CRISPR/Cas9n technology. The mixed populations of WT and KO cells had been synchronised to the G1-phase and then labelled with two antibodies targeting different parts of ZC3HC1 and with an antibody against NUP153 as reference. KO cells are here shown demarked by arrows. While laser power in the upper panel was chosen, as usual, to result in hardly any pixels reaching the maximum of the dynamic range, the same cells presented in the lower panel are shown overexposed far beyond saturation. We purposefully applied more laser to demonstrate that labelling of ZC3HC1 with two different ZC3HC1 antibodies was neither detectable at the NEs nor anywhere else in the KO cells. Areas marked by rectangles were analysed by ImageJ, allowing for plotting and merging the line profile for ZC3HC1 with that for NUP153 in the WT cell, thereby further underscoring the absence of ZC3HC1 in the neighbouring ZC3HC1 KO cell. Also note the encircled and 6x enlarged line profile sections, showing the signal of ZC3HC1 in the WT cell being slightly shifted towards the nuclear interior even relative to NUP153. Bar, 10 µm.

(B2) IFM of cell-cycle-synchronised mixed populations of HeLa WT and ZC3HC1 KO cells harvested in G2 and then labelled with antibodies for ZC3HC1 and TPR, and with an antibody for hsNUP153 as reference. The micrograph showing the immunolabelling for TPR is also shown colour-graded, next to a colour LUT. In addition, the nuclei of the WT cells (arrowheads) and some neighbouring ZC3HC1 KO cells (arrows) are marked. Note that signal intensities for TPR at the NEs of the KO cells in G2 again appeared reduced by about half, similar to the degree of reduction noted in G1, as shown in Figure 6. Bar, 10 µm.

(C) IFM of ZC3HC1 KO cells, exhibiting variations in the apparent amounts of nucleoplasmic TPR. While all ZC3HC1 KO sublines produced in the current study were found to exhibit a similar degree of reduced staining for TPR at their NEs in comparison to the cells of the corresponding WT cell lines (see also Figure S16), we had noted that the amounts of nucleoplasmic TPR could vary notably, either between the individual cells of the same KO subline, as exemplified in S15C1, or between the cells of different KO sublines that stemmed from the same starting population of CRISPR/Cas9n-edited cells, with examples shown in S15C2.

(C1) While cells of the HeLa ZC3HC1 KO subline presented in Figure 6A and S15B2 always exhibited a similar degree of reduced staining for TPR at their NEs in comparison to the HeLa WT cell line, we had also observed in this subline, as well as in some of the other clonal HeLa ZC3HC1 KO sublines (data not shown), that the amounts of nucleoplasmic TPR sometimes appeared to notably differ between the individual KO cells of the same population, for reasons yet unknown. Here we show the micrograph of a mixed population of such WT and ZC3HC1 KO cells, as an especially conspicuous example of neighbouring KO cells harbouring either a large, a reduced or no obvious pool of nucleoplasmic TPR, here labelled with arrows, arrowheads and asterisks, respectively. On the other hand, colour-graded illustration of such immunolabelling for TPR once again confirmed that staining intensities for TPR at the NEs of these KO cells did not differ notably, despite such conspicuous variability in the nucleoplasmic amounts. Bar, 10 µm.

(C2) Variability in the apparent amounts of nucleoplasmic TPR was also pronounced in the isolated HCT116 KO cell lines. However, in contrast to the example for the HeLa ZC3HC1 KO cell line shown in S15C1, where such variability was noted between individual KO cells of the same subline, variability in the case of the HCT116 KO cells mainly applied to apparent differences noted between the monoclonal HCT116 KO sublines in their entirety, with essentially every cell in the one subline exhibiting a prominent nucleoplasmic pool of TPR whereas such a pool was absent in essentially every cell of the other HCT116 KO sublines of this type.

For the two extreme examples shown here, WT and ZC3HC1 KO cells had once again been grown together on the same coverslip as an asynchronous population. The lines had been cloned from the same population of HCT116 cells treated with ZC3HC1-targeting sgRNAs, and the isolated cell clones had been confirmed to represent homozygous KO lines and to exhibit the essentially same degree of TPR reduction at their NEs. Nevertheless, only some of them (exemplified by clone #1, which is also presented in S15F) harboured a major and persisting pool of nucleoplasmic TPR that was still well detectable (some examples marked by arrows) within essentially all of the population's cells, even after passaging the cells for at least half a year. By striking contrast, in another homozygous HCT116 KO line (clone #2), such a nucleoplasmic pool was hardly or essentially no longer at all detectable in any of the cells (some marked by arrowheads), and this already after the early cell passages during clone isolation.

We had noticed a diminution in the amounts of a nucleoplasmic pool of TPR in most of the other cell types' ZC3HC1 KO clones too, and this again, often as early as during clonal growth (our unpublished data). While this might reflect adaptation processes that lead to the diminishment of those amounts of soluble TPR that were no longer needed or even disadvantageous for a ZC3HC1 KO cell, we could only guess at the various possibilities of how this might have come about. With scenarios imaginable ranging from diminished TPR expression to enhanced turnover of soluble TPR polypeptides, the final unriddling of what might actually apply to which KO cell line would have gone beyond the scope of the current study. Currently, we can only state that in some of the KO sublines of tumour origin inspected in this regard so far, the diminishment in soluble TPR amounts did not come along with any apparent downregulation in TPR gene transcription and in the copy numbers of TPR transcripts reaching the cytoplasm (our unpublished data). Bar, 10 µm.

(D) IFM of the subcellular distribution of CCNB1 in pairs of HeLa WT and ZC3HC1 KO cells from mixed populations of cells that had been grown together on one coverslip and harvested primarily in G2 and mitosis. Complementing Figure 6D, the pairs of WT and ZC3HC1 KO cells shown here, from the same coverslip as those shown in the overview image in Figure 6D, were selected because they were neighbouring cells at essentially the same cell cycle time point. Note that CCNB1 appeared similarly well excluded from the nuclei of both the WT and ZC3HC1 KO cells until the very end of the G2-phase, when H3-S10 phosphorylation had already commenced (marked by arrowheads). Note further that the time point and the degree of CCNB1 import into the nuclei of a neighbouring pair of WT and KO cells early in prophase were essentially indistinguishable. Bar, 10 µm.

(E1 and **E2)** Quantification of nuclear CCNB1 immunostaining in ZC3HC1 KO cells relative to that in WT cells (S15E1), and the same cells' corresponding nuclear to cytoplasmic ratios (S15E2), the latter thus also taking each cell's CCNB1 expression level into account. For such quantifications, via signal intensity measurements of defined areas in the cytoplasm and nucleoplasm, images had been randomly acquired from a mixed G2/M population of WT and ZC3HC1 KO cells that had been grown next to each other on the same coverslip and then stained for CCNB1 and ZC3HC1. The corresponding specimen was the same for which a representative collection of micrographs is presented in Figure 6D.

The actual signal intensity measurements, using only those images showing the CCNB1-labelled cells, were completed before correlating any of these images with the accompanying co-labellings for ZC3HC1. Not knowing, therefore, which cells were ZC3HC1-negative or -positive, the mean values were only calculated once each measurement value had been assigned to either a ZC3HC1 KO or WT cell after finally having unveiled the corresponding immunolabelling data for ZC3HC1. Furthermore, to increase the precision and significance of the comparisons, we also took the cells' total CCNB1 expression levels into account. Imaged cells exhibiting similar levels of cytoplasmic CCNB1 staining were assigned to one of three different groups of CCNB1 staining intensities, believed to reflect the gradual increase of cytoplasmic CCNB1 amounts during the G2-phase. This thus resulted in three separate datasets, representing a low, an average and a high degree of cytoplasmic CCNB1 staining. The resulting mean values of nuclear CCNB1 staining for each of the corresponding subgroups are presented, with the WT datasets' values set to 100% and the percentage values for the corresponding subgroups of KO cells presented as relative to the WT. Numbers n stand for the evaluated cells per dataset. The standard deviations provided for all of these datasets, reflecting notable differences in nuclear CCNB1 staining intensities, illustrated that the degree of CCNB1 immunolabelling notably differed even between those G2 cells that had been assigned to the same subgroup, yet with such differences being similarly pronounced in the WT and KO cells. The diagrams on the left side of S15E1 and S15E2 represent these individual sub-datasets, while the diagrams of the right represent the corresponding mean of all three. Note that neither the separate datasets nor the corresponding means for all three revealed any pronounced differences between the WT and ZC3HC1 KO cells' degree of nuclear CCNB1 staining. In fact, this quantification applying to the entire population of cells led to a KO to WT ratio of only 0.99 to 1, not pointing at any increase in CCNB1 levels within the KO cells' nuclei at all. Essentially the same also held true for the mean ratios of nuclear to cytoplasmic CCNB1 staining. Neither did they differ significantly between the separate datasets nor between the WT and KO cells' entire populations, with these calculations having resulted in a KO to WT ratio of 1.01 to 1.

(F to H) Characterisation of other KO cell lines, resulting from CRISPR/Cas9n-mediated ZC3HC1 gene disruption in HCT116, U-2 OS, and hTCEpi, revealing toleration of ZC3HC1 deficiency in karyotypically and morphologically dissimilar cells of endodermal, mesodermal and ectodermal origin.

(F) IFM of each of the cells' WT cells grown together with cells of a stable progeny line in which all ZC3HC1 alleles had been disrupted by CRISPR/Cas9n technology. The mixed populations of WT and ZC3HC1 KO cells had been cell cycle-synchronised, with those of lines HCT116 and U-2 OS then harvested in G1 and G2 phase, and those of line htCEpi in G1 phase only, then followed by immunolabelling for ZC3HC1, TPR and NUP153. The micrographs showing the immunolabelling for TPR are also shown colour-graded, next to a colour LUT. Some WT cells are marked by arrowheads, while arrows point out examples of neighbouring KO cells. Note that ZC3HC1 was not detectable in the KO cells and that the signal intensities for TPR at the NEs were notably reduced in all three KO cell lines. Conspicuous additional staining for TPR within the nuclear interior was only apparent though in the ZC3HC1-deficient cells of the particular HCT116 KO subline, whereas hardly any such nucleoplasmic TPR staining was notable for the U-2 OS and hTCEpi KO cells. As an aside, note that especially in the WT cells of line U-2 OS it was recurrently possible to detect a small number of cells in which staining intensities for ZC3HC1 and TPR were higher than in neighbouring WT cells, with an example here marked by the arrowhead in yellow. Since this was not found to be generally accompanied by higher NPC density (our unpublished data), we interpreted such finding as more ZC3HC1 and TPR polypeptides per NPC than in the other cells of the same population, suggesting that some of the U-2 OS WT cells had been capable of recruiting and appending more than the more common amounts of TPR and ZC3HC1 to their NBs. Bar, 10 µm.

(G) IFM micrographs of pairs of neighbouring WT and KO cells, with the plane of focus near their nuclear surfaces. Images were taken from the identical specimens of mixed cell populations shown in equatorial view for HeLa in S15B2, and for HCT116, U-2 OS and hTCEpi in S15F. The micrographs showing the immunolabelling for TPR and NUP153 are also shown colour-graded, next to a colour LUT. Note that the density as well as the intensity of the punctate signals from the immunolabellings of NUP153, representing NPCs in both the WT and KO cells, had remained largely unaffected in the

absence of ZC3HC1. Accordingly, NPC density having remained essentially unaffected in the ZC3HC1 KO cells was also apparent in the monochromatic images for TPR. However, the signal intensity of most of these TPR-positive dots again appeared notably reduced, which in many cases reflected a reduction by approximately half, as it was especially evident when inspecting the colour-graded images. In line with findings based on ZC3HC1-deficiency that had been achieved by RNAi in HeLa (Figure S10B), this indicated, only at first sight seemingly trivial and self-evident, that even at the level of an individual NPC, the total number of TPR polypeptides specifically attached to it was often reduced by about half when ZC3HC1 was absent. Bars, 10 µm.

(H) IB of total cell extracts from the three cell lines' WT and ZC3HC1 KO cells. Immunolabelling for TPR, NUP107 and NUP153, and with two ZC3HC1 antibodies, was on the membranes shown here and on duplicates with identical loadings. Single asterisks mark a cross-reaction of one of the ZC3HC1 antibodies, in the current exposures only seen in the immunoblots of the U-2 OS and HCT116 cell extracts. The arrowhead marks a cross-reaction by the NUP153 antibody only seen in hTCEpi. The double asterisk marks another cross-reaction, just beneath the band for ZC3HC1, which was only seen in hTCEpi cell extracts and which also arose when only using secondary antibodies (see S15I), with a more pronounced intensity of this band merely reflecting a longer exposure time, as it was the case for the immunoblots for NUP107 and NUP153. In particular, though, note that ZC3HC1 was not detectable in any of the KO cells' extracts while the total amounts of NUP107 and NUP153 were similar to those in the corresponding WT cells. Further note that the total TPR amounts were similar in the HCT116 WT and KO cells, too, the latter apparently harbouring a large nucleoplasmic pool of TPR no longer attached to the NE (A). By contrast, TPR amounts were reduced to some extent in the KO cells of line U-2 OS and, in particular, in those of hTCEpi, with both cell lines lacking conspicuous pools of nucleoplasmic TPR.

(I) IB of the LNN fraction of ZC3HC1 KO cells of line hTCEpi, revealing an unspecific cross-reaction, so far only detectable for this particular cell line. Immunolabelling was performed with a representative TPR antibody used for this purpose in a higher than standard dilution, with a commonly used ZC3HC1 antibody, and with a secondary antibody on its own. Such labelling was performed on the membrane here stained with Ponceau S and on two replicates with identical loadings. Note that even though incubation and washing steps for each immunoblot were conducted strictly separate from those for each of the other two, all of them eventually exhibited the same pattern of bands corresponding to polypeptides ranging between 30-55 kDa. Even though these IB images represented far longer exposure times than ordinary, the major cross-reaction here marked by a double asterisk was commonly still detectable as a faint but nonetheless unmistakable band in the immunoblots for hTCEpi that are shown in S15H and in Figure 7C and 7D, where this band is marked by a double asterisk as well.

(J) IFM of the subcellular distribution of CCNB1 in WT and ZC3HC1 KO cells of lines HCT116, U-2 OS, and hTCEpi, next to such of line HeLa for comparison, complementing Figure 6D and S15D. Each cell line's WT and ZC3HC1 KO cells had been grown together on one coverslip each. Following their release from a G1/S block, cells of lines HeLa, HCT116 and U-2 OS were harvested as populations primarily containing cells in G2 and at the onset of mitosis. Cells of the slow-growing cell line hTCEpi, by contrast, were released from a G0/G1 arrest and harvested as a population that also included cells in cell cycle phases other than G2 and mitosis. The cells were then immunolabelled for ZC3HC1 and CCNB1 and with the antibody targeting the phosphorylated serine 10 (S10p) of histone H3 (H3-S10p). KO cells marked by white arrows represent cells at the G2/M transition point when staining for H3-S10p all along the NE is generally prominent and when CCNB1 has already been imported into the nucleus. The white arrowheads, by contrast, mark a HeLa KO cell and a U-2 OS KO cell shortly before this time point. Note that while H3-S10 phosphorylation had already commenced in these cells, CCNB1 appeared still well excluded from these nuclei. The yellow arrowhead marks the nucleus of an HCT116 KO cell in which nuclear import of CCNB1 appeared to have just begun, while the arrowheads in blue mark the nuclei of neighbouring KO cells in G2, in which CCNB1 was still excluded from the nucleus. Bars, 10 µm.

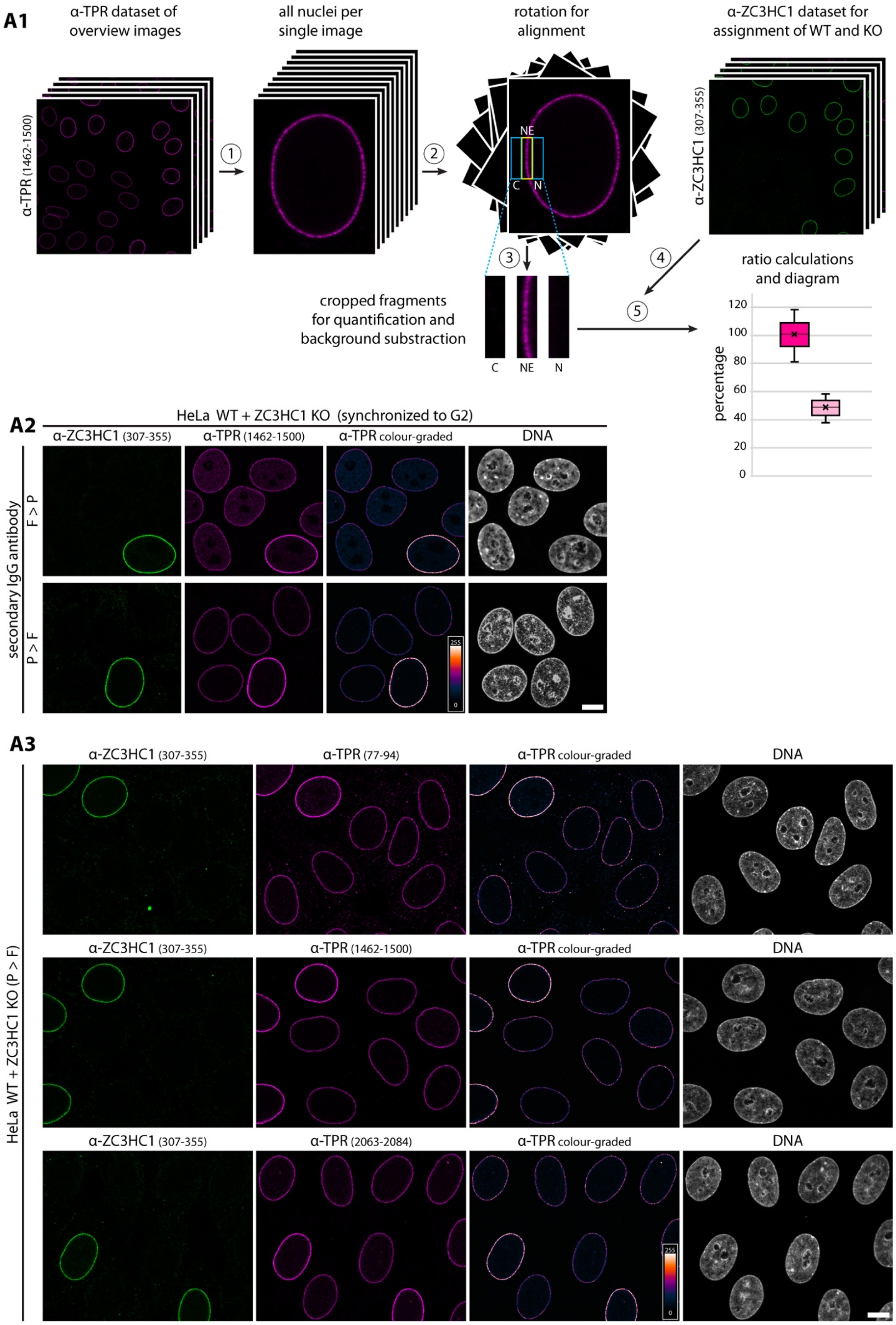
A1
α-TPR dataset of overview images
all nuclei per single image
rotation for alignment
α-ZC3HC1 dataset for assignment of WT and KO
α-TPR (1462-1500)
α-ZC3HC1 (307-355)
NE
C N
1
2
3
4
5
ratio calculations and diagram
cropped fragments for quantification and background substraction
C NE N
percentage
120
100
80
60
40
20
0
A2
HeLa WT + ZC3HC1 KO (synchronized to G2)
α-ZC3HC1 (307-355)
α-TPR (1462-1500)
α-TPR colour-graded
DNA
secondary IgG antibody
F > P
P > F
255
0
A3
α-ZC3HC1 (307-355)
α-TPR (77-94)
α-TPR colour-graded
DNA
α-ZC3HC1 (307-355)
α-TPR (1462-1500)
α-TPR colour-graded
DNA
α-ZC3HC1 (307-355)
α-TPR (2063-2084)
α-TPR colour-graded
DNA
HeLa WT + ZC3HC1 KO (P > F)
255
0

S16 (1/2)

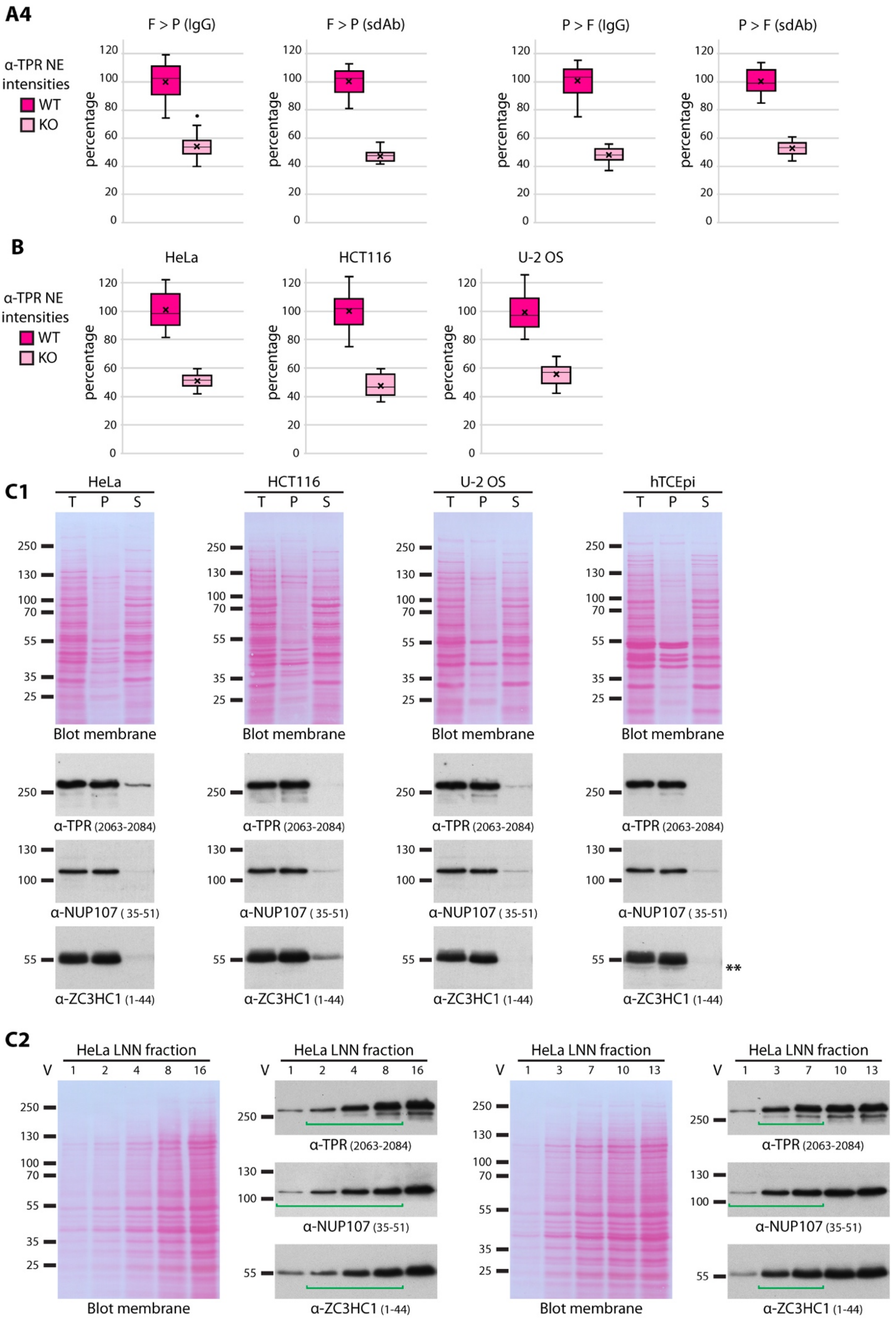
A4
α-TPR NE intensities
WT
KO
F > P (IgG)
F > P (sdAb)
P > F (IgG)
P > F (sdAb)
percentage
B
HeLa
HCT116
U-2 OS
C1
hTCEpi
T P S
Blot membrane
α-TPR (2063-2084)
α-NUP107 (35-51)
α-ZC3HC1 (1-44)
**
C2
HeLa LNN fraction
V 1 2 4 8 16
V 1 3 7 10 13

S16 (1/2)

Supplemental Figure S16. Approximations of the relative amounts of NE-associated TPR in WT and ZC3HC1 KO cells.

Pitfalls and advantages that can be encountered when approximating amounts of NB components via different immunolabelling approaches, involving the use of polyclonal and monoclonal bivalent IgGs or F(ab')$_2$ fragments, as well as monovalent Fab fragments and single-domain antibodies, sdAbs, are to be presented and discussed in exemplifying detail in another context elsewhere. Even so, we can already present in the current study a set of together diagnostically conclusive approximation data obtained via rapid, rather unchallenging approaches that have made use of bivalent IgGs.

In the one approach, we used an epitope-characterised TPR mAb as the primary antibody, in combination with either an IgG or a sdAb as the secondary antibody, for approximating the TPR amounts at the NEs of ZC3HC1 KO cells relative to WT cells by IFM, quantifying staining intensities at the NEs of neighbouring WT and KO cells that had been cell cycle-synchronised and grown together on the same coverslip. The flow chart presented as S16A1 illustrates this procedure of IFM data acquisition and signal intensity quantifications. Some exemplifying images of cells in G2, shown here in S16A2, and corresponding quantification of signal yields for immunolabelled TPR at the NEs of such HeLa WT and ZC3HC1 KO cells, presented in S16A4, complement the corresponding images and quantification data presented in the Figure 7A1 and 7A2, which represent and correspond to cell populations harvested in G1. Additional IFM micrographs presented in S16A3 show mixed populations of HeLa WT and ZC3HC1 KO cells cell cycle-synchronised in G2 that had been labelled with yet other TPR antibodies, performed in parallel to labellings with the abovementioned TPR mAb. While we used the TPR mAb for more systematic quantification of the NE-associated amounts of TPR, these two other TPR antibodies revealed virtually the same degree of signal reduction for NE-associated TPR in the ZC3HC1 KO cells. The diagrams in S16B represent quantification data for TPR at the NEs of WT and ZC3HC1 KO cells of lines HeLa, HCT116 and U-2 OS harvested in G2, complementing corresponding data presented in Figure 7B for cells harvested in G1.

Among the potential pitfalls when it comes to approximating target protein amounts with the use of antibodies, it is especially the IFM-based quantification of a target, as part of a cellular structure *in situ*, that can come along with several potential sources of error. Apart from those potentially caused by the bivalency of an intact IgG in general, we could not ignore the possibility that if a target protein occurs in different arrangements, it might well be better accessible for antibodies in the one arrangement than in the other. Moreover, we even had to consider the possibility that additional differences in accessibility might arise when the original arrangement of the target structure had been altered, as might have been the case with the residual NBs in the ZC3HC1 KO cells. In fact, at this point, we could not yet exclude the possibility that the residual amounts of NPC-appended TPR in the four KO cell lines might be better accessible for antibodies than in the WT cells. Such enhanced accessibility would be analogous to former observations when the absence of TPR caused by RNAi had been found to result in more intense immunofluorescent labelling of an NR-positioned protein like NUP107. In fact, contributing to the NB anchor platform at the NPC, NUP107 had turned out to be far better accessible for certain NUP107 antibodies when TPR was no longer around [71, and our unpublished data].

Because of such caveats and limitations regarding the diagnostic value of IFM-based quantifications when only standing for themselves, we thus regarded such data alone as not unambiguous and consequently as not yet sufficient for drawing conclusions as to the quantitative ratio of NE-associated TPR in WT to KO cells. Therefore, we also compared the relative amounts of TPR associated with the LNN fractions of WT and KO cells by quantitative IB.

We already knew that in two of the four human WT cell lines primarily used in the current study, namely in HCT116 and hTCEpi, essentially all TPR occurred appended to the NPC and was thus present within LNN fractions prepared under conditions preserving NB integrity while essentially no evident additional soluble pool of TPR was detectable in these cells in interphase. Only in the HeLa subline, and sometimes also in the U-2 OS cells, in which it appeared to also depend on cell culture density (our unpublished data), a minor pool of soluble TPR was detectable in addition to the vast amount of TPR being NPC-appended and present in the LNN fractions of these cell lines too. With examples of such fractionations presented in S16C1, and having proven by other means that (i) TPR's presence within the LNN-fractions mirrored its NPC-appendage and that (ii) the residual amounts of TPR appended to the NPCs in ZC3HC1 KO cells were also not detached by those conditions that allow for maintaining NB integrity in the WT cells (our unpublished data to be presented in another context elsewhere), we found us in a favourable starting position for comparing the NPC-attached amounts of TPR by quantitative IB.

However, in order for such quantitative IB approach to yield diagnostically conclusive data, it was still necessary to determine the ranges of detection within which the signal intensities for the WT and KO cells' TPR amounts, and those of NUP107 as the essential reference protein, were linearly proportional to the amount of each of these antigens, with sufficient overlap in the two protein's linear ranges of detection additionally required. Furthermore, while one could have, in principle, individually compared each cell line's pair of WT and KO cell-derived LNN fractions only between themselves, we aimed at being able also to compare the corresponding amounts directly between the different cell lines. The latter, however, differed notably with regard to their total NPC numbers per total cellular protein content, as can be deduced, e.g., from Figure 7C. This, in turn, meant that we not only had to see to it that the loaded NUP107 amounts were adjusted accurately but that such similar NUP107 amounts, among conspicuously different total protein amounts, were actually loadable for SDS-PAGE. Moreover, the same also held for the different WT and KO cells' TPR amounts, whose loaded amounts, of course, needed to conform with their linear IB detection range too. Furthermore, to also allow, as an

aside, for directly comparing the different WT cell lines' ZC3HC1 amounts, relative to those of the other NPC and NB components, as also presented in Figure 7C, one had to determine its linear range of detection too, which in the end meant that there needed to be sufficient overlap in all three proteins' linear ranges of detection. Eventually, with some of the TPR, NUP107 and ZC3HC1 antibodies available to us, it had then turned out possible to define conditions resulting in a narrow, but sufficient overlap among the linear detection ranges for all three proteins. Importantly, this could be achieved within the range of LNN amounts from all cell lines that could be loaded for SDS-PAGE, with this here exemplified by the corresponding IB data for HeLa cells, shown in S16C2.

However, such cell fractionation experiments did not only allow for comparing the NB-appended amounts of TPR in the WT and ZC3HC1 KO cells. Beyond that, findings like those presented in S16C1, representative for several other human cell lines too (our unpublished data), actually revealed a particular reciprocity that we regard as highly informative, namely that cell lines possessing a minor soluble pool of TPR have essentially no soluble pool of ZC3HC1, and, *vice versa*, cell lines with a soluble pool of ZC3HC1 have no soluble TPR. Not yet having detected any somatic cell type in which notable pools of soluble TPR and ZC3HC1 would unambiguously be co-existing, we interpret this finding as another indication for a strict dependence of distinct pools of TPR on ZC3HC1, and *vice versa*, of ZC3HC1 on TPR, in order for both ending up appended to the NB. In other words, if for one of the two proteins, there would still be a surplus of some freely available soluble polypeptides after having interacted with all available copies of its binding partner, such surplus would not be able to attach to the NB on its own but remain in solution instead. While this appears to be the case for TPR in the current study's HeLa cell line, the corresponding case of a surplus of ZC3HC1 remaining soluble is a characteristic for the cell line HCT116, as similarly evident in S16C1.

(**A**) Quantification of the relative amounts of NB-associated TPR, in the WT and ZC3HC1 KO cells of line HeLa, following different procedures of immunolabelling.

(A1) Flow chart illustrating the procedure leading to the quantification of signal yields for immunolabelled TPR at the NEs of WT and ZC3HC1 KO cells. First, randomly acquired overview images of mixed populations of WT and KO cells, which had been grown together on the same coverslip and then immunolabelled for TPR and ZC3HC1, were used to cut out the images of essentially every TPR-labelled complete nucleus that had been imaged clearly in focus at its equator ①. Not yet correlating this with the immunolabelling for ZC3HC1, this eventually resulted in large compilations of images representing individual nuclei of essentially unknown WT or KO cell origin. Then, following each image's rotation for rectilinear alignment ②, three same-sized rectangular areas were cut from each image ③, comprising an NE segment as well as flanking cytoplasmic and nuclear areas. Dimensions of the rectangles were initially chosen in such a manner that the area occupied by the labelled NE represented approximately one third of the surface area in the NE rectangle, allowing for subtracting the non-NE background intensity by the simple equation: signal yield at the NE equals total signal yield for rectangle NE minus 1/3 of total signal yields for both rectangle C and N. Only then, after having completed such quantification for each individual nucleus, was each dataset assigned to a WT or a KO cell, by correlating the immunolabelling for TPR with the corresponding labelling for ZC3HC1 ④, which then in turn allowed for ratio calculations ⑤. Since a few of the datasets presented in S16A4, S16B, and in Figure 7A2 and 7B had initially included some rare but extreme outliers, possibly also for non-biological reasons, each dataset's lowest and highest values (5% each) were categorically excluded, with such cut-off allowing for better comparability between all datasets.

(A2) IFM of mixed populations of HeLa WT and ZC3HC1 KO cells that had been grown as co-cultured, cell-cycle-synchronised populations and then harvested in G2. Cells were permeabilised with TX-100 after fixation (F > P) or before fixation (P > F), the latter resulting in the removal of the nuclear pool of soluble TPR occurring primarily within the ZC3HC1 KO cell lines in varying amounts. Labelling was with guinea pig antibodies for ZC3HC1 and a mAb for TPR. The latter was then detected with fluorophore-conjugated IgGs, as in the examples presented here, and with mouse IgG1-specific sdAbs (not shown). Micrographs of this kind, obtained from the four different types of specimens, were then used for random acquisition of NE segment-containing image segments and determining the signal intensities therein, as described in S16A1. The corresponding quantification of signal yields is shown in S16A4. In addition, the micrographs for TPR are once again shown colour-graded, next to a colour LUT. Bar, 10 µm.

(A3) IFM of mixed populations of HeLa WT and ZC3HC1 KO cells that had been grown as co-cultured, cell-cycle-synchronised populations and then harvested in G2. Cells were permeabilised before fixation (P > F). Labelling was with guinea pig antibodies for ZC3HC1 and with two different polyclonal rabbit TPR antibodies and one mouse monoclonal TPR antibody in parallel, with these, in turn, detected with fluorophore-conjugated IgGs. Note that the TPR mAb (1462-1500), which has been used for all systematic IFM-based quantification of NE-associated TPR amounts in different human cell types, and which also had been used for S16A1, was here merely used as reference antibody, allowing for a comparison with the results obtained with the other TPR antibodies, which similarly revealed that the NE-associated amounts of TPR were reduced by about half in the KO cells. Bar, 10 µm.

(A4) Quantification of signal yields for immunolabelled TPR at the NEs of HeLa WT (magenta-coloured) and ZC3HC1 KO (light magenta-coloured) cells in G2, following specimen preparation and immunolabelling as exemplified in S16A2. Randomly chosen NE segments used for quantifications via ImageJ (see also S16A1) were from essentially all labelled cells

seen in equatorial view within randomly chosen images of mixed populations of WT and KO cells. Box plots display the relative signal intensity values measured for the KO and WT cells' NEs, with the arithmetic mean value (marked by x) for the WT set to 100%. Note that the mean TPR signal yield for the KO cells' ZC3HC1-free NEs was only about half the WT cells' corresponding value.

(B) Quantification of signal yields for immunolabelled TPR at the NEs of WT and ZC3HC1 KO cells of lines HeLa, HCT116, and U-2 OS that had been grown and then evaluated in parallel. Like in S16A, WT and ZC3HC1 KO cells had been co-cultured as mixed populations, which here though had then been harvested in G2, followed by permeabilisation with TX-100 after fixation and labelling with antibodies for ZC3HC1 and TPR, with the latter then detected with fluorophore-conjugated, mouse IgG-specific sdAb (for exemplary micrographs see also S15B2 and S15F). Note that the dataset accumulated for HeLa represented an experiment distinct from the corresponding one presented in S16A4, which had been conducted separately at a different time point. Further note that the values determined for the KO cells of line U-2 OS likely moderately overrated the actual amounts of NE-associated TPR in the ZC3HC1-devoid cells of this particular cell line, due to certain peculiarities of U-2 OS cells that have already led to data misinterpretation in some other U-2 OS-based studies. While we will address this issue in further detail elsewhere, we measured signal intensities at the NEs of U-2 OS cells in the current study in the same manner as for the other cell lines to allow for direct data comparison. Apart from that, note that the mean TPR signal yield for the KO cells' ZC3HC1-free NEs was once again found to reach only about half the WT cells' corresponding value, with this applying essentially to all three cell lines.

(C) IB of cell extracts obtained from WT populations of the four cell lines HeLa, HCT116, U2OS and hTCEpi.

(C1) IB of total cell extracts and subcellular fractions from cells harvested shortly after having reached confluency in order to compare the NE-associated and soluble amounts of TPR within each cell type. Lanes had been loaded with the non-fractioned WT cells' total cell proteins (T), the soluble and detergent-extractable proteins (S) obtained upon treatment with TX-100 in NB-s buffer, and the corresponding proteins of the non-soluble, pelletable LNN material (P). Amounts of the different cell lines' loaded fractions represented the same volume fraction of the respective samples' total amount, thus essentially representing the same number of extracted cells per cell type while not representing the same numbers for the different cell lines. Immunolabellings were on the membranes here shown stained with Ponceau S and on duplicates with identical loadings. Note that only minor or hardly any soluble amounts of TPR or ZC3HC1 were detectable within the different cell lines. The minor amounts of detergent-extractable NUP107 likely comprised a mixed population of polypeptides originating from detergent-extractable cytoplasmic AL, from the minor soluble pool of NUP107 detergent-extractable from nuclei in interphase, and from probably only a few mitotic cells still among the asynchronous cell populations used for these cell fractionations. Note further that cell lines harbouring a minor soluble pool of TPR, like HeLa and sometimes also U-2 OS, had hardly any soluble ZC3HC1, while cell lines possessing a small pool of soluble ZC3HC1, like recurrently noted for HCT116, had hardly any soluble TPR.

(C2) Immunoblots for assessing the linear ranges of detection achievable for TPR, NUP107 and ZC3HC1. Lanes had been loaded with serial dilutions of the non-soluble, pelletable LNN material of HeLa WT cells. The smallest amount loaded per lane is marked as 1, with multiples thereof in the other lanes marked accordingly. Labellings with antibodies for TPR, ZC3HC1 and NUP107, pre-selected from among others as potentially suitable for quantitative IB, were on the membranes here shown stained with Ponceau S and on duplicates with identical loadings. Brackets mark the approximate range of amounts for which the signal intensity for a target protein was regarded as fairly proportional to the amount of antigen. Note an overlap in such ranges of detection for TPR, ZC3HC1 and NUP107.

Eventually, these findings allowed not only for comparing TPR, NUP107 and ZC3HC1 amount relationships directly between the WT cells of the different cell lines, as shown in Figure 7C, but also for more accurately assessing by comparative IB the differences in TPR amounts within the LNN material obtained from the different cell lines' WT and ZC3HC1 KO cells, as presented in Figure 7D. However, it also needs to be mentioned that while quantitative IB for determining relative amounts was technically feasible and allowed for unveiling copy number relationships that appear to be very similar in different cell lines, it had not been possible to determine absolute TPR numbers via such IB approaches. The reason was that we had not been able to isolate sufficiently large and pure amounts of tag-free full-length TPR suitable as quantity standard when using, for example, insect cells and the baculovirus expression system [see also 158]. Therefore, a subsequent study will need to address the absolute copy numbers of TPR and ZC3HC1 and determine these by other methods.

3. Supplemental Discussion: Former Perceptions of ZC3HC1 Function in Mammals

3.1. Former conception of ZC3HC1 as a protein with an anti-apoptotic function.

In the eponymous report for NIPA [120], and some other studies [113,159], ZC3HC1 had been portrayed as a protein with an anti-apoptotic function, while neither our data nor other lines of evidence support this notion.

In two of these studies, constitutive overexpression of recombinant ZC3HC1 homologs had been reported to inhibit or suppress apoptosis in different human cell lines [120,159]. In the third study, the RNAi-mediated KD of ZC3HC1 in HeLa cells had been described as instantaneously triggering apoptosis in most of the siRNA-treated cells, which was proposed to reflect enhanced sensitivity of tumour cells towards ZC3HC1-deficiency [113].

In a further study, ZC3HC1 was among a large number of proteins whose knockdown in the human breast adenocarcinoma cell line MB231 by RNAi, when followed by inducing apoptosis with the tumour necrosis factor-related apoptosis-inducing ligand TRAIL, had been found to result in higher rates of cell death, along with enhanced levels of the effector caspase-3 and caspase-7 [160]. On the other hand, though, ZC3HC1 had not been detected in several other, methodologically quite distinct screens for anti-apoptotic human proteins [161–163], and neither had Pml39p been found among the anti-apoptotic proteins in *S. cerevisiae* [164], with Pml39p being the ZC3HC1 homolog in budding yeast, as we will show in a separate study.

In the course of our work, we had not observed increased numbers of apoptotic cells in any of the human cell lines, including HeLa, in which ZC3HC1-deficiency had been achieved by RNAi or gene knockout. Moreover, even when having triggered apoptosis in ZC3HC1-deficient HeLa cells via the extrinsic or the intrinsic pathway, the apoptotic phenotypes were not more pronounced than in HeLa WT cells treated the same way. When eliciting apoptosis in the other ZC3HC1 KO cell lines, though, we sometimes did note slightly more enhanced signs of apoptosis in some of them (our unpublished data). Since the causal relationships between ZC3HC1 and other molecules that might explain such enhanced sensitivity to different elicitors of apoptosis in the absence of ZC3HC1 still remain obscure to us, we momentarily refrain from categorically ruling out the possibility that the presence of ZC3HC1 in some cell types might have some minor anti-apoptotic impact along the one or other pathway that leads to effector caspase activation.

Besides this reservation, though, we are sceptical about the notion that the anti-apoptotic effects observed upon marked overexpression of ZC3HC1 [120,159] are reflecting a genuine property of physiological relevance. In fact, in light of certain sequence similarities between ZC3HC1 and the BIR domains of the IAP proteins [159,165; our unpublished data], we have been considering a non-physiological scenario in which only very high levels of ectopically expressed ZC3HC1 would allow for some low-affinity inhibitory interactions with proteins like for example the effector caspase-3 and caspase-7, which normally would only be blocked by the IAP proteins themselves [e.g., 166,167].

While this issue will need to be addressed by future work, it is already certain that depleting the different cell lines of their natural content of ZC3HC1 in our study, either by RNAi-mediated KD or by CRISPR/Cas9n-mediated gene disruption, does not by itself trigger apoptosis, as neither did ZC3HC1 RNAi in the abovementioned MB231 cell line as long as it was not challenged by TRAIL [160]. We, therefore, deem it possible that the very conspicuous signs of apoptosis and cell cycle inhibitory effects formerly noted upon NIPA/ZC3HC1 RNAi [33,89,113] were rather reflecting off-target effects specific for the one ZC3HC1 siRNA that had been used as the only one in all these former studies.

Finally, however, when discussing ZC3HC1 in the context of apoptosis, it would be remiss not also to mention that signs of apoptosis had actually also been noted in the differentiation-arrested germ cells of ZC3HC1-deficient mice [113]. In this case, though, one also needs to consider that an arrest in germ cell differentiation, causing subsequent

apoptosis of these cells, is a relatively common phenotype, observed upon knockout of very diverse genes with no direct role in apoptosis [e.g., 168,169].

3.2. Former perception of ZC3HC1 as an F-box protein.

In principle, possession of a true F-box does allow proteins to bind to SKP1 [170] as one of the core components of the SCF complex, with SKP1 thus acting as the adaptor between the F-box protein and the other members of this complex [e.g., 171]. However, in the case of ZC3HC1, there is actually no F-box to be recognised as part of its sequence, with this at variance with the former assignment of an F-box to aa 170-210 of hsZC3HC1 [33]. Only some residues within the first half of the sequence located between 170-210 are arranged in a manner similar to those of the authentic F-boxes of other proteins, while the second half of this region clearly deviates from not only the F-box consensus [e.g., 172] but also from the other F-box-like sequences deposited in motif databases (e.g., http://pfam.xfam.org/family/PF00646, http://pfam.xfam.org/family/PF12937, http://pfam. xfam.org /family/PF13013, http://pfam.xfam.org/family/PF15966). Most importantly, several conserved residues and residue spacings, present in the second half of true F-box and F-box-like domains and directly involved in the interaction with SKP1, as they form part of the binding interface [e.g., 96,172], are absent in ZC3HC1. In fact, when using published F-box/SKP1 crystal structures, with an emphasis on human F-box and F-box-like proteins (e.g., SKP1-SKP2, PBD entry 1FQV, [96]; SKP1-beta-TrCP1 (BTRC), PDB 1P22, [172]; SKP1-FBW7, PDB 2OVP, [173]), for molecular re-modelling, by replacing the F-boxes, both in parts and *in toto*, for the alleged F-box of ZC3HC1, or parts thereof, we could eventually alleviate steric clashes between the F-box binding interface of SKP1 and the alleged F-box of ZC3HC1 by energy minimisation [e.g., 174], but this then resulted in the loss of most of the contact points usually existing between SKP1 and true F-boxes (our unpublished data). At the end of such *in silico* interaction studies, we, therefore, considered it in principle possible that the short segment of ZC3HC1 whose aa sequence is distantly reminiscent of the sequence signature of an F-box, might allow for some low-affinity interactions with SKP1, but not for such that one would deem stable and persistent. However, even if such weak binding might be able to occur, in particular when corresponding sites within the recombinant proteins might even have become more accessible by non-natural conformational changes, we currently do not regard such interactions as being of physiological relevance. This conclusion is also based on our finding that the endogenous ZC3HC1 polypeptides in the cells studied in the current investigation had turned out not to be capable of efficiently competing with the natural F-box proteins for the F-box binding site of SKP1.

Apart from our experimental data, this conclusion also takes generally accessible information into account. For example, most of the sequence segment that comprises residues 170-210 shares little or no sequence similarity among different phyla. Most notably, while P171 of human ZC3HC1 had been reported as essential for SKP1-binding [33] and supposed to correspond to an essentially invariant residue of the F-box-like consensus sequences (http://pfam.xfam.org/family/PF12937, http://prosite.expasy.org/ PDOC50181), we found this residue at this site already missing in other vertebrates, namely in birds, reptiles and amphibians, and thus also in *Xenopus laevis*. Accordingly, F-box-like domains were neither detectable in the ZC3HC1 homologs of these organisms nor any other. If, on the other hand, the reported interaction between ZC3HC1 and SKP1 were as fundamentally crucial for cell cycle regulation as formerly described, we would have expected to see the reported site of interaction to be evolutionarily conserved at least among other vertebrates, assuming that mammals would not be the only vertebrates possessing such a cell cycle control mechanism. In this context, we also regard it a noteworthy finding that when we had immunoprecipitated a tagged version of *Xenopus laevis* ZC3HC1 from cell extracts that also contained a tagged version of the human canonical SKP1, which shares 100% sequence identity with SKP1 from *Xenopus laevis*, the degree by which SKP1 had been co-sedimented together with the amphibian ZC3HC1

homolog had essentially been the same as upon co-sedimentation together with the tagged human ZC3HC1. This revealed that P171 of human ZC3HC1 did not even tend to improve some kind of interaction with SKP1.

3.3. Former perception of ZC3HC1 as a stable component of an E3 ubiquitin ligase of the SCF-type.

The feature that has been attributed to ZC3HC1 as its most significant one, as an alleged nuclear F-box protein, was to be a stable component of a multi-protein E3 ubiquitin ligase of the SCF-type [e.g., 33,85–87], with such findings also recurrently reported reproducible [e.g., 89,90]. Along this line, ZC3HC1 had been described as only detaching from such nuclear SCF core complex, consisting of subunits SKP1, CUL1 (Cullin 1) and RBX1, very late in G2, just prior to mitosis, which supposedly would then result in rapid degradation of ZC3HC1 [33,85,87].

However, summarising the outcome of series of experiments performed in the course of our study, it had not been possible for us to detect any naturally occurring direct and steady interaction, during normal cell growth and in different cell types in interphase, between the cell's endogenous ZC3HC1 polypeptides and the endogenous SCF subunits SKP1 and CUL1. Moreover, having initially also considered it possible that ZC3HC1 interaction with TPR might prevent binding to SKP1 and the other components of the SCF ubiquitin ligase complex in a mutually exclusive fashion, this turned out not to be the case either.

Furthermore, having also used for such IP experiments ectopically expressed versions of both N- and C-terminally tagged ZC3HC1, all of which in principle were well capable of attracting TPR, even these vast amounts of recombinant ZC3HC1 had not engaged in any noteworthy beyond-background interaction with endogenous CUL1. Similarly, endogenous SKP1 polypeptides had either not been found co-sedimented upon IP of such recombinant ZC3HC1 or, in the case of the FLAG-tagged ZC3HC1 polypeptides, only in minute amounts just above background levels. Further calculations then led us to the conclusion that the excess amounts of ectopically expressed ZC3HC1 within the transfected cells were apparently not capable of efficiently competing with the true F-box-containing proteins for the F-box-binding interface of the endogenous SKP1 polypeptides, even though the total number of the recombinant ZC3HC1 polypeptides was at least one and up to two orders of magnitude higher than the total number of all F-box proteins within such a transfected cell. In other words, within the same cell and within the same time frame during which an abundance of ZC3HC1 polypeptides had been newly synthesised, those endogenous SKP1 polypeptides that likely had been newly synthesised as well had managed to engage in lasting interactions with genuine, also newly synthesised endogenous F-box proteins, like SKP2, but not with the excess of ZC3HC1.

Likewise, having also ectopically expressed vast amounts of differently tagged versions of the canonical, i.e., most common isoform of SKP1, well capable of attracting the endogenous polypeptides of F-box protein SKP2, such recombinant SKP1 had not been found attracting any substantial beyond-background amounts of endogenous ZC3HC1. Furthermore, in order to investigate whether ZC3HC1 might instead be a preferred target of the shorter, less common isoform of SKP1, we had ectopically expressed and immunoprecipitated tagged versions of this shorter type of SKP1 too. This isoform differs from the canonical version only by a few amino acids at its C-terminus, which, however, likely affect the integrity of the protein's F-box binding interface. Indeed, we found this short version of SKP1 hardly capable of attracting endogenous SKP2. More relevant for us, we found this shorter version of SKP1 not interacting with ZC3HC1 either.

Finally, we had also performed IP experiments with cell extracts containing huge amounts of recombinant ZC3HC1, SKP2 and SKP1 in different combinations, after having ectopically co-expressed pairs of each two of these proteins. When immunoprecipitating the recombinant SKP1 from the cell extracts, the recombinant ZC3HC1 polypeptides were

once again not found co-sedimented or only in trace amounts. At the same time, the immunoprecipitated SKP1 had been well capable of attracting the recombinant version of SKP2, thereby confirming that the F-box binding interface of SKP1 was functional in the context of the protein's tagged version.

However, in one particular experimental set-up, we eventually and then recurrently noted an interaction between the tagged SKP1 and ZC3HC1 polypeptides. This was the case when such tagged versions of SKP1 and ZC3HC1 had been ectopically co-expressed and when it was ZC3HC1 that had then been immunoprecipitated. Irrespective of which type of tag we had used for this purpose, which also included the FLAG-tag commonly used in the former studies on NIPA/ZC3HC1 [33,86–90], such IP of recombinant ZC3HC1 was always found accompanied by co-sedimentation of a small amount of the recombinant SKP1.

Since such co-sedimentation generally appeared more pronounced for shorter, seemingly truncated versions of the chimeric polypeptides consisting of tag and SKP1, we also considered a scenario in which it might simply be the tag appended to SKP1 that might have prevented a more quantitative interaction with ZC3HC1. To address also this possibility, we had then ectopically expressed, together with ZC3HC1, either the canonical or the shorter isoform of SKP1 without any tags. However, instead of this then leading to an increase in co-sedimentation, the tag-free recombinant SKP1 polypeptides were no longer co-sedimented with ZC3HC1 at all, just as it had been the case for the tag-free endogenous SKP1 when ZC3HC1 had been immunoprecipitated in earlier experiments.

Even though we did not investigate the peculiar one-way interaction, noted only between the immunoprecipitated tagged versions of ZC3HC1 and the co-sedimented tagged SKP1, in further detail, the data accumulated by then already allowed for reaching several conclusions, with the most important one being that ZC3HC1 is not a protein that is a common natural component of an SCF complex.

Furthermore, while we cannot entirely exclude a scenario in which endogenous ZC3HC1 and SKP1 might engage in some interaction for whatever reason in some distinct situations, for example when signal-induced post-translational modifications of the one or other protein might result in some conformational changes and affinity enhancement, we currently see no evidence in support of any naturally occurring interaction of physiological relevance between ZC3HC1 and SKP1 in cells grown under normal conditions. Instead, we regard the only type of interaction that we could observe between the two proteins, namely between the overexpressed tagged versions of SKP1 and ZC3HC1 upon IP of the latter, to represent a non-physiological kind of low-affinity interaction, with this possibly involving some ZC3HC1 sequence segments that might have become slightly better accessible upon conformational changes caused by sequence tags fused to both proteins. In addition, we also consider it possible that constitutive overexpression of huge amounts of such recombinant proteins can come along with some of the polypeptides not being correctly folded, resulting in the exposure of potential sites for non-physiological interactions. We furthermore do not exclude that such sites might have included short segments located within a ZC3HC1 region comprising residues 170-210. However, while this segment has formerly been stated to represent an F-box that forces ZC3HC1 to interact with the SCF complex all throughout interphase [33,85–87,113], we regard this statement to now have been discounted by the here presented data.

We can also imagine that the tagging of ZC3HC1 and SKP1, in order to use them as chimeric bait and prey proteins in yeast two-hybrid (Y2H) screens, might have played a role in the study in which only SKP1 cDNAs had been reported to have been isolated in the course of having Y2H-screened a human testis cDNA library with hsZC3HC1 that had been N-terminally tagged with LexA [33]. However, with the Y2H system generally known to be a methodology producing large numbers of false-positive interactions [e.g., 175,176], there might well be also other reasons for why such SKP1 cDNAs have been isolated back then. On the other hand, the Y2H system is equally well known for leading

to high numbers of false-negatives too. We, therefore, cannot argue against some alleged ZC3HC1-SKP1 interaction merely because our own screening of a yeast GAL4-AD fusion library made of human cDNAs, representative of the transcriptome of various adult human tissues, using full-length hsZC3HC1 fused to GAL4-BD as the bait, had resulted in the isolation of TPR cDNAs but not of SKP1 cDNAs, as we will show in a separate study. However, we regard it as a complementing argument against a ZC3HC1-SKP1 interaction that recent large-scale Y2H studies that have investigated the human interactome by using different Y2H assays in a complementary manner with several iterations [e.g., 177; http://www.interactome-atlas.org] have identified 60 Y2H interaction partners for SKP1, the vast majority of which were actual F-Box proteins, yet without ZC3HC1 being among them.

Of further note, high-throughput screening of human protein interactions had identified TPR, but neither SKP1 nor other proteins of the SCF complex, as a binding partner of ZC3HC1 [178]. Similarly, systematic searches for interaction partners of SKP1 in *Arabidopsis, Dictyostelium* and *S. cerevisiae* [e.g., 179–181] had resulted in the identification of numerous F-box proteins, but these did not include the prototypic homologs of ZC3HC1 that are present in these species, as we will show in a separate study.

Finally though, while all currently available evidence forces us to conclude that the cells' endogenous ZC3HC1 polypeptides are generally not naturally occurring active constituents of SCF complexes or of any other E3 ubiquitin ligases, we deem it necessary to mention also that we have noted in the course of our investigations that certain components of the NPC-NB complex can be targets of stress-induced signalling. In this context, we do not wish to exclude the possibility that the NB itself, and individual components thereof, can, in certain situations, be targets for certain E3 ligases and even for subsequently recruited proteasomes.

3.4. Former perception of ZC3HC1 as a protein binding CCNB1 for subsequent degradation.

The formerly reported primary function of ZC3HC1 as an alleged SCF component was the binding, for subsequent destruction, of CCNB1 polypeptides that manage to untimely leak into the nucleus during interphase [33,85–88,113]. Accordingly, elimination of ZC3HC1 by RNAi in proliferating cells had been reported to go along with a notable nuclear enrichment of CCNB1 even in G1- and S-phase, and in premature mitotic entry as a consequence thereof. In addition, such ZC3HC1 deficiency had also been reported to be accompanied by a pronounced arrest in prometaphase in most cells [33,85]. However, we neither observed a physical interaction between CCNB1 and ZC3HC1, nor could we reproduce the CCNB1 phenotypes formerly described as a result of ZC3HC1 RNAi. Furthermore, our finding that ZC3HC1 deficiency does not cause any notable cell cycle phase-specific arrest is in line with ZC3HC1 not having been among the human proteins identified in genome-wide screens as necessary for cell division [182].

In this context, it is also worth mentioning studies that have already shown long ago that CCNB1 polypeptides leaking into the nucleus too early in interphase are rapidly cleared from the nuclear interior by being re-exported into the cytoplasm, simply by being bound by the nuclear export factor CRM1, which then ensures proper cytoplasmic localisation of CCNB1 before the onset of mitosis [124–126]. More recently, CCNB1 has even turned out to be one of those proteins most efficiently cleared from the nuclear interior of human cells via CRM1-RanGTP-mediated export [183]. This re-export mechanism counteracts the constantly occurring unintentional leakage into the nucleus of those proteins that are meant to remain in the cytoplasm, which is a consequence of NPCs not being perfect permeability barriers [e.g., 183,184] and which represents a general and permanent challenge for the cell. However, instead of dealing with this influx by degrading all the proteins that do not belong there, including such that would likely exhibit deleterious effects as a consequence of their mislocalisation, the standard mode of removing them from the nuclear interior appears to be their re-export back into the

cytoplasm [183]. This is not only a rather economical way of solving the problem, since one does not need to destroy functionally intact proteins that have just been synthesised, but also appears to make sense from a logistical point of view. Rather than having to maintain an additional machinery for recognising and destroying all the different proteins that are temporarily mislocalised into the nucleus, the cell can simply exploit its common export pathways for keeping the nucleus free of such proteins [183].

Even though degradation machinery in the form of proteasomes are known to be tethered to the inner nuclear membrane of the NE, and to a certain extent also to be associated with TPR homologs and the NB in several unicellular organisms with closed mitosis, including yeasts [e.g., 185,186] and the single-cell green alga *Chlamydomonas reinhardtii* [187], such proteasomes might rather be executing other functions at these sites [e.g., 185–188], instead of trying to handle the work overload they might likely encounter if they were supposed to eliminate all the cytoplasmic proteins continually leaking into the nucleus. Moreover, while it has been pondered on whether proteasomes might perhaps also exist at the NEs of terminally differentiated mammalian cells, they at least appear largely absent from the NE and NPCs of proliferating HeLa cells [e.g., 187,189].

In summary, we see neither indications nor simply any need for a nuclear SCF complex with a specific role in eliminating CCNB1. Instead, we consider it possible that the increase in cellular CCNB1 amounts formerly observed upon RNAi-mediated KD of ZC3HC1 [e.g., 33,89] reflected an off-target effect provoked by the one and the same siRNA used in those studies (also see Figure S12). By contrast, having found that CCNB1 is in fact not prematurely accumulating within the nuclei of ZC3HC1-deficient cells now even allows for concluding that efficient re-export of CCNB1 into the cytoplasm neither requires ZC3HC1 nor those TPR polypeptides that are only appended to the NE when ZC3HC1 is available.

3.5. Former perception of ZC3HC1 as a binding partner of the tyrosine kinase NPM-ALK.

Several former studies on the mammalian homolog of ZC3HC1 had addressed this protein by the name "nuclear interacting partner of anaplastic lymphoma kinase" (NIPA), as it had been initially reported [120] to be a binding partner of the oncogenic tyrosine kinase NPM-ALK. First isolated in a Y2H screen, ZC3HC1 had been described as forming a complex with the kinase-active NPM-ALK also *in vitro*. However, at least for the wild-type version of this tyrosine kinase, it is unlikely that it will encounter ZC3HC1 as a natural binding partner in interphase because ALK is a plasma membrane-anchored protein [e.g., 190]. Most of all, though, we deem it well possible that the isolation of ZC3HC1 during the original Y2H screen [120] actually did not reflect an interaction between ZC3HC1 and ALK. In fact, inspecting the origin of the binding domain (BD) construct that encoded the bait later used for the Y2H screen, we realised that this chimeric BD-ALK protein actually included a segment comprising aa 1-142 of human TPR [191]. Since this segment comprises residues of TPR that allow for robust interaction with ZC3HC1 in Y2H experiments (our unpublished data to be presented elsewhere), we consider it likely that this TPR segment, rather than ALK itself, had attracted ZC3HC1 in the former study. Of additional note, TPR is, in fact, among the fusion partners of ALK that exist as chimeric proteins in certain types of lung cancer due to *ALK* rearrangements occurring therein [192,193].

4. Supplemental Material and Methods

4.1. Production of antibodies

All antibodies for which data are presented in the current study are listed in Table S2. Among these were rabbit (rb) and guinea pig (gp) antibodies raised against recombinant protein segments expressed in and purified from bacteria, or against synthetic peptides (Peptide Specialty Laboratories, Heidelberg, Germany) coupled via a C-terminal or N-terminal cysteine to keyhole limpet hemocyanin (KLH). Affinity purification of antibodies raised against synthetic peptides followed standard procedures [53]. For the purification of antibody populations raised against larger protein segments, all of which had been equipped with a C-terminal cysteine, the sera were sometimes first loaded onto the corresponding recombinant polypeptides that had been either immobilised to CnBr-Sepharose (GE Healthcare, Uppsala, Sweden) or Maleimide-Sepharose (kindly provided by Dirk Görlich). This was followed by (i) eluting the bound antibodies from there, (ii) subsequently loading these antibody pools onto columns with bead-coupled peptides of 17 or 18 aa residues in length, which covered the recombinant polypeptide's entire sequence with a 9 aa long sequence overlap between consecutive peptides, and (iii) finally eluting the subpopulations of antibodies bound to the different peptides. More commonly, though, the sera were loaded directly onto such series of columns with Sepharose-immobilised peptides. As described in Figure S1A and S1B, this approach allowed for the isolation of nearly epitope-specific antibody subpopulations. Note further that only rabbit antibodies were used in the current study for immunogold-labellings of *Xenopus* oocyte specimens. This was so because we had recurrently noticed that labellings with at least some different batches of immunogold-coupled anti-guinea-pig antibodies caused some minor yet not to be ignored degree of cross-reaction at the NE's nuclear side in *Xenopus* oocytes, yet not in mammals. Such cross-reaction was not observed with secondary antibodies raised against rabbit IgGs. Note further that some antibody epitopes have been fine-mapped further in the course of this study, like in the case of the mouse monoclonal TPR antibody 203-37 and the ZC3HC1 B-10 mAb. Further note that for those commercial secondary antibodies here used for IFM, each of which was advertised as specific for their primary antibodies' host species, we additionally tested and re-confirmed such species-specificity.

Further information related to Figure S1 and the production and characterisation of antibodies, including the cloning of bacterial expression vectors encoding for protein segments of xlZC3HC1, xlNUP153 and xlTPR, the expression and purification of these recombinant proteins to be used for immunisations, the baculovirus expression system for xlZC3HC1, its expression in *Drosophila* Schneider 2 cells and its purification from these, in order to be used for immunisations as well, and the details regarding the ELISA procedure, are to be presented in a different context elsewhere but can also be provided upon request.

4.2. RNA isolation from Xenopus oocytes, and synthesis and cloning of cDNAs

Total RNA was isolated from *Xenopus* oocytes by extraction with TRIzol Reagent (Invitrogen, Carlsbad, CA, USA) and RNA then transcribed into cDNA using the SuperScript III First-Strand Synthesis System for RT-PCR (Invitrogen), with both procedures according to the manufacturer's instructions. The cDNA was then used for PCR amplification of target sequences using gene-specific primers, followed by standard methods for subcloning into expression vectors.

4.3. Feeding specifications for Xenopus laevis females

Most *Xenopus* data presented in the current study are based on *Xenopus laevis* females that had been purchased from NASCO (Atlanta, GA, USA) at the age of about 1.5 years, and that approximately one to 1.5 years later had undergone either their first ovariectomy for the isolation of small amounts of the ovary or had received the first hormone injections

for egg-laying. Groups of animals were fed on several different types of diets, including a lower-caloric but complete whole food diet with a 28% protein content for adult frogs with an average body weight of about 120 g, whose oocytes, in the current study, were primarily meant to be used for studies of the NE. By contrast, heavyweight frogs of more than 170 g weight, sometimes exceeding 200 g, that were to be used for the preparation of assembly-competent egg extracts or the isolation of oocytes with an especially high content of annulate lamellae (AL), were fed on a higher-caloric and more protein-rich (39%) diet. These specifications are based on findings made in the course of a several years-long feeding study in which we have investigated how different diets can affect how different types of proteins are stored in *Xenopus* oocytes for later use during embryogenesis.

4.4. IFM of cryostat and ultrathin sections

Cryostat sectioning of *Xenopus laevis* oocytes and animal tissues of different origin that had been frozen in isopentane and mounted with Tissue-Tek (Sakura Finetek, Torrance, CA, USA) was performed with a Leica CM1900 (Leica Microsystems, Wetzlar, Germany) at either -20°C or -25°C, with chosen section thicknesses of 5 or 7 µm, depending on the type of tissue. Sections were collected on SuperFrost Ultra Plus microscope slides (Gerhard Menzel B.V. & Co.KG, Braunschweig, Germany), air-dried for one hour, and then immediately used or stored at -20°C. For IFM, the defrosted sections were fixed with 2.0% or 2.4% formaldehyde (FA) in PBS, with or without 7 mM $MgCl_2$, for 30 min, washed with PBS, and then quenched with 50 mM NH_4Cl in PBS for 5 min, except for sections of *X. laevis* tissues of ectodermal germ layer origin. After defrosting, these were placed in -20°C acetone for 10 min and then washed with PBS. Except for the ectodermal tissue and the oocyte sections, that were then directly treated with 1% BSA in PBS and subsequently incubated with antibodies diluted in the same blocking solution, all other FA-fixed tissue sections were additionally detergent-treated with 0.4% or 0.5% Triton X-100 (TX-100) in PBS, prior to blocking with BSA and incubation with antibodies, with incubation times, washing steps, and mounting as already described for IFM of cultured cells. For IFM of ultrathin sections of high pressure frozen, freeze-substituted and K4M-embedded *Xenopus* stage II oocytes, such sections were mounted onto coverslips of 5 mm in diameter (Gerhard Menzel B.V. & Co.KG). The specimens were then incubated, for blocking, in PBS containing 25% (v/v) of DAKO antibody diluent solution S3022 (DAKO, Glostrup, Denmark) for 30 min. Dilutions of antibodies and subsequent incubations with them and all washing steps were performed in the same blocking buffer. Incubation with primary rabbit antibodies was for 90 min, followed by several washes, a 60 min incubation with fluorophore-coupled secondary antibodies, and further washes. Specimens, then mounted with anti-fade mounting medium, at the beginning of the project with Vectashield anti-fade mounting medium (Vector Laboratories, Burlingame, CA, USA) and later generally with SlowFade Gold Antifade Reagent (Invitrogen), were evaluated either with a Leica TCS SP5 or later with a Leica TCS SP8 confocal scanning microscope, both equipped with a 20x and a 63x oil immersion objective (Leica Microsystems).

4.5. Isolation of AL-enriched subcellular fractions, cytosol, and NEs from Xenopus oocytes

Prior to the AL isolation procedure, oocytes were manually defolliculated and then enucleated. Further biochemical fractionation of the enucleated oocytes was, in essence, as described [60], but with some modifications. First, the AL-enriched material, sedimented by a 10 min centrifugation at only 2500 g, in order to subsequently allow for quantitative resuspension of the AL, was resuspended in 5:1-H buffer and then recentrifuged once more at 2500 g to remove minor amounts of yolk material that had been resuspended together with the AL. After removing the supernatant, which was then referred to as the AL wash in Figure 1B2, the AL-containing layer of the pellet was

resuspended with 0.1x 5:1-H, followed by supplementing this suspension with the same volume of 2x protein sample buffer. Furthermore, the supernatant obtained after the first centrifugation at 2500 g was recentrifuged at 13,000 g, with the resulting supernatant referred to as the cytosolic fraction. Manual isolation of oocyte NEs and nuclear contents in 5:1-H buffer at 18°C was done with self-made pipettes, equipped with a system permitting tuned adjustment of suction pressure and glass capillaries whose diameters were smaller than that of an oocyte nucleus. This allowed for the detachment of the NE from the nuclear contents by shearing forces, and separate collection of both, as described earlier [194]. NEs meant to undergo NB removal were first washed with $MgCl_2$-free 5:1-H, with further NB destabilisation then achievable by applying one of several different physicochemical conditions. Some of these could be used in combination, with the efficiency of NB removal varying depending on the frog's diet plan, weight and age. In general, for NBs present in the later stages of oogenesis, i.e., in the virtual absence of NE-associated chromatin [e.g., 12], the destabilisation, followed by at least structural disintegration and subsequent partial, and sometimes also complete removal of their components, could already be achieved by transferring the nuclei into ice-cold (0°C to less than 1°C) $MgCl_2$-free 5:1-H, then followed by incubating and washing them therein (in the following called "cold-treatment") for various lengths of time. With this approach, essentially complete loss of some frogs' NBs was sometimes observed after less than 5 min, whilst in other cases, the removal of NB components remained incomplete even after 30 min at 0°-1°C. Alternatively, NBs could be rapidly detached by a 10 min incubation at 18°C in KOH-buffered 10 mM or 20 mM HEPES buffer, pH 7,4 or 7,2 (in the following called "no-salt-treatment), with the SEM micrographs shown in Figure 1A3 and the IBs in Figure 1B reflecting the result of such a no-salt-treatment at 18°C. NB release experiments needed to be carefully controlled, commonly by SDS-PAGE followed by silver-staining and IB, as they sometimes, again varying between frogs, caused small amounts of some of the other peripherally attached NPC components, or their binding partners, to be detached too. For the no-salt-treatments, this could be minimised by adding minor additional amounts of monovalent cations to the buffer, up to 5 mM, in addition to the K+ cations used for the HEPES buffer's pH adjustment, yet at the expense of the most efficient NB detachment. Furthermore, both the cold- and no-salt-treatments could be combined with additional treatments involving nucleases and detergents in different combinations.

4.6. Freeze-substitution

For freeze-substitution (FS) in a Leica EM AFS2 (Leica Microsystems), specimens were first placed into 100% acetone at -90°C for 24 hours, followed by 98% acetone with 2% MeOH and 0.4% UA (w/v; Ted Pella Inc., Redding, CA, USA) at -90°C for 72 hours. Known to be insoluble in pure acetone at -90°C, the UA in the MeOH-supplemented acetone mostly precipitated too, with the traces of soluble UA then far below the nominal concentration of 0.4% [see also 195]. Nonetheless, though, such treatment allowed us to observe more contrast at subcellular structures than when having incubated the specimens at these low temperatures only in pure acetone, in which all added 0.4% UA precipitated instantaneously, or in acetone supplemented with different amounts of water, with and without UA. We thus regarded the contrast observed upon FS with 98% acetone supplemented with 2% MeOH and 0.4% UA to be due to at least some UA deposited at subcellular structures, rather than to reflect, as a consequence of the 2% of methanol, some enhanced extraction of phospholipids or more pronounced aggregation of macromolecules due to some more rapidly occurring changes in their hydration shells. After an 8 h-long linear increase in temperature to -70°C, the solution was then exchanged for 100% acetone again, maintaining this temperature for six hours and then linearly increasing it stepwise first to -60°C and then to -35°C over a period of six hours, accompanied by further 100% acetone exchanges. Specimen infiltration with resin started with a 1:1 mixture of acetone and the hydrophilic Lowicryl resin K4M (Polysciences,

Warrington, PA, USA) for 24 hours, followed with a 1:2 ratio of acetone to K4M for yet again 24 hours, subsequently with pure K4M for 24 hours and, after increasing the temperature to -25°C, 24 hours with K4M supplemented with the initiator. After returning to -35°C, curing was under indirect UV light for 48 hours, followed by a linear temperature increase to +20°C without UV light, and final hardening in daylight and at RT for at least 24 hours. As an aside, we wish to note that we had systematically investigated, starting from corresponding freeze-substitution protocols for single-layer cultured cells [196], how the addition of small amounts of water, added to the acetone at different temperatures in the course of the freeze-substitution process, might affect an oocyte specimen. We found the latter's appearance not improved when applying such acetone-water fluids but instead sometimes notably worsened, e.g., by the formation of more pronounced ice crystals (our unpublished data).

4.7. Production of mitotic and nuclear assembly-competent Xenopus egg extracts

Preparation of mitotic egg extracts and egg extracts capable of *in vitro* assembly of nuclei was, in principle, as described [197], with some modifications. The laid eggs were dejellied for 5 min by incubation at 18°C in pH 7.8-readjusted 0.25x Marc's Modified Ringer's solution (MMR, see main text) containing 2% L-cysteine (w/v), and then rinsed with MMR buffer again, before adding 1V of MMR buffer supplemented with 160 µg/ml cycloheximide, 14 µg/ml cytochalasin B, 20 µM Z-VAD-FMK, and a protease inhibitor cocktail consisting of Aprotinin, Leupeptin, Elastatinal, Chymostatin and Pepstatin A, to about 7 volumes of compacted eggs . Extracts from these eggs, resembling mitotic extracts, were obtained by compacting the eggs by low-speed centrifugation at 100 g for 30 sec, and then at 600 g for 90 sec, followed by removal of excess buffer, and subsequently crushing these eggs by 20 min centrifugation at 4°C and 20,000 g. The resulting supernatant was supplemented with 3% glycerol, frozen in liquid nitrogen and then stored at -150°C. For obtaining nuclear-assembly-competent egg extracts, the MMR buffer-rinsed eggs were first incubated for 7 min in MMR containing 160 ng/ml of the calcium ionophore A23187, followed by ionophore removal and further incubation of eggs in MMR for 20 min, allowing for the release of the eggs from second meiotic metaphase. Subsequent preparation of extracts from these cells was as for the mitotic egg extracts.

4.8. Covalent binding of antibodies to Protein A-coupled magnetic beads

Purified rabbit or guinea-pig antibodies (5 to 10 mg) were bound to Protein A-coupled magnetic Dynabeads (Invitrogen) in 5:1-H5/T buffer (83 mM KCl, 17 mM NaCl, 5 mM $MgCl_2$, 20 mM HEPES pH 7.25, 0.02% Tween-20), by gentle rotation at RT for 75 min. After the removal of unbound antibodies and washes in 5:1-H5/T buffer, the beads were washed once in conjugation-buffer (150 mM NaCl, 20 mM sodium phosphate, pH 7.5) and then resuspended in coupling-buffer containing 500 µM of bis-sulfosuccinimidyl suberate (sulfo-DSS; Pierce, Rockford, IL, USA), followed by rotation at RT for 30 min, subsequent quenching by adding TRIS pH 7.5 to a final concentration of 25 mM, and further rotation at RT for 15 min. Beads were then washed once with coupling buffer and once with 0.5x 5:1-H5 buffer lacking Tween-20. Incompletely cross-linked antibodies were eluted with 0.1 M glycine at pH 2.5, followed by washing the beads with 5:1-H5/T buffer. The beads were either stored in 5:1-H5/T buffer at 4°C or directly transferred into buffers used for IP experiments with *Xenopus* egg extracts (41.5 mM KCl, 8.5 mM NaCl, 5 mM $MgCl_2$, 2.5 mM EGTA, 10% sucrose, 20 mM HEPES pH 7.25), oocyte extracts (83 mM KCl, 17 mM NaCl, 5 mM $MgCl_2$, 10% sucrose, 20 mM HEPES pH 7.4, 0.02% Tween-20) or XL-177 cell extracts (41.5 mM KCl, 8.5 mM NaCl, 5 mM $MgCl_2$, 2.5 mM EGTA, 10% sucrose, 20 mM HEPES pH 7.25, 0.036% TX-100), with all buffers supplemented with cOmplete Mini EDTA-free protease inhibitor cocktail (Roche, Basel, Switzerland).

4.9. Immunoblotting

Following SDS-PAGE, protein transfer onto nitrocellulose membranes, with pore diameters of 0.2 µm (Bio-Rad Laboratories, Hercules, CA, USA and Carl Roth, Karlsruhe, Germany), was in a wet-blotting chamber in a transfer solution of pH 8.4 containing 20% MeOH, 190.5 mM glycine and 23.5 mM Tris base, to which, in some cases, SDS was added to a final concentration of 0.03%, 1-2 hours after the start of the transfer. In most cases, the transfer was carried out at a constant current of 450 mA for one hour, followed by a reduced current (350 mA) overnight. For the transfer of small proteins that were to be immunodetected, the transfer was without the 1 h-long step at 450 mM and without SDS added. After the transfer, the membranes were first documented by staining with either Ponceau S (SigmaAldrich, St. Louis, MO, USA) or MemCode Reversible Protein Stain Kit (Pierce), with the original images of those membranes stained with MemCode in blue later colour-converted to red, in order to avoid mistaking with Coomassie Brilliant Blue-staining. The membranes were then again destained and commonly blocked in TBST (Tris-buffered saline with 0.1% Tween-20) containing 5% low-fat milk powder, to which 1% BSA was sometimes added to improve the signal to noise ratio obtainable with some of the antibodies. This was followed by incubations with primary and commonly HRP-conjugated secondary antibodies and detection of the immunolabelled proteins by the enhanced chemiluminescence (ECL) method, using ECL Plus or ECL Prime solutions (Amersham/GE Healthcare, Chicago, IL, USA), followed by exposure to fluorographic X-ray films (X-OMAT, Kodak, Rochester, NY, USA, or Super RX-N UV, Fujifilm, Tokyo, Japan). In some few cases in which membranes were to be re-used, after having already been incubated with antibodies, such membranes were first extensively washed with TBST and incubated therein overnight, which was followed by removing the previously bound first-round antibodies by shaking the membrane in a 100 mM glycine solution of pH 2.5 for one hour. After further washes, first with water and then with TBST for several hours, the membranes were re-incubated with primary antibodies whose species origin was different from those used for the first incubation, followed by incubations with secondary antibodies that were species-specific. This allowed for avoiding unwanted cross-reactions with any residual amounts of sometimes not altogether completely detached first-round antibodies.

4.10. Culturing of cell lines

Cell lines used in this study (see Table S4) were grown in a humidified atmosphere with 5% CO_2, at a temperature of 27°C for XL-177 and 37°C for the human cell lines. HeLa and HEK293T cells were cultured in high-glucose DMEM (D6429, Sigma-Aldrich) with 10% FBS (P40-37500; PAN-Biotech, Aidenbach, Germany) and additionally added 2 mM L-glutamine (GIBCO, Grand Island NY, USA) to a final concentration of 6 mM in the case of HEK293T cells. XL-177 cells were kept in DMEM diluted to 65% by adding 25% water and 10% FBS. HCT116 and U-2 OS cells required McCoy's 5A medium (M9309, Sigma-Aldrich) with 10% FBS. EpiLife medium containing 60 µM calcium (MEPI500CA; GIBCO) with EpiLife Defined Growth Supplement (S0125; GIBCO) was used to propagate hTCEpi cells. All media were either supplemented with 1% Antibiotic Antimycotic Solution (A5955; Sigma-Aldrich) or, in cases in which this was required, with 100 µg/ml Normocin instead (ant-nr-1; InvivoGen, San Diego CA, USA). Other molecules added to the media in the course of some experiments and that are not explicitly mentioned in the other method descriptions included ^{35}S-methionine (Hartmann Analytic GmbH, Braunschweig, Germany), applied at 90 mCi per ml medium for 3 hours, the CRM1-inhibitor leptomycin B (LMB, Sigma-Aldrich), applied at a concentration of 20 nM for 60 min, and the glucocorticoid dexamethasone (Sigma-Aldrich), applied at 1 µM for 60 min. All cell lines, including the ZC3HC1 KO progeny lines, were routinely tested for and confirmed free of contaminations by mycoplasmas and other microorganisms.

4.11. Induction of apoptosis and TUNEL assay

For inducing apoptosis, cells were incubated for 4 hours in culture medium containing either TRAIL/Apo2L (PeproTech, Rocky Hill, NJ, USA) or Raptinal (Sigma-Aldrich) at a final concentration of 250 ng/ml or 10 µM, respectively, and then harvested. Terminal deoxynucleotidyltransferase dUTP nick end labelling (TUNEL) was performed using the APO-DIRECT kit (BD Biosciences, San Jose, CA, USA), according to the manufacturer's instructions. Flow cytometry of cells was done with a Bio-Rad S3e cell sorter (Bio-Rad Laboratories).

4.12. Cell cycle synchronisation of human cells

For cell synchronisation of HeLa, HCT116, and U-2 OS, asynchronous cell populations were commonly incubated with medium supplemented with 2.5 mM thymidine for at least 14 hours, then washed with thymidine-free medium, and released into the cell cycle by incubation in medium without or with 24 µM deoxycytidine. The latter was applied to allow for more rapidly restoring normal ratios between cellular nucleotide pools and rectifying imbalances due to having been exposed to excess thymidine. The cells were allowed to pass through mitosis, and 9 hours after the release from the first thymidine block, they were once again treated with 2.5 mM thymidine for at least another 14 hours. The cells were then harvested at different time points after the release from this second thymidine block.

Of note, since treatment with thymidine has been reported to affect the cells' welfare and cause a plethora of deleterious effects, like for example resulting in a breakdown in the Na^+/K^+ gradient and influx of Na^+ into HeLa cells [198], and some induction of apoptosis [e.g., 199], and since we also found such treatment causing ZC3HC1 to be more phosphorylated than in the absence of thymidine (our unpublished data), we investigated to which extent these treatments, when performed as outlined above, might (i) perhaps come along with enhanced numbers of apoptotic cells within the finally harvested cell populations, as assessed by IB for PARP integrity, or might (ii) perhaps affect NB-localisation of TPR and ZC3HC1 within the synchronised populations. However, comparing asynchronous populations of cells with such obtained after a release from a first and from a second thymidine block, we neither observed any enhanced PARP degradation nor any conspicuous reduction in the NE-associated amounts of TPR and ZC3HC1 at this time point (our unpublished data).

4.13. Transfection of Xenopus cells with siRNAs and of human cells with expression vectors

Transfections of *Xenopus* cells of line XL-177 with siRNAs, using the latter at about 40 ng of double-stranded siRNA per cm^2 of growth area, were performed with Lipofectamine RNAiMAX (Invitrogen) according to the manufacturer's instructions. These cells were then harvested at 6 days post-transfection. For small scale transfections of cultured human cells with mammalian expression vectors, we used FuGENE 6 (Roche) or PolyJet *In Vitro* DNA Transfection Reagent (SignaGen Laboratories, Frederick, MD, USA), following the manufacturer's instructions. For large scale transfections of adherent HEK293T cells, for the ectopic expression of proteins to be used for IP experiments, we followed a protocol [200] that uses linear 25 kDa polyethylenimine (PEI; Polysciences) instead of branched PEI [201]. This protocol was adjusted for 10 cm culture dishes with about 60-70% cell density. In brief, 45 µg of plasmid DNA were first diluted in 1 ml Opti-MEM (GIBCO), to which 90 µl of a 1 mg/ml PEI stock solution was added, followed by brief mixing via vortexing and subsequent incubation at RT for 15 min. In the meantime, the cells' medium was refreshed, followed by dropwise application of the DNA:PE-containing solution onto the cells, resulting in typical transfection efficiencies of about 50-70%, and occasionally more. Harvest of cells was most commonly 24 hours later.

4.14. Immunoprecipitation of ectopically expressed proteins

Cell extracts containing recombinant versions of ZC3HC1, SKP1 or SKP2, tagged with either the FLAG sequence, different EGFP variants, or mCherry (see Table S5), and ectopically expressed in HEK293T cells treated with U0126 (Cell Signaling Technology, Danvers, MA, USA) or not, were prepared by using a variety of different buffers, by either lysing cells with detergents or by rupturing them via sonication, and by subjecting them to low speed (20,000 g) or high speed (200,000 g) centrifugations. However, since no specific interaction between the core of the SCF complex and ZC3HC1 was noted with any of these different buffers and protocols, only those procedures are described in the following that had made use of the low-speed extracts and corresponded to the data presented in Figures S11 and S12. For each IP experiment, about $1\text{-}2{\cdot}10^7$ cells, first collected in PBS of 30-37°C and then sedimented for 3 min at 1000 g, were resuspended either (i) in an ice-cold NB-destabilising buffer (NB-d-IP) containing 1% TX-100, 130 mM NaCl, 5 mM EDTA, and 10 mM Tris pH 7.4, with this NB-d buffer identical in composition to a buffer used in former IP studies [33,86,97], (ii) in an RT-warm (20-23°C) NB-stabilising buffer (NB-s-IP) with detergent, containing 0.125% TX-100, 41.5 mM KCl, 8.5 mM NaCl, 5 mM $MgCl_2$, 2.5 mM EGTA, 10% sucrose, and 20 mM HEPES, pH 7.25, and this buffer then supplemented with 2 mM DTT or not, depending on whether to be used for IP via sdAbs or via anti-FLAG beads, or (iii) the same NB-s-IP buffer but lacking TX-100, with this buffer then to be used for experiments in which cells were ruptured by sonication. All buffers were always supplemented with protease inhibitors (cOmplete Mini EDTA-free; Roche). Cells resuspended in the TX-100-containing buffers were extracted by vortexing for 4 sec, subsequent incubation for 1 min, either on ice in case of the NB-d-IP buffer, or at RT when using the NB-s-IP buffer, further vortexing for 2 sec, and then immediate centrifugation at either 4°C or RT at 20,000 g for 4 min. Suspensions of cells to be extracted in the absence of detergent were briefly sonicated with either a Hielscher UP50H ultrasonic homogeniser equipped with a sonotrode MS1 (Hielscher Ultrasonics GmbH, Teltow, Germany) or with a SONOPULS mini20, equipped with an MS 2.0 sonotrode (Bandelin Electronic GmbH, Berlin, Germany). Settings for each type of homogeniser had been heuristically determined by series of trial-and-error experiments to result in an adequate, i.e., essentially complete rupturing of suspended HEK293T cells without resulting in the disruption of the NEs primarily into fragments no longer sedimentable at 20,000 g, while at the same time allowing for minimising energy input and keeping the mean rise in temperature within the suspension clearly below two degrees. Such suspensions, too, were then centrifuged at RT and 20,000 g for 4 min. Added to 500 µl of resulting supernatant were then either a total volume of 25 µl of sedimented agarose beads with covalently immobilised sdAbs targeting either GFP/YFP or mCherry (GFP or RFP Selector, respectively; NanoTag Biotechnologies, Göttingen, Germany), or 50 µl of anti-FLAG M2 magnetic beads (Sigma-Aldrich), with both types of beads washed and equilibrated in the corresponding IP buffers in advance, which in the case of the anti-FLAG beads had even been preceded by a pre-treatment with 100 mM glycine pH 2.5. Suspensions in any of the NB-s-IP buffers were then incubated under continuous rotation at RT for 60 min, while suspensions in the NB-d-IP buffer were rotated in the cold room for 120 min. Following subsequent centrifugation of the agarose beads always at 2500 g for 2 min, whilst the anti-FLAG beads were collected via a magnetic field, the beads were washed twice with 400 µl and once with 100 µl of the corresponding IP buffer, with the supernatants always quantitatively removed by using a Pasteur pipette tapered to a very narrow point. Elution of agarose-bead-bound proteins was always done by first resuspending the beads in 50 µl NB-s buffer, followed by adding 50 µl of 2x protein sample buffer (10% SDS, 20% glycerol, 120 mM Tris pH 6.8 plus either 200 mM DTT or 10% 2-mercaptoethanol) and subsequent heating. While anti-FLAG-bead-bound proteins were in some cases eluted in the same manner, such IgG-bound proteins were generally eluted by incubation with 100 mM glycine, pH 2.5, followed by neutralisation of the eluate with K_2HPO_4, before adding 2x protein sample buffer.

4.15. EU and EdU incorporation experiments

In order to monitor replication and transcriptional activity in cells at three days post-transfection with non-target control and ZC3HC1 siRNAs, cell populations were treated with the nucleoside analogues 5-ethynyl-2′-deoxyuridine (EdU, conjugated with Alexa Fluor 488; Click-iT EdU Imaging Kit, Invitrogen) or 5-ethynyl-uridine (EU, conjugated with Alexa Fluor 488; Click-iT EU Imaging Kit, Invitrogen), respectively, following the manufacturer's instructions with some modifications. EU and EdU incorporation were then both analysed by IFM, and EdU incorporation was also quantified spectrophotometrically. In brief, EdU was added to the cell culture medium at a final concentration of 10 µM, while EU was used at a final concentration of 0.5 mM, followed by incubation for one hour, subsequent washes with PBS, fixation with 2.4% FA in PBS for 20 min, permeabilisation with 0.4% TX-100 for 5 min, and further washes with PBS. Subsequent incubation with the Click-iT reaction buffer in 1x reaction buffer was for 30 min, followed by washes in rinse buffer and PBS. Cells treated with EdU were additionally incubated with 3% BSA in PBS, first just prior to and later directly following incubation with the Click-iT reaction buffer. The cells treated with EU that were to be analysed spectrophotometrically by quantifying fluorescence intensities were next labelled with the DNA dye DAPI to later allow for normalisation via DAPI fluorescence, thereby correcting for occasional minor differences in cell numbers between wells. Spectrophotometric quantification of fluorescence emitted from DAPI and Alexa Fluor 488, conjugated to EU, was performed with a BioTek Synergy H4 Hybrid Multi-Mode Microplate Reader (BioTek Instruments, Winooski, VT, USA). For IFM, the cells were conventionally labelled with primary antibodies and followed by secondary antibodies in combination with DNA labelling with dyes like DAPI. Following subsequent washes in PBS, the specimens were mounted with an anti-fade mounting medium like Vectashield (Vector Laboratories). For experiments studying the subcellular ZC3HC1 localisation in transcriptionally active and silenced HeLa cells, they were first treated, for 3 hours, with DMSO for control, or with the transcription inhibitors Actinomycin D (ActD; Sigma-Aldrich), in a concentration of 5 µg/ml, or 5,6-Dichloro-1-β-D-ribofuranosyl-benzimidazole (DRB; Sigma-Aldrich), in a concentration of 75 µg/ml. For cells to be studied by IFM, this was followed by additionally adding EU according to the manufacturer's instruction, further incubation for one hour, and subsequent processing of cells for IFM as described above. For IB, the cells were not treated with EU but harvested and fractionated 4 hours after incubation with only DMSO or transcription inhibitors.

4.16. IFM-compatible fluorescence in situ hybridisation (FISH)

Following fixation with 2.4% FA in PBS for 30 min, quenching with 50 mM NH_4Cl in PBS for 5 min, and subsequent permeabilisation with 0.5% TX-100 in PBS for 7 min, the cells were blocked in 1% BSA in PBS for 30 min and incubated with primary antibodies for one hour. The latter were then cross-linked to their targets by post-fixation with 2.4% FA in PBS for 30 min, followed again by quenching with NH_4Cl in PBS and further washes with PBS. The cells were then washed twice with 2x SSC (saline-sodium citrate) solution (20x SSC: 3 M NaCl, 300 mM trisodium citrate, pH 7.0) at 42°C, followed by incubation in the hybridisation mix (2x SSC, 10% dextran sulfate, 25% formamide, 1.5 mg/ml *E. coli* MRE 600 tRNA [Roche], 1 U/ml RiboLock RNAse inhibitor [Thermo Fisher Scientific, Waltham, MA, USA], 0.01% Tween-20), with 1.5 ng/µl of oligo(dT)50-ATTO488 (Eurofins MWG Operon, Ebersberg, Germany), at 42°C overnight. The specimens were then washed at 42°C in 2x SSC containing 25% formamide for 30 min, in 2x SSC for 30 min, in 1x SSC for 15 min and 0.5x SSC for 15 min, followed by a wash in PBS at RT for 5 min. The cells were then again fixed with 2.4% FA in PBS for 30 min, followed by quenching in 50 mM NH_4Cl in PBS for 5 min and further washes in PBS. They were then incubated with secondary antibodies and DNA dye for one hour. Following

subsequent washes in PBS, the specimens were mounted with an anti-fade mounting medium like SlowFade Gold Antifade Reagent (Invitrogen).

5. Supplemental Tables

Supplemental Table S1: iSEM evaluation datasets.

**all BSE signals assignable to only one of 4 locations: NE, NPCs, and fibre-interconnected or solitary NBs**				
	**NB**	**NPC**	**NE**	**total**
**α-xlNUP153 (40-327)**	24 (5.4)	410 (91.5)	14 (3.1)	448
**α-xlTPR (9-25)**	566 (96.3)	22 (3.7)	0 (0.0)	588
**α-xlZC3HC1 (64-156)**	375 (85.4)	56 (12.8)	8 (1.8)	439
**α-xlZC3HC1 (282-392)**	510 (95.0)	27 (5.0)	0 (0.0)	537
**α-xlZC3HC1 (459-477)**	593 (94.0)	36 (5.7)	2 (0.3)	631
**all BSE signals at solitary NPC-NB units**				
	**NB**	**NPC**	**NE**	**total**
**α-xlNUP153 (40-327)**	11 (4.3)	246 (95.7)	0 (0.0)	257
**α-xlTPR (9-25)**	232 (95.9)	10 (4.1)	0 (0.0)	242
**α-xlZC3HC1 (64-156)**	199 (87.7)	28 (12.3)	0 (0.0)	227
**α-xlZC3HC1 (282-392)**	211 (94.2)	13 (5.8)	0 (0.0)	224
**α-xlZC3HC1 (459-477)**	202 (93.1)	15 (6.9)	0 (0.0)	217
**all solitary NPC-NB unit-associated BSE signals not assignable to IGPs**				
	**NB**	**NPC**	**NE**	**total**
**α-xlNUP153 (40-327)**	2 (0.8)	0 (0.0)	0 (0.0)	257
**α-xlTPR (9-25)**	5 (2.1)	0 (0.0)	0 (0.0)	242
**α-xlZC3HC1 (64-156)**	6 (2.6)	0 (0.0)	0 (0.0)	227
**α-xlZC3HC1 (282-392)**	1 (0.4)	0 (0.0)	0 (0.0)	224
**α-xlZC3HC1 (459-477)**	4 (1.8)	0 (0.0)	0 (0.0)	217
**all solitary NPC-NB unit-associated BSE signals assignable to IGPs**				
	**NB**	**NPC**	**NE**	**total**
**α-xlNUP153 (40-327)**	9 (3.5)	246 (96.5)	0 (0.0)	255
**α-xlTPR (9-25)**	227 (95.8)	10 (4.2)	0 (0.0)	237
**α-xlZC3HC1 (64-156)**	193 (87.3)	28 (12.7)	0 (0.0)	221
**α-xlZC3HC1 (282-392)**	210 (94.2)	13 (5.8)	0 (0.0)	223
**α-xlZC3HC1 (459-477)**	198 (93.0)	15 (7.0)	0 (0.0)	213

Supplemental Table S1: Categories of evaluated BSE and IGP signals. Signals from randomly acquired iSEM micrographs were assigned, by applying different criteria, to different categories, including those that allowed for subsequent radial distance measurements relative to the NPC-NB medial axis. The only category not tabulated included those BSE and IGP signals that could not be unambiguously assigned to either the NB, the NPC or the NE membrane areas between the NPCs. This was so because BSE signals (i) also originated from NE areas that in the SE modus turned out to include NE folds, in which no distinct structure could be unambiguously assigned to a BSE signal, or (ii) corresponded to gold grains that were located at dirt-like particles of unknown origin, or (iii) appeared to stem from additional fibrous materials that laterally interconnected some NBs or had seemingly collapsed onto them. These BSE signals, not further considered in the following, amounted to 41 IGPs for α-xlNUP153 (aa 40-327), 191 IGPs for α-xlTPR (aa 9-25), 143 IGPs for α-xlZC3HC1 (aa 64-156), 276 IGPs for α-xlZC3HC1 (aa 282-392) and 250 IGPs for α-xlZC3HC1 (aa 459-477).

All other signals here summarised in the upper row (termed “all BSE signals assignable to only one of 4 locations: NE, NPCs, and fibre-interconnected or solitary NBs”) thus included all of those BSE signals that appeared to clearly originate from a site either on the surface of the NE, at the NPC, or from an NB. Numbers represent counted BSE signal foci, with fractions relative to each dataset’s total of counted BSE signals listed as percentage values in brackets. However, among these signals were also those originating from NPCs and NBs decorated with IGPs that were excluded from the radial distance measurements. These included such (i) whose NB morphology appeared impaired or that were distorted in such a manner that an unbiased adjustment to the idealistic NB scheme used for subsequent measurements was not possible, and those (ii) that were laterally interconnected to another NB, as exemplified by the light blue arrow-marked pair of excluded NBs shown in Figure S3E. Similarly excluded from those radial distance measurements shown as plots in this study were those signals from NE sites located more than 90 nm away from an NPC-NB medial axis. All remaining BSE signals are tabulated in the second row (here termed “all BSE signals at solitary NPC-NB units”).

Furthermore, this category included BSE signals that could be either assigned or not assigned to IGPs seen in the SE modus. This population of signals was then once more sub-categorised, with signals apportioned to the table’s third and fourth row (here termed “all solitary NPC-NB unit-associated BSE signals not assignable to IGPs” and “all solitary NPC-NB unit-associated BSE signals assignable to IGPs”, respectively). Finally, for all of these, the radial distances to the NB’s medial axis were determined and presented as plots in Figure 2 and Figure S3.

Supplemental Table S2: Antibodies.

Antibody (target region)	Source
Guinea pig polyclonal anti-xlMAD1 (90-109)	This study
Guinea pig polyclonal anti-xlMAD2 (181-203)	This study
Guinea pig polyclonal anti-hsNUP107 (33-51)	[71]
Guinea pig polyclonal anti-hsNUP153 (1459-1475)	[18]
Guinea pig polyclonal anti-xlNUP62 (532-547)	This study
Guinea pig polyclonal anti-xlNUP96 (128-147)	This study
Guinea pig polyclonal anti-xlNUP153 (672-689)	This study
Guinea pig polyclonal anti-hs/xlNUP358 (2285-2314/2108-2132)	[202]
Guinea pig polyclonal anti-hsTPR (2063-2084)	[10]
Guinea pig polyclonal anti-xlTPR (570-587)	This study
Guinea pig polyclonal anti-xlTPR (631-653)	This study
Guinea pig polyclonal anti-xlTPR (2091-2106)	[10]
Guinea pig polyclonal anti-hsZC3HC1 (1-44)	This study
Guinea pig polyclonal anti-hsZC3HC1 (307-355)	This study
Guinea pig polyclonal anti-hsZC3HC1 (343-391)	This study
Guinea pig polyclonal anti-xlZC3HC1 (459-477)	This study
Mouse monoclonal anti-hsCCNB1, clone GNS1 (1-21)	Santa Cruz (sc-245)
Mouse monoclonal anti-FXFG NUPs, clone 414	Covance (MMS-120P)
Mouse monoclonal anti-hsKi-67, clone B56 (1213-1234)	BD Biosciences (550609)
Mouse monoclonal anti-hsLMNB2/xlLamin L_{II}, clone X223 (96-226/85-218)	[48]
Mouse monoclonal anti-hsMAPK1/3, clone L34F12 (250-320)	Cell Signaling Technology (#4696)
Mouse monoclonal anti-hsNeuN/RFOX3, clone A60 (1-21)	Kindly provided by Gabriele Flügge, Merck Millipore (MAB377)
Mouse monoclonal anti-xlNUP62, clone A225 (251-268)	[47,60]
Mouse monoclonal anti-hsNXF1/TAP, clone 53H8	Santa Cruz (sc-32319)
Mouse monoclonal anti-hs/xlSKP1, clone 52/p19	BD Biosciences (610530)
Mouse monoclonal anti-hs/xlSKP1, clone 3F3.1 (50-120)	Merck Millipore (MABS1229)
Mouse monoclonal anti-hsTPR, clone 203-37 (1462-1500)	[10]
Mouse monoclonal anti-hsZC3HC1, clone B-10 (1-60)	Santa Cruz (sc-365058)
Rabbit monoclonal anti-hsMAPK1/3, clone 137F5 (300-360)	Cell Signaling Technology (#4695)
Rabbit monoclonal anti-hsMAPK1/3, clone D13.14.4E (T185p&Y187p/T202p&Y204p)	Cell Signaling Technology (#4370)
Rabbit polyclonal anti-hs/xlCUL1 (111-462)	Proteintech (12895-1-AP)
Rabbit polyclonal anti-hs/xlCUL1 (737-776)	Cohesion Biosciences (CPA2279)
Rabbit polyclonal anti-hs/xlCUL1 (745-776)	Assay Biotech (C0162)
Rabbit polyclonal anti-hsHistone H3 (S10p)	Kindly provided by Wolfgang Fischle
Rabbit polyclonal anti-hsNUP96 (880-900)	[71]
Rabbit polyclonal anti-hsNUP107 (1-338)	Proteintech (19217-1-AP)

Rabbit polyclonal anti-hsNUP153 (50-100)	Abcam (ab84872)
Rabbit polyclonal anti-hsNUP155 (622-707)	Atlas Antibodies (HPA037775)
Rabbit polyclonal anti-hsNUP358 (1592-1607)	Abcam (ab64276)
Rabbit polyclonal anti-xlNUP107 (2-100)	Kindly provided by Dirk Görlich
Rabbit polyclonal anti-xlNUP153 (40-327)	This study
Rabbit polyclonal anti-xlNUP153 (387-533)	[29]
Rabbit polyclonal anti-hs/xlNUP358 (2285-2314/2108-2132)	[202]
Rabbit polyclonal anti-hsPARP (200-250)	Cell Signaling Technology (#9542)
Rabbit polyclonal anti-hsSKP2 (1-344)	Proteintech (15010-1-AP)
Rabbit polyclonal anti-hsTPR (2338-2363)	[53]
Rabbit polyclonal anti-xlTPR (1-17)	This study
Rabbit polyclonal anti-xlTPR (9-25)	This study
Rabbit polyclonal anti-xlTPR (25-41)	This study
Rabbit polyclonal anti-xlTPR (462-479)	This study
Rabbit polyclonal anti-xlTPR (570-587)	This study
Rabbit polyclonal anti-xlTPR (1666-1683)	This study
Rabbit polyclonal anti-xlTPR (2088-2106)	This study
Rabbit polyclonal anti-xlTPR (2091-2106)	[10]
Rabbit polyclonal anti-xlTPR (2304-2321)	This study
Rabbit polyclonal anti-xlZC3HC1 (64-156)	This study
Rabbit polyclonal anti-xlZC3HC1 (169-281)	This study
Rabbit polyclonal anti-xlZC3HC1 (282-392)	This study
Rabbit polyclonal anti-xlZC3HC1 (459-477)	This study
Donkey polyclonal anti-guinea pig IgG (H&L, minimal cross reactions) conjugated with Alexa488, Cy3, Cy5 or HRP	Jackson ImmunoResearch (706-545-148, 706-165-148, 706-175-148 or 706-035-148)
Donkey polyclonal anti-mouse IgG (H&L, minimal cross reactions) conjugated with Alexa488, Cy3, Cy5 or HRP	Jackson ImmunoResearch (715-545-150, 715-165-150, 715-175-150 or 715-035-150)
Donkey polyclonal anti-rabbit IgG (H&L, minimal cross reactions) conjugated with Alexa488, Cy3, Cy5 or HRP	Jackson ImmunoResearch (711-545-152, 711-165-152, 711-175-152 or 711-035-152)
F(ab')$_2$ fragment of goat polyclonal anti-rabbit IgG (H&L) conjugated with 10 nm gold	Aurion (810.166)
FluoTag-X2 anti-mouse(IgG1) sdAb conjugated with Star635P	Nanotag (N2002)

Supplemental Table S2: *Xenopus laevis* target regions relate to the following isoforms of this allotetraploid organism: CUL1 (NP_001089364), LMNB2 (NP_001095239), MAD1 (NP_001080972), MAD2 (NP_001080741), NUP107 (NP_001091312), NUP153 (NP_001082284), NUP358 (XP_018102541), NUP62 (NP_001084339), NUP96 (XP_018101083, with NUP96 aa128-147 corresponding to aa254-273 of this database-deposited sequence, with the latter including additional NUP98 sequence segments at the N-terminus of NUP96), SKP1 (NP_001080844), TPR (XP_018116680) and ZC3HC1 (NP_001083590). Amino acid ranges provided for some antibodies correspond to a mapped region harbouring the epitope for a monoclonal antibody, or to protein segments used for immunisation, or to segments used for purification of subpopulations of antibodies.

Supplemental Table 3: siRNAs.

Target	Sense strand (5′->3′)	Source (order number)
AllStars Negative Control siRNA	proprietary information	Qiagen (SI03650318)
Silencer Select negative control #1	proprietary information	Ambion (4390843)
Silencer Select negative control #2	proprietary information	Ambion (4390846)
siGenome non-targeting siRNA #2	proprietary information	Dharmacon (D-001210-02)
hsCUL1-1	GGCUUGUGGUCGCUUCAUA	Ambion (s228374)
hsCUL1-2	CCGUGAAUGUGACGAAGGA	Ambion (s16055)
hsCUL1-3	GAAUUAUAUAAACGACUUA	Qiagen (SI00053410)
hsCUL1-4	CGUAGUUAUCAGCGAUUCA	Qiagen (SI02225657)
hsMAPK1-1	CAGGGUUCCUGACAGAAUA	Ambion (S11137)
hsMAPK1-2	CAACCAUCGAGCAAAUGAA	Ambion (S11138)
hsMAPK3-1	GGAUCAGCUCAACCACAUU	Ambion (s11140)
hsMAPK3-2	GGACCGGAUGUUAACCUUU	Ambion (s11141)
xlNUP153-1	GAAGGAAGGGAGUGUGUUA	Dharmacon (custom)
hsNXF1-1	CTGGATGACATGTCTAGCA	Qiagen (SI04307492)
hsNXF1-2	CGAACGAUUUCCCAAGUUA	Ambion (s20532)
hsNXF1-3	CGAAGGAUAUCUAUCAUCA	Ambion (s20533)
hsSKP1-1	GGAACACUUUUUGAACUCA	Ambion (s12889)
hsSKP1-2	AACAAUCUGUGACUAUUAA	Ambion (s12890)
hsSKP1-3	GAUGAUGAGAACAAAGAAA	Qiagen (SI02758707)
hsSKP1-4	AAACAAUCUGUGACUAUUA	Qiagen (SI02777411)
hsSKP2-1	GAAUCUUAGCGGCUACAGA	Ambion (s12892)
hsSKP2-2	GGCCUAAGCUAAAUCGAGA	Ambion (s12893)
hsTPR-1	CAAUAUGAAGGUCGAAUUA	Ambion (s14355)
hsTPR-2	GAAGUUCAUACUAAGCGUA	Ambion (s14354)
hsTPR-3	GAGUCUGCGUUAUCGACAA	Ambion (s14353)
xlTPR-1	UGAAGGAAUUGGUGCAGAA	Dharmacon (custom)
hsZC3HC1-1	GAACAACCUUCAUUGGAAU	Ambion (s28276)
hsZC3HC1-2	CAAGGAAAGUAUUCCGAAU	Ambion (s28277)
hsZC3HC1-3	CACUCGUCUGUGCAAAAUA	Ambion (s28278)
hsZC3HC1-4	UGCUAUUCUUGUUAGUGAA	Qiagen (SI00116305)
hsZC3HC1-5	GCCACAUCCCAGUCAGUUA	Qiagen (SI03649905)
xlZC3HC1-1	GCAUGGACAUCACAGAAGA	Dharmacon (custom)
xlZC3HC1-2	GUUAAAGUGUUCAAGUUGU	Dharmacon (custom)

Supplemental Table S3: Dharmacon siRNAs against *Xenopus laevis* mRNAs were designed using the siDesign Center (https://dharmacon.horizondiscovery.com/design-center/). The Thermo Fisher Scientific/Ambion siRNAs all belonged to the product type “Silencer Select”. Note that the siRNA numbering, in the left column, also including the Qiagen siRNAs used for the oldest series of RNAi experiments, does not reflect their purchase order.

Supplemental Table S4: Cell lines.

**Cell line**	**Source**
HCT116	RRID:CVCL_0291 (CCL-247; ATCC, Manassas, VA, USA)
HCT116 ZC3HC1 KO, clone #1	This study
HCT116 ZC3HC1 KO, clone #2	This study
HEK293T	RRID:CVCL_0063 (CRL-3216; ATCC)
HeLa P2	High passage # of RRID:CVCL_0030 (CCL-2; ATCC); see Information SI 11
HeLa P2 ZC3HC1 KO	This study
HeLa W	RRID:CVCL_WN71, kindly provided by Dirk Görlich
hTCEpi	RRID:CVCL_AQ44 (Evercyte, Vienna, Austria)
hTCEpi ZC3HC1 KO	This study
U-2 OS	RRID:CVCL_0042 (HTB-96; ATCC)
U-2 OS ZC3HC1 KO	This study
XL-177	RRID:CVCL_T684, kindly provided by Werner Franke

Supplemental Table S5: Mammalian expression vectors.

Expression construct	Backbone (resistance)	Source
EGFP	pEGFP-N1 (Kan)	Clontech
hsZC3HC1(1-502 ǀ H363)-EGFP	pEGFP-N1 (Kan)	This study
EGFP-xlZC3HC1(1-477 ǀ isoform 1 ǀ NP_001083590)	pEGFP-C1 (Kan)	This study
EGFP-xlZC3HC1(1-473 ǀ isoform 2 ǀ XP_018111179)	pEGFP-C1 (Kan)	This study
EYFP	pEYFP-C1 (Kan)	Clontech
EYFP-hsSKP1a(1-163)	pEYFP-C1 (Kan)	This study
EYFP-hsZC3HC1(1-502 ǀ H363)	pEYFP-C1 (Kan)	This study
EYFP-hsZC3HC1(50-402 ǀ H363)	pEYFP-C1 (Kan)	This study
EYFP-hsZC3HC1(1-502 ǀ R363)	pEYFP-C1 (Kan)	This study
FLAG-hsZC3HC1(1-502 ǀ H363)	pEYFP-C1 (Kan)	This study
mCherry	pEYFP-C1 (Kan)	This study
mCherry-hsSKP1a(1-163)	pEYFP-C1 (Kan)	This study
mCherry-hsSKP1b(1-160)	pEYFP-C1 (Kan)	This study
mCherry-hsSKP2(1-424)	pEYFP-C1 (Kan)	This study
mCherry-hsZC3HC1(1-502 ǀ H363)	pEYFP-C1 (Kan)	This study
mCherry-hsZC3HC1(1-502 ǀ R363)	pEYFP-C1 (Kan)	This study
hsZC3HC1(1-502 ǀ H363)-mCherry	pEYFP-C1 (Kan)	This study
hsSKP1a(1-163)-mCherry	pEYFP-C1 (Kan)	This study
mEGFP (L221K)	pEYFP-C1 (Kan)	This study
mEGFP-hsSKP1a(1-163)	pEYFP-C1 (Kan)	This study
mEGFP-hsSKP1b(1-160)	pEYFP-C1 (Kan)	This study
mEGFP-hsSKP2(1-424)	pEYFP-C1 (Kan)	This study
mEGFP-hsZC3HC1(1-502 ǀ H363)	pEYFP-C1 (Kan)	This study
mEGFP-hsZC3HC1(1-502 ǀ R363)	pEYFP-C1 (Kan)	This study
mEGFP-xlZC3HC1(1-477 ǀ isoform 1 ǀ NP_001083590)	pEGFP-C1 (Kan)	This study
hsSKP1a(1-163)	pEYFP-C1 (Kan)	This study
hsSKP1b(1-160)	pEYFP-C1 (Kan)	This study
hSpCas9n(BB)-2A-Puro	PX462 V2.0 (Amp)	[203], gift from Feng Zhang (Addgene plasmid # 62987)
sgRNA1 (hsZC3HC1) / hSpCas9n(BB)-2A-Puro	PX462 V2.0 (Amp)	This study
sgRNA2 (hsZC3HC1) / hSpCas9n(BB)-2A-Puro	PX462 V2.0 (Amp)	This study
sgRNA3 (hsZC3HC1) / hSpCas9n(BB)-2A-Puro	PX462 V2.0 (Amp)	This study
sgRNA4 (hsZC3HC1) / hSpCas9n(BB)-2A-Puro	PX462 V2.0 (Amp)	This study
Rev/Gr/GFP	pGreenLantern-1 (Amp)	[152], kindly provided by John Hanover
GFP_2-RanBP1	pK7-GFP (Amp)	[153], kindly provided by Ian Macara

Author Contributions: Classification and degree of contribution according to CRediT taxonomy [204,205]. Conceptualization, V.C.C. (L) and P.G. (S); data curation, P.G. (L) and V.C.C. (S); formal analysis, P.G. (E), V.C.C. (E), H.I. (S), and S.K. (S); investigation, P.G. (L), V.C.C. (E), H.I. (S), and S.K. (S); methodology, V.C.C. (L) P.G. (S), and S.K. (S); project administration, V.C.C. (L), P.G. (E) and S.K. (S); resources, V.C.C.; supervision, V.C.C.; validation, P.G. (E), V.C.C. (E), S.K. (S), and H.I. (S); visualization, P.G. (L), V.C.C. (S), and H.I. (S); writing—original draft preparation, V.C.C. (L) and P.G. (S); writing—review and editing, V.C.C. (L), P.G. (L), S.K. (E), and H.I. (S). All authors have read and agreed to the submitted version of the manuscript.

Funding: This research received no external funding.

Institutional Review Board Statement: All animal husbandry and handling were in accordance with German Law and with approval by the Veterinary Institute of LAVES (Niedersächsisches Landesamt für Verbraucherschutz und Lebensmittelsicherheit) (33.42502-05/A-005/07).

Informed Consent Statement: Not applicable

Data Availability Statement: The data presented in this study are available on request to the corresponding author.

Acknowledgments: We gratefully acknowledge Thomas Ruppert, Armin Bosserhoff, and Margrit Ellis at the core facility for mass spectrometry at the Center for Molecular Biology in Heidelberg (ZMBH) and Henning Urlaub and Uwe Plessmann at the MPIBPC for contributions to mass spectrometric analyses of *Xenopus* proteins, and Ulrike Mau-Holzmann at the Institute for Medical Genetics and Applied Genomics in Tübingen for contributions to the karyotyping of cell lines. In addition, we wish to thank Hans-Richard Rackwitz at the Peptide Specialty Laboratories in Heidelberg for peptide syntheses, and Julia Dörries-Iino at the MPIBPC for technical assistance in immuno-TEM. Furthermore, we thank Dirk Görlich, Reinhard Lührmann, Dietmar Riedel and Paul Walther for sharing equipment, as well as Heinz-Jürgen Dehne, Wolfgang Fischle, Gabriele Flügge, Werner Franke, Steffen Frey, Dirk Görlich, Lareen Gräser, John Hanover, Bastian Hülsmann, Georg Krohne, Jens Krull, Ian Macara, Ian Mattaj, Michael Ratz, Kerstin Mätz-Rensing, Dietmar Riedel, Uli Scheer, Ulrike Teichmann, Sergei Trakhanov, Paul Walther and Feng Zhang for kindly providing research materials, technical advice or helpful suggestions. Finally, we appreciate Georg Krohne for the critical reading of the manuscript.

Conflicts of Interest: The authors declare no conflict of interest.

Abbreviations: aa, amino acids; AL, annulate lamellae; ALPC, annulate lamellae pore complexes; BSE, backscattered electron; CRISPR/Cas9n, Clustered Regularly Interspaced Short Palindromic Repeats/CRISPR-associated protein 9 nickase; CT, carboxy-terminus; FA, formaldehyde; GA, glutaraldehyde; IB, immunoblotting; IFM, immunofluorescence microscopy; IGP, immunogold particle; iSEM, immuno-scanning electron microscopy; iTEM, immuno-transmission electron microscopy; KD, knockdown; KO, knockout; LNN, lamina-NPC-NB; LUT, look-up table; NB, nuclear basket; NB-d, nuclear basket-destabilising; NBLS, NB-like structures; NB-s, nuclear basket-stabilising; NE, nuclear envelope; NPBD, nuclear pore complex-binding domain; NMBD, nuclear membrane-binding domain; NPC, nuclear pore complex; NR, nuclear ring; NT, amino-terminus; NUP, nucleoporin; PS, Ponceau S; RNAi, RNA interference; SD, standard deviation; SEM, scanning electron microscope/microscopy; siRNA, small interfering RNA; TBD, TPR-binding domain; TEM, transmission electron microscope/microscopy; TR, terminal ring.

Supplemental References

1. Scheer, U.; Dabauvalle, M.C.; Merkert, H.; Benevente, R. The Nuclear Envelope and the Organization of the Pore Complexes. *Cell Biol. Int. Rep.* **1988**, *12*, 669–689, doi:10.1006/jsbi.1996.0066.
2. Goldberg, M.W.; Allen, T.D. High Resolution Scanning Electron Microscopy of the Nuclear Envelope: Demonstration of a New, Regular, Fibrous Lattice Attached to the Baskets of the Nucleoplasmic Face of the Nuclear Pores. *J. Cell Biol.* **1992**, *119*, 1429–1440, doi:10.1083/jcb.119.6.1429.
3. Ris, H.; Malecki, M. High-Resolution Field Emission Scanning Electron Microscope Imaging of Internal Cell Structures after Epon Extraction from Sections: A New Approach to Correlative Ultrastructural and Immunocytochemical Studies. *J. Struct. Biol.* **1993**, *111*, 148–157, doi:10.1006/jsbi.1993.1045.
4. Cordes, V.C.; Reidenbach, S.; Köhler, A.; Stuurman, N.; van Driel, R.; Franke, W.W. Intranuclear Filaments Containing a Nuclear Pore Complex Protein. *J. Cell Biol.* **1993**, *123*, 1333–1344, doi:10.1083/jcb.123.6.1333.
5. Arlucea, J.; Andrade, R.; Alonso, R.; Aréchaga, J. The Nuclear Basket of the Nuclear Pore Complex Is Part of a Higher-Order Filamentous Network That Is Related to Chromatin. *J. Struct. Biol.* **1998**, *124*, 51–58, doi:10.1006/jsbi.1998.4054.

6. Kiseleva, E.; Drummond, S.P.; Goldberg, M.W.; Rutherford, S.A.; Allen, T.D.; Wilson, K.L. Actin- and Protein-4.1-Containing Filaments Link Nuclear Pore Complexes to Subnuclear Organelles in Xenopus Oocyte Nuclei. *J. Cell Sci.* **2004**, *117*, 2481–2490, doi:10.1242/jcs.01098.
7. Walther, P. High-Resolution Cryo-SEM Allows Direct Identification of F-Actin at the Inner Nuclear Membrane of Xenopus Oocytes by Virtue of Its Structural Features. *J. Microsc.* **2008**, *232*, 379–385, doi:10.1111/j.1365-2818.2008.02109.x.
8. Hase, M.E.; Kuznetsov, N. V; Cordes, V.C. Amino Acid Substitutions of Coiled-Coil Protein TPR Abrogate Anchorage to the Nuclear Pore Complex but Not Parallel, in-Register Homodimerization. *Mol. Biol. Cell* **2001**, *12*, 2433–2452, doi:10.1091/mbc.12.8.2433.
9. Krull, S.; Thyberg, J.; Björkroth, B.; Rackwitz, H.-R.; Cordes, V.C. Nucleoporins as Components of the Nuclear Pore Complex Core Structure and TPR as the Architectural Element of the Nuclear Basket. *Mol. Biol. Cell* **2004**, *15*, 4261–4277, doi:10.1091/mbc.e04-03-0165.
10. Cordes, V.C.; Reidenbach, S.; Rackwitz, H.R.; Franke, W.W. Identification of Protein P270/TPR as a Constitutive Component of the Nuclear Pore Complex-Attached Intranuclear Filaments. *J. Cell Biol.* **1997**, *136*, 515–529, doi:10.1083/jcb.136.3.515.
11. Kiseleva, E.; Goldberg, M.W.; Daneholt, B.; Allen, T.D. RNP Export Is Mediated by Structural Reorganization of the Nuclear Pore Basket. *J. Mol. Biol.* **1996**, *260*, 304–311, doi:10.1006/jmbi.1996.0401.
12. Dumont, J.N. Oogenesis in Xenopus Laevis (Daudin). I. Stages of Oocyte Development in Laboratory Maintained Animals. *J. Morphol.* **1972**, *136*, 153–179, doi:10.1002/jmor.1051360203.
13. Kleinschmidt, J.A.; Scheer, U.; Dabauvalle, M.C.; Bustin, M.; Franke, W.W. High Mobility Group Proteins of Amphibian Oocytes: A Large Storage Pool of a Soluble High Mobility Group-1-like Protein and Involvement in Transcriptional Events. *J. Cell Biol.* **1983**, *97*, 838–848, doi:10.1083/jcb.97.3.838.
14. Ori, A.; Banterle, N.; Iskar, M.; Andrés-Pons, A.; Escher, C.; Khanh Bui, H.; Sparks, L.; Solis-Mezarino, V.; Rinner, O.; Bork, P.; et al. Cell Type-Specific Nuclear Pores: A Case in Point for Context-Dependent Stoichiometry of Molecular Machines. *Mol. Syst. Biol.* **2013**, *9*, 648, doi:10.1038/msb.2013.4.
15. Eibauer, M.; Pellanda, M.; Turgay, Y.; Dubrovsky, A.; Wild, A.; Medalia, O. Structure and Gating of the Nuclear Pore Complex. *Nat. Commun.* **2015**, *6*, 7532, doi:10.1038/ncomms8532.
16. Holzer, G.; Antonin, W. Breaking the Y. *PLoS Genet.* **2019**, *15*, e1008109, doi:10.1371/journal.pgen.1008109.
17. Zhang, Y.; Li, S.; Zeng, C.; Huang, G.; Zhu, X.; Wang, Q.; Wang, K.; Zhou, Q.; Yan, C.; Zhang, W.; et al. Molecular Architecture of the Luminal Ring of the Xenopus Laevis Nuclear Pore Complex. *Cell Res.* **2020**, *30*, 532–540, doi:10.1038/s41422-020-0320-y.
18. Krull, S.; Dörries, J.; Boysen, B.; Reidenbach, S.; Magnius, L.; Norder, H.; Thyberg, J.; Cordes, V.C. Protein TPR Is Required for Establishing Nuclear Pore-Associated Zones of Heterochromatin Exclusion. *EMBO J.* **2010**, *29*, 1659–1673, doi:10.1038/emboj.2010.54.
19. Galy, V.; Gadal, O.; Fromont-Racine, M.; Romano, A.; Jacquier, A.; Nehrbass, U. Nuclear Retention of Unspliced MRNAs in Yeast Is Mediated by Perinuclear Mlp1. *Cell* **2004**, *116*, 63–73, doi:10.1016/s0092-8674(03)01026-2.
20. Palancade, B.; Zuccolo, M.; Loeillet, S.; Nicolas, A.; Doye, V. Pml39, a Novel Protein of the Nuclear Periphery Required for Nuclear Retention of Improper Messenger Ribonucleoparticles. *Mol. Biol. Cell* **2005**, *16*, 5258–5268, doi:10.1091/mbc.e05-06-0527.
21. Léger-Silvestre, I.; Trumtel, S.; Noaillac-Depeyre, J.; Gas, N. Functional Compartmentalization of the Nucleus in the Budding Yeast Saccharomyces Cerevisiae. *Chromosoma* **1999**, *108*, 103–113, doi:10.1007/s004120050357.
22. Herrup, K.; Yang, Y. Cell Cycle Regulation in the Postmitotic Neuron: Oxymoron or New Biology? *Nat. Rev. Neurosci.* **2007**, *8*, 368–378, doi:10.1038/nrn2124.
23. Ninkovic, J.; Götz, M. Fate Specification in the Adult Brain - Lessons for Eliciting Neurogenesis from Glial Cells. *Bioessays* **2013**, *35*, 242–252, doi:10.1002/bies.201200108.
24. Bergmann, O.; Frisén, J. Neuroscience. Why Adults Need New Brain Cells. *Science* **2013**, *340*, 695–696, doi:10.1126/science.1237976.
25. Franke, W.W.; Scheer, U. The Ultrastructure of the Nuclear Envelope of Amphibian Oocytes: A Reinvestigation. I. The Mature Oocyte. *J. Ultrastruct. Res.* **1970**, *30*, 288–316, doi:10.1016/s0022-5320(70)80064-8.
26. Scheer, U.; Franke, W.W. Negative Staining and Adenosine Triphosphatase Activity of Annulate Lamellae of Newt Oocytes. *J. Cell Biol.* **1969**, *42*, 519–533, doi:10.1083/jcb.42.2.519.
27. Scheer, U.; Kartenbeck, J.; Trendelenburg, M.F.; Stadler, J.; Franke, W.W. Experimental Disintegration of the Nuclear Envelope. Evidence for Pore-Connecting Fibrils. *J. Cell Biol.* **1976**, *69*, 1–18, doi:10.1083/jcb.69.1.1.
28. Jarnik, M.; Aebi, U. Toward a More Complete 3-D Structure of the Nuclear Pore Complex. *J. Struct. Biol.* **1991**, *107*, 291–308, doi:10.1016/1047-8477(91)90054-z.
29. Walther, T.C.; Fornerod, M.; Pickersgill, H.; Goldberg, M.; Allen, T.D.; Mattaj, I.W. The Nucleoporin NUP153 Is Required for Nuclear Pore Basket Formation, Nuclear Pore Complex Anchoring and Import of a Subset of Nuclear Proteins. *EMBO J.* **2001**, *20*, 5703–5714, doi:10.1093/emboj/20.20.5703.
30. Cronshaw, J.M.; Krutchinsky, A.N.; Zhang, W.; Chait, B.T.; Matunis, M.J. Proteomic Analysis of the Mammalian Nuclear Pore Complex. *J. Cell Biol.* **2002**, *158*, 915–927, doi:10.1083/jcb.200206106.
31. Zhang, Q.H.; Ye, M.; Wu, X.Y.; Ren, S.X.; Zhao, M.; Zhao, C.J.; Fu, G.; Shen, Y.; Fan, H.Y.; Lu, G.; et al. Cloning and Functional Analysis of CDNAs with Open Reading Frames for 300 Previously Undefined Genes Expressed in CD34+ Hematopoietic Stem/Progenitor Cells. *Genome Res.* **2000**, *10*, 1546–1560, doi:10.1101/gr.140200.

32. Strausberg, R.L.; Feingold, E.A.; Grouse, L.H.; Derge, J.G.; Klausner, R.D.; Collins, F.S.; Wagner, L.; Shenmen, C.M.; Schuler, G.D.; Altschul, S.F.; et al. Generation and Initial Analysis of More than 15,000 Full-Length Human and Mouse CDNA Sequences. *Proc. Natl. Acad. Sci. U. S. A.* **2002**, *99*, 16899–16903, doi:10.1073/pnas.242603899.
33. Bassermann, F.; Klitzing, C. von; Münch, S.; Bai, R.-Y.; Kawaguchi, H.; Morris, S.W.; Peschel, C.; Duyster, J. NIPA Defines an SCF-Type Mammalian E3 Ligase That Regulates Mitotic Entry. *Cell* **2005**, *122*, 45–57, doi:10.1016/j.cell.2005.04.034.
34. Blethrow, J.D.; Glavy, J.S.; Morgan, D.O.; Shokat, K.M. Covalent Capture of Kinase-Specific Phosphopeptides Reveals Cdk1-Cyclin B Substrates. *Proc. Natl. Acad. Sci. U. S. A.* **2008**, *105*, 1442–1447, doi:10.1073/pnas.0708966105.
35. Dephoure, N.; Zhou, C.; Villén, J.; Beausoleil, S.A.; Bakalarski, C.E.; Elledge, S.J.; Gygi, S.P. A Quantitative Atlas of Mitotic Phosphorylation. *Proc. Natl. Acad. Sci. U. S. A.* **2008**, *105*, 10762–10767, doi:10.1073/pnas.0805139105.
36. Sharma, K.; D'Souza, R.C.J.; Tyanova, S.; Schaab, C.; Wiśniewski, J.R.; Cox, J.; Mann, M. Ultradeep Human Phosphoproteome Reveals a Distinct Regulatory Nature of Tyr and Ser/Thr-Based Signaling. *Cell Rep.* **2014**, *8*, 1583–1594, doi:10.1016/j.celrep.2014.07.036.
37. Session, A.M.; Uno, Y.; Kwon, T.; Chapman, J.A.; Toyoda, A.; Takahashi, S.; Fukui, A.; Hikosaka, A.; Suzuki, A.; Kondo, M.; et al. Genome Evolution in the Allotetraploid Frog Xenopus Laevis. *Nature* **2016**, *538*, 336–343, doi:10.1038/nature19840.
38. Hellsten, U.; Khokha, M.K.; Grammer, T.C.; Harland, R.M.; Richardson, P.; Rokhsar, D.S. Accelerated Gene Evolution and Subfunctionalization in the Pseudotetraploid Frog Xenopus Laevis. *BMC Biol.* **2007**, *5*, 31, doi:10.1186/1741-7007-5-31.
39. Itami, C.; Ishida, R.; Utsumi, K.R.; Nakabayashi, T. Variation in Three Groups of HeLa Cell Sublines as Revealed by Karyotype Analysis and O6-Methylguanine-DNA-Methyltransferase Activity. *Tissue Cult. Res. Commun.* **1998**, *17*, 101–106, doi:10.11418/jtca1981.17.3_101.
40. Macville, M.; Schröck, E.; Padilla-Nash, H.; Keck, C.; Ghadimi, B.M.; Zimonjic, D.; Popescu, N.; Ried, T. Comprehensive and Definitive Molecular Cytogenetic Characterization of HeLa Cells by Spectral Karyotyping. *Cancer Res.* **1999**, *59*, 141–150.
41. Rutledge, S. What HeLa Cells Are You Using? *The Winnower* **2014**, 1–9, doi:10.15200/winn.143896.65158.
42. Adey, A.; Burton, J.N.; Kitzman, J.O.; Hiatt, J.B.; Lewis, A.P.; Martin, B.K.; Qiu, R.; Lee, C.; Shendure, J. The Haplotype-Resolved Genome and Epigenome of the Aneuploid HeLa Cancer Cell Line. *Nature* **2013**, *500*, 207–211, doi:10.1038/nature12064.
43. Landry, J.J.M.; Pyl, P.T.; Rausch, T.; Zichner, T.; Tekkedil, M.M.; Stütz, A.M.; Jauch, A.; Aiyar, R.S.; Pau, G.; Delhomme, N.; et al. The Genomic and Transcriptomic Landscape of a HeLa Cell Line. *G3* **2013**, *3*, 1213–1224, doi:10.1534/g3.113.005777.
44. Weidenfeld, I.; Gossen, M.; Löw, R.; Kentner, D.; Berger, S.; Görlich, D.; Bartsch, D.; Bujard, H.; Schönig, K. Inducible Expression of Coding and Inhibitory RNAs from Retargetable Genomic Loci. *Nucleic Acids Res.* **2009**, *37*, e50, doi:10.1093/nar/gkp108.
45. Sievers, F.; Wilm, A.; Dineen, D.; Gibson, T.J.; Karplus, K.; Li, W.; Lopez, R.; McWilliam, H.; Remmert, M.; Söding, J.; et al. Fast, Scalable Generation of High-Quality Protein Multiple Sequence Alignments Using Clustal Omega. *Mol. Syst. Biol.* **2011**, *7*, 539, doi:10.1038/msb.2011.75.
46. Wühr, M.; Freeman, R.M.; Presler, M.; Horb, M.E.; Peshkin, L.; Gygi, S.; Kirschner, M.W. Deep Proteomics of the Xenopus Laevis Egg Using an MRNA-Derived Reference Database. *Curr. Biol.* **2014**, *24*, 1467–1475, doi:10.1016/j.cub.2014.05.044.
47. Cordes, V.C.; Gajewski, A.; Stumpp, S.; Krohne, G. Immunocytochemistry of Annulate Lamellae: Potential Cell Biological Markers for Studies of Cell Differentiation and Pathology. *Differentiation.* **1995**, *58*, 307–312, doi:10.1046/j.1432-0436.1995.5840307.x.
48. Höger, T.H.; Zatloukal, K.; Waizenegger, I.; Krohne, G. Characterization of a Second Highly Conserved B-Type Lamin Present in Cells Previously Thought to Contain Only a Single B-Type Lamin. *Chromosoma* **1990**, *99*, 379–390, doi:10.1007/BF01726689.
49. Fichtman, B.; Shaulov, L.; Harel, A. Imaging Metazoan Nuclear Pore Complexes by Field Emission Scanning Electron Microscopy. *Methods Cell Biol.* **2014**, *122*, 41–58, doi:10.1016/B978-0-12-417160-2.00002-3.
50. Goldberg, M.W. High-Resolution Scanning Electron Microscopy and Immuno-Gold Labeling of the Nuclear Lamina and Nuclear Pore Complex. *Methods Mol. Biol.* **2016**, *1411*, 441–459, doi:10.1007/978-1-4939-3530-7_27.
51. Goldberg, M.W.; Fišerová, J. Immunogold Labeling for Scanning Electron Microscopy. *Methods Mol. Biol.* **2016**, *1474*, 309–325, doi:10.1007/978-1-4939-6352-2_20.
52. Goldberg, M.W.; Allen, T.D. The Nuclear Pore Complex and Lamina: Three-Dimensional Structures and Interactions Determined by Field Emission in-Lens Scanning Electron Microscopy. *J. Mol. Biol.* **1996**, *257*, 848–865, doi:10.1006/jmbi.1996.0206.
53. Kuznetsov, N. V; Sandblad, L.; Hase, M.E.; Hunziker, A.; Hergt, M.; Cordes, V.C. The Evolutionarily Conserved Single-Copy Gene for Murine TPR Encodes One Prevalent Isoform in Somatic Cells and Lacks Paralogs in Higher Eukaryotes. *Chromosoma* **2002**, *111*, 236–255, doi:10.1007/s00412-002-0208-2.
54. Zimmermann, L.; Stephens, A.; Nam, S.-Z.; Rau, D.; Kübler, J.; Lozajic, M.; Gabler, F.; Söding, J.; Lupas, A.N.; Alva, V. A Completely Reimplemented MPI Bioinformatics Toolkit with a New HHpred Server at Its Core. *J. Mol. Biol.* **2018**, *430*, 2237–2243, doi:10.1016/j.jmb.2017.12.007.
55. Sellés, J.; Penrad-Mobayed, M.; Guillaume, C.; Fuger, A.; Auvray, L.; Faklaris, O.; Montel, F. Nuclear Pore Complex Plasticity during Developmental Process as Revealed by Super-Resolution Microscopy. *Sci. Rep.* **2017**, *7*, 14732, doi:10.1038/s41598-017-15433-2.
56. Grote, M.; Kubitscheck, U.; Reichelt, R.; Peters, R. Mapping of Nucleoporins to the Center of the Nuclear Pore Complex by Post-Embedding Immunogold Electron Microscopy. *J. Cell Sci.* **1995**, *108*, 2963–2972.
57. Heasman, J.; Quarmby, J.; Wylie, C.C. The Mitochondrial Cloud of Xenopus Oocytes: The Source of Germinal Granule Material. *Dev. Biol.* **1984**, *105*, 458–469, doi:10.1016/0012-1606(84)90303-8.

58. Kessel, R.G. The Annulate Lamellae - from Obscurity to Spotlight. *Electron. Microsc. Rev.* **1989**, *2*, 257–348, doi:10.1016/0892-0354(89)90003-8.
59. Kessel, R.G. Annulate Lamellae: A Last Frontier in Cellular Organelles. *Int. Rev. Cytol.* **1992**, *133*, 43–120, doi:10.1016/s0074-7696(08)61858-6.
60. Cordes, V.C.; Reidenbach, S.; Franke, W.W. High Content of a Nuclear Pore Complex Protein in Cytoplasmic Annulate Lamellae of Xenopus Oocytes. *Eur. J. Cell Biol.* **1995**, *68*, 240–255, doi:10.1073/pnas.0908269107.
61. Hagler, H.K. Ultramicrotomy for Biological Electron Microscopy. *Methods Mol. Biol.* **2007**, *369*, 67–96, doi:10.1007/978-1-59745-294-6_5.
62. Watson, M.L. Further Observations on the Nuclear Envelope of the Animal Cell. *J. Biophys. Biochem. Cytol.* **1959**, *6*, 147–156, doi:10.1083/jcb.6.2.147.
63. Walther, T.C.; Pickersgill, H.S.; Cordes, V.C.; Goldberg, M.W.; Allen, T.D.; Mattaj, I.W.; Fornerod, M. The Cytoplasmic Filaments of the Nuclear Pore Complex Are Dispensable for Selective Nuclear Protein Import. *J. Cell Biol.* **2002**, *158*, 63–77, doi:10.1083/jcb.200202088.
64. Kiseleva, E.; Richardson, A.C.; Fiserova, J.; Strunov, A.A.; Spink, M.C.; Johnson, S.R.; Goldberg, M.W. Imaging Yeast NPCs: From Classical Electron Microscopy to Immuno-SEM. *Methods Cell Biol.* **2014**, *122*, 59–79, doi:10.1016/B978-0-12-417160-2.00003-5.
65. Maimon, T.; Elad, N.; Dahan, I.; Medalia, O. The Human Nuclear Pore Complex as Revealed by Cryo-Electron Tomography. *Structure* **2012**, *20*, 998–1006, doi:10.1016/j.str.2012.03.025.
66. Ris, H. High-Resolution Field-Emission Scanning Electron Microscopy of Nuclear Pore Complex. *Scanning* **1997**, *19*, 368–375, doi:10.1002/sca.4950190504.
67. Malecki, M.; Malecki, B. Routing of Biomolecules and Transgenes' Vectors in Nuclei of Oocytes. *J. Fertili. In Vitro* **2012**, *2012*, 108–118, doi:10.4172/2165-74.
68. Goldberg, M.W.; Solovei, I.; Allen, T.D. Nuclear Pore Complex Structure in Birds. *J. Struct. Biol.* **1997**, *119*, 284–294, doi:10.1006/jsbi.1997.3877.
69. Bohnsack, M.T.; Stüven, T.; Kuhn, C.; Cordes, V.C.; Görlich, D. A Selective Block of Nuclear Actin Export Stabilizes the Giant Nuclei of Xenopus Oocytes. *Nat. Cell Biol.* **2006**, *8*, 257–263, doi:10.1038/ncb1357.
70. Bensaude, O. Inhibiting Eukaryotic Transcription: Which Compound to Choose? How to Evaluate Its Activity? *Transcription* **2011**, *2*, 103–108, doi:10.4161/trns.2.3.16172.
71. Hase, M.E.; Cordes, V.C. Direct Interaction with NUP153 Mediates Binding of TPR to the Periphery of the Nuclear Pore Complex. *Mol. Biol. Cell* **2003**, *14*, 1923–1940, doi:10.1091/mbc.e02-09-0620.
72. Dultz, E.; Zanin, E.; Wurzenberger, C.; Braun, M.; Rabut, G.; Sironi, L.; Ellenberg, J. Systematic Kinetic Analysis of Mitotic Dis- and Reassembly of the Nuclear Pore in Living Cells. *J. Cell Biol.* **2008**, *180*, 857–865, doi:10.1083/jcb.200707026.
73. Laurell, E.; Beck, K.; Krupina, K.; Theerthagiri, G.; Bodenmiller, B.; Horvath, P.; Aebersold, R.; Antonin, W.; Kutay, U. Phosphorylation of NUP98 by Multiple Kinases Is Crucial for NPC Disassembly during Mitotic Entry. *Cell* **2011**, *144*, 539–550, doi:10.1016/j.cell.2011.01.012.
74. Mendjan, S.; Taipale, M.; Kind, J.; Holz, H.; Gebhardt, P.; Schelder, M.; Vermeulen, M.; Buscaino, A.; Duncan, K.; Mueller, J.; et al. Nuclear Pore Components Are Involved in the Transcriptional Regulation of Dosage Compensation in Drosophila. *Mol. Cell* **2006**, *21*, 811–823, doi:10.1016/j.molcel.2006.02.007.
75. Sabri, N.; Roth, P.; Xylourgidis, N.; Sadeghifar, F.; Adler, J.; Samakovlis, C. Distinct Functions of the Drosophila NUP153 and NUP214 FG Domains in Nuclear Protein Transport. *J. Cell Biol.* **2007**, *178*, 557–565, doi:10.1083/jcb.200612135.
76. Umlauf, D.; Bonnet, J.; Waharte, F.; Fournier, M.; Stierle, M.; Fischer, B.; Brino, L.; Devys, D.; Tora, L. The Human TREX-2 Complex Is Stably Associated with the Nuclear Pore Basket. *J. Cell Sci.* **2013**, *126*, 2656–2667, doi:10.1242/jcs.118000.
77. Vollmer, B.; Lorenz, M.; Moreno-Andrés, D.; Bodenhöfer, M.; De Magistris, P.; Astrinidis, S.A.; Schooley, A.; Flötenmeyer, M.; Leptihn, S.; Antonin, W. NUP153 Recruits the NUP107-160 Complex to the Inner Nuclear Membrane for Interphasic Nuclear Pore Complex Assembly. *Dev. Cell* **2015**, *33*, 717–728, doi:10.1016/j.devcel.2015.04.027.
78. Aksenova, V.; Smith, A.; Lee, H.; Bhat, P.; Esnault, C.; Chen, S.; Iben, J.; Kaufhold, R.; Yau, K.C.; Echeverria, C.; et al. Nucleoporin TPR Is an Integral Component of the TREX-2 MRNA Export Pathway. *Nat. Commun.* **2020**, *11*, 4577, doi:10.1038/s41467-020-18266-2.
79. Lussi, Y.C.; Shumaker, D.K.; Shimi, T.; Fahrenkrog, B. The Nucleoporin NUP153 Affects Spindle Checkpoint Activity Due to an Association with MAD1. *Nucleus* **2010**, *1*, 71–84, doi:10.4161/nucl.1.1.10244.
80. Duheron, V.; Chatel, G.; Sauder, U.; Oliveri, V.; Fahrenkrog, B. Structural Characterization of Altered Nucleoporin NUP153 Expression in Human Cells by Thin-Section Electron Microscopy. *Nucleus* **2014**, *5*, 601–612, doi:10.4161/19491034.2014.990853.
81. Bodoor, K.; Shaikh, S.; Salina, D.; Raharjo, W.H.; Bastos, R.; Lohka, M.; Burke, B. Sequential Recruitment of NPC Proteins to the Nuclear Periphery at the End of Mitosis. *J. Cell Sci.* **1999**, *112*, 2253–2264.
82. Haraguchi, T.; Koujin, T.; Hayakawa, T.; Kaneda, T.; Tsutsumi, C.; Imamoto, N.; Akazawa, C.; Sukegawa, J.; Yoneda, Y.; Hiraoka, Y. Live Fluorescence Imaging Reveals Early Recruitment of Emerin, LBR, RanBP2, and NUP153 to Reforming Functional Nuclear Envelopes. *J. Cell Sci.* **2000**, *113*, 779–794.
83. Radu, A.; Blobel, G.; Wozniak, R.W. NUP155 Is a Novel Nuclear Pore Complex Protein That Contains Neither Repetitive Sequence Motifs nor Reacts with WGA. *J. Cell Biol.* **1993**, *121*, 1–9, doi:10.1083/jcb.121.1.1.

84. von Appen, A.; Kosinski, J.; Sparks, L.; Ori, A.; DiGuilio, A.L.; Vollmer, B.; Mackmull, M.-T.; Banterle, N.; Parca, L.; Kastritis, P.; et al. In Situ Structural Analysis of the Human Nuclear Pore Complex. *Nature* **2015**, *526*, 140–143, doi:10.1038/nature15381.
85. Bassermann, F.; Peschel, C.; Duyster, J. Mitotic Entry: A Matter of Oscillating Destruction. *Cell Cycle* **2005**, *4*, 1515–1517, doi:10.4161/cc.4.11.2192.
86. Bassermann, F.; Klitzing, C. von; Illert, A.L.; Münch, S.; Morris, S.W.; Pagano, M.; Peschel, C.; Duyster, J. Multisite Phosphorylation of Nuclear Interaction Partner of ALK (NIPA) at G2/M Involves Cyclin B1/Cdk1. *J. Biol. Chem.* **2007**, *282*, 15965–15972, doi:10.1074/jbc.M610819200.
87. Klitzing, C. von; Huss, R.; Illert, A.L.; Fröschl, A.; Wötzel, S.; Peschel, C.; Bassermann, F.; Duyster, J. APC/C(Cdh1)-Mediated Degradation of the F-Box Protein NIPA Is Regulated by Its Association with SKP1. *PLoS ONE* **2011**, *6*, e28998, doi:10.1371/journal.pone.0028998.
88. Illert, A.L.; Zech, M.; Moll, C.; Albers, C.; Kreutmair, S.; Peschel, C.; Bassermann, F.; Duyster, J. Extracellular Signal-Regulated Kinase 2 (ERK2) Mediates Phosphorylation and Inactivation of Nuclear Interaction Partner of Anaplastic Lymphoma Kinase (NIPA) at G2/M. *J. Biol. Chem.* **2012**, *287*, 37997–38005, doi:10.1074/jbc.M112.373464.
89. Liu, Y.-Q.; Wang, X.-L.; Cheng, X.; Lu, Y.; Wang, G.; Li, X.-C.; Zhang, J.; Wen, Z.-S.; Huang, Z.-L.; Gao, Q.-L.; et al. SKP1 in Lung Cancer: Clinical Significance and Therapeutic Efficacy of Its Small Molecule Inhibitors. *Oncotarget* **2015**, *6*, 34953–34967, doi:10.18632/oncotarget.5547.
90. Gengenbacher, A.; Müller-Rudorf, A.; Poggio, T.; Gräßel, L.; Dumit, V.I.; Kreutmair, S.; Lippert, L.J.; Duyster, J.; Illert, A.L. Proteomic Phosphosite Analysis Identified Crucial NPM-ALK-Mediated NIPA Serine and Threonine Residues. *Int. J. Mol. Sci.* **2019**, *20*, 4060, doi:10.3390/ijms20164060.
91. Lisztwan, J.; Marti, A.; Sutterlüty, H.; Gstaiger, M.; Wirbelauer, C.; Krek, W. Association of Human CUL1 and Ubiquitin-Conjugating Enzyme CDC34 with the F-Box Protein P45(SKP2): Evidence for Evolutionary Conservation in the Subunit Composition of the CDC34-SCF Pathway. *EMBO J.* **1998**, *17*, 368–383, doi:10.1093/emboj/17.2.368.
92. Lyapina, S.A.; Correll, C.C.; Kipreos, E.T.; Deshaies, R.J. Human CUL1 Forms an Evolutionarily Conserved Ubiquitin Ligase Complex (SCF) with SKP1 and an F-Box Protein. *Proc. Natl. Acad. Sci. U. S. A.* **1998**, *95*, 7451–7456, doi:10.1073/pnas.95.13.7451.
93. Michel, J.J.; Xiong, Y. Human CUL1, but Not Other Cullin Family Members, Selectively Interacts with SKP1 to Form a Complex with SKP2 and Cyclin A. *Cell Growth Differ.* **1998**, *9*, 435–449.
94. Yu, Z.K.; Gervais, J.L.M.; Zhang, H. Human CUL1 Associates with the SKP1/SKP2 Complex and Regulates P21(CIP1/WAF1) and Cyclin D Proteins. *Proc. Natl. Acad. Sci. U. S. A.* **1998**, *95*, 11324–11329, doi:10.1073/pnas.95.19.11324.
95. Ng, R.W.M.; Arooz, T.; Yam, C.H.; Chan, I.W.Y.; Lau, A.W.S.; Poon, R.Y.C. Characterization of the Cullin and F-Box Protein Partner SKP1. *FEBS Lett.* **1998**, *438*, 183–189, doi:10.1016/s0014-5793(98)01299-x.
96. Schulman, B.A.; Carrano, A.C.; Jeffrey, P.D.; Bowen, Z.; Kinnucan, E.R.E.; Finnin, M.S.; Elledge, S.J.; Harper, J.W.; Pagano, M.; Pavletich, N.P. Insights into SCF Ubiquitin Ligases from the Structure of the Skp1-Skp2 Complex. *Nature* **2000**, *408*, 381–386, doi:10.1038/35042620.
97. Duyster, J.; Baskaran, R.; Wang, J.Y. Src Homology 2 Domain as a Specificity Determinant in the C-Abl-Mediated Tyrosine Phosphorylation of the RNA Polymerase II Carboxyl-Terminal Repeated Domain. *Proc. Natl. Acad. Sci. U. S. A.* **1995**, *92*, 1555–1559, doi:10.1073/pnas.92.5.1555.
98. Wang, J.; Shan, J.; Xu, Q.; Ruan, X.; Gong, Y.; Kuang, T.; Zhao, N. Spectroscopic Study of Trypsin, Heat and Triton X-100-Induced Denaturation of the Chlorophyll-Binding Protein CP43. *J. Photochem. Photobiol. B.* **2000**, *58*, 136–142, doi:10.1016/s1011-1344(00)00118-4.
99. Singh, S.K.; Kishore, N. Thermodynamic Insights into the Binding of Triton X-100 to Globular Proteins: A Calorimetric and Spectroscopic Investigation. *J. Phys. Chem. B* **2006**, *110*, 9728–9737, doi:10.1021/jp0608426.
100. Hopp, T.P.; Prickett, K.S.; Price, V.L.; Libby, R.T.; March, C.J.; Cerretti, D.P.; Urdal, D.L.; Conlon, P.J. A Short Polypeptide Marker Sequence Useful for Recombinant Protein Identification and Purification. *Bio/Technology* **1988**, *6*, 1204–1210, doi:10.1038/nbt1088-1204.
101. Rothbauer, U.; Zolghadr, K.; Muyldermans, S.; Schepers, A.; Cardoso, M.C.; Leonhardt, H. A Versatile Nanotrap for Biochemical and Functional Studies with Fluorescent Fusion Proteins. *Mol. Cell. proteomics* **2008**, *7*, 282–289, doi:10.1074/mcp.M700342-MCP200.
102. Schunkert, H.; König, I.R.; Kathiresan, S.; Reilly, M.P.; Assimes, T.L.; Holm, H.; Preuss, M.; Stewart, A.F.R.; Barbalic, M.; Gieger, C.; et al. Large-Scale Association Analysis Identifies 13 New Susceptibility Loci for Coronary Artery Disease. *Nat. Genet.* **2011**, *43*, 333–338, doi:10.1038/ng.784.
103. López-Mejías, R.; Genre, F.; García-Bermúdez, M.; Corrales, A.; González-Juanatey, C.; Llorca, J.; Miranda-Filloy, J.A.; Rueda-Gotor, J.; Blanco, R.; Castañeda, S.; et al. The ZC3HC1 Rs11556924 Polymorphism Is Associated with Increased Carotid Intima-Media Thickness in Patients with Rheumatoid Arthritis. *Arthritis Res. Ther.* **2013**, *15*, R152, doi:10.1186/ar4335.
104. Dichgans, M.; Malik, R.; König, I.R.; Rosand, J.; Clarke, R.; Gretarsdottir, S.; Thorleifsson, G.; Mitchell, B.D.; Assimes, T.L.; Levi, C.; et al. Shared Genetic Susceptibility to Ischemic Stroke and Coronary Artery Disease: A Genome-Wide Analysis of Common Variants. *Stroke* **2014**, *45*, 24–36, doi:10.1161/STROKEAHA.113.002707.
105. Kunnas, T.; Nikkari, S.T. Association of Zinc Finger, C3HC-Type Containing 1 (ZC3HC1) Rs11556924 Genetic Variant with Hypertension in a Finnish Population, the TAMRISK Study. *Medicine (Baltimore).* **2015**, *94*, e1221, doi:10.1097/MD.0000000000001221.

106. Jones, P.D.; Kaiser, M.A.; Ghaderi Najafabadi, M.; McVey, D.G.; Beveridge, A.J.; Schofield, C.L.; Samani, N.J.; Webb, T.R. The Coronary Artery Disease-Associated Coding Variant in Zinc Finger C3HC-Type Containing 1 (ZC3HC1) Affects Cell Cycle Regulation. *J. Biol. Chem.* **2016**, *291*, 16318–16327, doi:10.1074/jbc.M116.734020.
107. Wirtwein, M.; Melander, O.; Sjőgren, M.; Hoffmann, M.; Narkiewicz, K.; Gruchala, M.; Sobiczewski, W. The Relationship between Gene Polymorphisms and Dipping Profile in Patients with Coronary Heart Disease. *Am. J. Hypertens.* **2016**, *29*, 1094–1102, doi:10.1093/ajh/hpw040.
108. Yamase, Y.; Kato, K.; Horibe, H.; Ueyama, C.; Fujimaki, T.; Oguri, M.; Arai, M.; Watanabe, S.; Murohara, T.; Yamada, Y. Association of Genetic Variants with Atrial Fibrillation. *Biomed. reports* **2016**, *4*, 178–182, doi:10.3892/br.2015.551.
109. Linseman, T.; Soubeyrand, S.; Martinuk, A.; Nikpay, M.; Lau, P.; McPherson, R. Functional Validation of a Common Nonsynonymous Coding Variant in ZC3HC1 Associated with Protection from Coronary Artery Disease. *Circ. Cardiovasc. Genet.* **2017**, *10*, e001498, doi:10.1161/CIRCGENETICS.116.001498.
110. Henzl, M.T.; Thalmann, I.; Thalmann, R. OCP2 Exists as a Dimer in the Organ of Corti. *Hear. Res.* **1998**, *126*, 37–46, doi:10.1016/s0378-5955(98)00148-8.
111. Sheikh, M.O.; Schafer, C.M.; Powell, J.T.; Rodgers, K.K.; Mooers, B.H.M.; West, C.M. Glycosylation of SKP1 Affects Its Conformation and Promotes Binding to a Model F-Box Protein. *Biochemistry* **2014**, *53*, 1657–1669, doi:10.1021/bi401707y.
112. Kim, H.W.; Eletsky, A.; Gonzalez, K.J.; van der Wel, H.; Strauch, E.-M.; Prestegard, J.H.; West, C.M. SKP1 Dimerization Conceals Its F-Box Protein Binding Site. *Biochemistry* **2020**, *59*, 1527–1536, doi:10.1021/acs.biochem.0c00094.
113. Illert, A.L.; Kawaguchi, H.; Antinozzi, C.; Bassermann, F.; Quintanilla-Martinez, L.; Klitzing, C. von; Hiwatari, M.; Peschel, C.; de Rooij, D.G.; Morris, S.W.; et al. Targeted Inactivation of Nuclear Interaction Partner of ALK Disrupts Meiotic Prophase. *Development* **2012**, *139*, 2523–2534, doi:10.1242/dev.073072.
114. Hoshino, R.; Chatani, Y.; Yamori, T.; Tsuruo, T.; Oka, H.; Yoshida, O.; Shimada, Y.; Ari-i, S.; Wada, H.; Fujimoto, J.; et al. Constitutive Activation of the 41-/43-KDa Mitogen-Activated Protein Kinase Signaling Pathway in Human Tumors. *Oncogene* **1999**, *18*, 813–822, doi:10.1038/sj.onc.1202367.
115. Chambard, J.-C.; Lefloch, R.; Pouysségur, J.; Lenormand, P. ERK Implication in Cell Cycle Regulation. *Biochim. Biophys. Acta* **2007**, *1773*, 1299–1310, doi:10.1016/j.bbamcr.2006.11.010.
116. Favata, M.F.; Horiuchi, K.Y.; Manos, E.J.; Daulerio, A.J.; Stradley, D.A.; Feeser, W.S.; Van Dyk, D.E.; Pitts, W.J.; Earl, R.A.; Hobbs, F.; et al. Identification of a Novel Inhibitor of Mitogen-Activated Protein Kinase Kinase. *J. Biol. Chem.* **1998**, *273*, 18623–18632, doi:10.1074/jbc.273.29.18623.
117. Geiger, T.; Wehner, A.; Schaab, C.; Cox, J.; Mann, M. Comparative Proteomic Analysis of Eleven Common Cell Lines Reveals Ubiquitous but Varying Expression of Most Proteins. *Mol. Cell. Proteomics* **2012**, *11*, M111.014050, doi:10.1074/mcp.M111.014050.
118. Bekker-Jensen, D.B.; Kelstrup, C.D.; Batth, T.S.; Larsen, S.C.; Haldrup, C.; Bramsen, J.B.; Sørensen, K.D.; Høyer, S.; Ørntoft, T.F.; Andersen, C.L.; et al. An Optimized Shotgun Strategy for the Rapid Generation of Comprehensive Human Proteomes. *Cell Syst.* **2017**, *4*, 587-599.e4, doi:10.1016/j.cels.2017.05.009.
119. Pan, Z.-Q.; Kentsis, A.; Dias, D.C.; Yamoah, K.; Wu, K. Nedd8 on Cullin: Building an Expressway to Protein Destruction. *Oncogene* **2004**, *23*, 1985–1997, doi:10.1038/sj.onc.1207414.
120. Ouyang, T.; Bai, R.-Y.; Bassermann, F.; Klitzing, C. von; Klumpen, S.; Miething, C.; Morris, S.W.; Peschel, C.; Duyster, J. Identification and Characterization of a Nuclear Interacting Partner of Anaplastic Lymphoma Kinase (NIPA). *J. Biol. Chem.* **2003**, *278*, 30028–30036, doi:10.1074/jbc.M300883200.
121. Fischle, W.; Tseng, B.S.; Dormann, H.L.; Ueberheide, B.M.; Garcia, B.A.; Shabanowitz, J.; Hunt, D.F.; Funabiki, H.; Allis, C.D. Regulation of HP1-Chromatin Binding by Histone H3 Methylation and Phosphorylation. *Nature* **2005**, *438*, 1116–1122, doi:10.1038/nature04219.
122. Hirota, T.; Lipp, J.J.; Toh, B.-H.; Peters, J.-M. Histone H3 Serine 10 Phosphorylation by Aurora B Causes HP1 Dissociation from Heterochromatin. *Nature* **2005**, *438*, 1176–1180, doi:10.1038/nature04254.
123. Sawicka, A.; Seiser, C. Histone H3 Phosphorylation - a Versatile Chromatin Modification for Different Occasions. *Biochimie* **2012**, *94*, 2193–2201, doi:10.1016/j.biochi.2012.04.018.
124. Toyoshima, F.; Moriguchi, T.; Wada, A.; Fukuda, M.; Nishida, E. Nuclear Export of Cyclin B1 and Its Possible Role in the DNA Damage-Induced G2 Checkpoint. *EMBO J.* **1998**, *17*, 2728–2735, doi:10.1093/emboj/17.10.2728.
125. Yang, J.; Bardes, E.S.G.; Moore, J.D.; Brennan, J.; Powers, M.A.; Kornbluth, S. Control of Cyclin B1 Localization through Regulated Binding of the Nuclear Export Factor CRM1. *Genes Dev.* **1998**, *12*, 2131–2143, doi:10.1101/gad.12.14.2131.
126. Hagting, A.; Karlsson, C.; Clute, P.; Jackman, M.; Pines, J. MPF Localization Is Controlled by Nuclear Export. *EMBO J.* **1998**, *17*, 4127–4138, doi:10.1093/emboj/17.14.4127.
127. Maity, A.; McKenna, W.G.; Muschel, R.J. Evidence for Post-Transcriptional Regulation of Cyclin B1 MRNA in the Cell Cycle and Following Irradiation in HeLa Cells. *EMBO J.* **1995**, *14*, 603–609.
128. Takizawa, C.G.; Weis, K.; Morgan, D.O. Ran-Independent Nuclear Import of Cyclin B1-Cdc2 by Importin Beta. *Proc. Natl. Acad. Sci.* **1999**, *96*, 7938–7943, doi:10.1073/pnas.96.14.7938.
129. Moore, J.D.; Yang, J.; Truant, R.; Kornbluth, S. Nuclear Import of Cdk/Cyclin Complexes: Identification of Distinct Mechanisms for Import of Cdk2/Cyclin E and Cdc2/Cyclin B1. *J. Cell Biol.* **1999**, *144*, 213–224, doi:10.1083/jcb.144.2.213.
130. Hagting, A.; Jackman, M.; Simpson, K.; Pines, J. Translocation of Cyclin B1 to the Nucleus at Prophase Requires a Phosphorylation-Dependent Nuclear Import Signal. *Curr. Biol.* **1999**, *9*, 680–689, doi:10.1016/s0960-9822(99)80308-x.

131. Ferrando-May, E.; Cordes, V.; Biller-Ckovric, I.; Mirkovic, J.; Görlich, D.; Nicotera, P. Caspases Mediate Nucleoporin Cleavage, but Not Early Redistribution of Nuclear Transport Factors and Modulation of Nuclear Permeability in Apoptosis. *Cell Death Differ.* **2001**, *8*, 495–505, doi:10.1038/sj.cdd.4400837.
132. Patre, M.; Tabbert, A.; Hermann, D.; Walczak, H.; Rackwitz, H.-R.; Cordes, V.C.; Ferrando-May, E. Caspases Target Only Two Architectural Components within the Core Structure of the Nuclear Pore Complex. *J. Biol. Chem.* **2006**, *281*, 1296–1304, doi:10.1074/jbc.M511717200.
133. Scacheri, P.C.; Rozenblatt-Rosen, O.; Caplen, N.J.; Wolfsberg, T.G.; Umayam, L.; Lee, J.C.; Hughes, C.M.; Shanmugam, K.S.; Bhattacharjee, A.; Meyerson, M.; et al. Short Interfering RNAs Can Induce Unexpected and Divergent Changes in the Levels of Untargeted Proteins in Mammalian Cells. *Proc. Natl. Acad. Sci. U. S. A.* **2004**, *101*, 1892–1897, doi:10.1073/pnas.0308698100.
134. Kurreck, J. Expediting Target Identification and Validation through RNAi. *Expert Opin. Biol. Ther.* **2004**, *4*, 427–429, doi:10.1517/14712598.4.3.427.
135. Tschuch, C.; Schulz, A.; Pscherer, A.; Werft, W.; Benner, A.; Hotz-Wagenblatt, A.; Barrionuevo, L.S.; Lichter, P.; Mertens, D. Off-Target Effects of SiRNA Specific for GFP. *BMC Mol. Biol.* **2008**, *9*, 60, doi:10.1186/1471-2199-9-60.
136. Moses, J.; Goodchild, A.; Rivory, L.P. Intended Transcriptional Silencing with SiRNA Results in Gene Repression through Sequence-Specific off-Targeting. *RNA* **2010**, *16*, 430–441, doi:10.1261/rna.1808510.
137. Sigoillot, F.D.; King, R.W. Vigilance and Validation: Keys to Success in RNAi Screening. *ACS Chem. Biol.* **2011**, *6*, 47–60, doi:10.1021/cb100358f.
138. Jackson, A.L.; Bartz, S.R.; Schelter, J.; Kobayashi, S. V; Burchard, J.; Mao, M.; Li, B.; Cavet, G.; Linsley, P.S. Expression Profiling Reveals Off-Target Gene Regulation by RNAi. *Nat. Biotechnol.* **2003**, *21*, 635–637, doi:10.1038/nbt831.
139. Anonymous Whither RNAi? *Nat. Cell Biol.* **2003**, *5*, 489–490, doi:10.1038/ncb0603-490.
140. Lazebnik, Y.A.; Kaufmann, S.H.; Desnoyers, S.; Poirier, G.G.; Earnshaw, W.C. Cleavage of Poly(ADP-Ribose) Polymerase by a Proteinase with Properties like ICE. *Nature* **1994**, *371*, 346–347, doi:10.1038/371346a0.
141. Nuñez, G.; Benedict, M.A.; Hu, Y.; Inohara, N. Caspases: The Proteases of the Apoptotic Pathway. *Oncogene* **1998**, *17*, 3237–3245, doi:10.1038/sj.onc.1202581.
142. Nagaraj, N.; Wisniewski, J.R.; Geiger, T.; Cox, J.; Kircher, M.; Kelso, J.; Pääbo, S.; Mann, M. Deep Proteome and Transcriptome Mapping of a Human Cancer Cell Line. *Mol. Syst. Biol.* **2011**, *7*, 548, doi:10.1038/msb.2011.81.
143. Gorczyca, W.; Gong, J.; Darzynkiewicz, Z. Detection of DNA Strand Breaks in Individual Apoptotic Cells by the in Situ Terminal Deoxynucleotidyl Transferase and Nick Translation Assays. *Cancer Res.* **1993**, *53*, 1945–1951, doi:10.1016/j.semcdb.2008.10.002.
144. Weil, M.; Jacobson, M.D.; Coles, H.S.; Davies, T.J.; Gardner, R.L.; Raff, K.D.; Raff, M.C. Constitutive Expression of the Machinery for Programmed Cell Death. *J. Cell Biol.* **1996**, *133*, 1053–1059, doi:10.1083/jcb.133.5.1053.
145. Palchaudhuri, R.; Lambrecht, M.J.; Botham, R.C.; Partlow, K.C.; van Ham, T.J.; Putt, K.S.; Nguyen, L.T.; Kim, S.-H.; Peterson, R.T.; Fan, T.M.; et al. A Small Molecule That Induces Intrinsic Pathway Apoptosis with Unparalleled Speed. *Cell Rep.* **2015**, *13*, 2027–2036, doi:10.1016/j.celrep.2015.10.042.
146. Cordes, V.C.; Hase, M.E.; Müller, L. Molecular Segments of Protein Tpr That Confer Nuclear Targeting and Association with the Nuclear Pore Complex. *Exp. Cell Res.* **1998**, *245*, 43–56, doi:10.1006/excr.1998.4246.
147. Gerdes, J.; Lemke, H.; Baisch, H.; Wacker, H.H.; Schwab, U.; Stein, H. Cell Cycle Analysis of a Cell Proliferation-Associated Human Nuclear Antigen Defined by the Monoclonal Antibody Ki-67. *J. Immunol.* **1984**, *133*, 1710–1715.
148. Heidebrecht, H.J.; Buck, F.; Haas, K.; Wacker, H.H.; Parwaresch, R. Monoclonal Antibodies Ki-S3 and Ki-S5 Yield New Data on the "Ki-67" Proteins. *Cell Prolif.* **1996**, *29*, 413–425, doi:10.1111/j.1365-2184.1996.tb00984.x.
149. Bruno, S.; Darzynkiewicz, Z. Cell Cycle Dependent Expression and Stability of the Nuclear Protein Detected by Ki-67 Antibody in HL-60 Cells. *Cell Prolif.* **1992**, *25*, 31–40, doi:10.1111/j.1365-2184.1992.tb01435.x.
150. Brown, D.C.; Gatter, K.C. Ki67 Protein: The Immaculate Deception? *Histopathology* **2002**, *40*, 2–11, doi:10.1046/j.1365-2559.2002.01343.x.
151. Thomas, J.O.; Kornberg, R.D. An Octamer of Histones in Chromatin and Free in Solution. *Proc. Natl. Acad. Sci. U. S. A.* **1975**, *72*, 2626–2630, doi:10.1073/pnas.72.7.2626.
152. Love, D.C.; Sweitzer, T.D.; Hanover, J.A. Reconstitution of HIV-1 Rev Nuclear Export: Independent Requirements for Nuclear Import and Export. *Proc. Natl. Acad. Sci. U. S. A.* **1998**, *95*, 10608–10613, doi:10.1073/pnas.95.18.10608.
153. Plafker, K.; Macara, I.G. Facilitated Nucleocytoplasmic Shuttling of the Ran Binding Protein RanBP1. *Mol. Cell. Biol.* **2000**, *20*, 3510–3521, doi:10.1128/mcb.20.10.3510-3521.2000.
154. Pontén, J.; Saksela, E. Two Established in Vitro Cell Lines from Human Mesenchymal Tumours. *Int. J. cancer* **1967**, *2*, 434–447, doi:10.1002/ijc.2910020505.
155. Ben-Shoshan, S.O.; Simon, A.J.; Jacob-Hirsch, J.; Shaklai, S.; Paz-Yaacov, N.; Amariglio, N.; Rechavi, G.; Trakhtenbrot, L. Induction of Polyploidy by Nuclear Fusion Mechanism upon Decreased Expression of the Nuclear Envelope Protein LAP2β in the Human Osteosarcoma Cell Line U2OS. *Mol. Cytogenet.* **2014**, *7*, 9, doi:10.1186/1755-8166-7-9.
156. Raftopoulou, C.; Roumelioti, F.-M.; Dragona, E.; Gimelli, S.; Sloan-Béna, F.; Gorgoulis, V.; Antonarakis, S.E.; Gagos, S. Karyotypic Flexibility of the Complex Cancer Genome and the Role of Polyploidization in Maintenance of Structural Integrity of Cancer Chromosomes. *Cancers (Basel).* **2020**, *12*, 1–15, doi:10.3390/cancers12030591.
157. Ran, F.A.; Hsu, P.D.; Lin, C.-Y.; Gootenberg, J.S.; Konermann, S.; Trevino, A.E.; Scott, D.A.; Inoue, A.; Matoba, S.; Zhang, Y.; et al. Double Nicking by RNA-Guided CRISPR Cas9 for Enhanced Genome Editing Specificity. *Cell* **2013**, *154*, 1380–1389, doi:10.1016/j.cell.2013.08.021.

158. Ben-Efraim, I.; Frosst, P.D.; Gerace, L. Karyopherin Binding Interactions and Nuclear Import Mechanism of Nuclear Pore Complex Protein TPR. *BMC Cell Biol.* **2009**, *10*, 74, doi:10.1186/1471-2121-10-74.
159. Higashi, K.; Takasawa, R.; Yoshimori, A.; Goh, T.; Tanuma, S.; Kuchitsu, K. Identification of a Novel Gene Family, Paralogs of Inhibitor of Apoptosis Proteins Present in Plants, Fungi, and Animals. *Apoptosis* **2005**, *10*, 471–480, doi:10.1007/s10495-005-1876-1.
160. Garimella, S. V; Gehlhaus, K.; Dine, J.L.; Pitt, J.J.; Grandin, M.; Chakka, S.; Nau, M.M.; Caplen, N.J.; Lipkowitz, S. Identification of Novel Molecular Regulators of Tumor Necrosis Factor-Related Apoptosis-Inducing Ligand (TRAIL)-Induced Apoptosis in Breast Cancer Cells by RNAi Screening. *Breast Cancer Res.* **2014**, *16*, R41, doi:10.1186/bcr3645.
161. Kimura, J.; Nguyen, S.T.; Liu, H.; Taira, N.; Miki, Y.; Yoshida, K. A Functional Genome-Wide RNAi Screen Identifies TAF1 as a Regulator for Apoptosis in Response to Genotoxic Stress. *Nucleic Acids Res.* **2008**, *36*, 5250–5259, doi:10.1093/nar/gkn506.
162. Sudo, H.; Tsuji, A.B.; Sugyo, A.; Kohda, M.; Sogawa, C.; Yoshida, C.; Harada, Y.; Hino, O.; Saga, T. Knockdown of COPA, Identified by Loss-of-Function Screen, Induces Apoptosis and Suppresses Tumor Growth in Mesothelioma Mouse Model. *Genomics* **2010**, *95*, 210–216, doi:10.1016/j.ygeno.2010.02.002.
163. Eißmann, M.; Schwamb, B.; Melzer, I.M.; Moser, J.; Siele, D.; Köhl, U.; Rieker, R.J.; Wachter, D.L.; Agaimy, A.; Herpel, E.; et al. A Functional Yeast Survival Screen of Tumor-Derived CDNA Libraries Designed to Identify Anti-Apoptotic Mammalian Oncogenes. *PLoS ONE* **2013**, *8*, e64873, doi:10.1371/journal.pone.0064873.
164. Sousa, M.; Duarte, A.M.; Fernandes, T.R.; Chaves, S.R.; Pacheco, A.; Leão, C.; Côrte-Real, M.; Sousa, M.J. Genome-Wide Identification of Genes Involved in the Positive and Negative Regulation of Acetic Acid-Induced Programmed Cell Death in Saccharomyces Cerevisiae. *BMC Genomics* **2013**, *14*, 838, doi:10.1186/1471-2164-14-838.
165. Kokoszynska, K.; Rychlewski, L.; Wyrwicz, L.S. The Mitotic Entry Regulator NIPA Is a Prototypic BIR Domain Protein. *Cell cycle* **2008**, *7*, 2073–2075, doi:10.4161/cc.7.13.6237.
166. Portt, L.; Norman, G.; Clapp, C.; Greenwood, M.; Greenwood, M.T. Anti-Apoptosis and Cell Survival: A Review. *Biochim. Biophys. Acta* **2011**, *1813*, 238–259, doi:10.1016/j.bbamcr.2010.10.010.
167. Silke, J.; Meier, P. Inhibitor of Apoptosis (IAP) Proteins-Modulators of Cell Death and Inflammation. *Cold Spring Harb. Perspect. Biol.* **2013**, *5*, a008730, doi:10.1101/cshperspect.a008730.
168. de Rooij, D.G.; de Boer, P. Specific Arrests of Spermatogenesis in Genetically Modified and Mutant Mice. *Cytogenet. Genome Res.* **2003**, *103*, 267–276, doi:10.1159/000076812.
169. Shaha, C.; Tripathi, R.; Mishra, D.P. Male Germ Cell Apoptosis: Regulation and Biology. *Philos. Trans. R. Soc. Lond. B. Biol. Sci.* **2010**, *365*, 1501–1515, doi:10.1098/rstb.2009.0124.
170. Bai, C.; Sen, P.; Hofmann, K.; Ma, L.; Goebl, M.; Harper, J.W.; Elledge, S.J. SKP1 Connects Cell Cycle Regulators to the Ubiquitin Proteolysis Machinery through a Novel Motif, the F-Box. *Cell* **1996**, *86*, 263–274, doi:10.1016/s0092-8674(00)80098-7.
171. Cardozo, T.; Pagano, M. The SCF Ubiquitin Ligase: Insights into a Molecular Machine. *Nat. Rev. Mol. Cell Biol.* **2004**, *5*, 739–751, doi:10.1038/nrm1471.
172. Wu, G.; Xu, G.; Schulman, B.A.; Jeffrey, P.D.; Harper, J.W.; Pavletich, N.P. Structure of a Beta-TrCP1-Skp1-Beta-Catenin Complex: Destruction Motif Binding and Lysine Specificity of the SCF(Beta-TrCP1) Ubiquitin Ligase. *Mol. Cell* **2003**, *11*, 1445–1456, doi:10.1016/s1097-2765(03)00234-x.
173. Lin, S.-C.; Huang, Y.; Lo, Y.-C.; Lu, M.; Wu, H. Crystal Structure of the BIR1 Domain of XIAP in Two Crystal Forms. *J. Mol. Biol.* **2007**, *372*, 847–854, doi:10.1016/j.jmb.2007.07.019.
174. Ramachandran, S.; Kota, P.; Ding, F.; Dokholyan, N. V Automated Minimization of Steric Clashes in Protein Structures. *Proteins* **2011**, *79*, 261–270, doi:10.1002/prot.22879.
175. Fields, S. High-Throughput Two-Hybrid Analysis. The Promise and the Peril. *FEBS J.* **2005**, *272*, 5391–5399, doi:10.1111/j.1742-4658.2005.04973.x.
176. Huang, H.; Jedynak, B.M.; Bader, J.S. Where Have All the Interactions Gone? Estimating the Coverage of Two-Hybrid Protein Interaction Maps. *PLoS Comput. Biol.* **2007**, *3*, e214, doi:10.1371/journal.pcbi.0030214.
177. Luck, K.; Kim, D.-K.; Lambourne, L.; Spirohn, K.; Begg, B.E.; Bian, W.; Brignall, R.; Cafarelli, T.; Campos-Laborie, F.J.; Charloteaux, B.; et al. A Reference Map of the Human Binary Protein Interactome. *Nature* **2020**, *580*, 402–408, doi:10.1038/s41586-020-2188-x.
178. Huttlin, E.L.; Ting, L.; Bruckner, R.J.; Gebreab, F.; Gygi, M.P.; Szpyt, J.; Tam, S.; Zarraga, G.; Colby, G.; Baltier, K.; et al. The BioPlex Network: A Systematic Exploration of the Human Interactome. *Cell* **2015**, *162*, 425–440, doi:10.1016/j.cell.2015.06.043.
179. Risseeuw, E.P.; Daskalchuk, T.E.; Banks, T.W.; Liu, E.; Cotelesage, J.; Hellmann, H.; Estelle, M.; Somers, D.E.; Crosby, W.L. Protein Interaction Analysis of SCF Ubiquitin E3 Ligase Subunits from Arabidopsis. *Plant. J.* **2003**, *34*, 753–767, doi:10.1046/j.1365-313x.2003.01768.x.
180. Seol, J.H.; Shevchenko, A.; Shevchenko, A.; Deshaies, R.J. SKP1 Forms Multiple Protein Complexes, Including RAVE, a Regulator of V-ATPase Assembly. *Nat. Cell Biol.* **2001**, *3*, 384–391, doi:10.1038/35070067.
181. Sheikh, M.O.; Xu, Y.; van der Wel, H.; Walden, P.; Hartson, S.D.; West, C.M. Glycosylation of SKP1 Promotes Formation of SKP1-Cullin-1-F-Box Protein Complexes in Dictyostelium. *Mol. Cell. Proteomics* **2015**, *14*, 66–80, doi:10.1074/mcp.M114.044560.
182. Kittler, R.; Pelletier, L.; Heninger, A.-K.; Slabicki, M.; Theis, M.; Miroslaw, L.; Poser, I.; Lawo, S.; Grabner, H.; Kozak, K.; et al. Genome-Scale RNAi Profiling of Cell Division in Human Tissue Culture Cells. *Nat. Cell Biol.* **2007**, *9*, 1401–1412, doi:10.1038/ncb1659.

183. Kırlı, K.; Karaca, S.; Dehne, H.J.; Samwer, M.; Pan, K.T.; Lenz, C.; Urlaub, H.; Görlich, D. A Deep Proteomics Perspective on CRM1-Mediated Nuclear Export and Nucleocytoplasmic Partitioning. *Elife* **2015**, *4*, e11466, doi:10.7554/eLife.11466.
184. Güttler, T.; Görlich, D. Ran-Dependent Nuclear Export Mediators: A Structural Perspective. *EMBO J.* **2011**, *30*, 3457–3474, doi:10.1038/emboj.2011.287.
185. Niepel, M.; Molloy, K.R.; Williams, R.; Farr, J.C.; Meinema, A.C.; Vecchietti, N.; Cristea, I.M.; Chait, B.T.; Rout, M.P.; Strambio-De-Castillia, C. The Nuclear Basket Proteins Mlp1p and Mlp2p Are Part of a Dynamic Interactome Including Esc1p and the Proteasome. *Mol. Biol. Cell* **2013**, *24*, 3920–3938, doi:10.1091/mbc.E13-07-0412.
186. Salas-Pino, S.; Gallardo, P.; Barrales, R.R.; Braun, S.; Daga, R.R. The Fission Yeast Nucleoporin Alm1 Is Required for Proteasomal Degradation of Kinetochore Components. *J. Cell Biol.* **2017**, *216*, 3591–3608, doi:10.1083/jcb.201612194.
187. Albert, S.; Schaffer, M.; Beck, F.; Mosalaganti, S.; Asano, S.; Thomas, H.F.; Plitzko, J.M.; Beck, M.; Baumeister, W.; Engel, B.D. Proteasomes Tether to Two Distinct Sites at the Nuclear Pore Complex. *Proc. Natl. Acad. Sci. U. S. A.* **2017**, *114*, 13726–13731, doi:10.1073/pnas.1716305114.
188. Gallardo, P.; Salas-Pino, S.; Daga, R.R. A New Role for the Nuclear Basket Network. *Microb. cell* **2017**, *4*, 423–425, doi:10.15698/mic2017.12.604.
189. Mahamid, J.; Pfeffer, S.; Schaffer, M.; Villa, E.; Danev, R.; Cuellar, L.K.; Förster, F.; Hyman, A.A.; Plitzko, J.M.; Baumeister, W. Visualizing the Molecular Sociology at the HeLa Cell Nuclear Periphery. *Science* **2016**, *351*, 969–972, doi:10.1126/science.aad8857.
190. Palmer, R.H.; Vernersson, E.; Grabbe, C.; Hallberg, B. Anaplastic Lymphoma Kinase: Signalling in Development and Disease. *Biochem. J.* **2009**, *420*, 345–361, doi:10.1042/BJ20090387.
191. Bischof, D.; Pulford, K.; Mason, D.Y.; Morris, S.W. Role of the Nucleophosmin (NPM) Portion of the Non-Hodgkin's Lymphoma-Associated NPM-Anaplastic Lymphoma Kinase Fusion Protein in Oncogenesis. *Mol. Cell. Biol.* **1997**, *17*, 2312–2325, doi:10.1128/MCB.17.4.2312.
192. Choi, Y.-L.; Lira, M.E.; Hong, M.; Kim, R.N.; Choi, S.-J.; Song, J.-Y.; Pandy, K.; Mann, D.L.; Stahl, J.A.; Peckham, H.E.; et al. A Novel Fusion of TPR and ALK in Lung Adenocarcinoma. *J. Thorac. Oncol.* **2014**, *9*, 563–566, doi:10.1097/JTO.0000000000000093.
193. Du, X.; Shao, Y.; Gao, H.; Zhang, X.; Zhang, H.; Ban, Y.; Qin, H.; Tai, Y. CMTR1-ALK: An ALK Fusion in a Patient with No Response to ALK Inhibitor Crizotinib. *Cancer Biol. Ther.* **2018**, *19*, 962–966, doi:10.1080/15384047.2018.1480282.
194. Krohne, G.; Franke, W.W. Proteins of Pore Complex - Lamina Structures from Nuclei and Nuclear Membranes. *Methods Enzymol.* **1983**, *96*, 597–608, doi:10.1016/s0076-6879(83)96052-4.
195. Steinbrecht, R.A.; Müller, M. Freeze-Substitution and Freeze-Drying. In *Cryotechniques in Biological Electron Microscopy*; Springer Berlin Heidelberg: Berlin, Heidelberg, 1987; pp. 149–172.
196. Buser, C.; Walther, P. Freeze-Substitution: The Addition of Water to Polar Solvents Enhances the Retention of Structure and Acts at Temperatures around -60 Degrees C. *J. Microsc.* **2008**, *230*, 268–277, doi:10.1111/j.1365-2818.2008.01984.x.
197. Blow, J.J.; Laskey, R.A. Initiation of DNA Replication in Nuclei and Purified DNA by a Cell-Free Extract of Xenopus Eggs. *Cell* **1986**, *47*, 577–587.
198. Morrill, G.A.; Robbins, E. Changes in Intracellular Cations during the Cell Cycle in HeLa Cells. *Physiol. Chem. Phys. Med. NMR* **1984**, *16*, 209–219.
199. Kurose, A.; Tanaka, T.; Huang, X.; Traganos, F.; Darzynkiewicz, Z. Synchronization in the Cell Cycle by Inhibitors of DNA Replication Induces Histone H2AX Phosphorylation: An Indication of DNA Damage. *Cell Prolif.* **2006**, *39*, 231–240, doi:10.1111/j.1365-2184.2006.00380.x.
200. Reed, S.E.; Staley, E.M.; Mayginnes, J.P.; Pintel, D.J.; Tullis, G.E. Transfection of Mammalian Cells Using Linear Polyethylenimine Is a Simple and Effective Means of Producing Recombinant Adeno-Associated Virus Vectors. *J. Virol. Methods* **2006**, *138*, 85–98, doi:10.1016/j.jviromet.2006.07.024.
201. Aricescu, A.R.; Lu, W.; Jones, E.Y. A Time- and Cost-Efficient System for High-Level Protein Production in Mammalian Cells. *Acta Crystallogr. Sect. D Biol. Crystallogr.* **2006**, *62*, 1243–1250, doi:10.1107/S0907444906029799.
202. Cordes, V.C.; Reidenbach, S.; Franke, W.W. Cytoplasmic Annulate Lamellae in Cultured Cells: Composition, Distribution, and Mitotic Behavior. *Cell Tissue Res.* **1996**, *284*, 177–191, doi:10.1007/s004410050578.
203. Ran, F.A.; Hsu, P.D.; Wright, J.; Agarwala, V.; Scott, D.A.; Zhang, F. Genome Engineering Using the CRISPR-Cas9 System. *Nat. Protoc.* **2013**, *8*, 2281–2308, doi:10.1038/nprot.2013.143.
204. Brand, A.; Allen, L.; Altman, M.; Hlava, M.; Scott, J. Beyond Authorship: Attribution, Contribution, Collaboration, and Credit. *Learn. Publ.* **2015**, *28*, 151–155, doi:10.1087/20150211.
205. Contributor Role Taxonomy (CRediT). Available online: http://casrai.org/CRediT (https://web.archive.org/web/20210712104751/https://casrai.org/credit/; archived on 12 July 2021) (accessed on 12 July 2021)
